# Preliminary species diversity and community phylogenetics of wood-inhabiting basidiomycetous fungi in the Dabie Mountains, Central China reveal unexpected richness

**DOI:** 10.1186/s43008-023-00130-9

**Published:** 2023-11-14

**Authors:** Xiang-Yang Liu, Shi-Liang Liu, Hao-Wen Wei, Xue-Wei Wang, Jia Yu, Shan Shen, Li-Wei Zhou

**Affiliations:** 1 State Key Laboratory of Mycology, Institute of Microbiology, Chinese Academy of Sciences, Beijing, 100101 People’s Republic of China; 2 College of Life Science, Liaoning University, Shenyang, 110036 People’s Republic of China; 3 University of Chinese Academy of Sciences, Beijing, 100049 People’s Republic of China

**Keywords:** *Basidiomycota*, Conservation, Funga, Macrofungi, Taxonomy

## Abstract

Wood-inhabiting fungi have important economic values as well as playing a major ecological role in forest ecosystem cycles. The Dabie Mountains, at the junction of Henan, Hubei, and Anhui Provinces, Central China, provide an ideal climate and favorable niches for the speciation and diversification of various forms of life including fungi. We studied the species diversity and community phylogenetics of wood-inhabiting basidiomycetous fungi that revealed 175 wood-inhabiting basidiomycetous species, of which 20 represented unidentified species, based on morphological and phylogenetic analyses of 575 specimens collected from ten sampling sites. These species belonged to two classes, 11 orders, 42 families, and 106 genera of *Basidiomycota*, and included 12 edible species, 28 medicinal species, four poisonous species, and seven forest pathogens. Four types of fungal distribution pattern at the genus level were recognized for 65 genera, while another 41 genera could not be placed in any known distribution pattern. The five sampling sites in the eastern part of the Dabie Mountains had significantly higher species diversity and phylogenetic diversity of wood-inhabiting basidiomycetous fungi than those in the western part, and thus deserve priority in terms of conservation. The community of wood-inhabiting basidiomycetous fungi in the Dabie Mountains is generally affected by a combination of habitat filtering and competitive exclusion. This study provides a basis on which to build actions for the comprehensive recognition, utilization, and conservation of wood-inhabiting basidiomycetous fungi in the region.

**Supplementary Information:**

The online version contains supplementary material available at 10.1186/s43008-023-00130-9.

## INTRODUCTION

Wood-inhabiting fungi are macrofungi growing on various woody substrates and most belong to *Basidiomycota* (Wang et al. 2021a). Many species of wood-inhabiting fungi have edible and medicinal values, while others contain toxic metabolites (Wu et al. 2019; Zhou et al. 2022; Cheng et al. 2023). These properties make wood-inhabiting fungi economically valuable for development (Zhou 2020). As wood-inhabiting fungi efficiently degrade lignocellulose in wood (Floudas et al. 2012), they play a crucial ecological role in material recycling and energy flow in forest ecosystems (Zhou and Dai 2012; Dai et al. 2015). On the other hand, some wood-inhabiting fungi are forest pathogens, which can lead to huge economic losses (Wang et al. 2022). They are therefore an important strategic biological resources (Bai et al. 2023). To utilize these resources and protect them from harmful species, it is important to recognize and sample wood-inhabiting fungi as more as possible. The recognition of species diversity will also aid in the conservation of wood-inhabiting fungi worldwide (Krah et al. 2018; Yu et al. 2021a; Zhou and May 2023).

Systematic surveys of wood-inhabiting fungi are being carried out in most parts of the world, including Africa (Kinge et al. 2013), Asia (Doğan & Kurt 2016; Cho et al. 2019; Fedorenko 2019; Semwal and Bhatt 2019; Gafforov et al. 2020; Yusran et al. 2021; Aman et al. 2022), Europe (Dimou et al. 2016; Fink et al. 2021), and North America (Zhou et al. 2016). In China, besides the country-wide records of wood-inhabiting fungi (Dai 2011, 2012a), there are also assessments from some provinces (Dai et al. 2011; Bau et al. 2013; Lu et al. 2015; Ma et al. 2022) and famous reserves (Zhou et al. 2011; Zhou and Dai 2012; Dai et al. 2015; Yang et al. 2021; Wang et al. 2021c; Tuo et al. 2022). However, such data must be supplemented from other poorly known regions of China, especially for post-2020 global biodiversity conservation (Wei 2021).

The Dabie Mountains are located at the junction of Henan, Hubei, and Anhui Provinces, Central China, and are the geographic demarcation between North and South China. The climate of the Dabie Mountains provides favorable niches for the speciation and diversification of various forms of life (Cai et al. 2020). For example, 1108 species of vascular plants are reported in the Tiantangzhai Mountains of the Dabie Mountains (Shen 1986), 208 species of terrestrial vertebrates in the Hubei part of the Dabie Mountains (Fang et al. 2007), 283 species of birds in the Dabie Mountains (Sun et al. 2021), and 50 species of entomogenous fungi are known from the Anhui part of the Dabie Mountains (Wang et al. 2004). However, these studies are now mostly dated. Moreover, studies of wood-inhabiting fungi have rarely focused on the whole area of the Dabie Mountains (Yao et al. 2008; He et al. 2012; Yu et al. 2021b). It is to be expected that many wood-inhabiting fungi in the Dabie Mountains await to be recognized, utilized, and protected.

In a joint biodiversity survey of multiple forms of life in the Dabie Mountains initiated in 2020, wood-inhabiting basidiomycetous fungi were, for the first time, systematically investigated from all of the area. The aim of this study is to recognize the species diversity and community phylogenetics of wood-inhabiting basidiomycetous fungi from this area, and then to provide scientific understanding for the utilization and conservation of this fungal resource.

## MATERIALS AND METHODS

### Sampling area and strategy

**Fig. 1:**
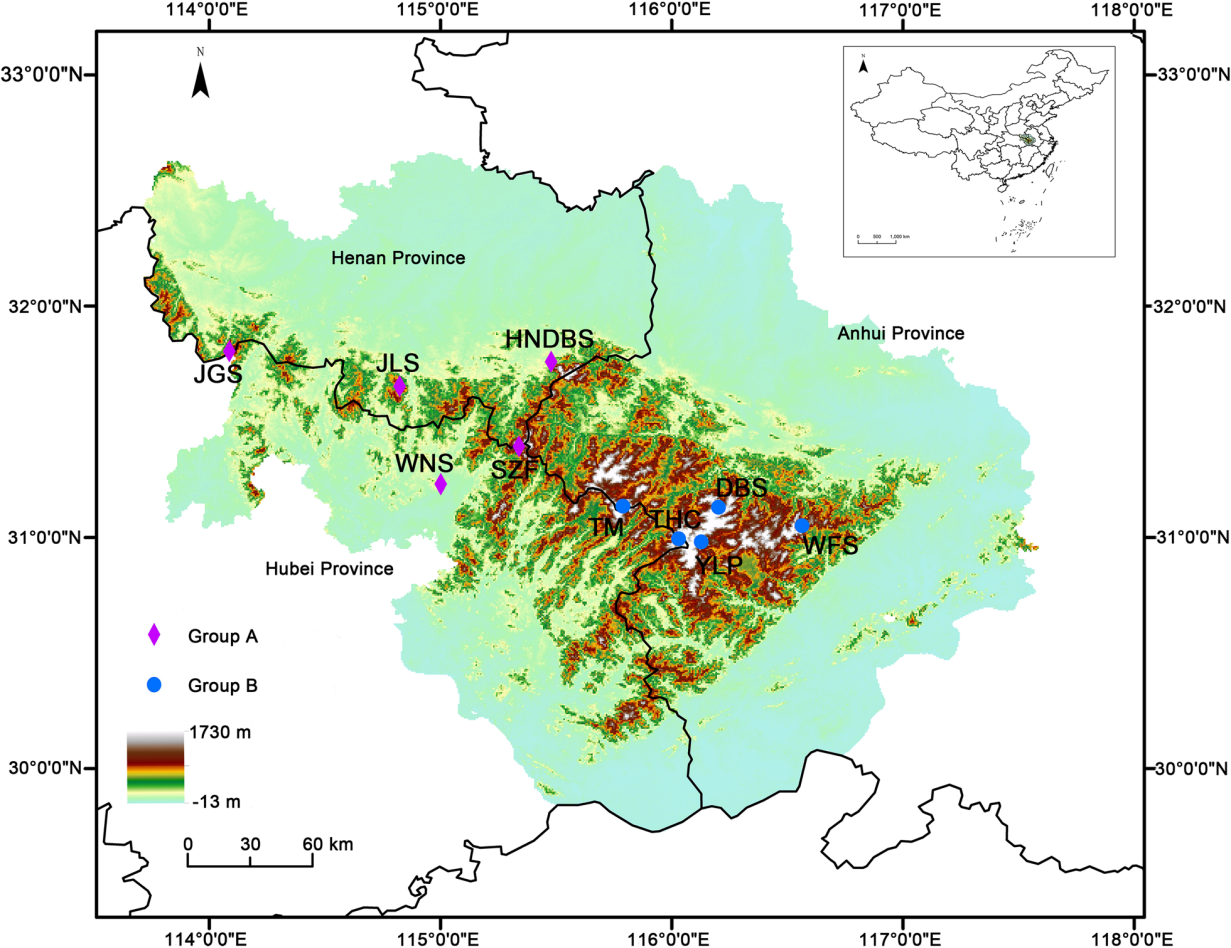
Locations of the ten sampling sites in the Dabie Mountains, Central China. The purple diamonds represent sampling sites in Group A, while the blue circles represent those in Group B. DBS is short for Dabieshan Main Peak Park, HNDBS for Henandabieshan National Nature Reserve, JGS for Jigongshan National Nature Reserve, JLS for Jinlanshan National Forest Park, SZF for Shizifeng Provincial Nature Reserve, THC for Taohuachong Scenic Area, TM for Tianma National Nature Reserve, WFS for Wanfoshan Provincial Nature Reserve, WNS for Wunaoshan National Forest Park, and YLP for Yaoluoping National Nature Reserve

The Dabie Mountains (30°10′–32°30′ N, 112°40′–117°10′ E) are located at the junction of three provinces, viz. Henan, Hubei, and Anhui, in the north subtropical zone of China. The Dabie Mountains mainly range from 500 to 800 m, while that of the highest is about 1500 m. The annual mean temperature is 12.5 °C, with January the coldest month (2 °C) and July the hottest (23 °C); annual mean precipitation is about 1833 mm, of which 45% occurs in summer (Cai et al. 2020). According to the vegetation types and accessibility, ten sampling sites were selected, viz. Dabieshan Main Peak Park (DBS), Henan-dabieshan National Nature Reserve (HNDBS), Jigongshan National Nature Reserve (JGS), Jinlanshan National Forest Park (JLS), Shizifeng Provincial Nature Reserve (SZF), Taohuachong Scenic Area (THC), Tianma National Nature Reserve (TM), Wanfoshan Provincial Nature Reserve (WFS), Wunaoshan National Forest Park (WNS), and Yaoluoping National Nature Reserve (YLP). Geologically, the Shangcheng-Macheng fault divides the Dabie orogenic belt into western and eastern parts (Luo et al. 2012), the eastern having a more humid climate than the western (Wu et al. 2022). Accordingly, the ten sampling sites were divided into two groups, viz. Group A comprising HNDBS, JGS, JLS, SZF, and WNS in the western part, and Group B DBS, THC, TM, WFS, and YLP in the eastern part. The geographic position of each sampling site was determined using portable GPS and mapped using ArcGIS 10.7 (Fig. 1).

Two field trips were made: the first in October 2020 and the second in September 2021. Each sampling site was surveyed with a similar intensity regarding the number of persons (four) and time (1–2 days) involved. Basidiomes of wood-inhabiting basidiomycetous fungi were collected and dried at 35 °C overnight using a portable oven. The dried basidiomes were frozen at − 80 °C for two weeks and then preserved at the Fungarium, Institute of Microbiology, Chinese Academy of Sciences (HMAS), Beijing, China.

### Species identification and annotation

Specimens were morphologically identified following Wang et al. (2021b) and Liu et al. (2022). Macromorphological characteristics were examined with a Leica M 125 stereomicroscope (Wetzlar, Germany) at magnifications up to 100×. For micromorphological characteristics, sections were separately prepared in Cotton Blue, Melzer’s reagent, and 5% potassium hydroxide; these were examined using an Olympus BX43 light microscope (Tokyo, Japan) at magnifications up to 1000×.

According to the morphological identifications, one representative specimen for each species was selected for molecular sequencing of the ITS and nLSU regions. A small piece was pulverized using a tissue grinder and DNA was extracted using a CTAB rapid plant genome extraction kit-DN14 (Aidlab Biotechnologies, Beijing, China) according to the manufacturer’s instructions. The crude DNA was used as a template for PCR amplification. The primer pairs ITS5/ITS4 (Gardes and Bruns 1993; White et al. 1990) and LR0R/LR7 (Vilgalys and Hester 1990) were selected to amplify the ITS and nLSU regions, respectively. The PCR procedures were as follows: for the ITS region, initial denaturation was performed at 95 °C for 3 min, 34 cycles at 94 °C for 40 s, 45 s at 57.2 °C, 1 min at 72 °C, and finally a 10 min extension at 72 °C; for the nLSU region, the initial denaturation was performed at 94 °C for 1 min, followed by 34 cycles at 94 °C for 30 s, 1 min at 47.2 °C, 1.5 min at 72 °C, and finally a 10 min extension at 72 °C. The PCR products were sequenced with the same primers as those used in PCR amplification at Beijing Tianyi Huiyuan Biotechnology (Beijing, China).

The newly generated sequences were submitted to GenBank (https://www.ncbi.nlm.nih.gov/genbank/; Additional file 1: Table S1). These sequences were used as queries for a BLAST search (https://blast.ncbi.nlm.nih.gov/Blast.cgi) to further confirm the morphological identification of specimens. The corresponding species names of the resulting hits with greater than 98% similarity and 90% coverage were considered reliable, besides the consistent morphological characteristics.

Following the practice of Lücking et al. (2020), phylogenetic analyses were finally performed for species identification. According to the preliminary species names resulting from morphological examinations and BLAST searches, 78 datasets with both newly generated sequences and related sequences downloaded from GenBank (Additional file 1: Table S1) were selected. For each dataset, the ITS and nLSU sequences were separately aligned using MAFFT 7.110 (Katoh and Standley 2013) and manually adjusted in MEGA 7 (Kumar et al. 2016). The resulting alignments were concatenated for phylogenetic analyses of the Maximum Likelihood (ML) algorithm. ML algorithm was conducted using raxmlGUI 2.0 (Edler et al. 2021; Stamatakis 2014) under the GTR + I + G model, and bootstrap (BS) replicates were determined under the auto FC option (Pattengale et al. 2010). Eventually, 78 phylogenetic trees were constructed for species identification (Additional file 3). The taxonomic status of accurately identified species is generally adjusted following Species Fungorum (https://www.speciesfungorum.org/).

The economic values (edible, medicinal, and poisonous) and pathogenicity of the identified wood-inhabiting basidiomycetous fungi were annotated based on Wu et al. (2019) and Dai (2012b), respectively. The types of fungal distribution pattern at the genus level were summarized with reference to previous publications (Song and Deng 2001; Song et al. 2001; Bian and Dai 2015; Zhang et al. 2015; Chen et al. 2019; Wang et al. 2021c).

### Community phylogenetics

All newly generated ITS and nLSU sequences were separately aligned using MAFFT 7.490 (Katoh and Standley 2013) and manually adjusted in MEGA 7 (Kumar et al. 2016). The resulting alignments of these two gene regions were concatenated to estimate the best-fit evolutionary model using jModelTest under the Akaike information criterion (Guindon and Gascuel 2003; Posada 2008). Following the estimated model, a phylogenetic analysis was performed for the concatenated alignment (Additional file 5) (Bouckaert et al. 2019) implemented in the CIPRES Science Gateway v3.3 (http://www.phylo.org/). The lognormal strict molecular clock model and the Yule speciation prior were used to estimate their corresponding credibility intervals. The root age was simply set to 1. The trees were sampled every 1000th generation from a total of 200 million generations. The top 10% of the sampled trees were discarded as burn-in and the resulting log file was checked to judge the chain convergence using Tracer 1.5. The maximum-clade-credibility (MCC) tree and Bayesian posterior probabilities were generated using TreeAnnotator v1.10.4 implemented in the CIPRES Science Gateway v3.3.

Based on the MCC tree as a template, the phylogenetic diversity, indicated by the phylogenetic distance amongst the wood-inhabiting basidiomycetous fungi in a single sampling site, was determined for each such site using program PD module of Phylocom 4.2 (Webb et al. 2008). Moreover, the community phylogenetic structure of each sampling site was evaluated using the net relatedness index (NRI) and nearest taxa index (NTI). The NRI is a standardized measure of the mean phylogenetic distance of all species pairs within a sampling site, while the NTI is a standardized measure of the mean of the nearest phylogenetic distance for each species within a sampling site (Webb et al. 2002). These two indices were calculated via randomly drawing species 999 times from the phylogenetic pool for the null model of random substitution using the construct module of Phylocom 4.2 (Webb et al. 2008). The positive value of NRI or NTI indicates that the species assemblage at a sampling site is closer (phylogenetic clustering) than the null model of random prediction and thus, the community consists of closely related species, while the negative value of NRI or NTI indicates that the species assemblage at a sampling site is more diverse (phylogenetic dispersion) than the null model of random prediction and the community consists of species with distant relatives (Webb et al. 2002).

### Statistical analysis

The differential significance of species richness and phylogenetic diversity between Group A and Group B was determined with an unpaired T-test implemented in GraphPad Prism v8.0.2.

## RESULTS

### Species diversity

**Fig. 2:**
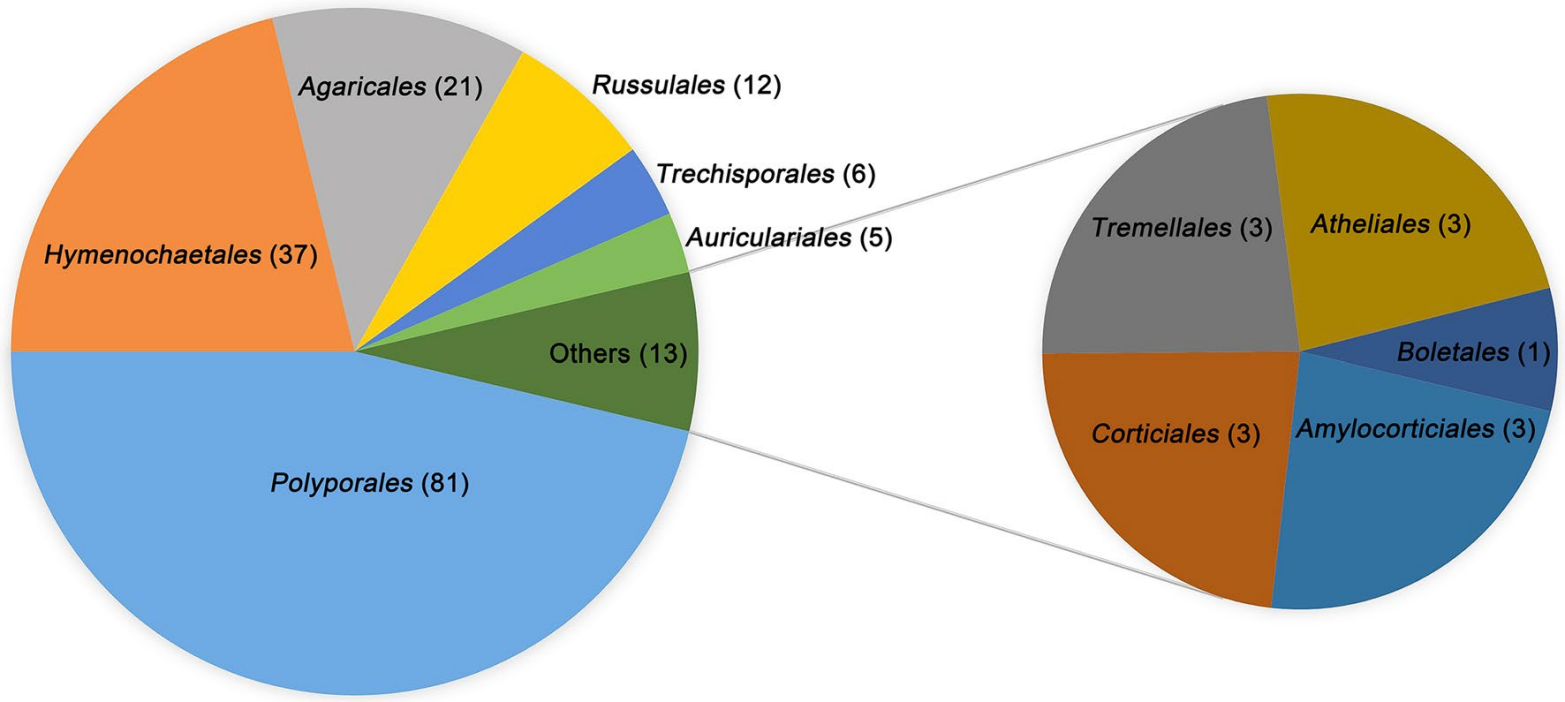
Taxonomic composition of 175 wood-inhabiting basidiomycetous species at the order level in the Dabie Mountains

From the 575 specimens collected from the two field trips in 2020 and 2021, 175 species of wood-inhabiting basidiomycetous fungi, including 20 unidentified species, were identified from the ten sampling sites (Additional file 2: Table S2). A total of 161 ITS and 157 nLSU sequences were newly generated from 175 specimens, each representing one of the species; these belonged to two classes, 11 orders, 42 families, and 106 genera within *Basidiomycota* (Table 1). Of the 11 orders, the most speciose orders were *Polyporales* (81 species), *Hymenochaetales* (37 species), *Agaricales* (21 species), and *Russulales* (12 species); another seven orders each had less than ten species, accounting for 13.7% of the total wood-inhabiting basidiomycetous species identified from the Dabie Mountains (Fig. 2).

**Table 1:** The taxonomic position of 175 wood-inhabiting basidiomycetous species in the dabie mountains

Class	Order	Family	Number of genera	Number of species
*Agaricomycetes*	*Agaricales*	*Crepidotaceae*	1	1
		*Cyphellaceae*	2	2
		*Hymenogastraceae*	1	1
		*Mycenaceae*	2	3
		*Phyllotopsidaceae*	1	1
		*Physalacriaceae*	2	3
		*Pleurotaceae*	1	2
		*Radulomycetaceae*	1	2
		*Schizophyllaceae*	1	1
		*Strophariaceae*	3	5
	*Amylocorticiales*	*Amylocorticiaceae*	3	3
	*Atheliales*	*Atheliaceae*	3	3
	*Auriculariales*	*Auriculariaceae*	4	5
	*Boletales*	*Coniophoraceae*	1	1
	*Corticiales*	*Corticiaceae*	1	1
		*Punctulariaceae*	1	1
		*Vuilleminiaceae*	1	1
	*Hymenochaetales*	*Hymenochaetaceae*	5	11
		*Hyphodontiaceae*	1	1
		*Oxyporaceae*	1	3
		*Schizoporaceae*	4	12
		*Xenasmataceae*	1	1
		Incertae sedis	5	9
	*Polyporales*	*Cerrenaceae*	1	2
		*Dacryobolaceae*	2	3
		*Fibroporiaceae*	1	1
		*Fomitopsidaceae*	4	7
		*Gelatoporiaceae*	1	1
		*Grifolaceae*	1	1
		*Hyphodermataceae*	1	6
		*Incrustoporiaceae*	1	3
		*Irpicaceae*	4	6
		*Meruliaceae*	3	6
		*Phanerochaetaceae*	6	12
		*Polyporaceae*	13	20
		*Steccherinaceae*	3	7
		Incertae sedis	5	6
	*Russulales*	*Auriscalpiaceae*	2	2
		*Bondarzewiaceae*	1	1
		*Hericiaceae*	1	1
		*Peniophoraceae*	3	4
		*Stereaceae*	3	4
	*Trechisporales*	*Hydnodontaceae*	1	6
*Tremellomycetes*	*Tremellales*	*Tremellaceae*	2	2
		Incertae sedis	1	1

**Fig. 3:**
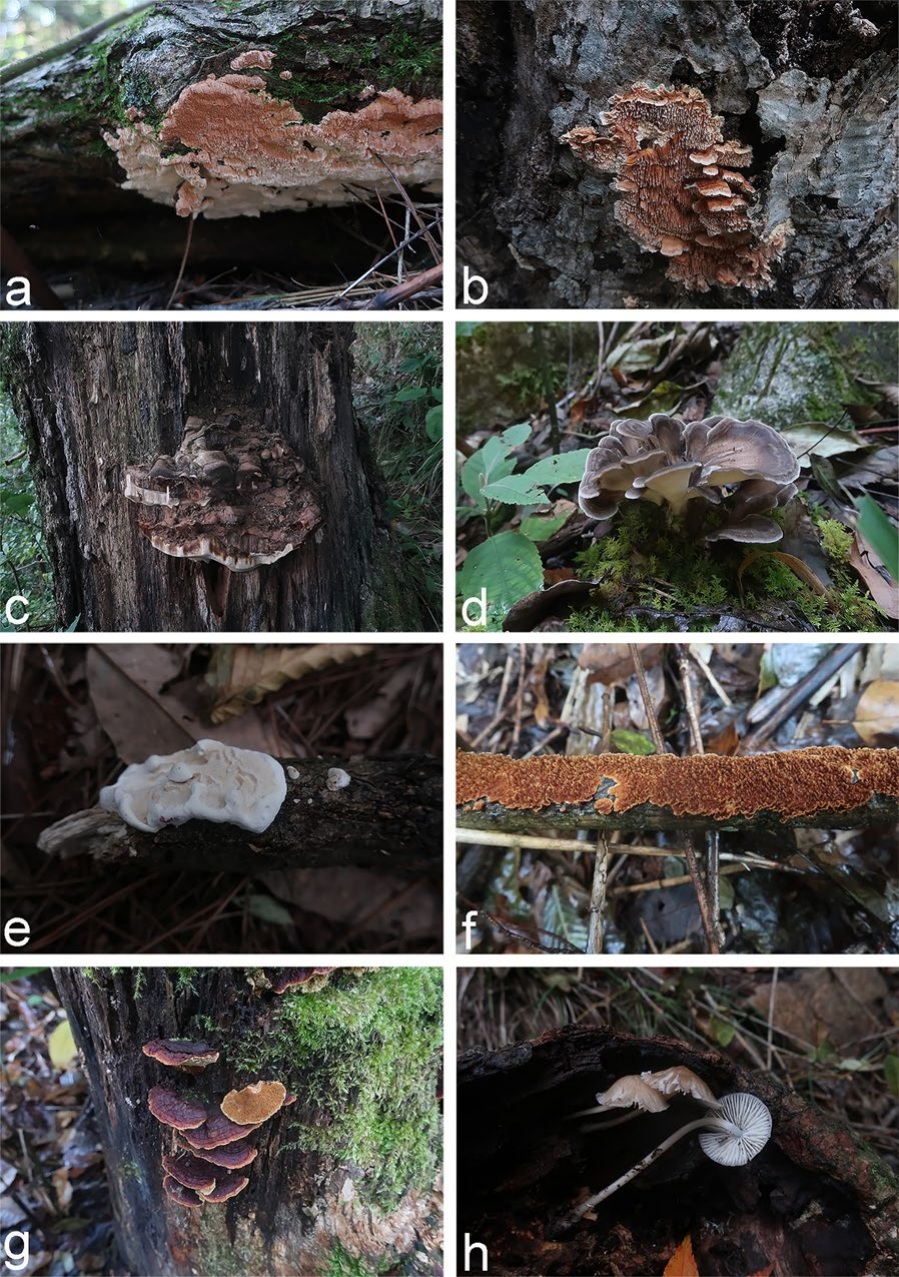
Fruiting bodies in situ of (**a**) *Cerrena albocinnamomea* (LWZ 20210918-12a, HMAS 256498) (**b**) *Cerrena zonata* (LWZ 20201012-40, HMAS 256315) (**c**) *Ganoderma gibbosum* (LWZ 20201013-11, HMAS 256327) (**d**) *Grifola frondosa* (LWZ 20201011-10, HMAS 256250) (**e**) *Heterobasidion araucariae* (LWZ 20210919-26a, HMAS 256572) (**f**) *Hymenochaete huangshanensis* (LWZ 20201015-10, HMAS 256380) (**g**) *Hymenochaete xerantica* (LWZ 20201011-51, HMAS 256287) (**h**) *Mycena galericulata* (LWZ 20201017-57, HMAS 256465) in the Dabie Mountains

**Fig. 4:**
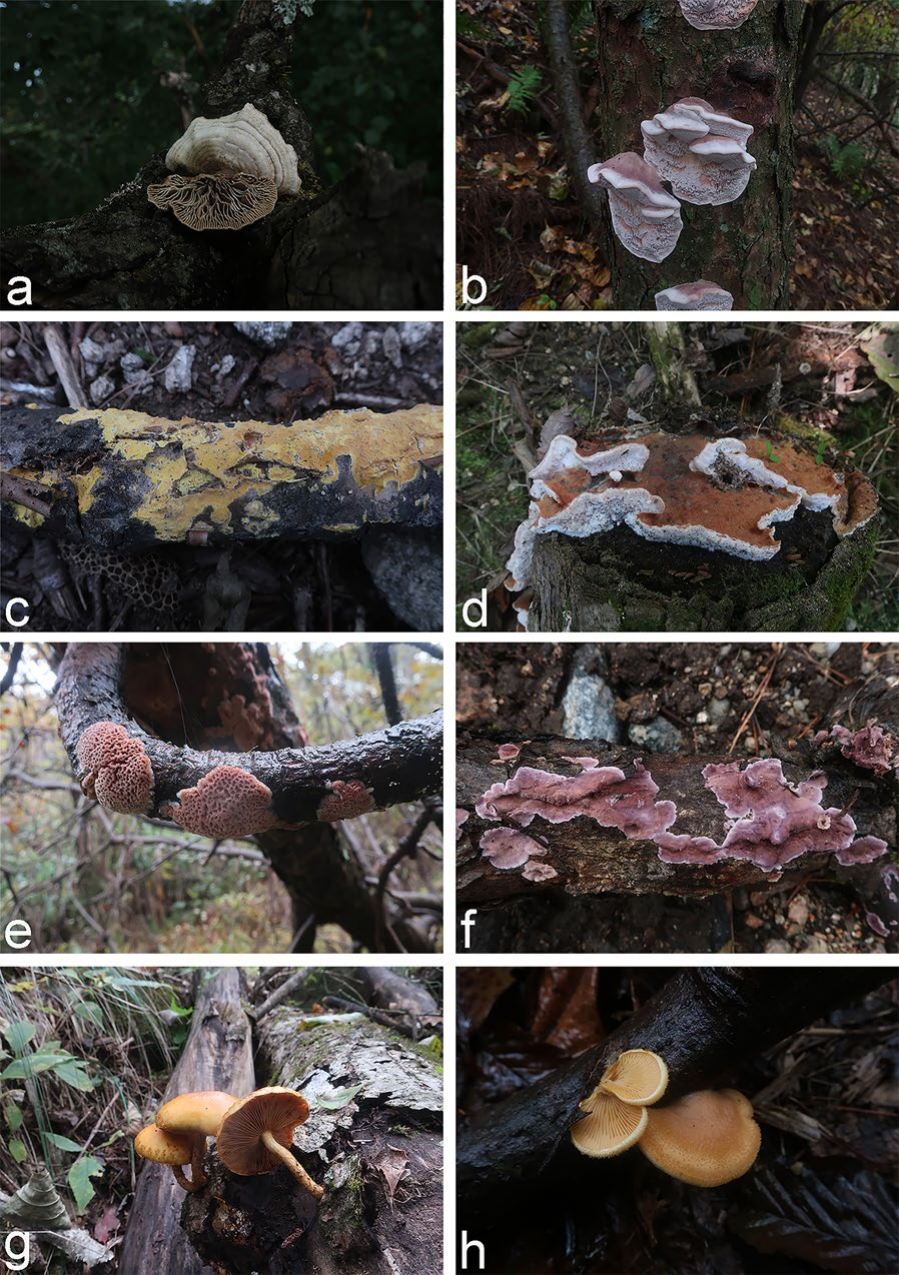
Fruiting bodies in situ of (**a**) *Lenzites betulinus* (LWZ 20210919-50a, HMAS 256605) (**b**) *Leptoporus mollis* (LWZ 20201014-19, HMAS 256350) (**c**) *Mycoacia lutea* (LWZ 20210919-30a, HMAS 256578) (**d**) *Phlebia tremellosa* (LWZ 20201013-7, HMAS 256323) (**e**) *Phlebiopsis castanea* (LWZ 20201015–4, HMAS 256374) (**f**) *Phlebiopsis crassa* (LWZ 20201017-9, HMAS 256426) (**g**) *Pholiota limonella* (LWZ 20201012-24, HMAS 256305) (**h**) *Phyllotopsis nidulans* (LWZ 20201015-24, HMAS 256393) in the Dabie Mountains

**Fig. 5:**
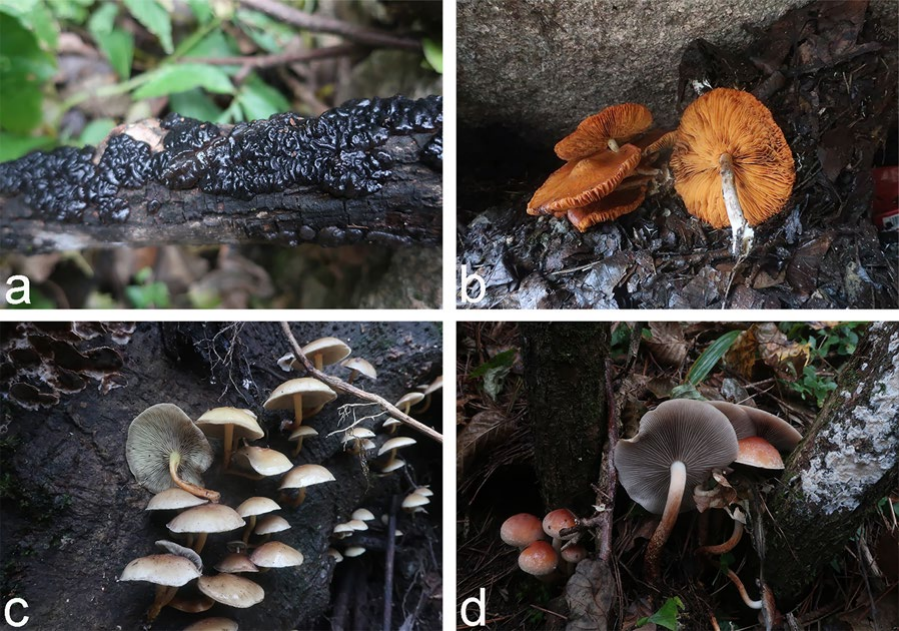
Fruiting bodies of four poisonous wood-inhabiting basidiomycetous fungi in situ in the Dabie Mountains. **a** *Exidia glandulosa* (LWZ 20201017-65, HMAS 256473). **b** *Gymnopilus penetrans* (LWZ 20201017-25, HMAS 256441). **c** *Hypholoma fasciculare* (LWZ 20201017-59, HMAS 256467). **d** *Hypholoma lateritium* (LWZ 20201014-28, HMAS 256358)

An annotated checklist of the 175 species of wood-inhabiting basidiomycetous fungi identified from the Dabie Mountains is given in Additional file 4. The voucher specimens and corresponding sampling information for the 175 species are included. Photos of basidiomes in situ are presented for selected species (Figs. 3, 4 and 5).

### Generic diversity

**Fig. 6:**
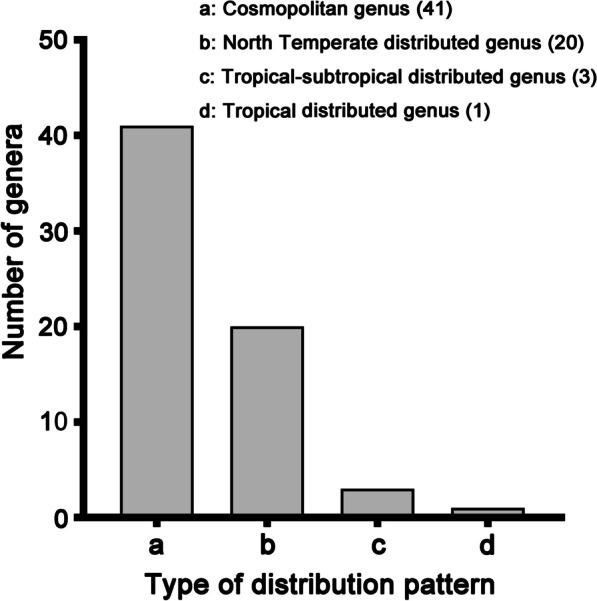
Type of fungal distribution pattern at the genus level in the Dabie Mountains. **a** Cosmopolitan genus. **b** North Temperate distributed genus. **c** Tropical-subtropical distributed genus. **d** Tropical distributed genus

Four types of distribution pattern of wood-inhabiting basidiomycetous fungi at the genus level were found in the Dabie Mountains (Fig. 6).

The dominant type were 41 genera regarded as cosmopolitan: *Antrodia*, *Antrodiella*, *Armillaria*, *Auricularia*, *Bjerkandera*, *Cerrena*, *Corticium*, *Crepidotus*, *Cyanosporus*, *Daedalea*, *Daedaleopsis*, *Exidia*, *Fomitiporia*, *Fomitopsis*, *Fuscoporia*, *Gloeocystidiellum*, *Gymnopilus*, *Heterobasidion*, *Hyphodontia*, *Hypholoma*, *Irpex*, *Junghuhnia*, *Lenzites*, *Lopharia*, *Mycena*, *Panellus*, *Peniophora*, *Perenniporia*, *Phyllotopsis*, *Pleurotus*, *Radulomyces*, *Rigidoporus*, *Schizophyllum*, *Skeletocutis*, *Stereum*, *Stropharia*, *Trametes*, *Trechispora*, *Tremella*, *Trichaptum*, and *Truncospora*.

North Temperate distributed genera numbered found were 20: *Abundisporus*, *Amaropostia*, *Anomoloma*, *Artomyces*, *Auriscalpium*, *Cinereomyces*, *Cystidiopostia*, *Fibroporia*, *Fuscopostia*, *Ganoderma*, *Grifola*, *Hydnophlebia*, *Hymenochaete*, *Lentinus*, *Leptoporus*, *Pholiota*, *Poriodontia*, *Postia*, *Rhodofomes*, and *Steccherinum*.

Three genera were tropical-subtropical elements: *Megasporoporiella*, *Sirobasidium*, and *Vitreoporus*, while and *Xylodon* is considered to be the tropical distributed genus.

In addition to the above-mentioned 65 genera, 41 genera cannot be referred to these broad geographical categories from current knowledge.

### Community phylogenetic diversity and structure

**Fig. 7:**
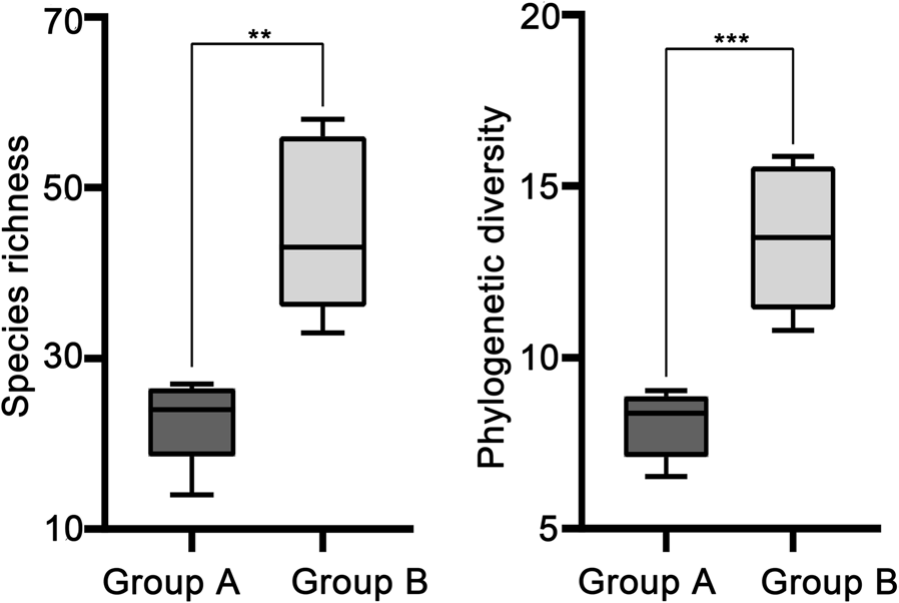
Species richness and phylogenetic diversity of wood-inhabiting basidiomycetous fungi in Group A and Group B. ** represents *p* value less than 0.01, while *** represents *p* value less than 0.001

DBS is the most speciose of the ten sampling sites with 58 species of wood-inhabiting basidiomycetous fungi, followed by the four sites in Group B, viz. TM, WFS, YLP and THC (Table 2). Compared with the sampling sites in Group B, the number of species at each site of Group A is lower, with JGS being the least speciose (14 species; Table 2). The species richness in Group B is significantly higher than in Group A (Fig. 7).

**Table 2:** Species richness, phylogenetic diversity, net relatedness index and nearest taxon index of ten sampling sites in the Dabie Mountains

Group	Sampling site	Species richness	Phylogenetic diversity	Net relatedness index	Nearest taxon index
A	HNDBS	23	7.674	1.2763	0.8673
	JGS	14	6.527	− 0.7837	− 1.4981
	JLS	26	8.382	0.8335	0.4380
	SZF	24	8.711	0.1047	− 0.3279
	WNS	27	9.035	0.3396	0.7410
B	DBS	58	15.869	0.1340	1.5445
	THC	33	10.797	− 0.8840	0.4516
	TM	54	15.272	0.5114	0.5499
	WFS	43	13.499	− 0.9601	1.0645
	YLP	39	11.979	0.3986	0.7779

An alignment of 2397 characters was generated from the combined dataset of ITS and nLSU regions for community phylogenetic analysis (Additional file 5). The best-fit evolutionary model of this alignment was estimated as GTR + I + G. The phylogenetic relationships among the 175 species in the resulting MCC tree (Additional file 6) were used as a template for subsequent analyses. Similar to the trend of species richness, DBS has the highest phylogenetic diversity and the JGS has the lowest among the ten sampling sites (Table 2), while that in Group B is significantly higher than in Group A (Fig. 7).

For the two indices of phylogenetic structure, both NRI and NTI are positive in the sampling sites DBS, HNDBS, JLS, TM, WNS, and YLP, which indicates that their communities are prone to phylogenetic clustering (Table 2). In contrast, site JGS has negative values of both NRI and NTI, and thus the communities in these two sampling sites show a trend of phylogenetic dispersion (Table 2). The trend in phylogenetic structure in the communities of another four sampling sites, viz. SZF, THC, and WFS, could not be consistently summarized from the NRI and NTI.

## DISCUSSION

From ten sampling sites in the Dabie Mountains, 175 wood-inhabiting basidiomycetous fungi from 106 genera were recognized, based on both morphological and molecular evidence (with vouchers preserved in HMAS) following good practice in fungal taxonomy (Aime et al. 2021). In contrast, previous investigations had generally been restricted to a single reserve in the Dabie Mountains and provided species names without any deposition of voucher specimens or molecular sequencing (Yao et al. 2008; He et al. 2012), so identifications cannot be verified. Although the survey intensity in terms of people/hours was not massive, and some species will surely have been missed, this study provides the first standardized procedure for the recognition of wood-inhabiting basidiomycetous fungi in the region. In the future, more field trips adopting this standard procedure will be needed to comprehensively understand the species diversity of these fungi.

Besides the traditional basidiome-based method of surveying wood-inhabiting basidiomycetous fungi, the metabarcoding method with the rapid development of high throughput sequencing technology is becoming increasingly used for exploring species diversity (Runnel et al. 2015; Tedersoo et al. 2022; Zhou 2023). However, a recent comparison indicated that both methods could give similar accounts of species diversity, but that the basidiome-based method was superior when conservation assessment was the main interest (Frøslev et al. 2019). Ideally, both methods should be simultaneously performed to increase our knowledge of the species diversity of wood-inhabiting basidiomycetous fungi (Truong et al. 2017) and also to test the conclusions of Frøslev et al. (2019) in a different region.

It is well known that some wood-inhabiting basidiomycetous fungi are edible and have medicinal properties (Wu et al. 2019; Zhou et al 2022; Cheng et al. 2023). They can be of nutritional value and a benefit to human health (Zhang et al. 2021). More importantly, edible and medicinal fungi are the fifth largest crop industry in China (Dong et al. 2017). Therefore, the resources of wood-inhabiting basidiomycetous fungi in the Dabie Mountains can contribute to economic development in the surrounding areas. Of the recognized edible and medicinal fungi in the Dabie Mountains, eight species, viz. *Armillaria gallica*, *A. mellea*, *Auricularia cornea*, *Bjerkandera fumosa*, *Grifola frondosa*, *Phaeotremella foliacea*, *Pleurotus pulmonarius*, and *Schizophyllum commune* are edible as well as having medicinal properties. These species could be treated as the first candidates for exploitation. Further, *Armillaria mellea*, *Auricularia cornea*, *Grifola frondosa*, and *Schizophyllum commune* have been cultivated outside the Dabie Mountains (Kim et al. 1992; Mayuzumi and Mizuno 1997; Dasanayaka and Wijeyaratne 2017; Bandara et al. 2020). However, because even the same fungal species, when coming from various regions, may possess differential biological properties (Taylor et al. 2006), fresh sources are of value.

*Exidia glandulosa*, *Gymnopilus penetrans*, *Hypholoma fasciculare*, and *H. lateritium* are four known poisonous species of wood-inhabiting basidiomycetous fungi so far found in the Dabie Mountains (Fig. 5); these can cause gastroenteritis (Chen et al. 2016) and so should be avoided. However, as in other areas (Wu et al. 2019), current knowledge of poisonous wood-inhabiting basidiomycetous fungi in the Dabie Mountains remains insufficient. Furthermore, there is no guarantee that all other wood-inhabiting basidiomycetous fungi in the Dabie Mountains are safe to be eaten.

Seven of the wood-inhabiting basidiomycetous fungi we recorded are forest pathogens (Dai 2012b). These merit more attention to prevent ecological and economic losses arising from their pathogenicity. For example, *Chondrostereum purpureum*, causal agent of silver leaf disease on apple trees leads to a reduction in yield (Setliff 2002). Another 168 species of the wood-inhabiting basidiomycetous fungi we recorded are wood decomposers. Their ability to degrade complex compounds makes them a potential resource for biotechnology. For example, *Trametes hirsuta* is capable of hydrolyzing biomass into fermentable sugars and converting them directly into ethanol, thus offering promising applications in bioprocessing (Okamoto et al. 2011). More importantly, these wood decomposers are irreplaceable in the turnover of woody plants (Zhou and Dai 2012; Dai et al. 2015). Therefore, in addition to economically important species, the conservation of all wood-inhabiting basidiomycetous fungi is important to preserve forest ecosystems.

Community phylogenetics is the integration of species phylogenetic relationships into the study of community ecology and includes both phylogenetic diversity and phylogenetic structure assemblages (Webb et al. 2002). Phylogenetic diversity can be used as a complementary measure for nature conservation (Faith 1992; Faith et al. 2004). Community phylogenetic structure is used to analyze the status and causes of community species composition from an evolutionary perspective (Cavender-Bares et al. 2009). Compared with species richness that cannot reflect the changes in species numbers among communities (Warwick and Clarke 1995), community phylogenetics is critical in identifying evolutionary distinct lineages that are considered to be priorities for conservation (Forest et al. 2007; Thuiller et al. 2011; Owen et al. 2019). Therefore, community phylogenetics has attracted increasing attention in the field of conservation biology (Crisp and Cook 2012), especially in the ecology and conservation of plants (Li et al. 2015; Zhang and Zhang 2017) and animals (Graham et al. 2009; Kuntner et al. 2011; Tims and Alroy 2021). In fungi, community phylogenetics has so far been mainly applied to arbuscular mycorrhizal fungi (Egan et al. 2017; Horn et al. 2017; Chai et al. 2018) and lichenized fungi (Nascimento et al. 2021), and only rarely in wood-inhabiting fungi (Abrego et al. 2022). At the scale of sampling sites and groups, the species richness and phylogenetic diversity have similar changing trends, indicating that community phylogenetics is suitable for analyzing wood-inhabiting basidiomycetous fungi in a site such as the Dabie Mountains. In addition, 64.5% of the species of edible and medicinal wood-inhabiting basidiomycetous fungi found in this survey were exclusive to sampling sites in Group B. Given above, the eastern part of the Dabie Mountains represented by the sampling sites in Group B therefore deserves priority in terms of the conservation of these fungi.

Normally, NRI and NTI are considered to be meaningful when their absolute values are higher than 1.96 (Vamosi et al. 2009). In our current study, all of the absolute values were lower than 1.96 (Table 2), suggesting that the community of wood-inhabiting basidiomycetous fungi in the Dabie Mountains is affected by a combination of habitat filtering and competitive exclusion. Therefore, to preserve the diversity of wood-inhabiting basidiomycetous fungi, conserving their habitat in the forest ecosystems is crucial.

## CONCLUSION

In summary, the current study, for the first time, provides an annotated species checklist with voucher specimens preserved in a fungarium for wood-inhabiting basidiomycetous fungi for the entire Dabie Mountains. Moreover, a combination of habitat filtering and competitive exclusion determines the community of wood-inhabiting basidiomycetous fungi in the Dabie Mountains, and the five sampling sites in the eastern part of the Dabie Mountains deserve priority in terms of conservation.

### Supplementary Information

**Additional file 1**. **Table S1**. Information of sequences used in the phylogenetic analyses.

**Additional file 2**. **Table S2**: Species distribution in each sampling site in the Dabie Mountains.

**Additional file 3**. A total of 78 phylogenetic trees for species identification. The phylogenetic trees were inferred from ITS and nLSU regions by the maximum likelihood algorithm. The bootstrap values above 50% are labeled at the nodes.

**Additional file 4**. An annotated checklist of wood-inhabiting basidiomycetous fungi in the Dabie Mountain.

**Additional file 5**. The concatenated alignment of ITS and nLSU regions for community phylogenetics.

**Additional file 6**. The phylogenetic relationships among the 175 wood-inhabiting basidiomycetous species in the Dabie Mountains. The maximum-clade-credibility tree was inferred from ITS and nLSU regions. The Bayesian posterior probabilities above 0.8 are labeled at the nodes.

## Data Availability

All sequence data generated for this study can be accessed via GenBank (https://www.ncbi.nlm.nih.gov/genbank/).

## Abbreviations

CTAB: Cetyl-trimethyl-ammonium bromide
ITS: Nuclear ribosomal internal transcribed spacer
nLSU: Large subunit nuclear ribosomal RNA gene
PCR: Polymerase chain reaction
BEAST: Bayesian evolutionary analysis sampling trees
