# Supplementary material for: Preliminary species diversity and community phylogenetics of wood-inhabiting basidiomycetous fungi in the Dabie Mountains, Central China reveal unexpected richness
Source: IMA Fungus. 2023 Nov 14;14:23. doi: 10.1186/s43008-023-00130-9 (PMC10644440; doi:10.1186/s43008-023-00130-9)
Supplement: Supplementary file 3 — Additional file 3. A total of 78 phylogenetic trees for species identification. The phylogenetic trees were inferred from ITS and nLSU regions by the maximum likelihood algorithm. The bootstrap values above 50% are labeled at the nodes. [file 43008_2023_130_MOESM3_ESM.pdf]

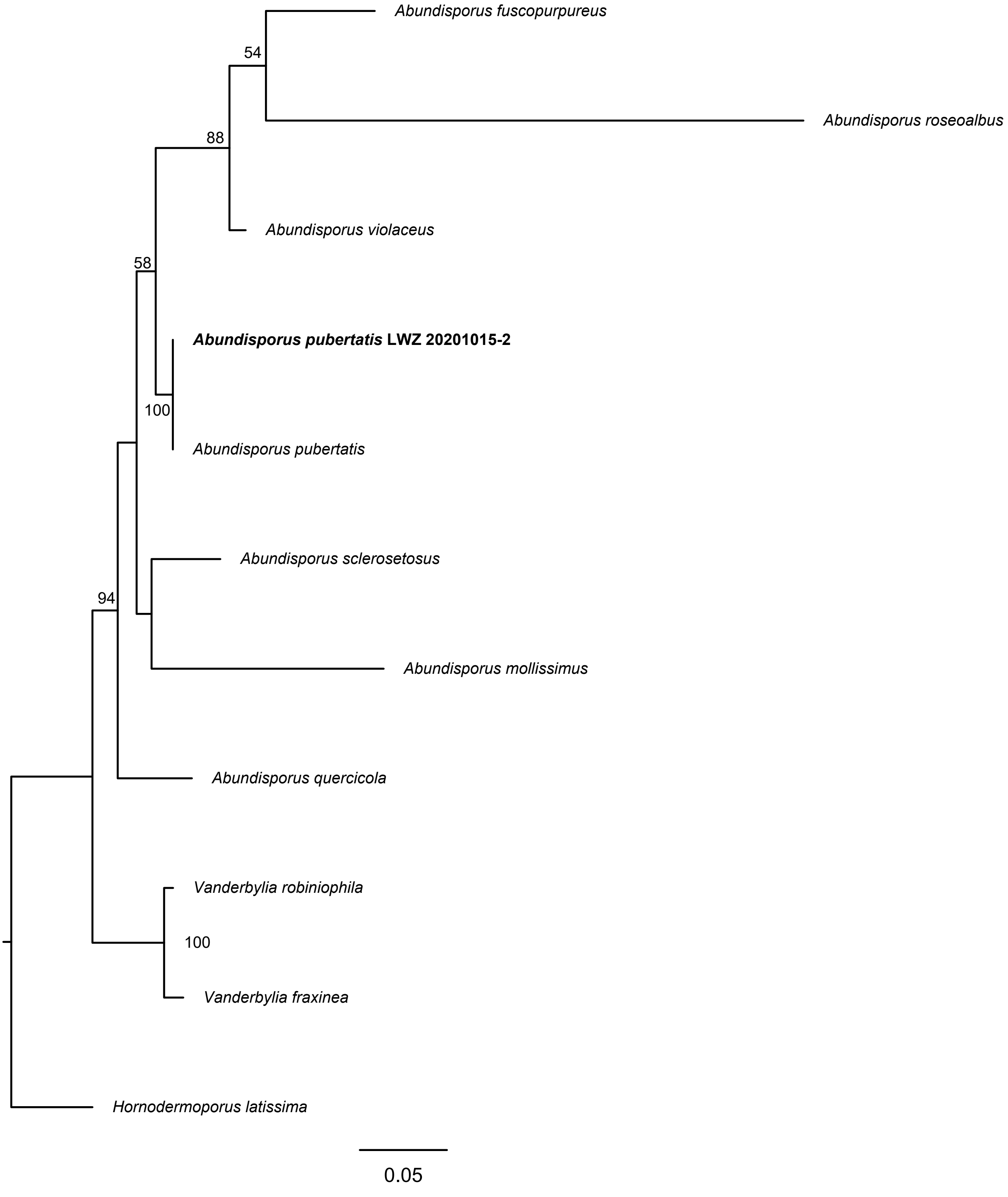

Abundisporus fuscopurpureus
54
Abundisporus roseoalbus
88
Abundisporus violaceus
58
Abundisporus pubertatis LWZ 20201015-2
100
Abundisporus pubertatis
Abundisporus sclerosetosus
94
Abundisporus mollissimus
Abundisporus quercicola
Vanderbylia robiniophila
100
Vanderbylia fraxinea
Hornodermoporus latissima
0.05

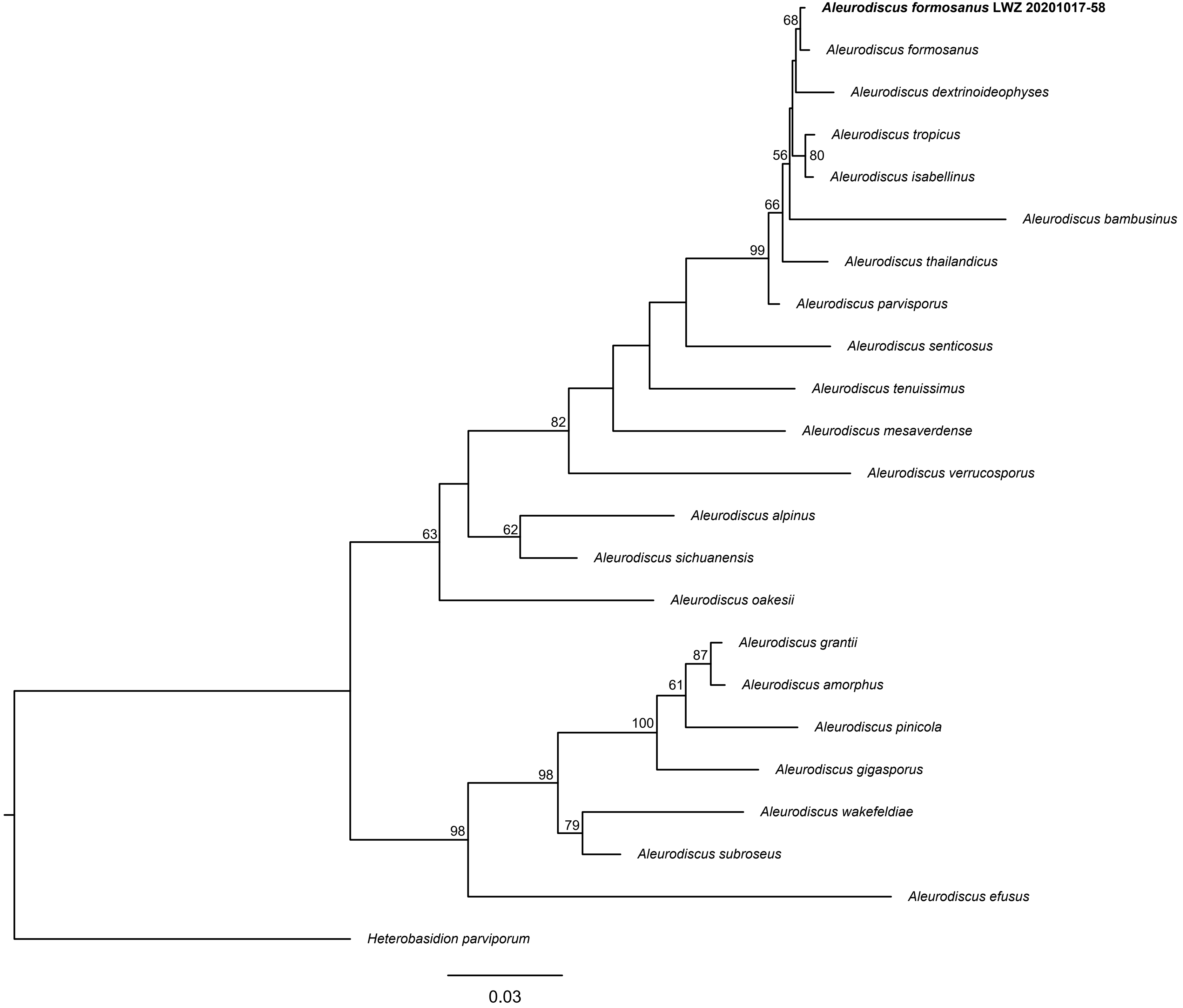
Aleurodiscus formosanus LWZ 20201017-58
Aleurodiscus formosanus
Aleurodiscus dextrinoideophyses
Aleurodiscus tropicus
Aleurodiscus isabellinus
Aleurodiscus bambusinus
Aleurodiscus thailandicus
Aleurodiscus parvisporus
Aleurodiscus senticosus
Aleurodiscus tenuissimus
Aleurodiscus mesaverdense
Aleurodiscus verrucosporus
Aleurodiscus alpinus
Aleurodiscus sichuanensis
Aleurodiscus oakesii
Aleurodiscus grantii
Aleurodiscus amorphus
Aleurodiscus pinicola
Aleurodiscus gigasporus
Aleurodiscus wakefeldiae
Aleurodiscus subroseus
Aleurodiscus efusus
Heterobasidion parviporum
68
80
56
66
99
82
63
62
87
61
100
98
79
98
0.03

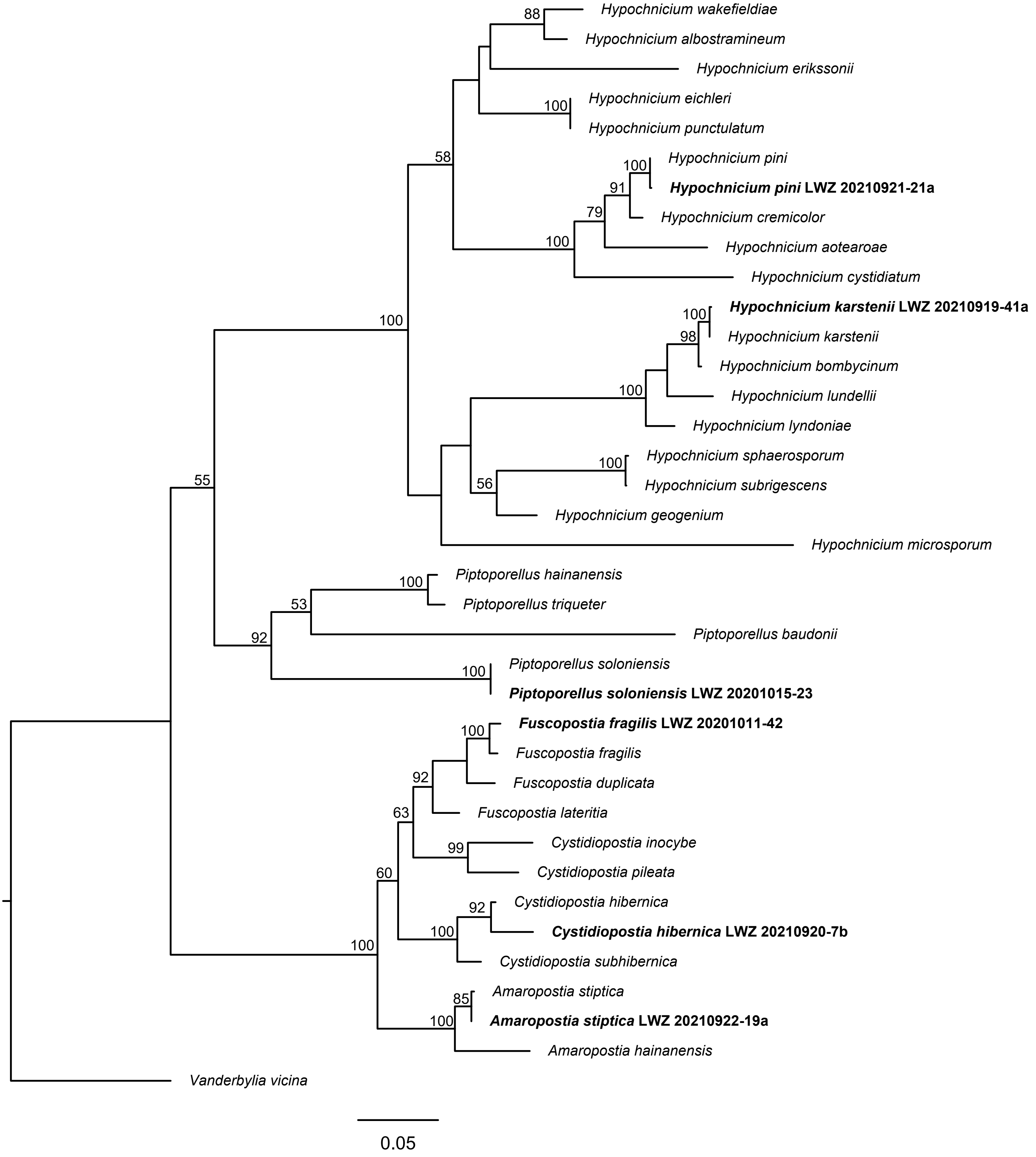

Hypochnicium wakefieldiae
Hypochnicium albostramineum
Hypochnicium erikssonii
Hypochnicium eichleri
Hypochnicium punctulatum
Hypochnicium pini
Hypochnicium pini LWZ 20210921-21a
Hypochnicium cremicolor
Hypochnicium aotearoae
Hypochnicium cystidiatum
Hypochnicium karstenii LWZ 20210919-41a
Hypochnicium karstenii
Hypochnicium bombycinum
Hypochnicium lundellii
Hypochnicium lyndoniae
Hypochnicium sphaerosporum
Hypochnicium subrigescens
Hypochnicium geogenium
Hypochnicium microsporum
Piptoporellus hainanensis
Piptoporellus triqueter
Piptoporellus baudonii
Piptoporellus soloniensis
Piptoporellus soloniensis LWZ 20201015-23
Fuscopostia fragilis LWZ 20201011-42
Fuscopostia fragilis
Fuscopostia duplicata
Fuscopostia lateritia
Cystidiopostia inocybe
Cystidiopostia pileata
Cystidiopostia hibernica
Cystidiopostia hibernica LWZ 20210920-7b
Cystidiopostia subhibernica
Amaropostia stiptica
Amaropostia stiptica LWZ 20210922-19a
Amaropostia hainanensis
Vanderbylia vicina
88
100
58
100
91
79
100
100
100
98
100
100
56
55
100
53
92
100
100
92
63
99
60
92
100
100
85
100
0.05

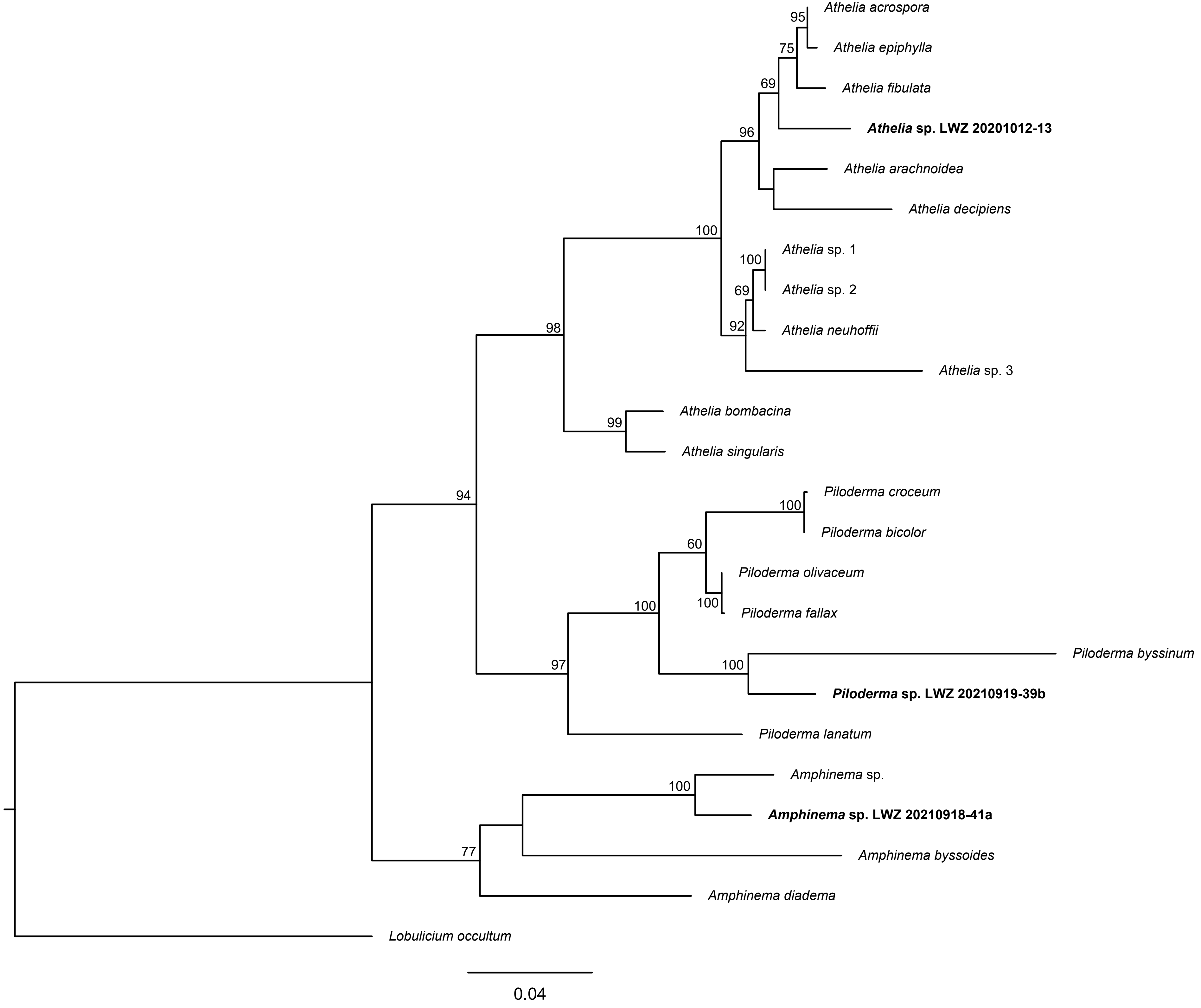
Athelia acrospora
Athelia epiphylla
Athelia fibulata
Athelia sp. LWZ 20201012-13
Athelia arachnoidea
Athelia decipiens
Athelia sp. 1
Athelia sp. 2
Athelia neuhoffii
Athelia sp. 3
Athelia bombacina
Athelia singularis
Piloderma croceum
Piloderma bicolor
Piloderma olivaceum
Piloderma fallax
Piloderma byssinum
Piloderma sp. LWZ 20210919-39b
Piloderma lanatum
Amphinema sp.
Amphinema sp. LWZ 20210918-41a
Amphinema byssoides
Amphinema diadema
Lobulicium occultum
95
75
69
96
100
100
69
92
98
99
94
100
60
100
100
100
97
100
77
0.04

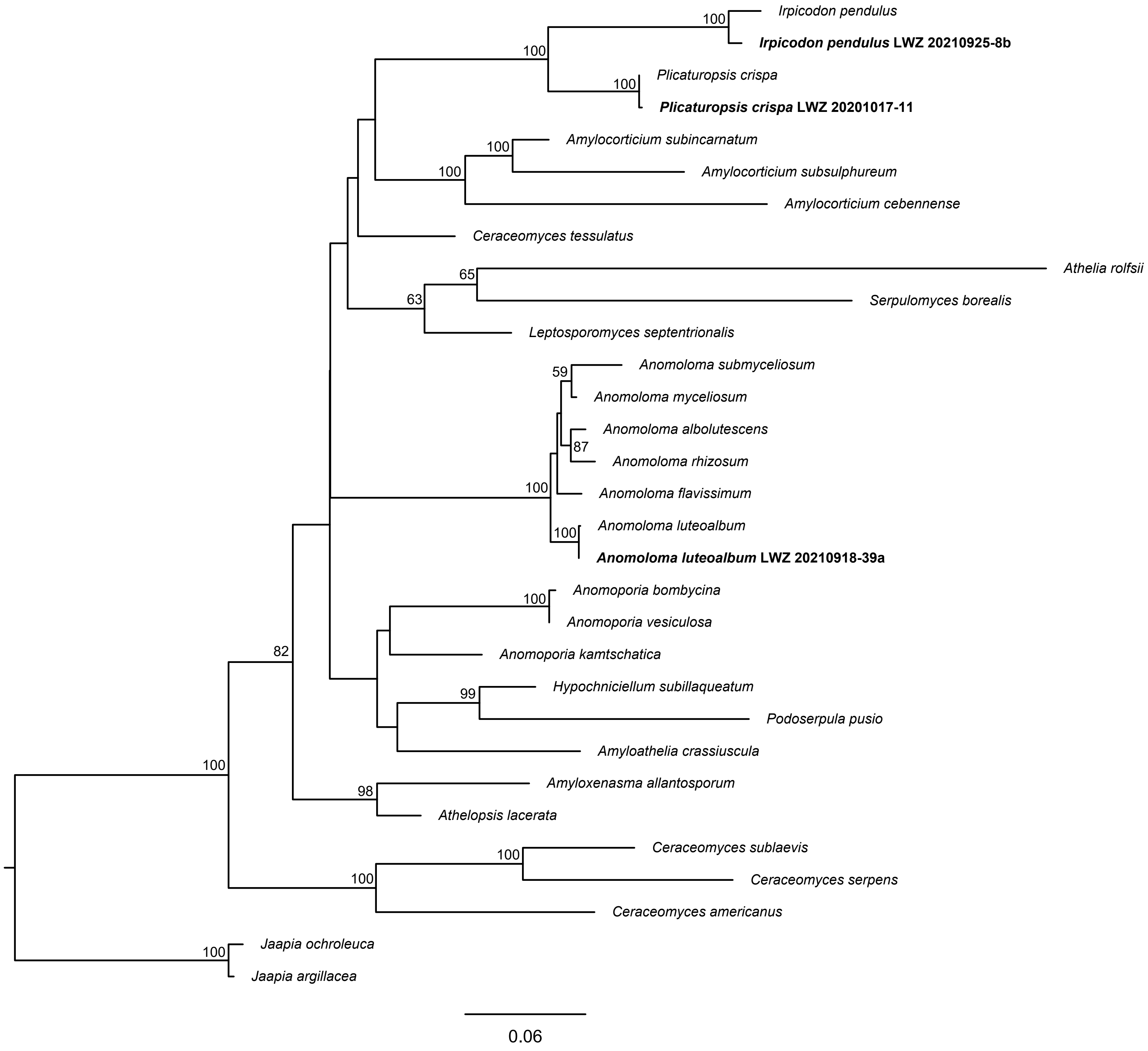

Irpicodon pendulus
Irpicodon pendulus LWZ 20210925-8b
Plicaturopsis crispa
Plicaturopsis crispa LWZ 20201017-11
Amylocorticium subincarnatum
Amylocorticium subsulphureum
Amylocorticium cebennense
Ceraceomyces tessulatus
Athelia rolfsii
Serpulomyces borealis
Leptosporomyces septentrionalis
Anomoloma submyceliosum
Anomoloma myceliosum
Anomoloma albolutescens
Anomoloma rhizosum
Anomoloma flavissimum
Anomoloma luteoalbum
Anomoloma luteoalbum LWZ 20210918-39a
Anomoporia bombycina
Anomoporia vesiculosa
Anomoporia kamtschatica
Hypochniciellum subillaqueatum
Podoserpula pusio
Amyloathelia crassiuscula
Amyloxenasma allantosporum
Athelopsis lacerata
Ceraceomyces sublaevis
Ceraceomyces serpens
Ceraceomyces americanus
Jaapia ochroleuca
Jaapia argillacea
0.06

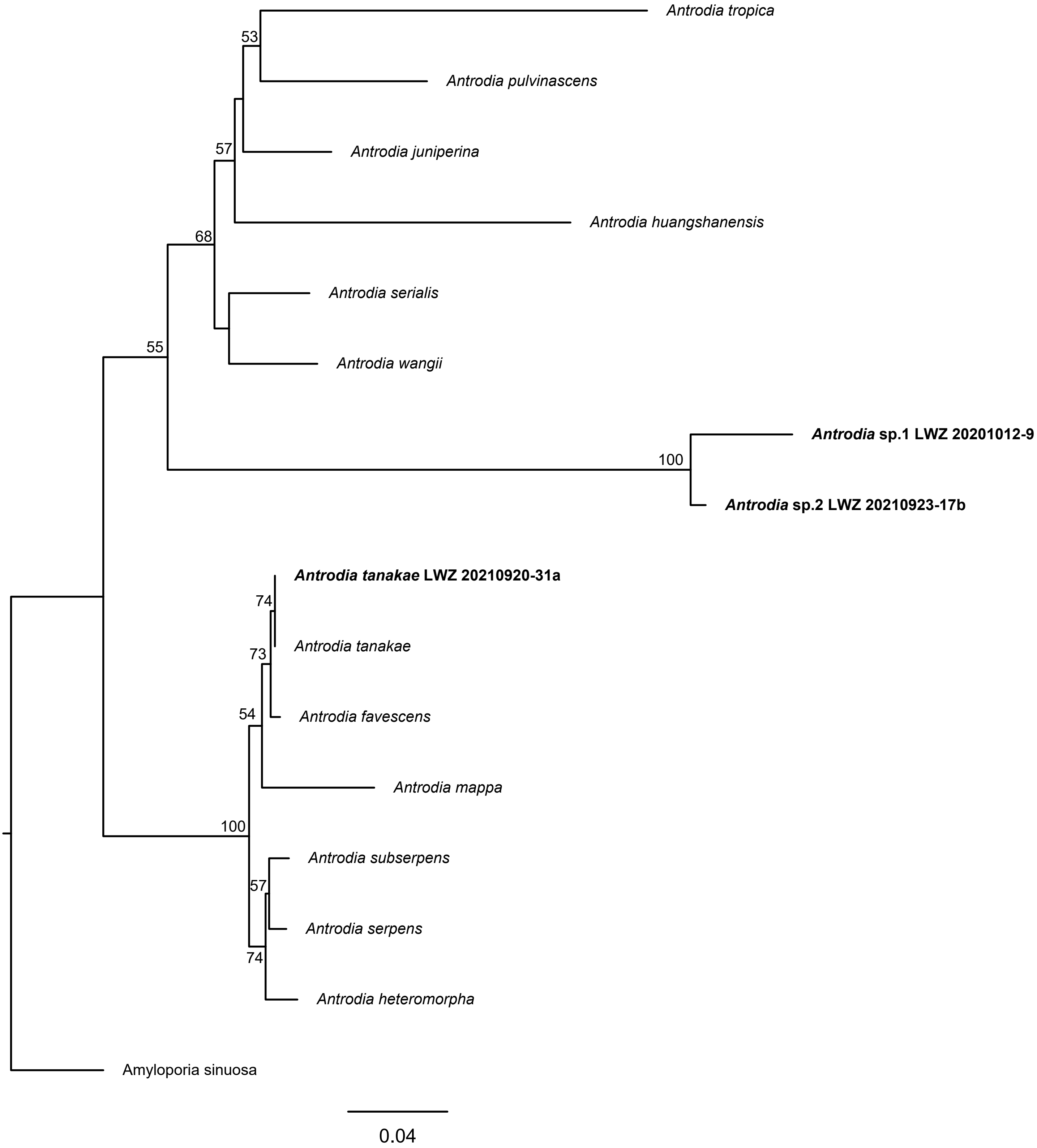

Antrodia tropica
53
Antrodia pulvinascens
57
Antrodia juniperina
Antrodia huangshanensis
68
Antrodia serialis
55
Antrodia wangii
Antrodia sp.1 LWZ 20201012-9
100
Antrodia sp.2 LWZ 20210923-17b
Antrodia tanakae LWZ 20210920-31a
74
Antrodia tanakae
73
Antrodia favescens
54
Antrodia mappa
100
Antrodia subserpens
57
Antrodia serpens
74
Antrodia heteromorpha
Amyloporia sinuosa
0.04

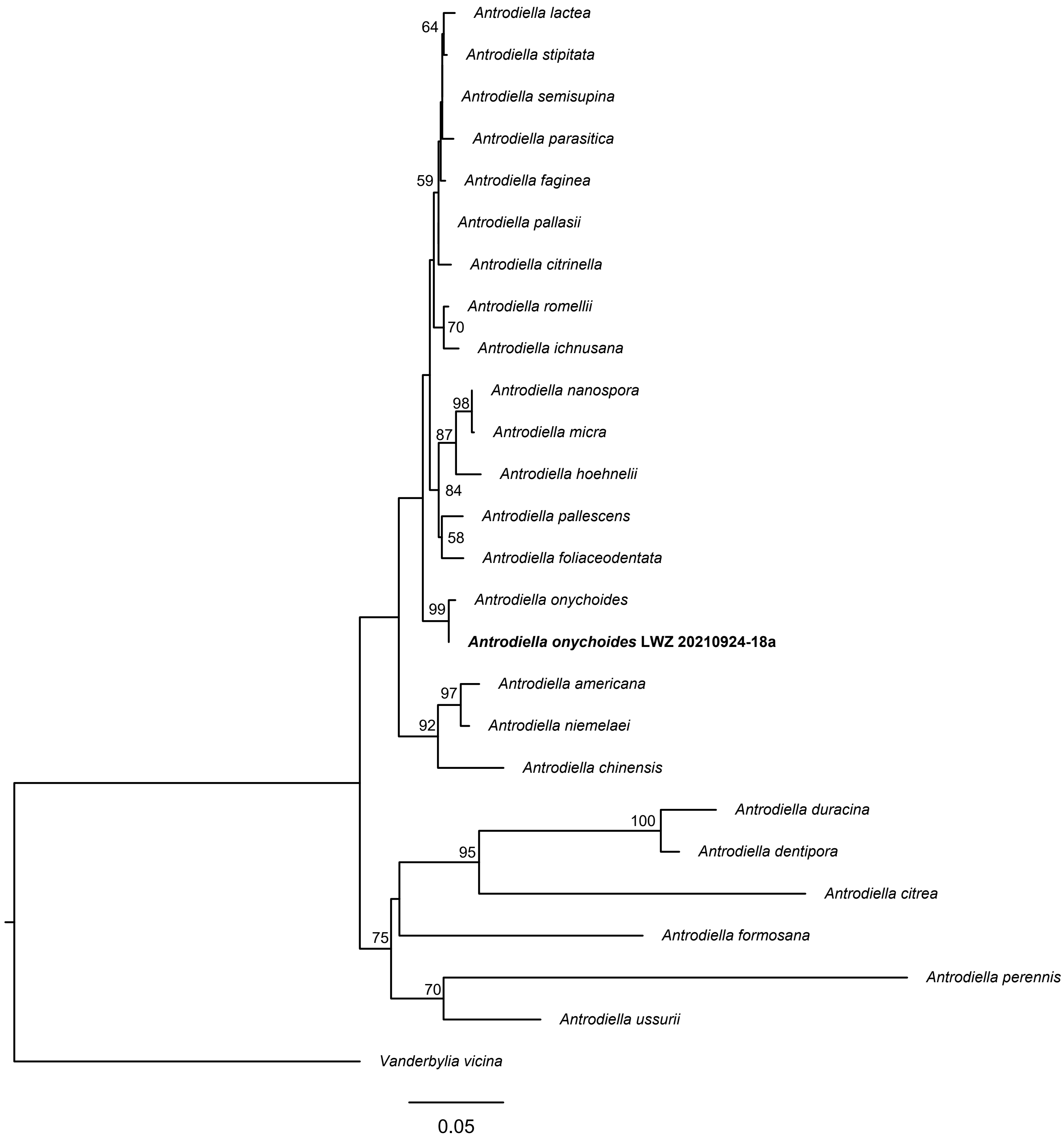
Antrodiella lactea
64
Antrodiella stipitata
Antrodiella semisupina
Antrodiella parasitica
59
Antrodiella faginea
Antrodiella pallasii
Antrodiella citrinella
Antrodiella romellii
70
Antrodiella ichnusana
Antrodiella nanospora
98
Antrodiella micra
87
Antrodiella hoehnelii
84
Antrodiella pallescens
58
Antrodiella foliaceodentata
Antrodiella onychoides
99
Antrodiella onychoides LWZ 20210924-18a
Antrodiella americana
97
Antrodiella niemelaei
92
Antrodiella chinensis
Antrodiella duracina
100
Antrodiella dentipora
95
Antrodiella citrea
Antrodiella formosana
75
Antrodiella perennis
70
Antrodiella ussurii
Vanderbylia vicina
0.05

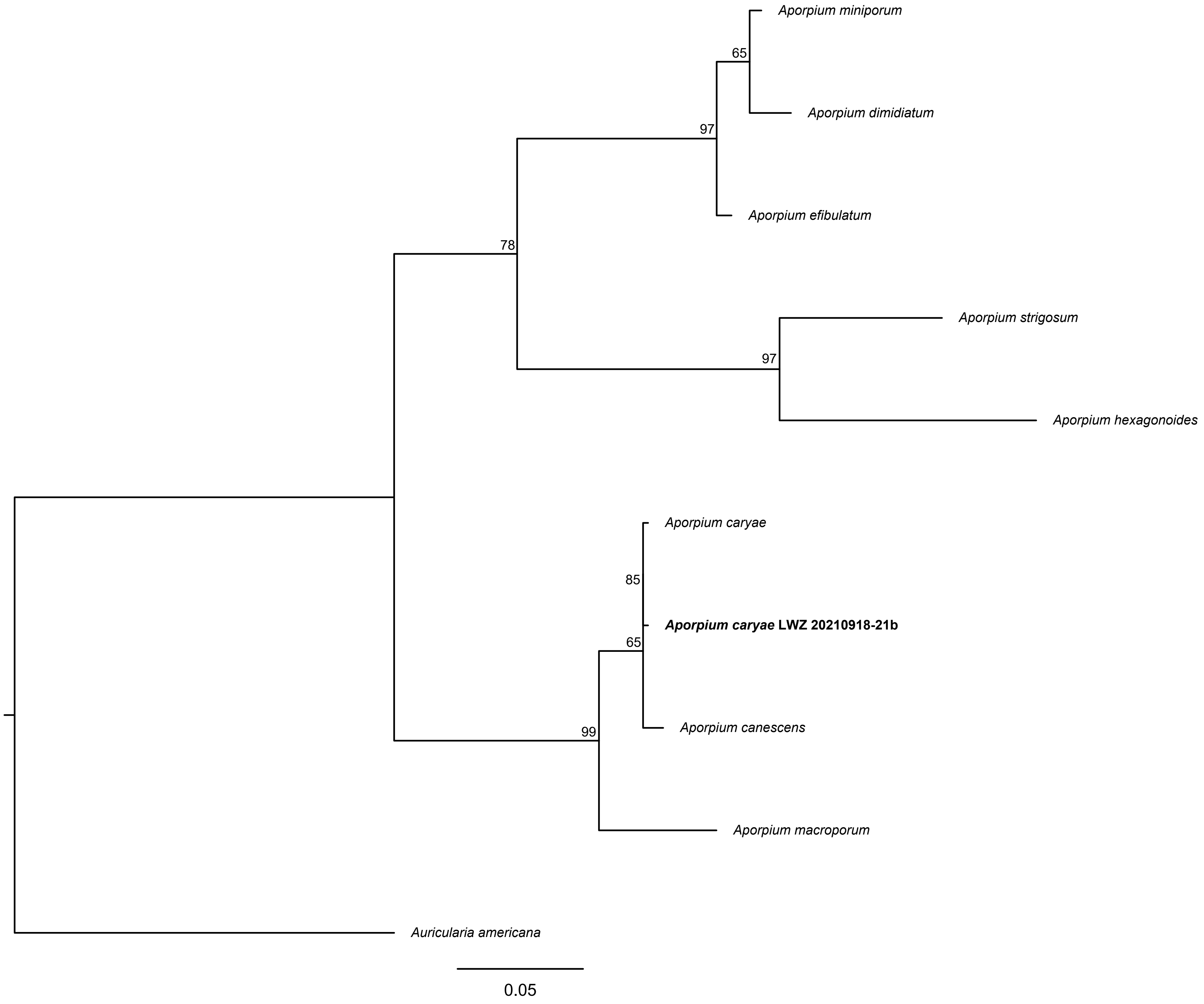
Aporpium miniporum
65
Aporpium dimidiatum
97
Aporpium efibulatum
78
Aporpium strigosum
97
Aporpium hexagonoides
Aporpium caryae
85
Aporpium caryae LWZ 20210918-21b
65
Aporpium canescens
99
Aporpium macroporum
Auricularia americana
0.05

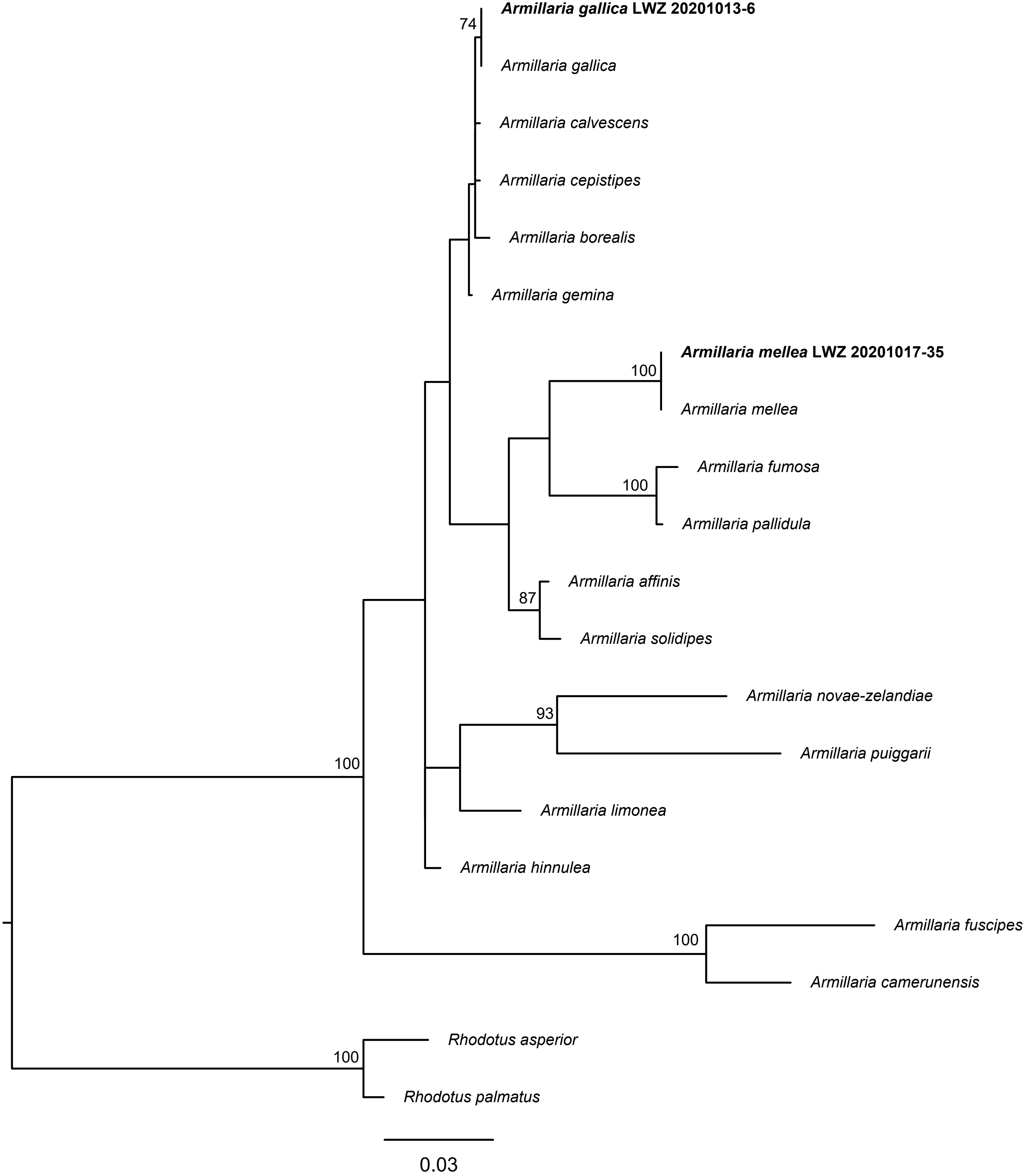
Armillaria gallica LWZ 20201013-6
74
Armillaria gallica
Armillaria calvescens
Armillaria cepistipes
Armillaria borealis
Armillaria gemina
Armillaria mellea LWZ 20201017-35
100
Armillaria mellea
Armillaria fumosa
100
Armillaria pallidula
Armillaria affinis
87
Armillaria solidipes
Armillaria novae-zelandiae
93
Armillaria puiggarii
Armillaria limonea
100
Armillaria hinnulea
Armillaria fuscipes
100
Armillaria camerunensis
Rhodotus asperior
100
Rhodotus palmatus
0.03

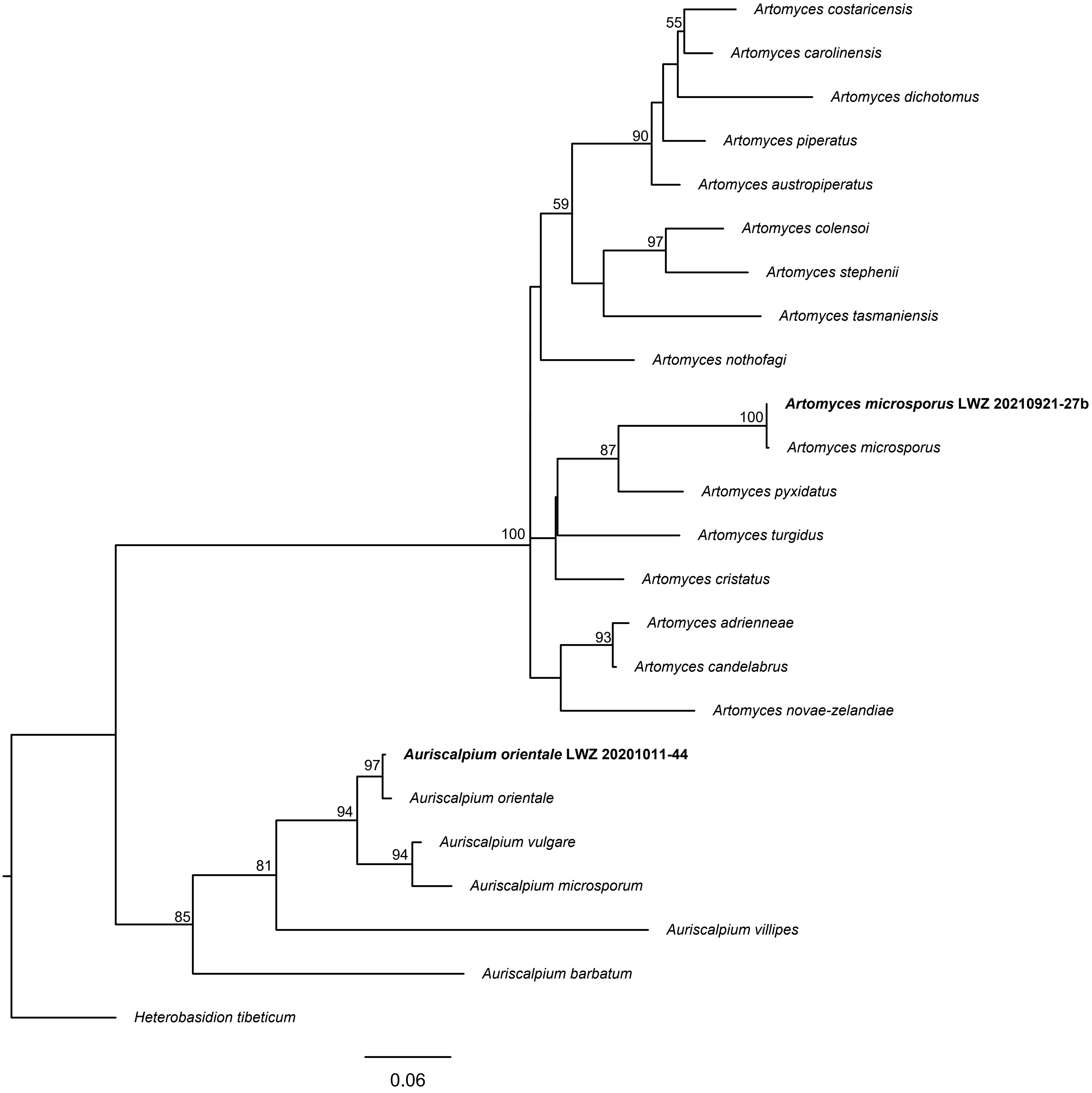

Artomyces costaricensis
55
Artomyces carolinensis
Artomyces dichotomus
90
Artomyces piperatus
Artomyces austropiperatus
59
Artomyces colensoi
97
Artomyces stephenii
Artomyces tasmaniensis
Artomyces nothofagi
Artomyces microsporus LWZ 20210921-27b
100
Artomyces microsporus
87
Artomyces pyxidatus
100
Artomyces turgidus
Artomyces cristatus
Artomyces adrienneae
93
Artomyces candelabrus
Artomyces novae-zelandiae
Auriscalpium orientale LWZ 20201011-44
97
Auriscalpium orientale
94
Auriscalpium vulgare
94
81
Auriscalpium microsporum
85
Auriscalpium villipes
Auriscalpium barbatum
Heterobasidion tibeticum
0.06

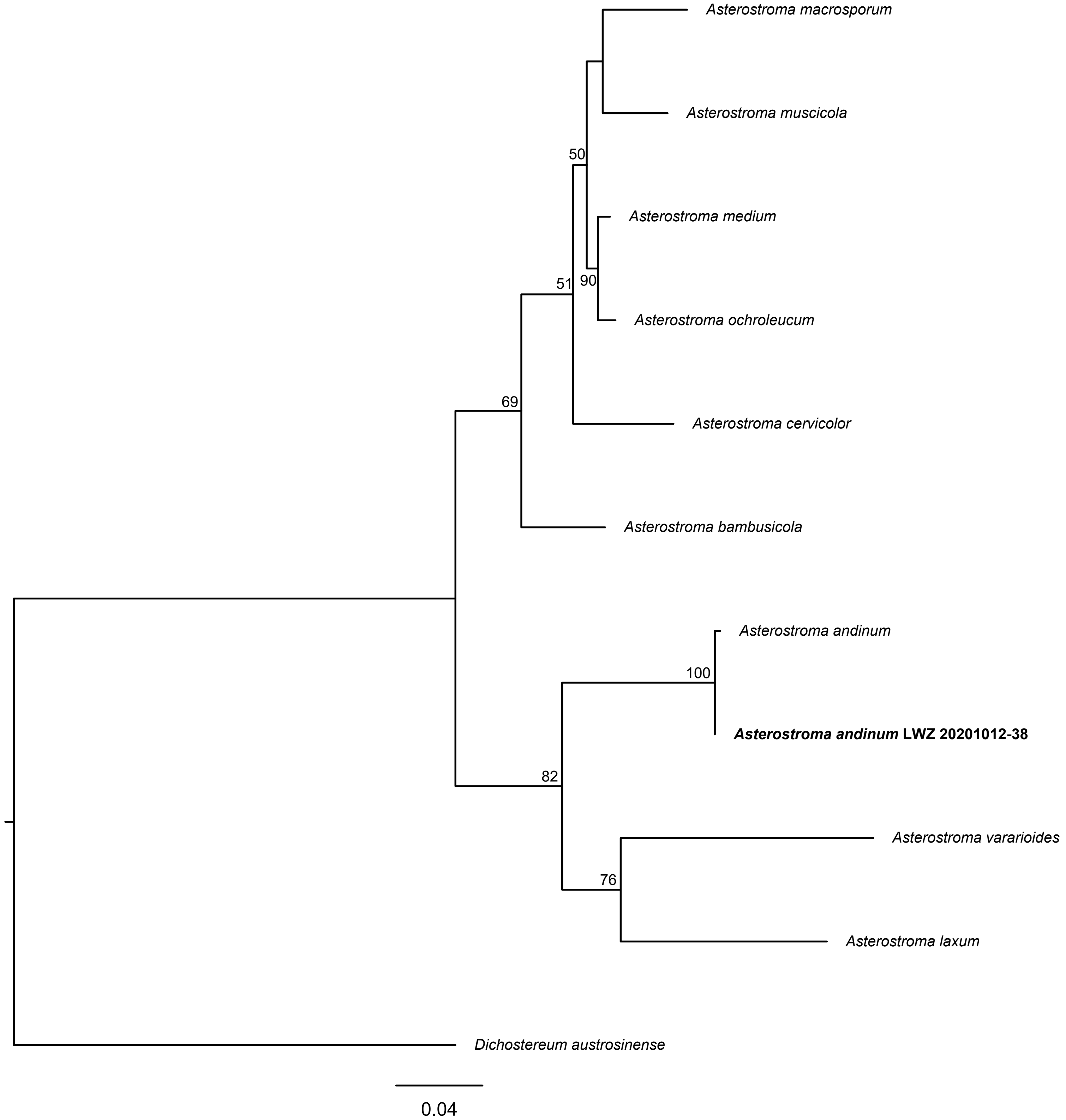

Asterostroma macrosporum
Asterostroma muscicola
50
Asterostroma medium
51
90
Asterostroma ochroleucum
69
Asterostroma cervicolor
Asterostroma bambusicola
Asterostroma andinum
100
Asterostroma andinum LWZ 20201012-38
82
Asterostroma vararioides
76
Asterostroma laxum
Dichostereum austrosinense
0.04

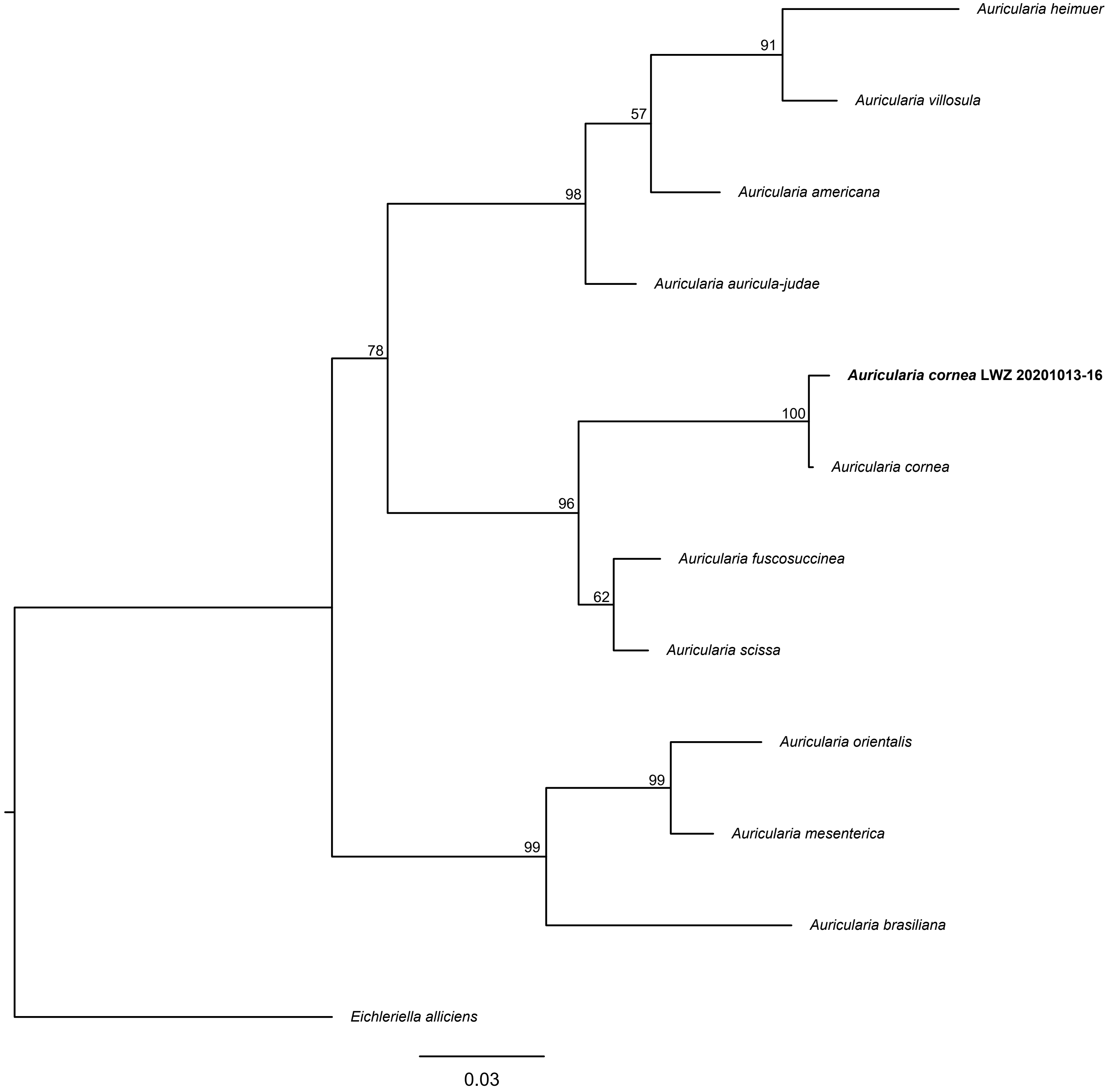

Auricularia heimuer
91
Auricularia villosula
57
Auricularia americana
98
Auricularia auricula-judae
78
Auricularia cornea LWZ 20201013-16
100
Auricularia cornea
96
Auricularia fuscosuccinea
62
Auricularia scissa
Auricularia orientalis
99
Auricularia mesenterica
99
Auricularia brasiliana
Eichleriella alliciens
0.03

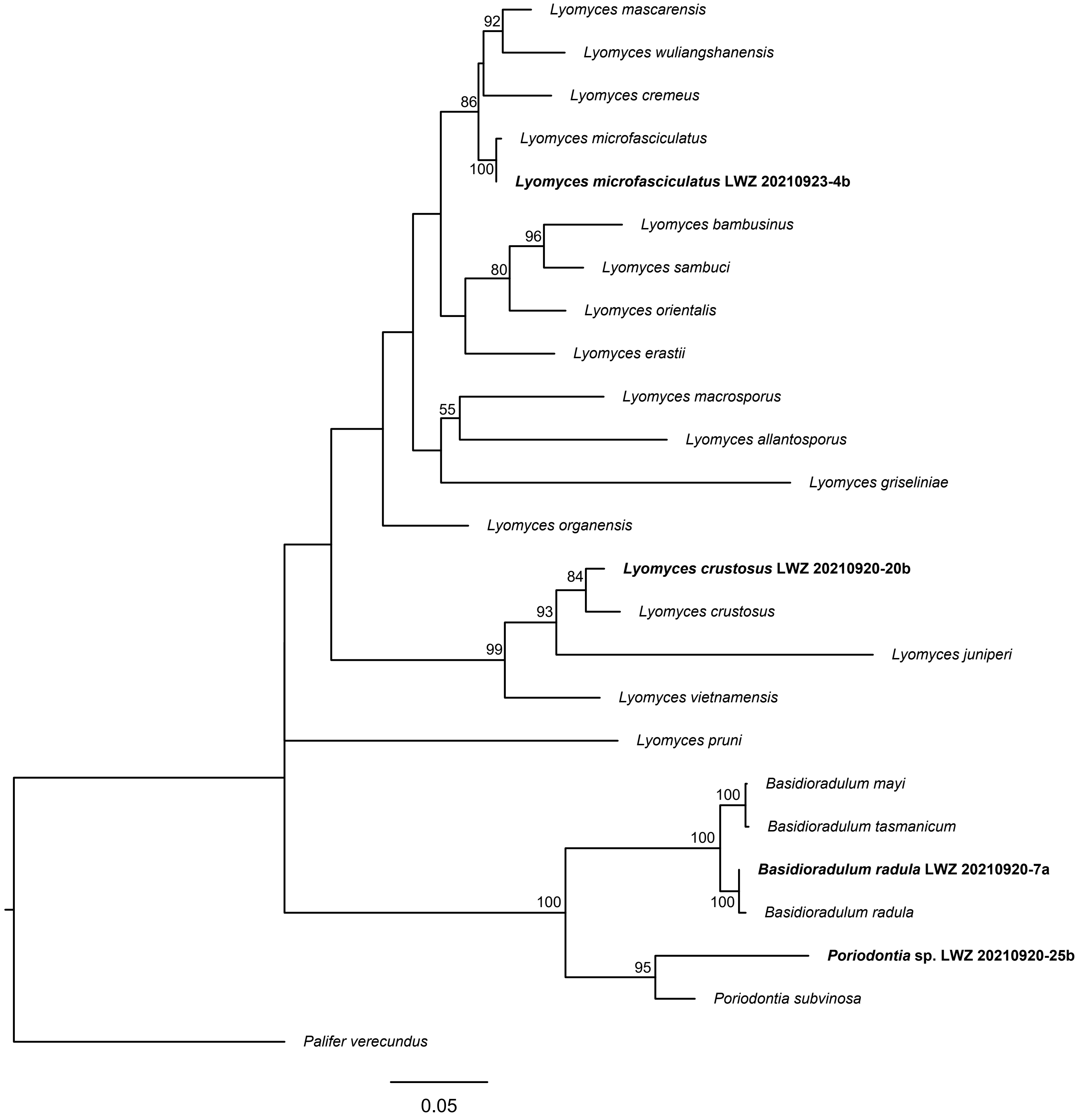

Lyomyces mascarensis
Lyomyces wuliangshanensis
Lyomyces cremeus
Lyomyces microfasciculatus
Lyomyces microfasciculatus LWZ 20210923-4b
Lyomyces bambusinus
Lyomyces sambuci
Lyomyces orientalis
Lyomyces erastii
Lyomyces macrosporus
Lyomyces allantosporus
Lyomyces griseliniae
Lyomyces organensis
Lyomyces crustosus LWZ 20210920-20b
Lyomyces crustosus
Lyomyces juniperi
Lyomyces vietnamensis
Lyomyces pruni
Basidioradulum mayi
Basidioradulum tasmanicum
Basidioradulum radula LWZ 20210920-7a
Basidioradulum radula
Poriodontia sp. LWZ 20210920-25b
Poriodontia subvinosa
Palifer verecundus
92
86
100
96
80
55
84
93
99
100
100
100
100
95
0.05

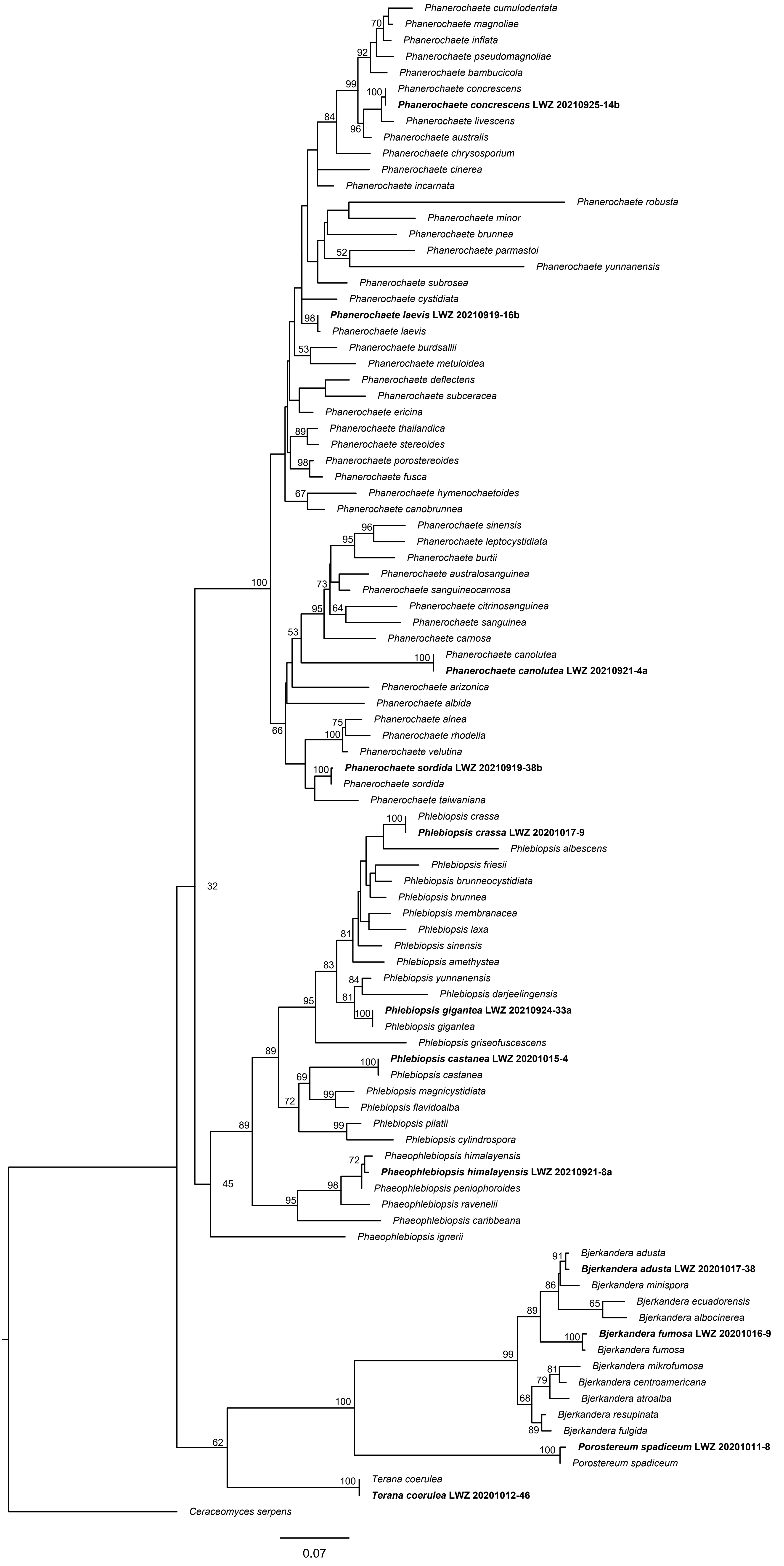

Phanerochaete cumulodentata
Phanerochaete magnoliae
Phanerochaete inflata
Phanerochaete pseudomagnoliae
Phanerochaete bambucicola
Phanerochaete concrescens
Phanerochaete concrescens LWZ 20210925-14b
Phanerochaete livescens
Phanerochaete australis
Phanerochaete chrysosporium
Phanerochaete cinerea
Phanerochaete incarnata
Phanerochaete robusta
Phanerochaete minor
Phanerochaete brunnea
Phanerochaete parmastoi
Phanerochaete yunnanensis
Phanerochaete subrosea
Phanerochaete cystidiata
Phanerochaete laevis LWZ 20210919-16b
Phanerochaete laevis
Phanerochaete burdsallii
Phanerochaete metuloidea
Phanerochaete deflectens
Phanerochaete subceracea
Phanerochaete ericina
Phanerochaete thailandica
Phanerochaete stereoides
Phanerochaete porostereoides
Phanerochaete fusca
Phanerochaete hymenochaetoides
Phanerochaete canobrunnea
Phanerochaete sinensis
Phanerochaete leptocystidiata
Phanerochaete burtii
Phanerochaete australosanguinea
Phanerochaete sanguineocarnosa
Phanerochaete citrinosanguinea
Phanerochaete sanguinea
Phanerochaete carnosa
Phanerochaete canolutea
Phanerochaete canolutea LWZ 20210921-4a
Phanerochaete arizonica
Phanerochaete albida
Phanerochaete alnea
Phanerochaete rhodella
Phanerochaete velutina
Phanerochaete sordida LWZ 20210919-38b
Phanerochaete sordida
Phanerochaete taiwaniana
Phlebiopsis crassa
Phlebiopsis crassa LWZ 20201017-9
Phlebiopsis albescens
Phlebiopsis friesii
Phlebiopsis brunneocystidiata
Phlebiopsis brunnea
Phlebiopsis membranacea
Phlebiopsis laxa
Phlebiopsis sinensis
Phlebiopsis amethystea
Phlebiopsis yunnanensis
Phlebiopsis darjeelingensis
Phlebiopsis gigantea LWZ 20210924-33a
Phlebiopsis gigantea
Phlebiopsis griseofuscescens
Phlebiopsis castanea LWZ 20201015-4
Phlebiopsis castanea
Phlebiopsis magnicystidiata
Phlebiopsis flavidoalba
Phlebiopsis pilatii
Phlebiopsis cylindrospora
Phaeophlebiopsis himalayensis
Phaeophlebiopsis himalayensis LWZ 20210921-8a
Phaeophlebiopsis peniophoroides
Phaeophlebiopsis ravenelii
Phaeophlebiopsis caribbeana
Phaeophlebiopsis ignerii
Bjerkandera adusta
Bjerkandera adusta LWZ 20201017-38
Bjerkandera minispora
Bjerkandera ecuadorensis
Bjerkandera albocinerea
Bjerkandera fumosa LWZ 20201016-9
Bjerkandera fumosa
Bjerkandera mikrofumosa
Bjerkandera centroamericana
Bjerkandera atroalba
Bjerkandera resupinata
Bjerkandera fulgida
Porostereum spadiceum LWZ 20201011-8
Porostereum spadiceum
Terana coerulea
Terana coerulea LWZ 20201012-46
Ceraceomyces serpens
0.07

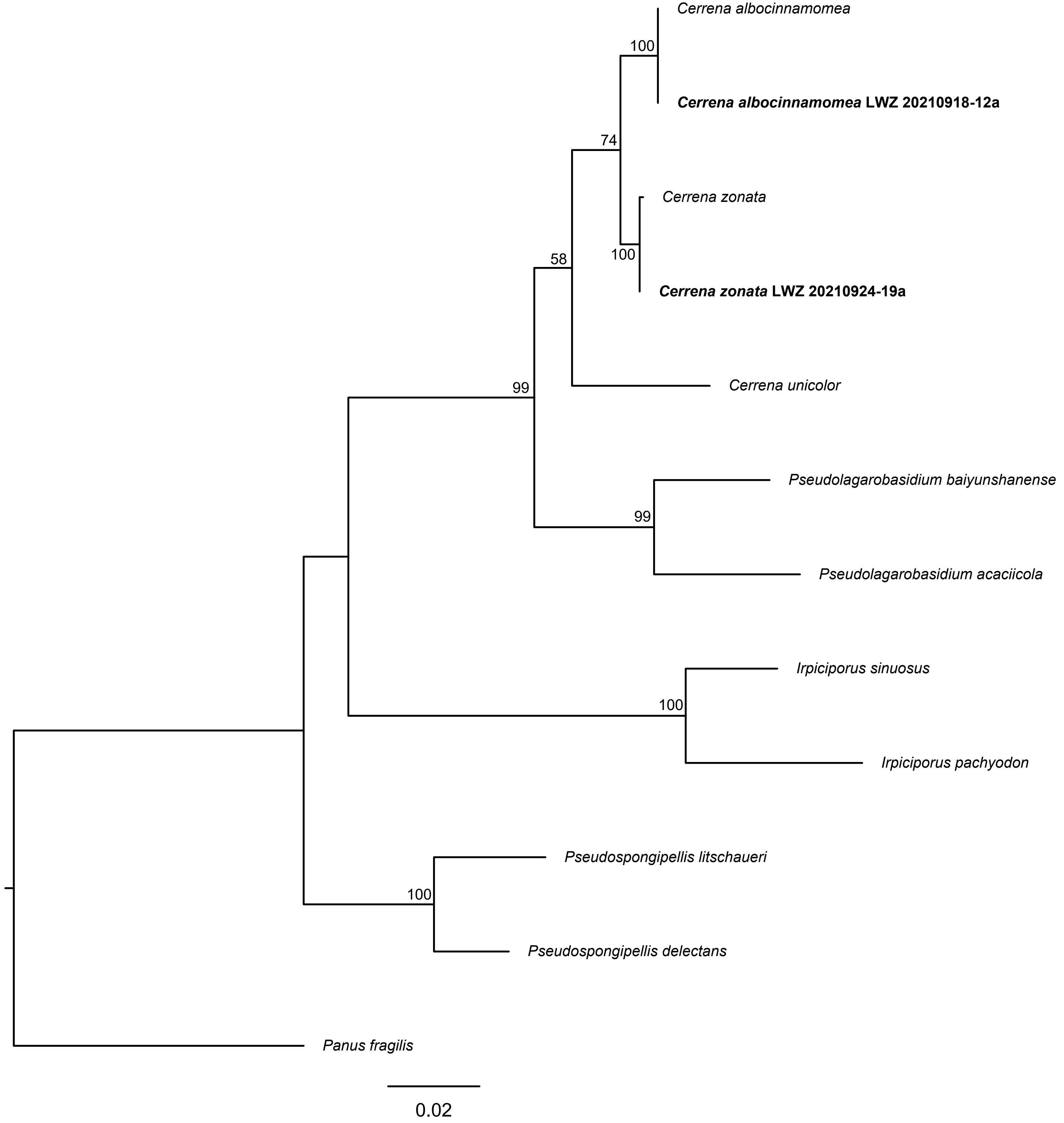

Cerrena albocinnamomea
100
Cerrena albocinnamomea LWZ 20210918-12a
74
Cerrena zonata
100
Cerrena zonata LWZ 20210924-19a
58
Cerrena unicolor
99
Pseudolagarobasidium baiyunshanense
99
Pseudolagarobasidium acaciicola
Irpiciporus sinuosus
100
Irpiciporus pachyodon
Pseudospongipellis litschaueri
100
Pseudospongipellis delectans
Panus fragilis
0.02

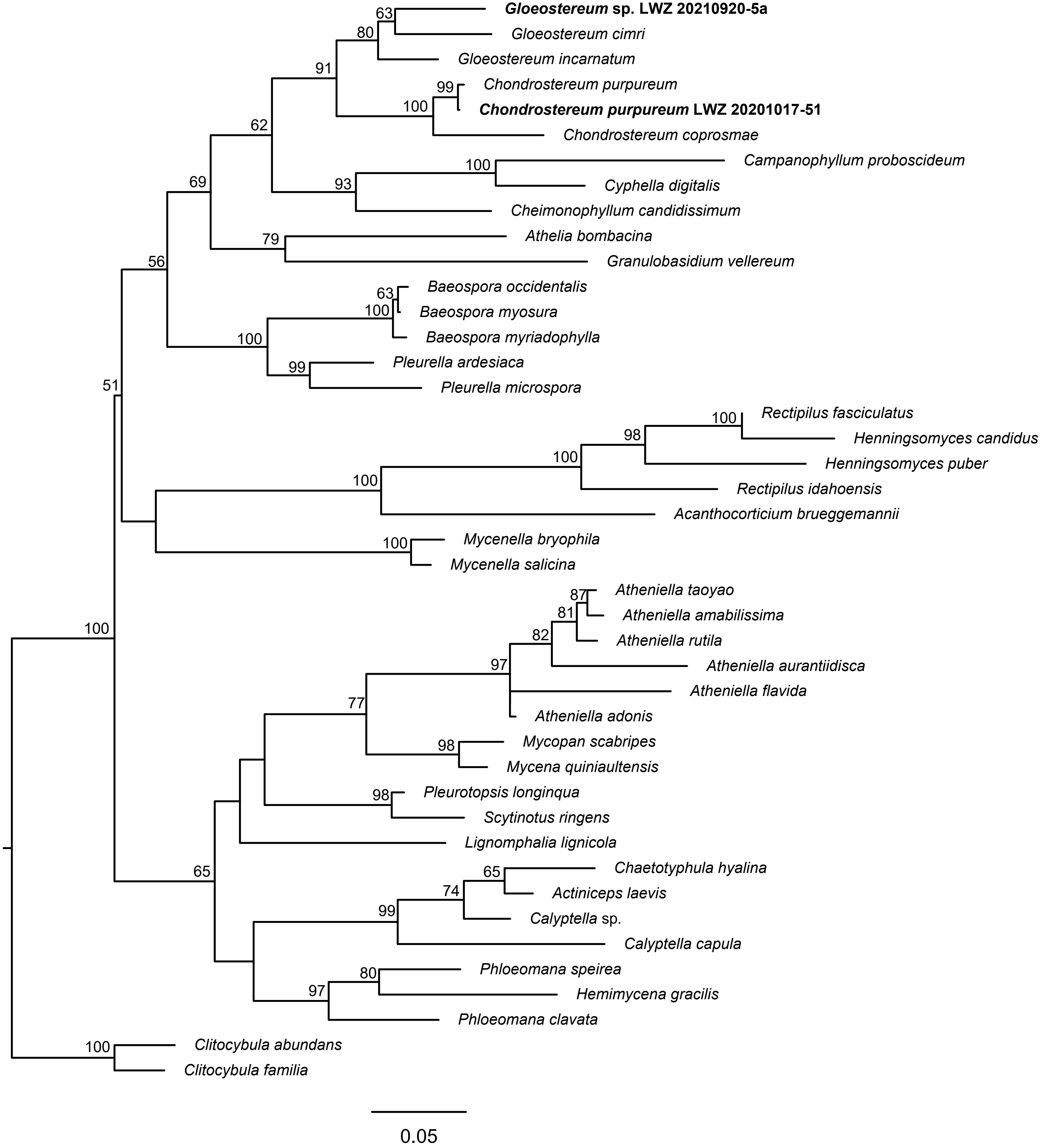

Gloeostereum sp. LWZ 20210920-5a
Gloeostereum cimri
Gloeostereum incarnatum
Chondrostereum purpureum
Chondrostereum purpureum LWZ 20201017-51
Chondrostereum coprosmae
Campanophyllum proboscideum
Cyphella digitalis
Cheimonophyllum candidissimum
Athelia bombacina
Granulobasidium vellereum
Baeospora occidentalis
Baeospora myosura
Baeospora myriadophylla
Pleurella ardesiaca
Pleurella microspora
Rectipilus fasciculatus
Henningsomyces candidus
Henningsomyces puber
Rectipilus idahoensis
Acanthocorticium brueggemannii
Mycenella bryophila
Mycenella salicina
Atheniella taoyao
Atheniella amabilissima
Atheniella rutila
Atheniella aurantiidisca
Atheniella flavida
Atheniella adonis
Mycopan scabripes
Mycena quiniaultensis
Pleurotopsis longinqua
Scytinotus ringens
Lignomphalia lignicola
Chaetotyphula hyalina
Actiniceps laevis
Calyptella sp.
Calyptella capula
Phloeomana speirea
Hemimycena gracilis
Phloeomana clavata
Clitocybula abundans
Clitocybula familia
0.05

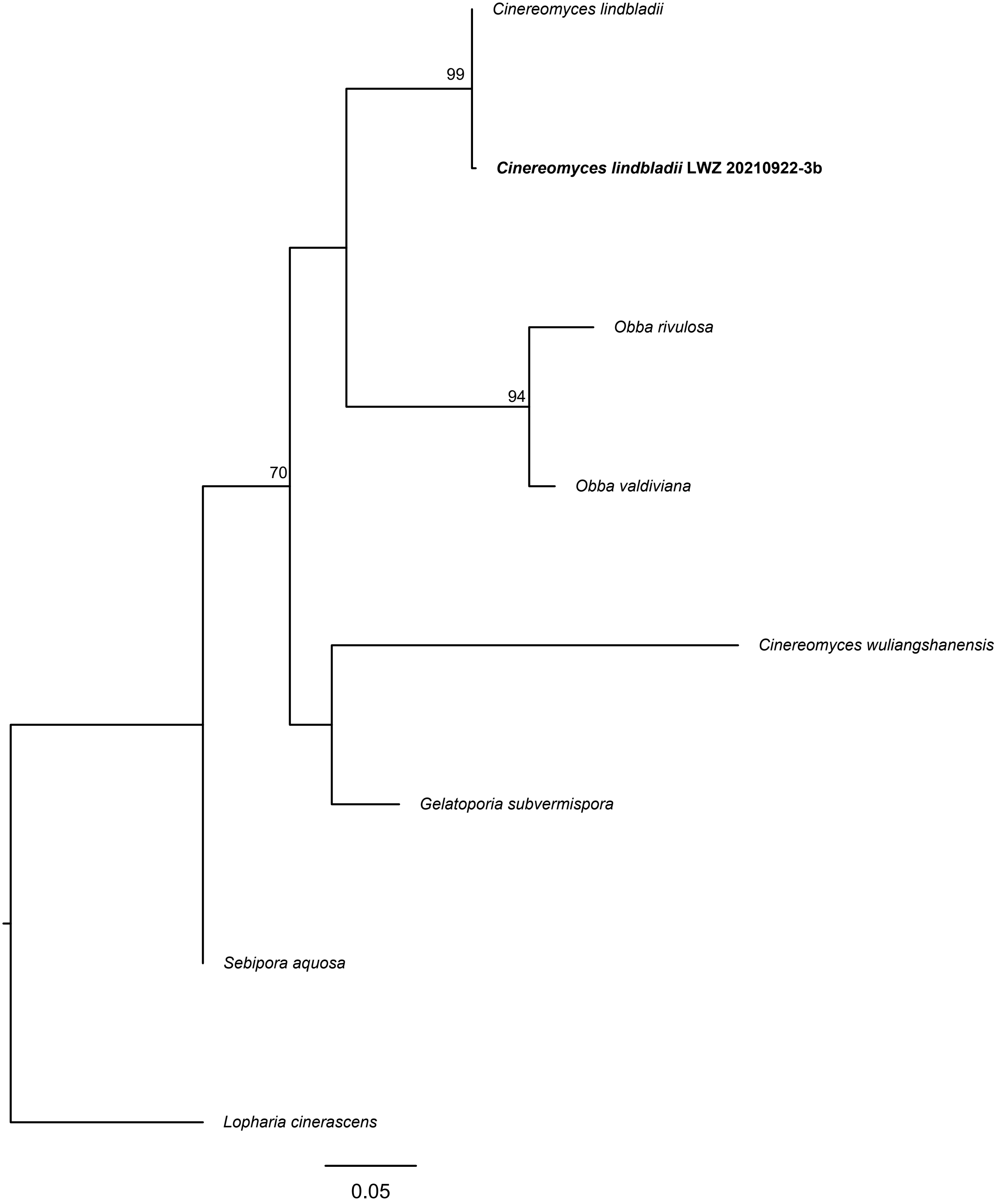

Cinereomyces lindbladii
99
Cinereomyces lindbladii LWZ 20210922-3b
Obba rivulosa
94
70
Obba valdiviana
Cinereomyces wuliangshanensis
Gelatoporia subvermispora
Sebipora aquosa
Lopharia cinerascens
0.05

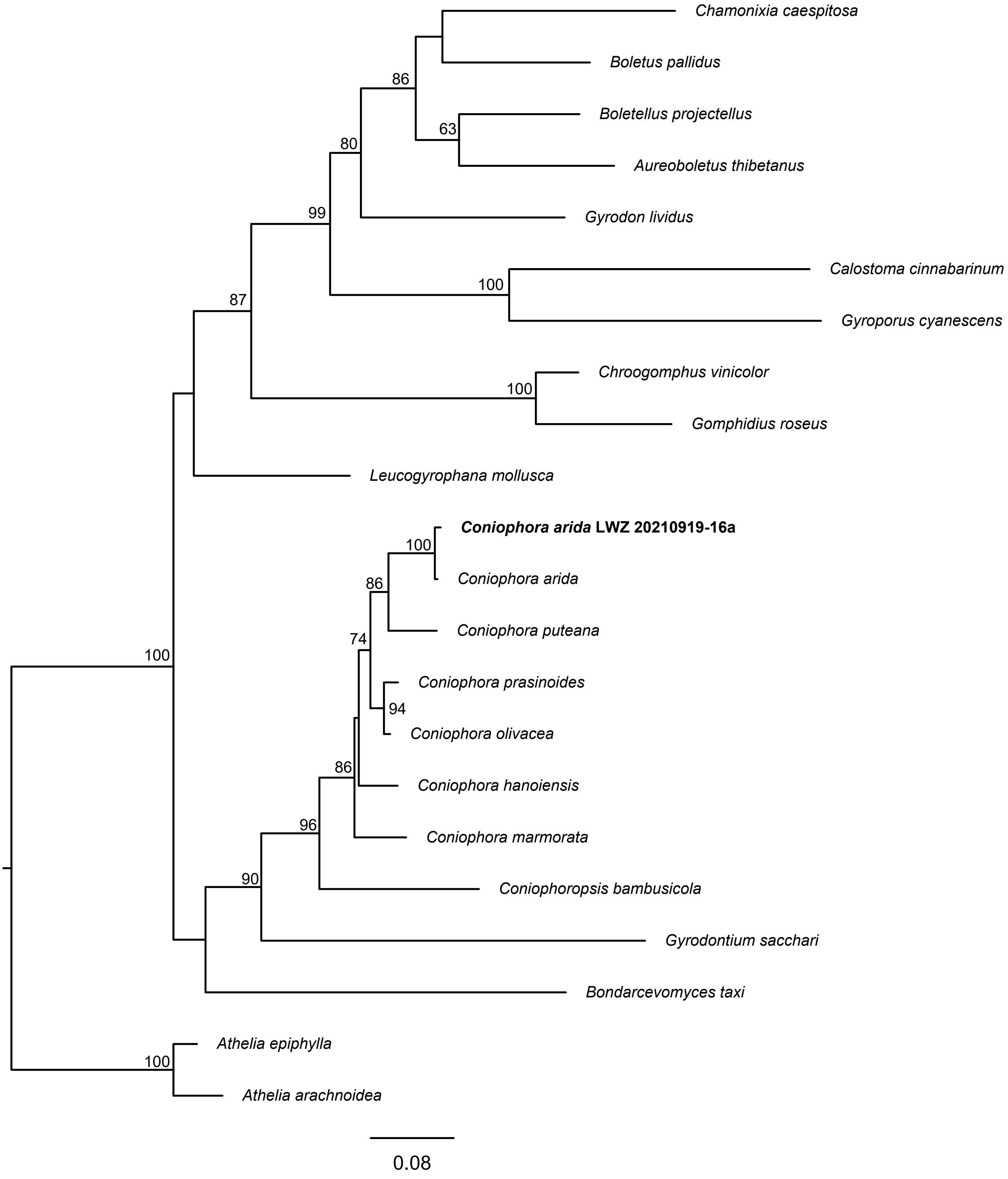

Chamonixia caespitosa
Boletus pallidus
86
Boletellus projectellus
63
Aureoboletus thibetanus
80
99
Gyrodon lividus
Calostoma cinnabarinum
100
Gyroporus cyanescens
87
Chroogomphus vinicolor
100
Gomphidius roseus
Leucogyrophana mollusca
Coniophora arida LWZ 20210919-16a
100
Coniophora arida
86
Coniophora puteana
74
Coniophora prasinoides
94
Coniophora olivacea
86
Coniophora hanoiensis
96
Coniophora marmorata
90
Coniophoropsis bambusicola
Gyrodontium sacchari
100
Bondarcevomyces taxi
Athelia epiphylla
100
Athelia arachnoidea
0.08

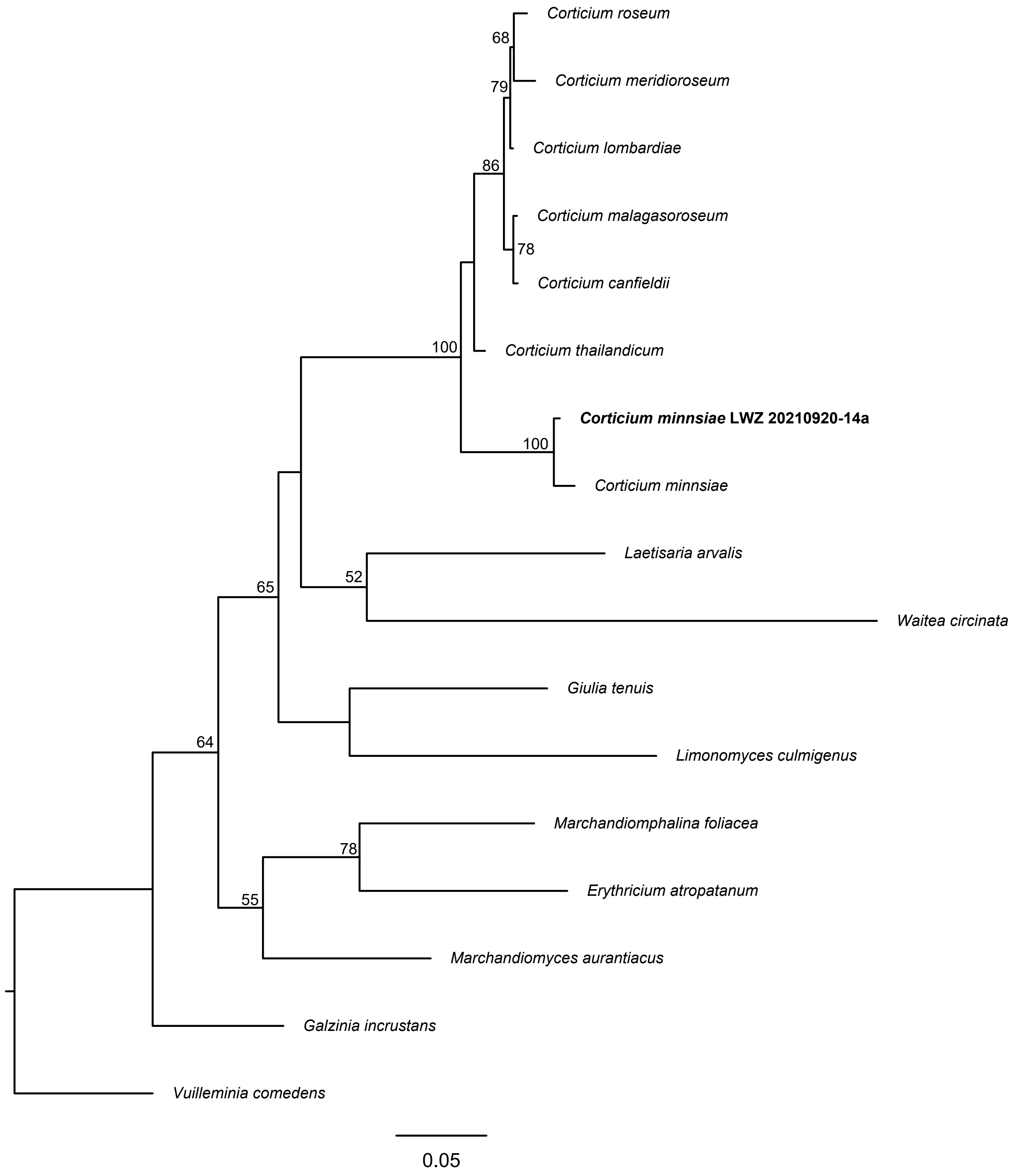

Corticium roseum
68
Corticium meridioroseum
79
Corticium lombardiae
86
Corticium malagasoroseum
78
Corticium canfieldii
Corticium thailandicum
100
Corticium minnsiae LWZ 20210920-14a
100
Corticium minnsiae
Laetisaria arvalis
52
Waitea circinata
65
Giulia tenuis
Limonomyces culmigenus
64
Marchandiomphalina foliacea
78
Erythricium atropatanum
55
Marchandiomyces aurantiacus
Galzinia incrustans
Vuilleminia comedens
0.05

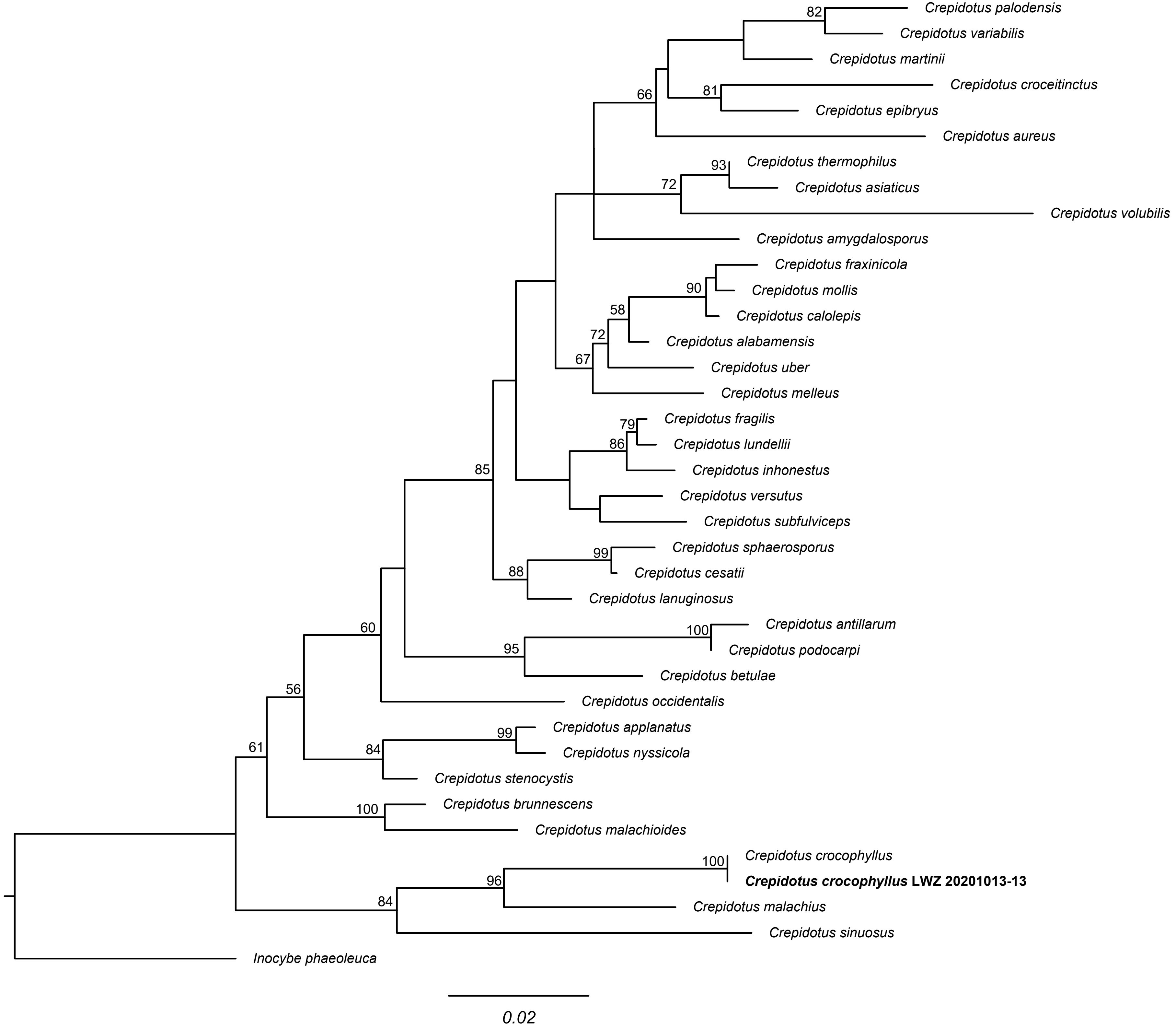

Crepidotus palodensis
Crepidotus variabilis
Crepidotus martinii
Crepidotus croceitinctus
Crepidotus epibryus
Crepidotus aureus
Crepidotus thermophilus
Crepidotus asiaticus
Crepidotus volubilis
Crepidotus amygdalosporus
Crepidotus fraxinicola
Crepidotus mollis
Crepidotus calolepis
Crepidotus alabamensis
Crepidotus uber
Crepidotus melleus
Crepidotus fragilis
Crepidotus lundellii
Crepidotus inhonestus
Crepidotus versutus
Crepidotus subfulviceps
Crepidotus sphaerosporus
Crepidotus cesatii
Crepidotus lanuginosus
Crepidotus antillarum
Crepidotus podocarpi
Crepidotus betulae
Crepidotus occidentalis
Crepidotus applanatus
Crepidotus nyssicola
Crepidotus stenocystis
Crepidotus brunnescens
Crepidotus malachioides
Crepidotus crocophyllus
Crepidotus crocophyllus LWZ 20201013-13
Crepidotus malachius
Crepidotus sinuosus
Inocybe phaeoleuca
82
81
66
93
72
90
58
72
67
79
86
85
99
88
100
95
60
56
99
84
61
100
100
96
84
0.02

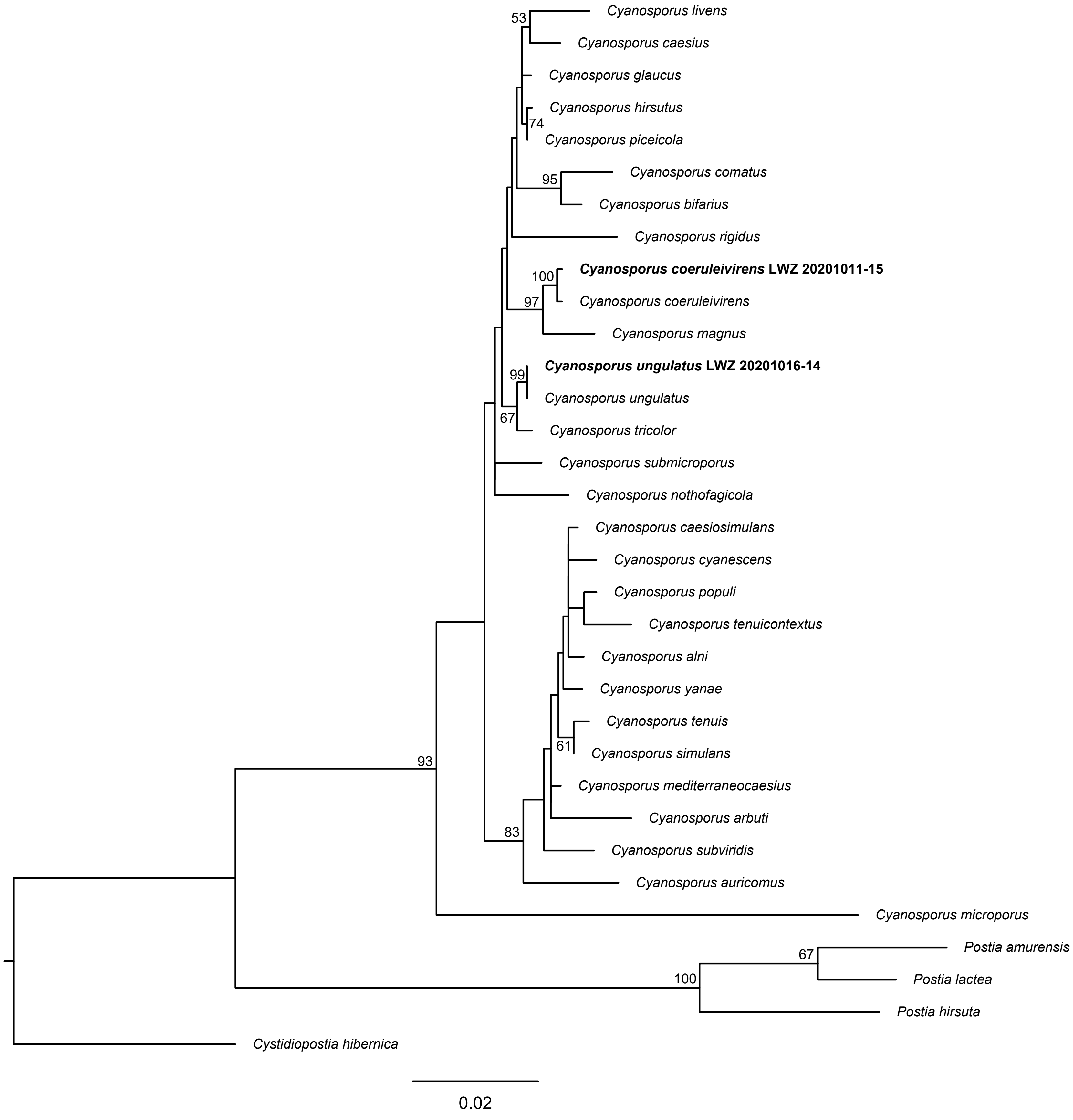
Cyanosporus livens
Cyanosporus caesius
Cyanosporus glaucus
Cyanosporus hirsutus
Cyanosporus piceicola
Cyanosporus comatus
Cyanosporus bifarius
Cyanosporus rigidus
Cyanosporus coeruleivirens LWZ 20201011-15
Cyanosporus coeruleivirens
Cyanosporus magnus
Cyanosporus ungulatus LWZ 20201016-14
Cyanosporus ungulatus
Cyanosporus tricolor
Cyanosporus submicroporus
Cyanosporus nothofagicola
Cyanosporus caesiosimulans
Cyanosporus cyanescens
Cyanosporus populi
Cyanosporus tenuicontextus
Cyanosporus alni
Cyanosporus yanae
Cyanosporus tenuis
Cyanosporus simulans
Cyanosporus mediterraneocaesius
Cyanosporus arbuti
Cyanosporus subviridis
Cyanosporus auricomus
Cyanosporus microporus
Postia amurensis
Postia lactea
Postia hirsuta
Cystidiopostia hibernica
53
74
95
100
97
99
67
61
83
93
67
100
0.02

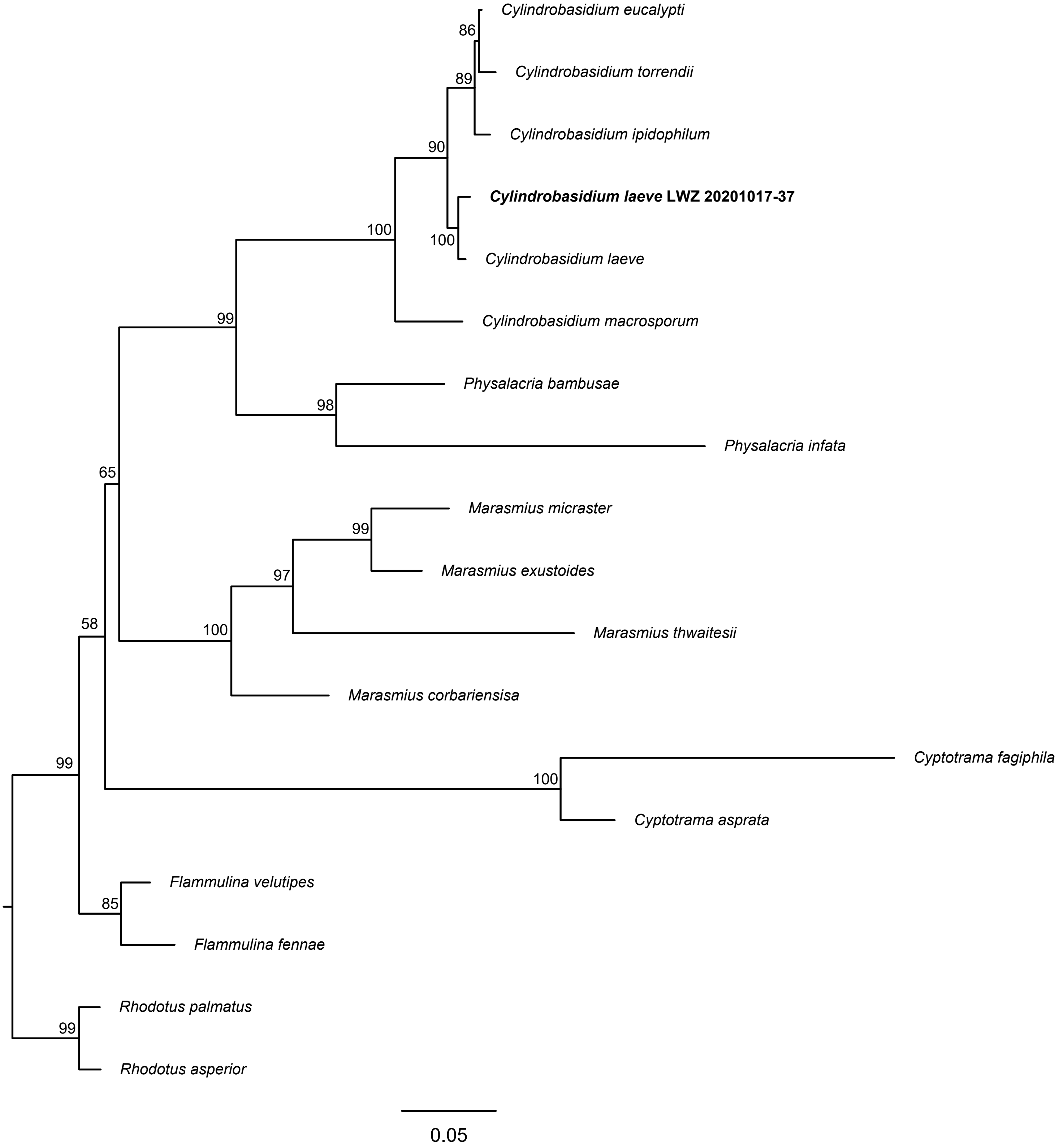

Cylindrobasidium eucalypti
86
Cylindrobasidium torrendii
89
Cylindrobasidium ipidophilum
90
Cylindrobasidium laeve LWZ 20201017-37
100
100
Cylindrobasidium laeve
99
Cylindrobasidium macrosporum
Physalacria bambusae
98
Physalacria infata
65
Marasmius micraster
99
Marasmius exustoides
97
58
100
Marasmius thwaitesii
Marasmius corbariensisa
99
Cyptotrama fagiphila
100
Cyptotrama asprata
Flammulina velutipes
85
Flammulina fennae
Rhodotus palmatus
99
Rhodotus asperior
0.05

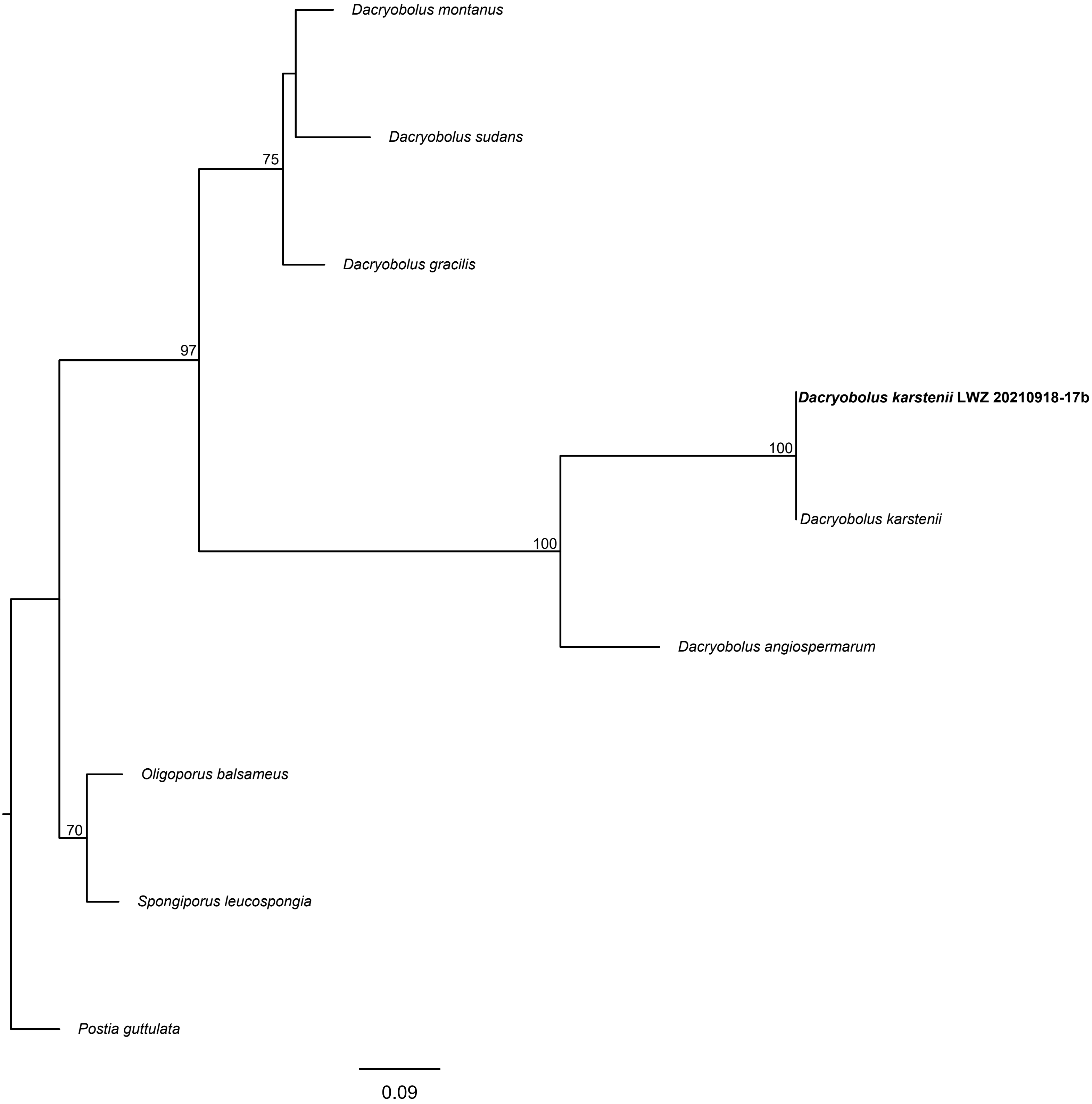
Dacryobolus montanus
Dacryobolus sudans
75
Dacryobolus gracilis
97
Dacryobolus karstenii **LWZ 20210918-17b**
100
Dacryobolus karstenii
100
Dacryobolus angiospermarum
Oligoporus balsameus
70
Spongiporus leucospongia
Postia guttulata
0.09

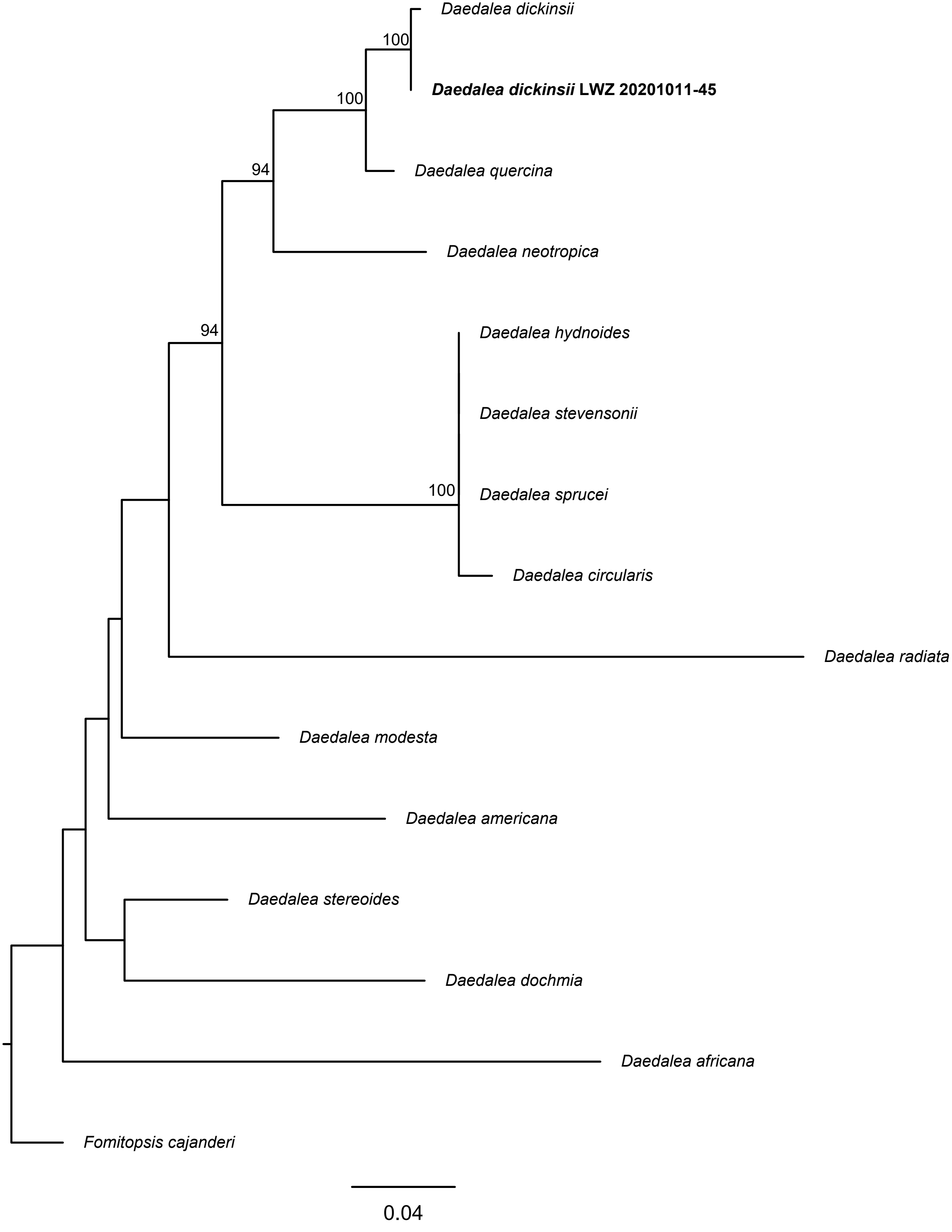

Daedalea dickinsii
Daedalea dickinsii LWZ 20201011-45
Daedalea quercina
Daedalea neotropica
Daedalea hydnoides
Daedalea stevensonii
Daedalea sprucei
Daedalea circularis
Daedalea radiata
Daedalea modesta
Daedalea americana
Daedalea stereoides
Daedalea dochmia
Daedalea africana
Fomitopsis cajanderi
100
100
94
94
100
0.04

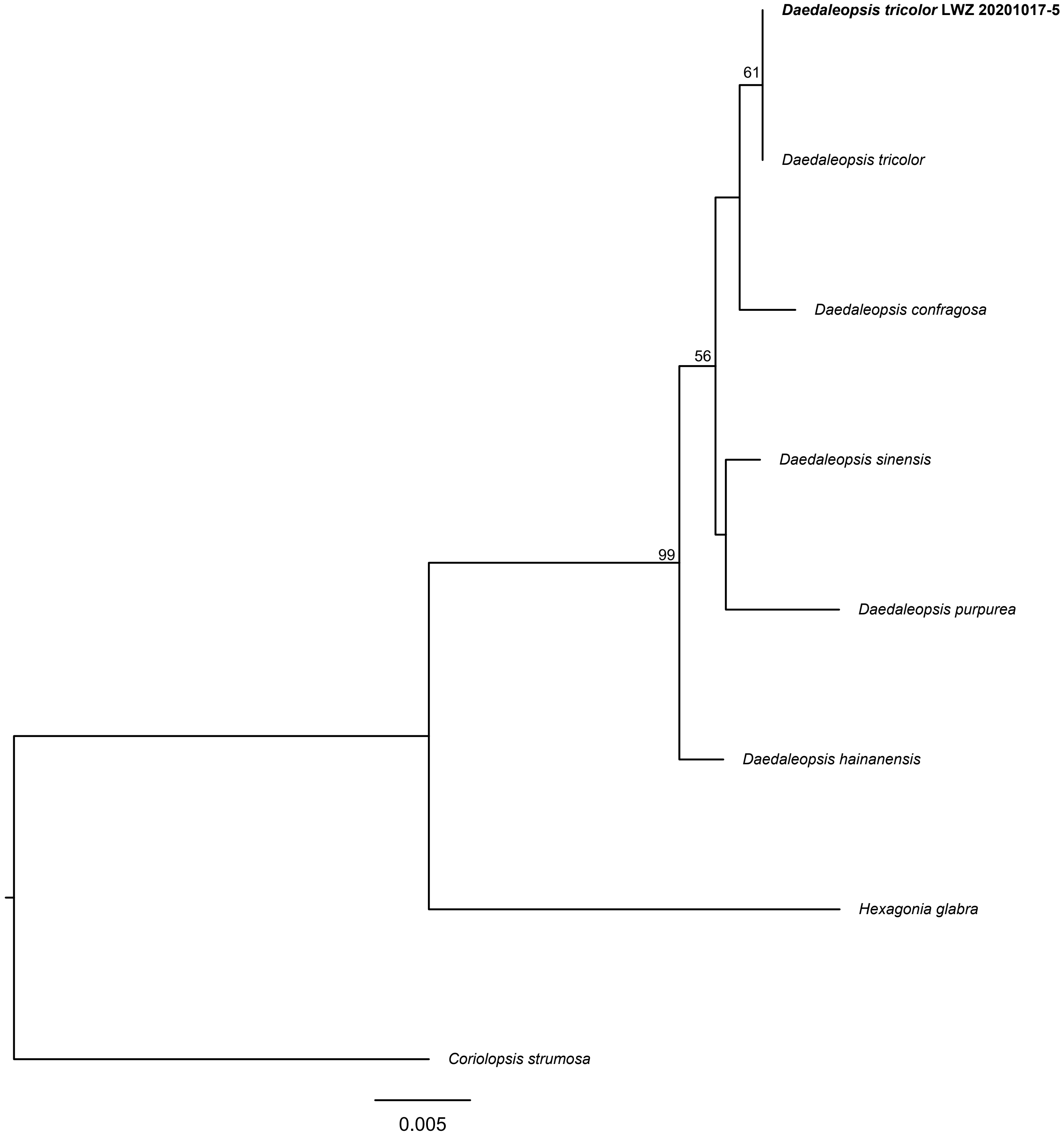

Daedaleopsis tricolor LWZ 20201017-5
61
Daedaleopsis tricolor
Daedaleopsis confragosa
56
Daedaleopsis sinensis
99
Daedaleopsis purpurea
Daedaleopsis hainanensis
Hexagonia glabra
Coriolopsis strumosa
0.005

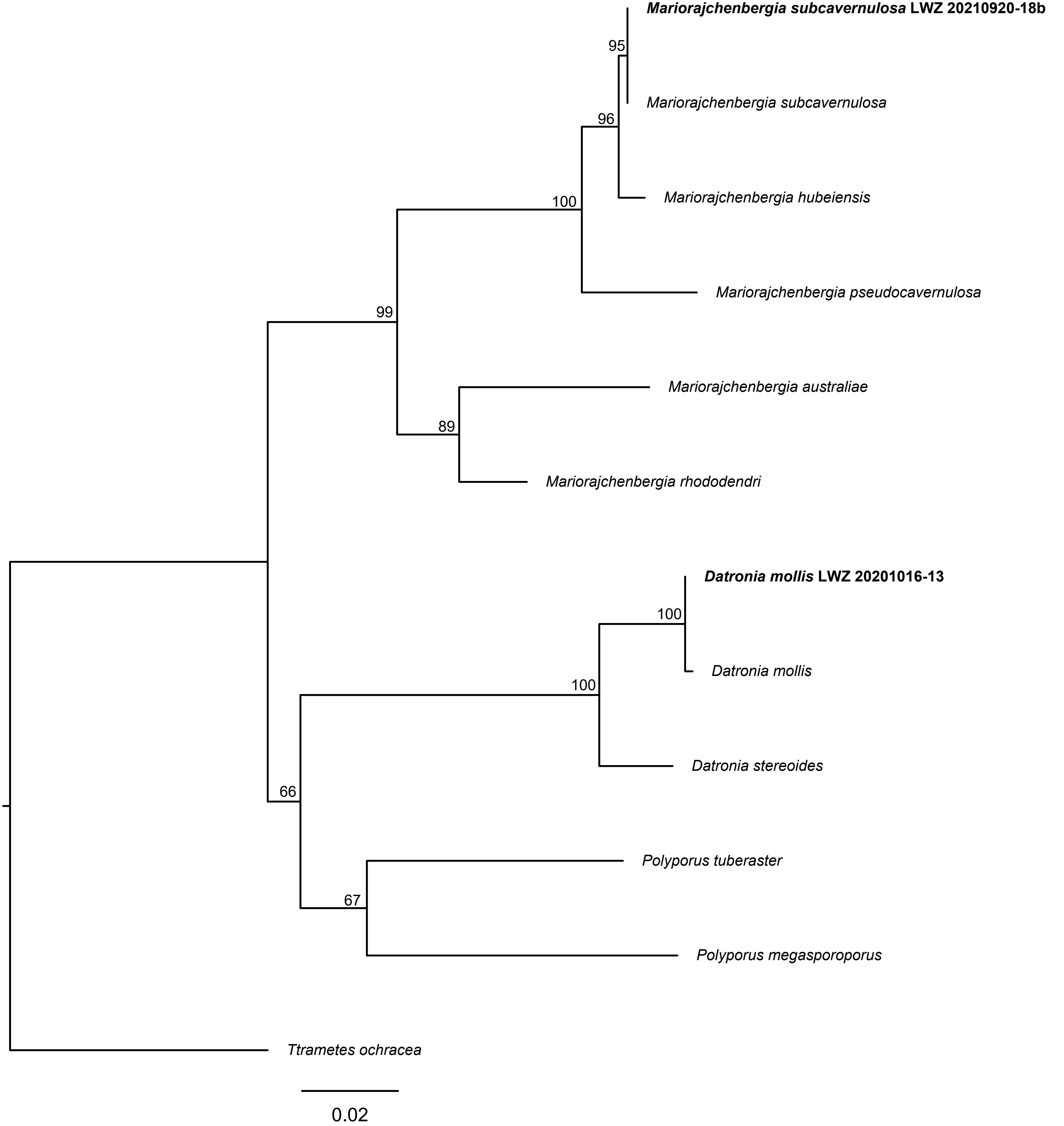

Mariorajchenbergia subcavernulosa LWZ 20210920-18b
95
Mariorajchenbergia subcavernulosa
96
Mariorajchenbergia hubeiensis
100
Mariorajchenbergia pseudocavernulosa
99
Mariorajchenbergia australiae
89
Mariorajchenbergia rhododendri
Datronia mollis LWZ 20201016-13
100
Datronia mollis
100
Datronia stereoides
66
Polyporus tuberaster
67
Polyporus megasporoporus
Ttrametes ochracea
0.02

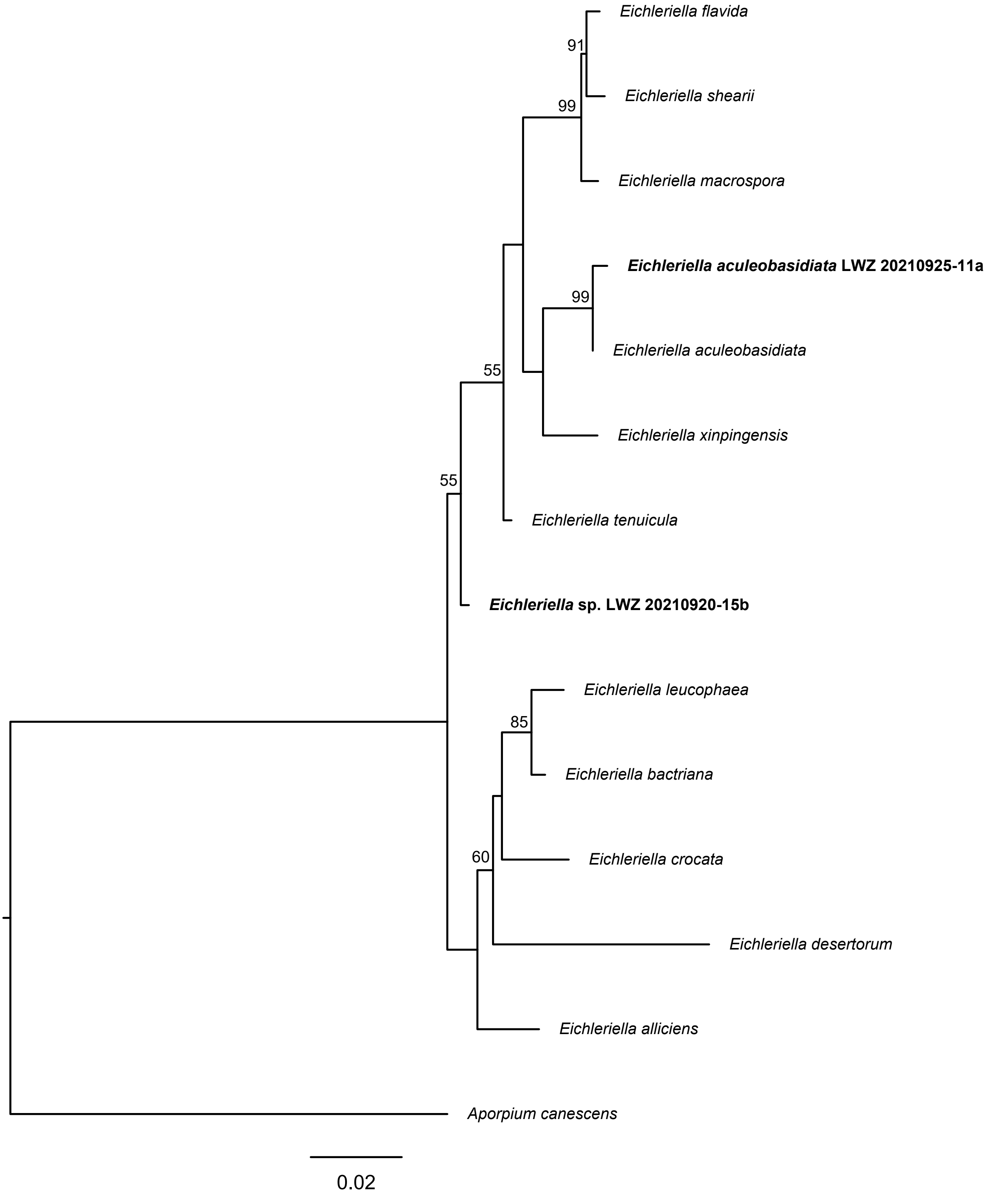
Eichleriella flavida
91
Eichleriella shearii
99
Eichleriella macrospora
Eichleriella aculeobasidiata LWZ 20210925-11a
99
Eichleriella aculeobasidiata
55
Eichleriella xinpingensis
55
Eichleriella tenuicula
Eichleriella sp. LWZ 20210920-15b
Eichleriella leucophaea
85
Eichleriella bactriana
Eichleriella crocata
60
Eichleriella desertorum
Eichleriella alliciens
Aporpium canescens
0.02

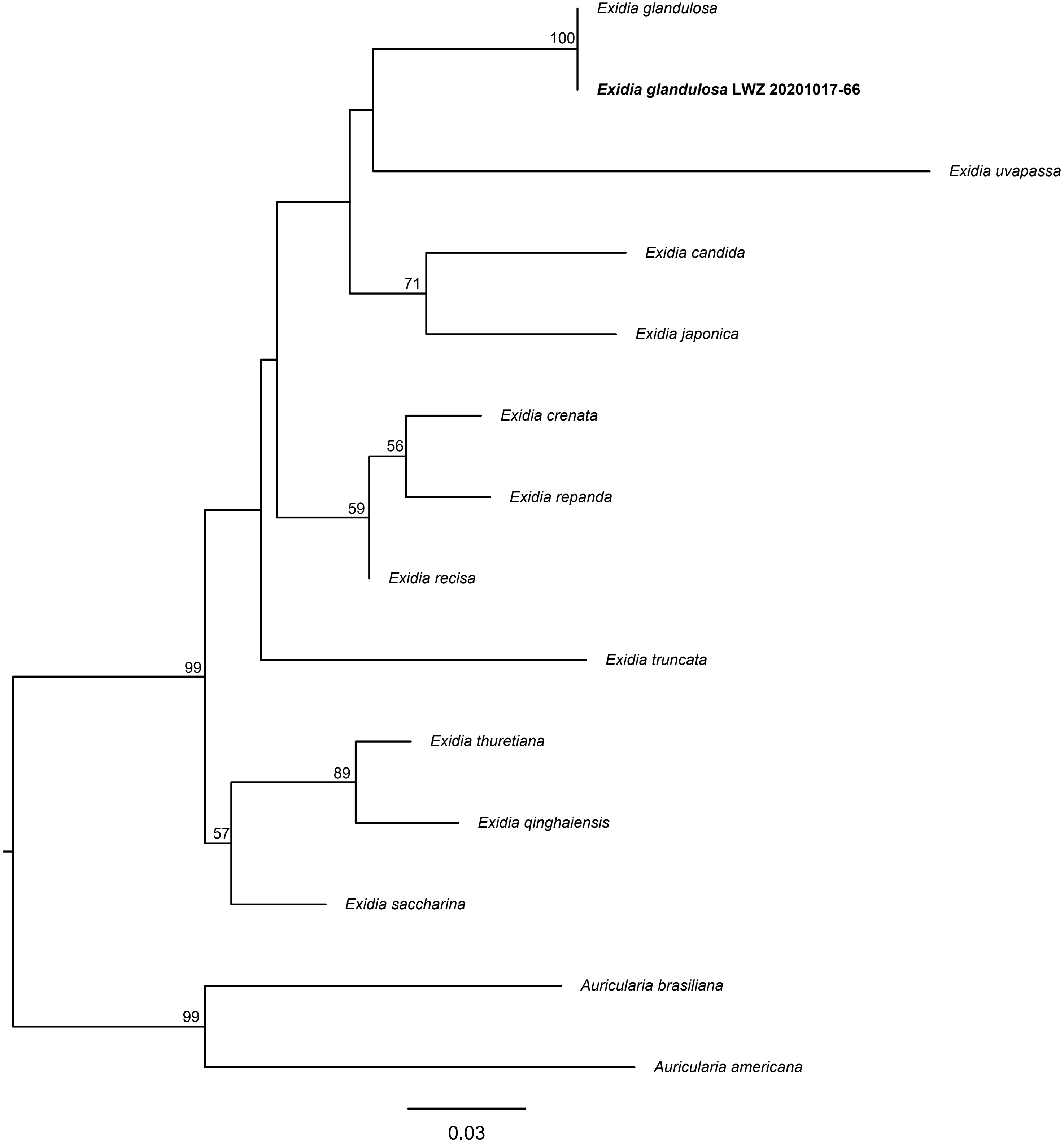
Exidia glandulosa
100
Exidia glandulosa LWZ 20201017-66
Exidia uvapassa
Exidia candida
71
Exidia japonica
Exidia crenata
56
Exidia repanda
59
Exidia recisa
Exidia truncata
99
Exidia thuretiana
89
Exidia qinghaiensis
57
Exidia saccharina
Auricularia brasiliana
99
Auricularia americana
0.03

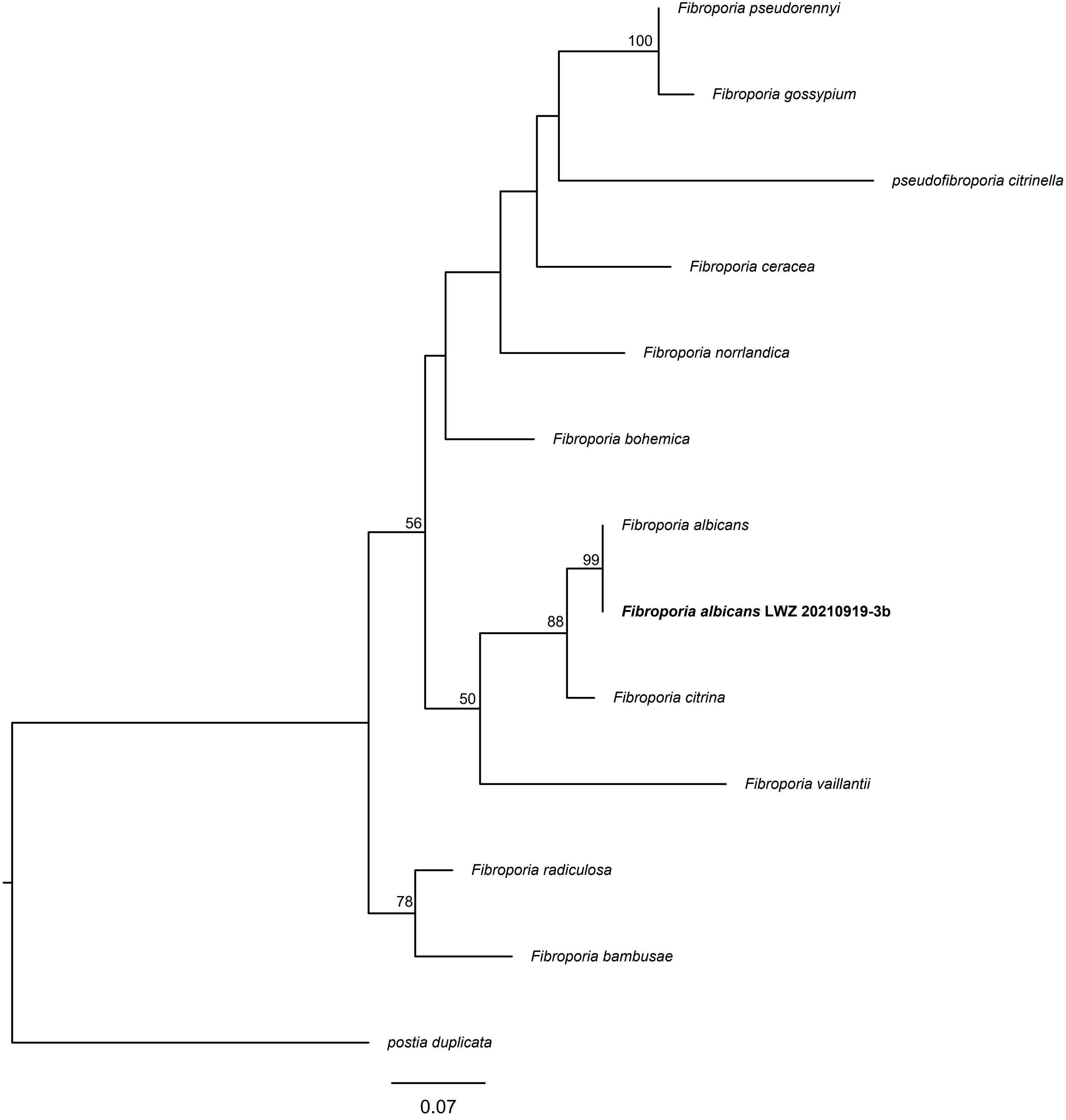
Fibroporia pseudorennyi
100
Fibroporia gossypium
pseudofibroporia citrinella
Fibroporia ceracea
Fibroporia norrlandica
Fibroporia bohemica
56
Fibroporia albicans
99
Fibroporia albicans LWZ 20210919-3b
88
Fibroporia citrina
50
Fibroporia vaillantii
Fibroporia radiculosa
78
Fibroporia bambusae
postia duplicata
0.07

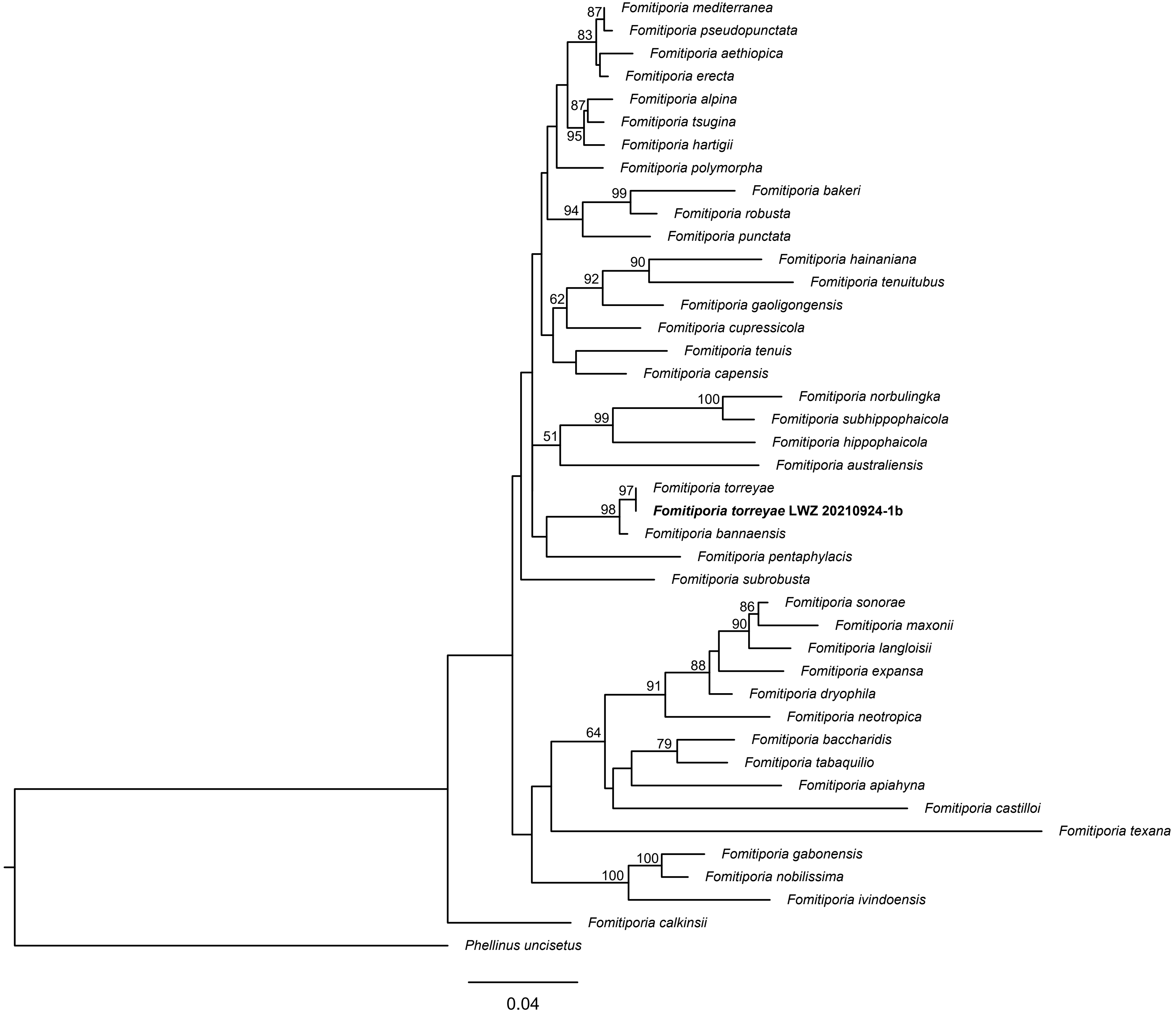
Fomitiporia mediterranea
Fomitiporia pseudopunctata
Fomitiporia aethiopica
Fomitiporia erecta
Fomitiporia alpina
Fomitiporia tsugina
Fomitiporia hartigii
Fomitiporia polymorpha
Fomitiporia bakeri
Fomitiporia robusta
Fomitiporia punctata
Fomitiporia hainaniana
Fomitiporia tenuitubus
Fomitiporia gaoligongensis
Fomitiporia cupressicola
Fomitiporia tenuis
Fomitiporia capensis
Fomitiporia norbulingka
Fomitiporia subhippophaicola
Fomitiporia hippophaicola
Fomitiporia australiensis
Fomitiporia torreyae
Fomitiporia torreyae LWZ 20210924-1b
Fomitiporia bannaensis
Fomitiporia pentaphylacis
Fomitiporia subrobusta
Fomitiporia sonorae
Fomitiporia maxonii
Fomitiporia langloisii
Fomitiporia expansa
Fomitiporia dryophila
Fomitiporia neotropica
Fomitiporia baccharidis
Fomitiporia tabaquilio
Fomitiporia apiahyna
Fomitiporia castilloi
Fomitiporia texana
Fomitiporia gabonensis
Fomitiporia nobilissima
Fomitiporia ivindoensis
Fomitiporia calkinsii
Phellinus uncisetus
87
83
87
95
99
94
90
92
62
100
99
51
97
98
86
90
88
91
64
79
100
100
0.04

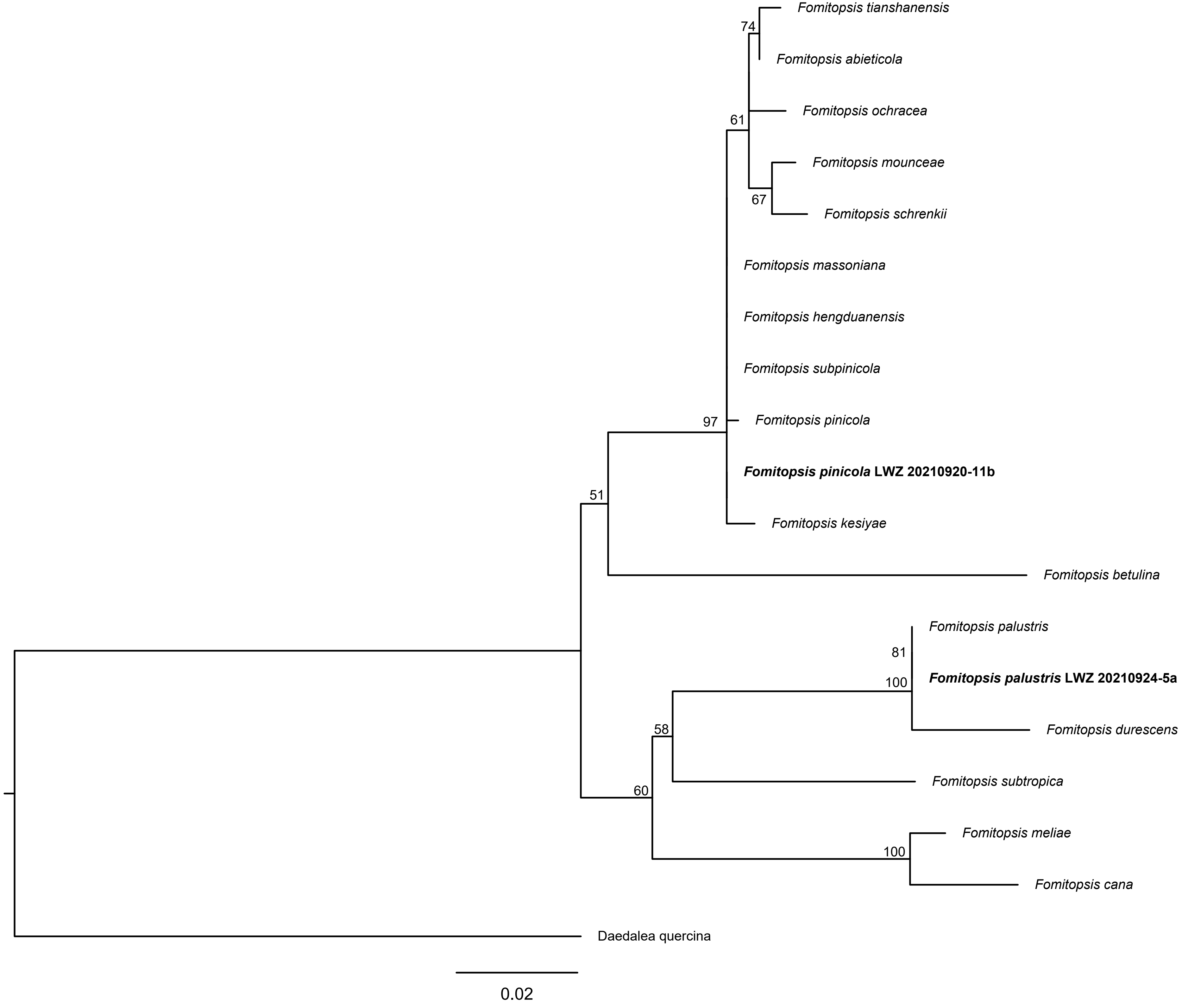
Fomitopsis tianshanensis
74
Fomitopsis abieticola
Fomitopsis ochracea
61
Fomitopsis mounceae
67
Fomitopsis schrenkii
Fomitopsis massoniana
Fomitopsis hengduanensis
Fomitopsis subpinicola
97
Fomitopsis pinicola
Fomitopsis pinicola LWZ 20210920-11b
51
Fomitopsis kesiyae
Fomitopsis betulina
Fomitopsis palustris
81
100
Fomitopsis palustris LWZ 20210924-5a
Fomitopsis durescens
58
60
Fomitopsis subtropica
Fomitopsis meliae
100
Fomitopsis cana
Daedalea quercina
0.02

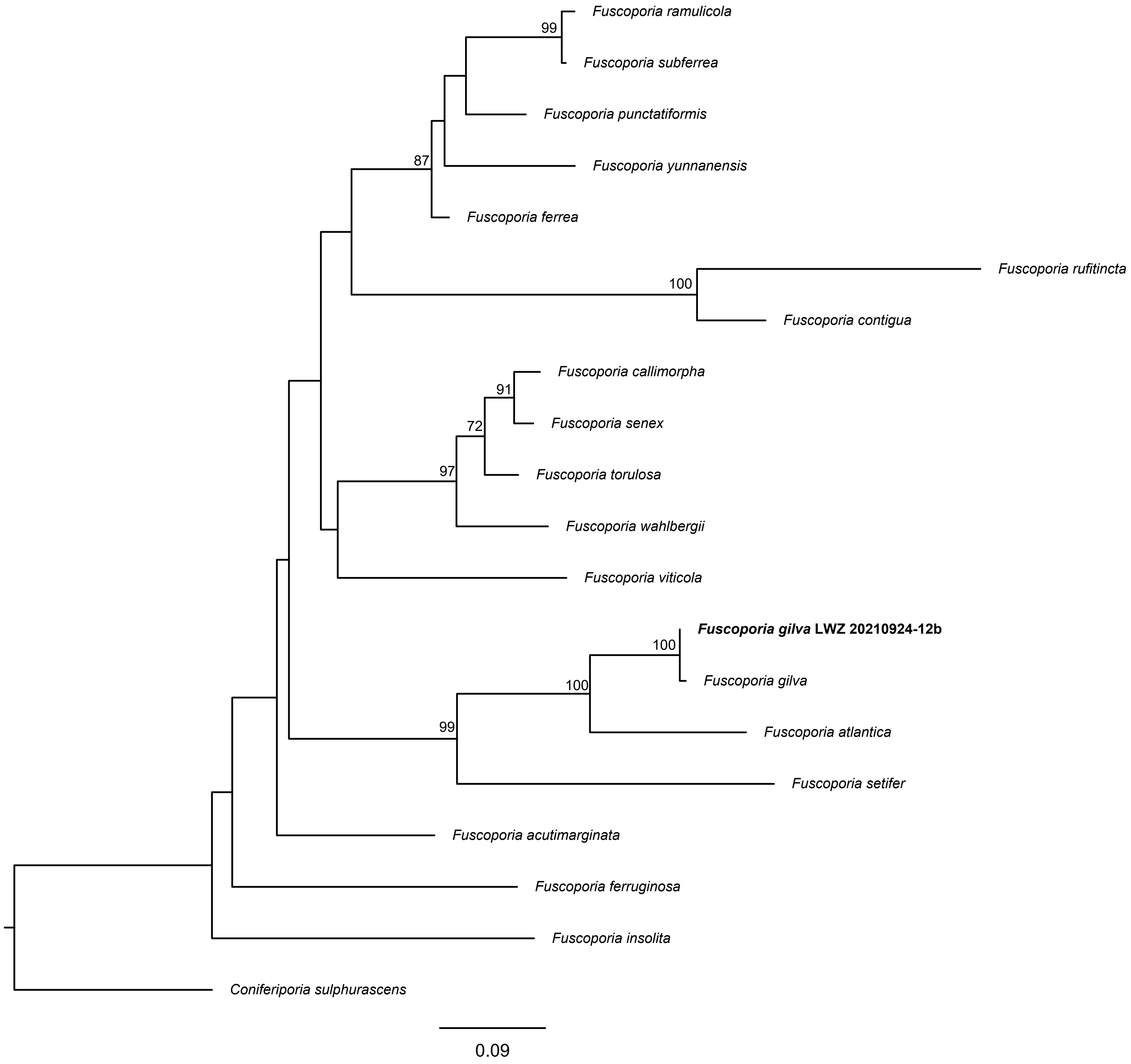
Fuscoporia ramulicola
99
Fuscoporia subferrea
Fuscoporia punctatiformis
87
Fuscoporia yunnanensis
Fuscoporia ferrea
Fuscoporia rufitincta
100
Fuscoporia contigua
Fuscoporia callimorpha
91
Fuscoporia senex
72
Fuscoporia torulosa
97
Fuscoporia wahlbergii
Fuscoporia viticola
Fuscoporia gilva LWZ 20210924-12b
100
Fuscoporia gilva
100
Fuscoporia atlantica
99
Fuscoporia setifer
Fuscoporia acutimarginata
Fuscoporia ferruginosa
Fuscoporia insolita
Coniferiporia sulphurascens
0.09

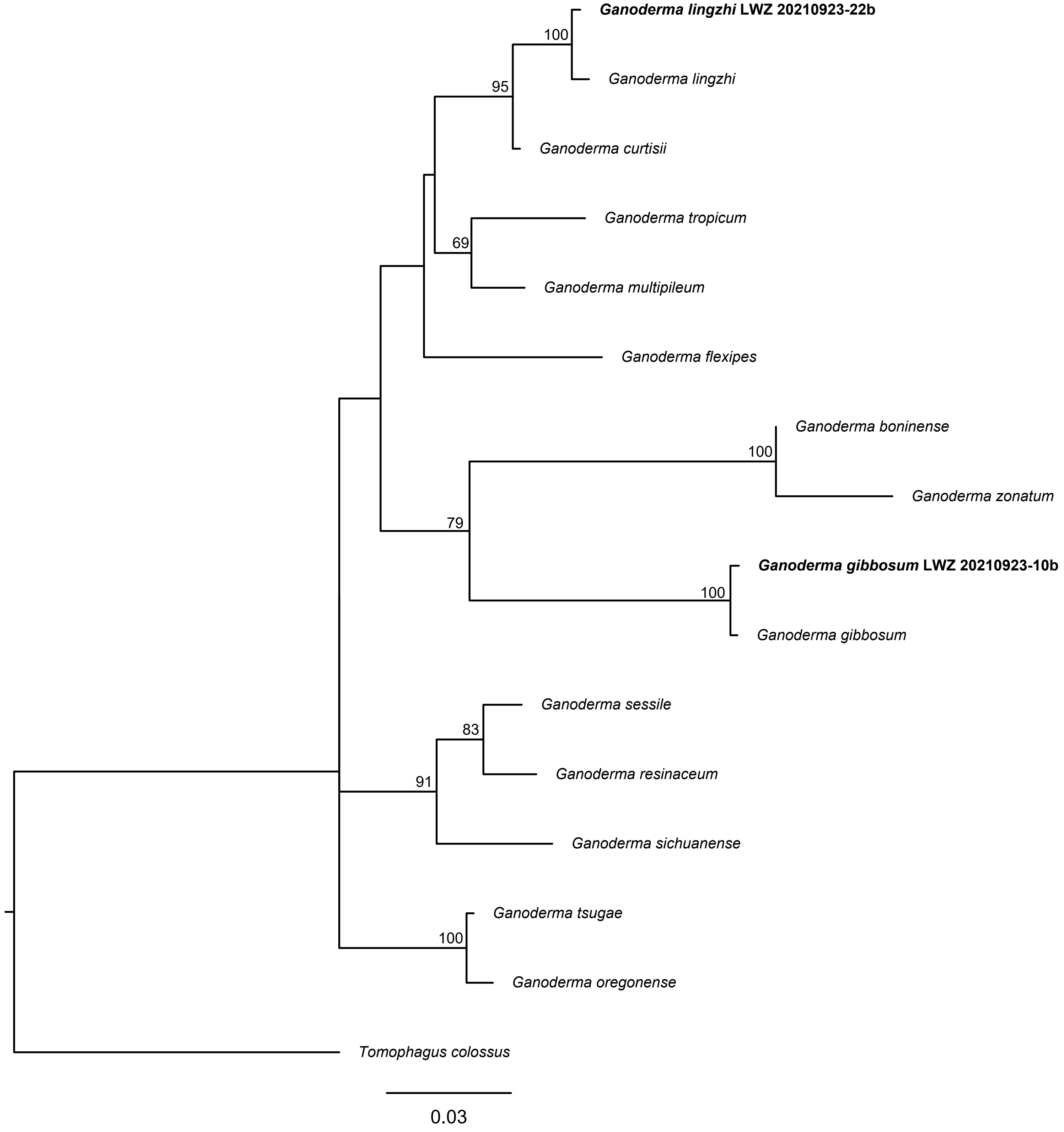

Ganoderma lingzhi LWZ 20210923-22b
100
Ganoderma lingzhi
95
Ganoderma curtisii
Ganoderma tropicum
69
Ganoderma multipileum
Ganoderma flexipes
Ganoderma boninense
100
Ganoderma zonatum
79
Ganoderma gibbosum LWZ 20210923-10b
100
Ganoderma gibbosum
Ganoderma sessile
83
Ganoderma resinaceum
91
Ganoderma sichuanense
Ganoderma tsugae
100
Ganoderma oregonense
Tomophagus colossus
0.03

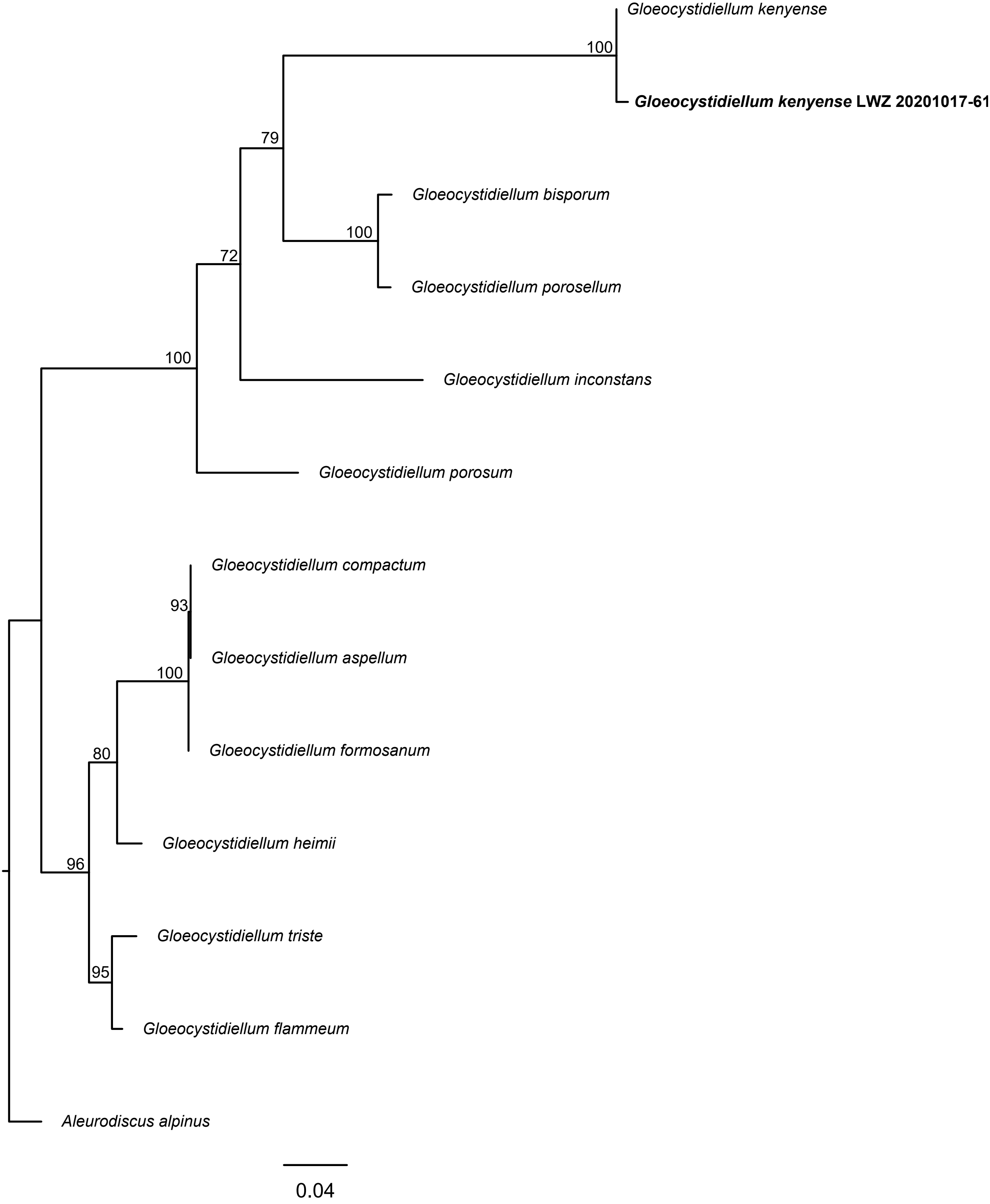
Gloeocystidiellum kenyense
100
Gloeocystidiellum kenyense LWZ 20201017-61
79
Gloeocystidiellum bisporum
100
Gloeocystidiellum porosellum
72
100
Gloeocystidiellum inconstans
Gloeocystidiellum porosum
Gloeocystidiellum compactum
93
Gloeocystidiellum aspellum
100
Gloeocystidiellum formosanum
80
Gloeocystidiellum heimii
96
Gloeocystidiellum triste
95
Gloeocystidiellum flammeum
Aleurodiscus alpinus
0.04

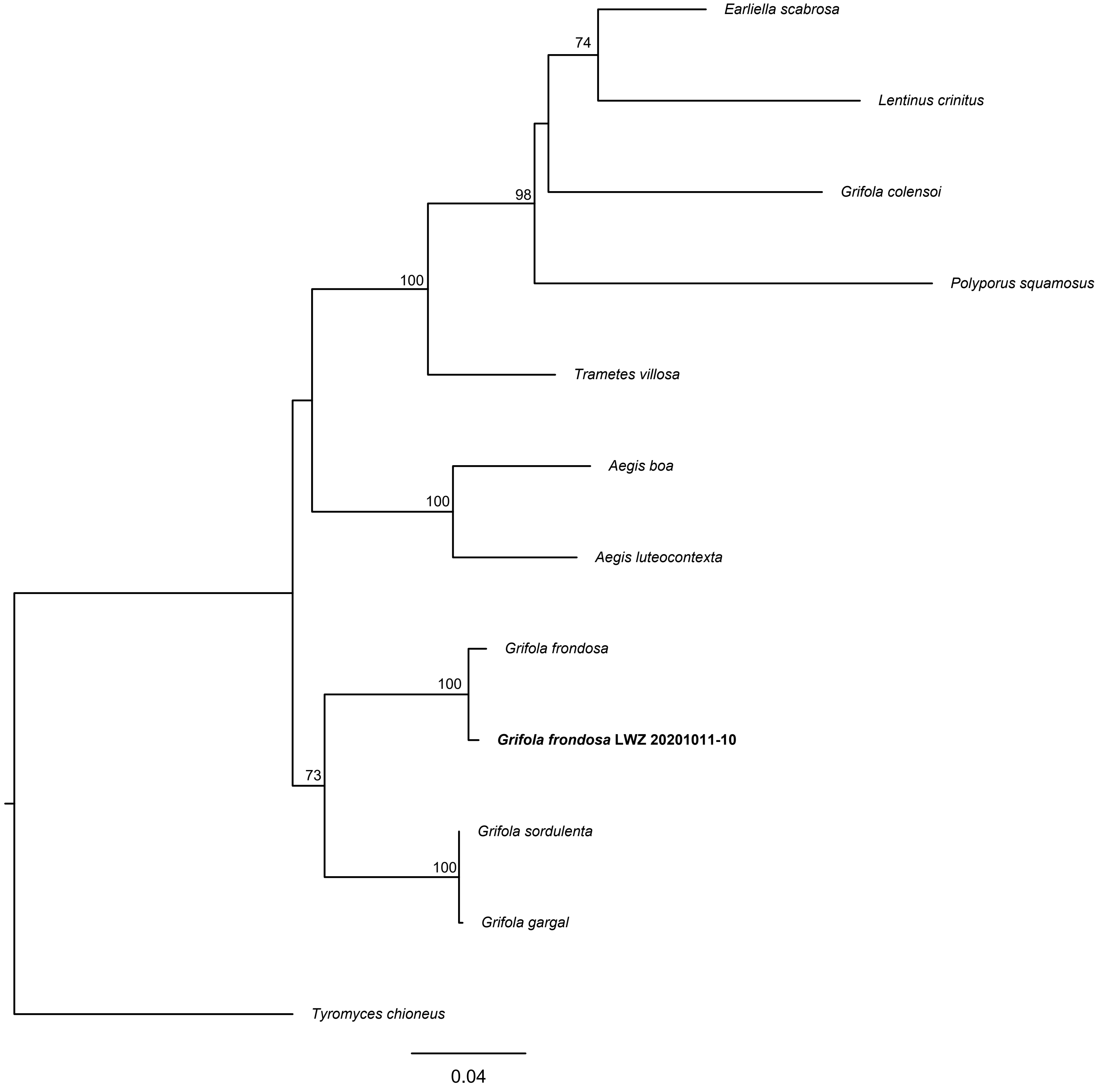

Earliella scabrosa
74
Lentinus crinitus
Grifola colensoi
98
Polyporus squamosus
100
Trametes villosa
Aegis boa
100
Aegis luteocontexta
Grifola frondosa
100
Grifola frondosa LWZ 20201011-10
73
Grifola sordulenta
100
Grifola gargal
Tyromyces chioneus
0.04

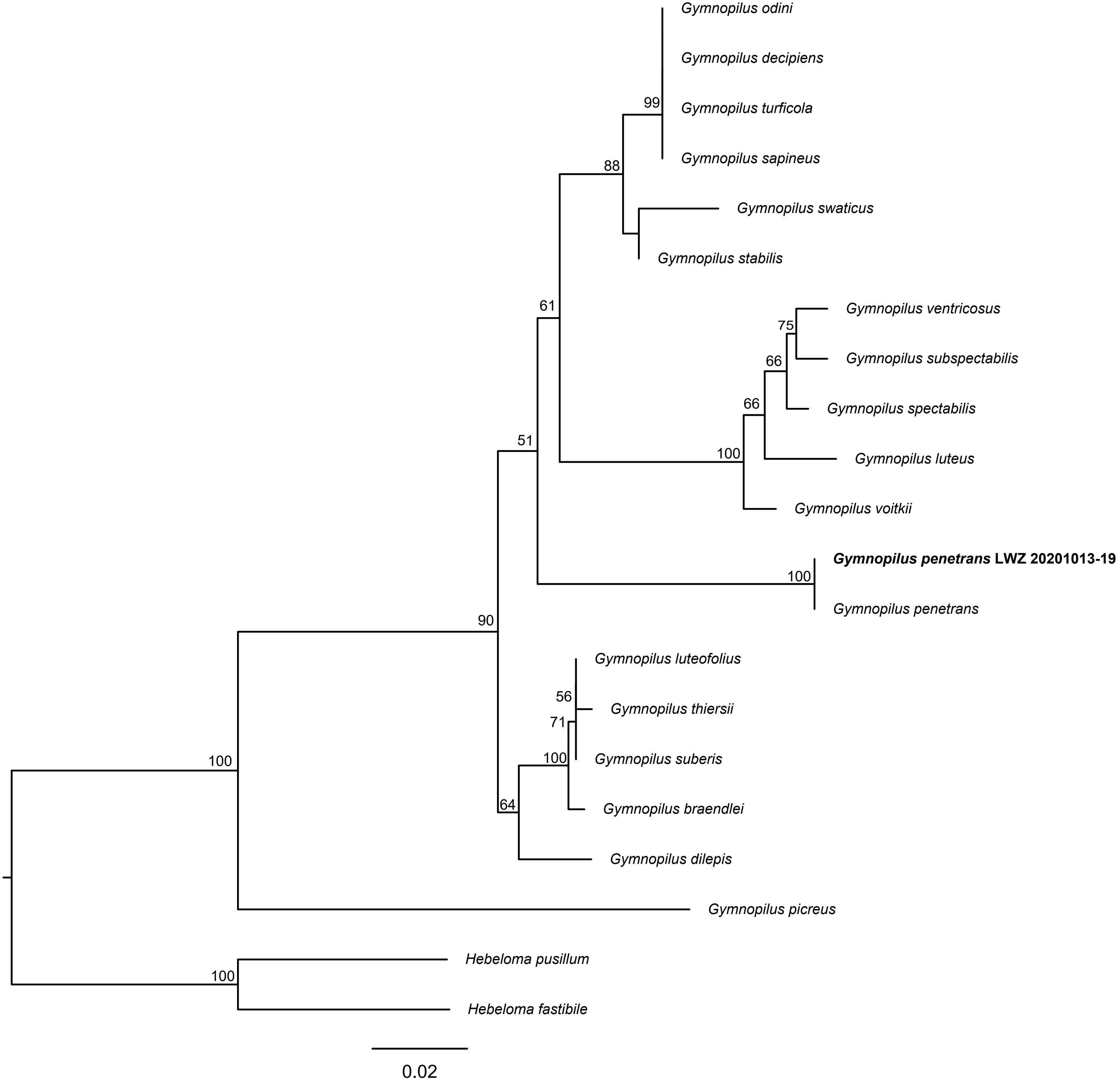

Gymnopilus odini
Gymnopilus decipiens
Gymnopilus turficola
Gymnopilus sapineus
Gymnopilus swaticus
Gymnopilus stabilis
Gymnopilus ventricosus
Gymnopilus subspectabilis
Gymnopilus spectabilis
Gymnopilus luteus
Gymnopilus voitkii
Gymnopilus penetrans LWZ 20201013-19
Gymnopilus penetrans
Gymnopilus luteofolius
Gymnopilus thiersii
Gymnopilus suberis
Gymnopilus braendlei
Gymnopilus dilepis
Gymnopilus picreus
Hebeloma pusillum
Hebeloma fastibile
99
88
61
75
66
66
100
51
100
90
56
71
100
64
100
100
0.02

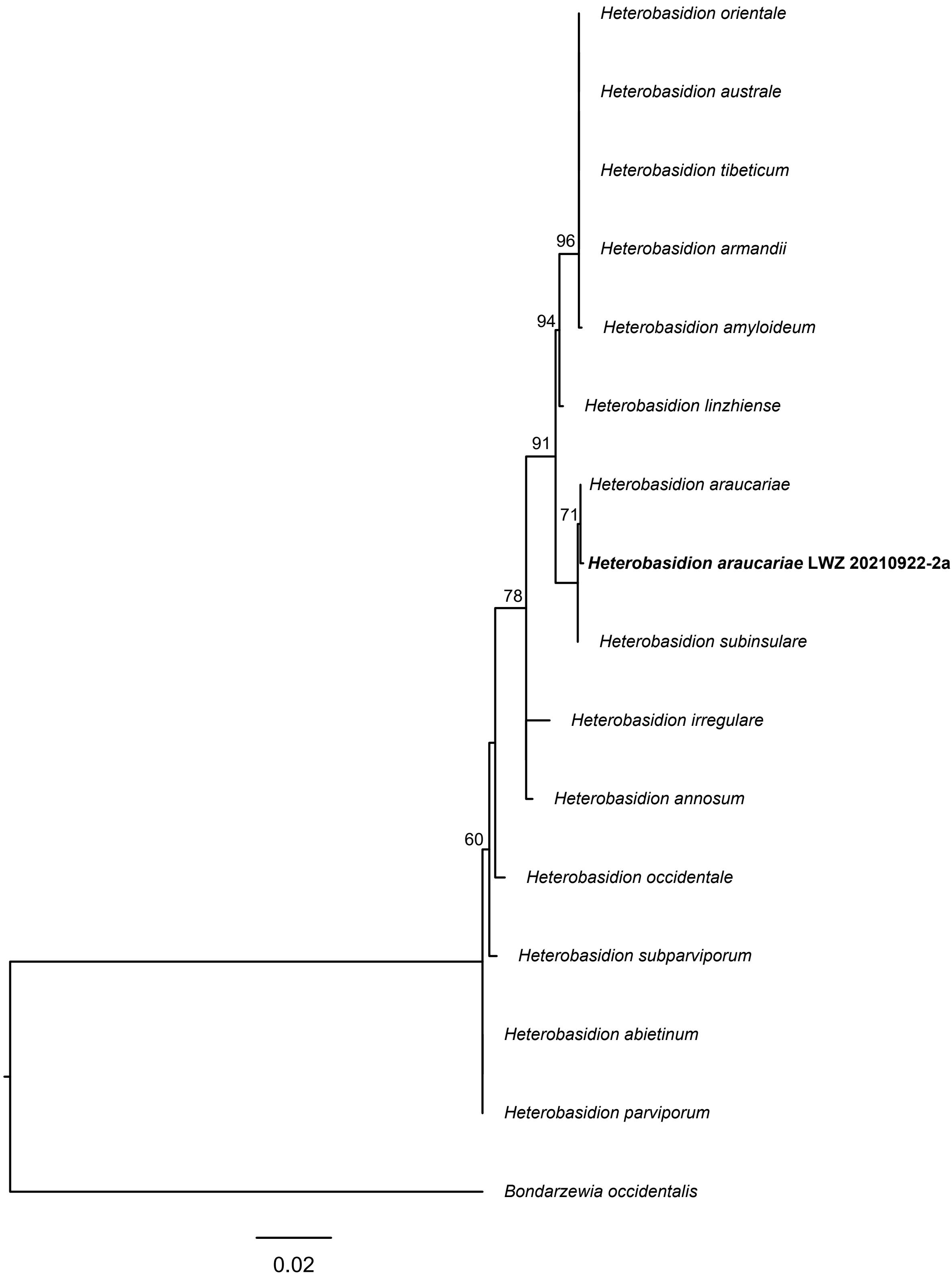
Heterobasidion orientale
Heterobasidion australe
Heterobasidion tibeticum
Heterobasidion armandii
Heterobasidion amyloideum
Heterobasidion linzhiense
Heterobasidion araucariae
Heterobasidion araucariae LWZ 20210922-2a
Heterobasidion subinsulare
Heterobasidion irregulare
Heterobasidion annosum
Heterobasidion occidentale
Heterobasidion subparviporum
Heterobasidion abietinum
Heterobasidion parviporum
Bondarzewia occidentalis
96
94
91
71
78
60
0.02

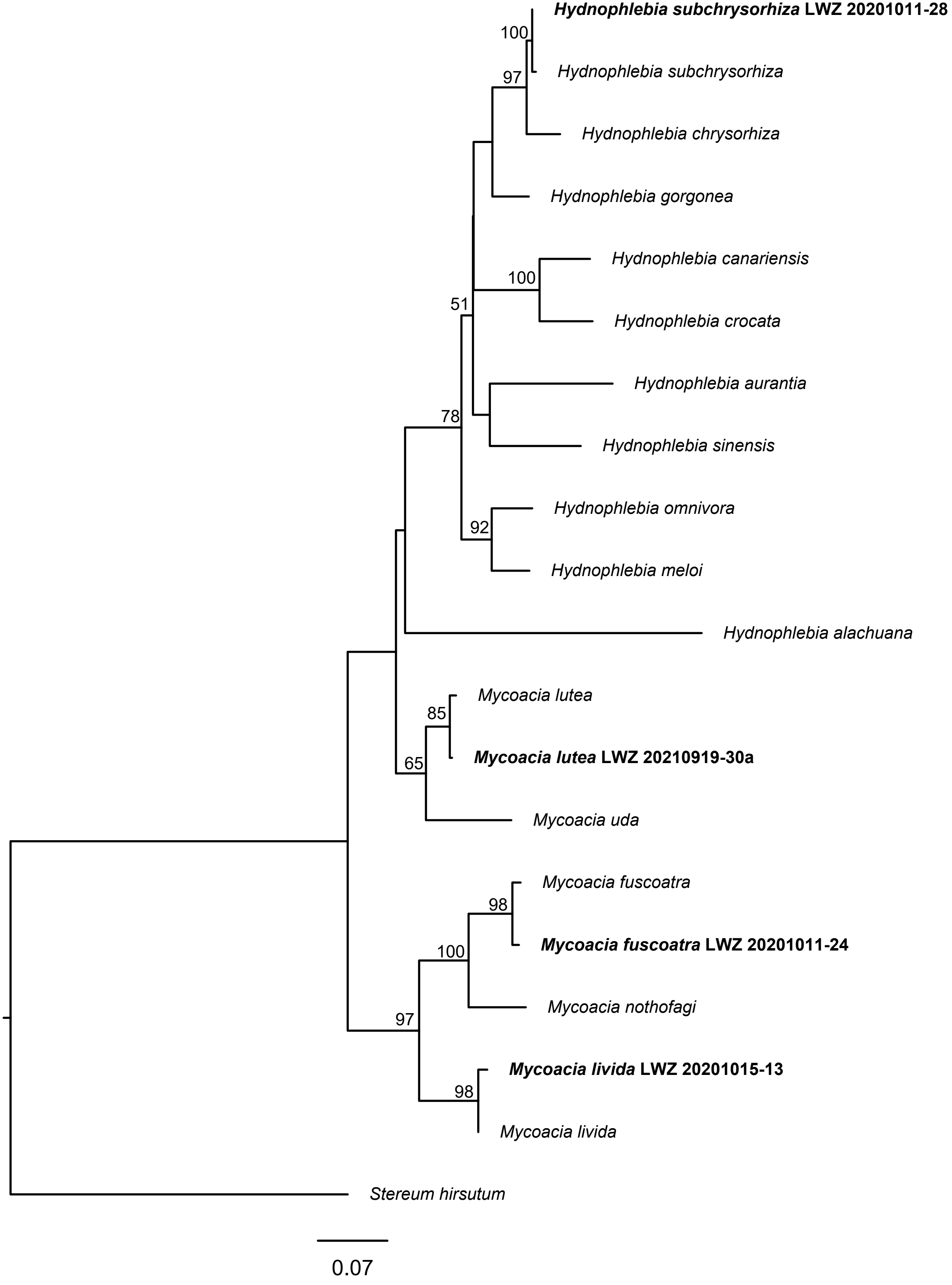

Hydnophlebia subchrysorhiza LWZ 20201011-28
Hydnophlebia subchrysorhiza
Hydnophlebia chrysorhiza
Hydnophlebia gorgonea
Hydnophlebia canariensis
Hydnophlebia crocata
Hydnophlebia aurantia
Hydnophlebia sinensis
Hydnophlebia omnivora
Hydnophlebia meloi
Hydnophlebia alachuana
Mycoacia lutea
Mycoacia lutea LWZ 20210919-30a
Mycoacia uda
Mycoacia fuscoatra
Mycoacia fuscoatra LWZ 20201011-24
Mycoacia nothofagi
Mycoacia livida LWZ 20201015-13
Mycoacia livida
Stereum hirsutum
100
97
100
51
78
92
85
65
98
100
97
98
0.07

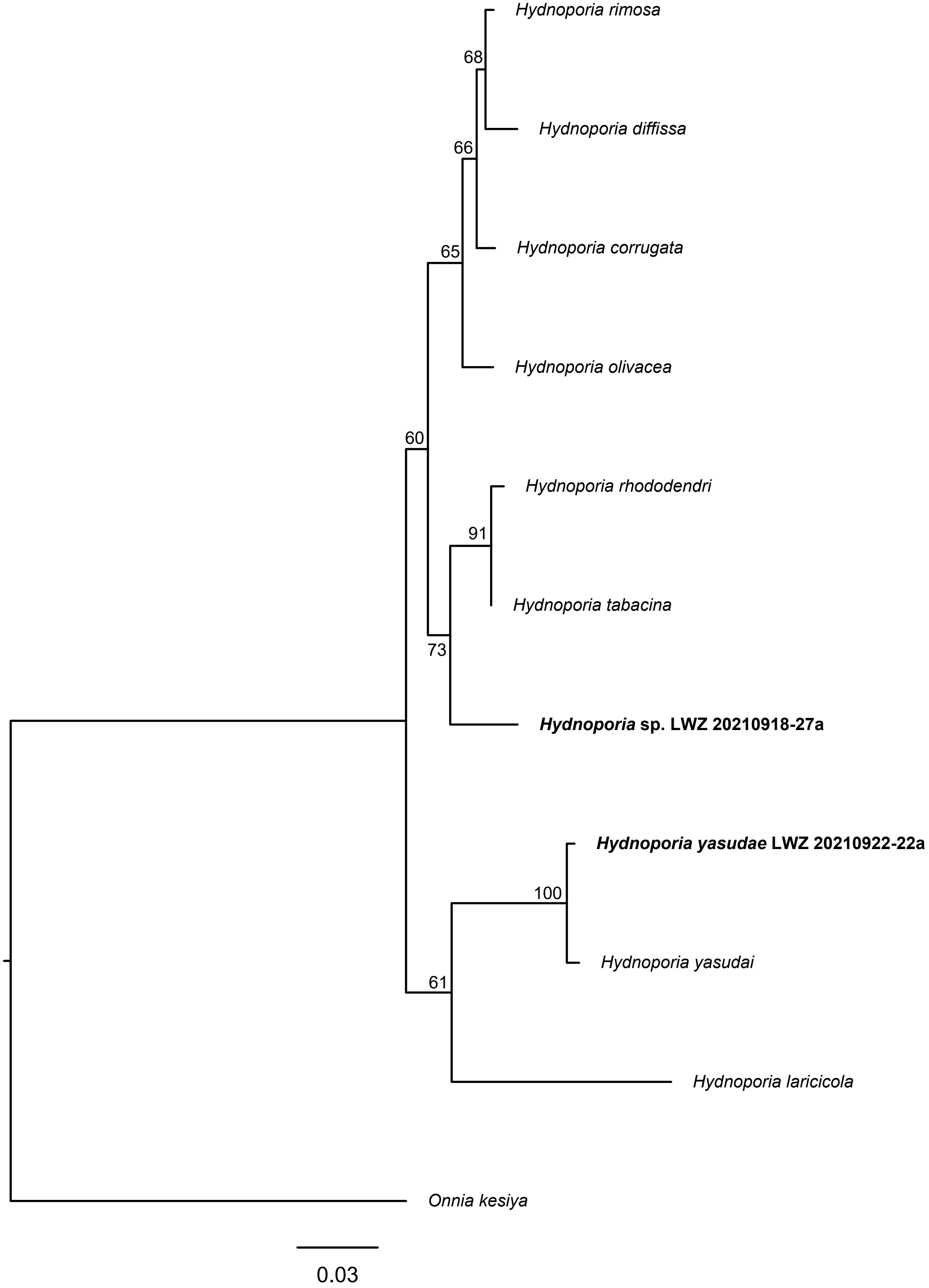
Hydnoporia rimosa
68
Hydnoporia diffissa
66
Hydnoporia corrugata
65
Hydnoporia olivacea
60
Hydnoporia rhododendri
91
Hydnoporia tabacina
73
Hydnoporia **sp. LWZ 20210918-27a**
Hydnoporia yasudae **LWZ 20210922-22a**
100
Hydnoporia yasudai
61
Hydnoporia laricicola
Onnia kesiya
0.03

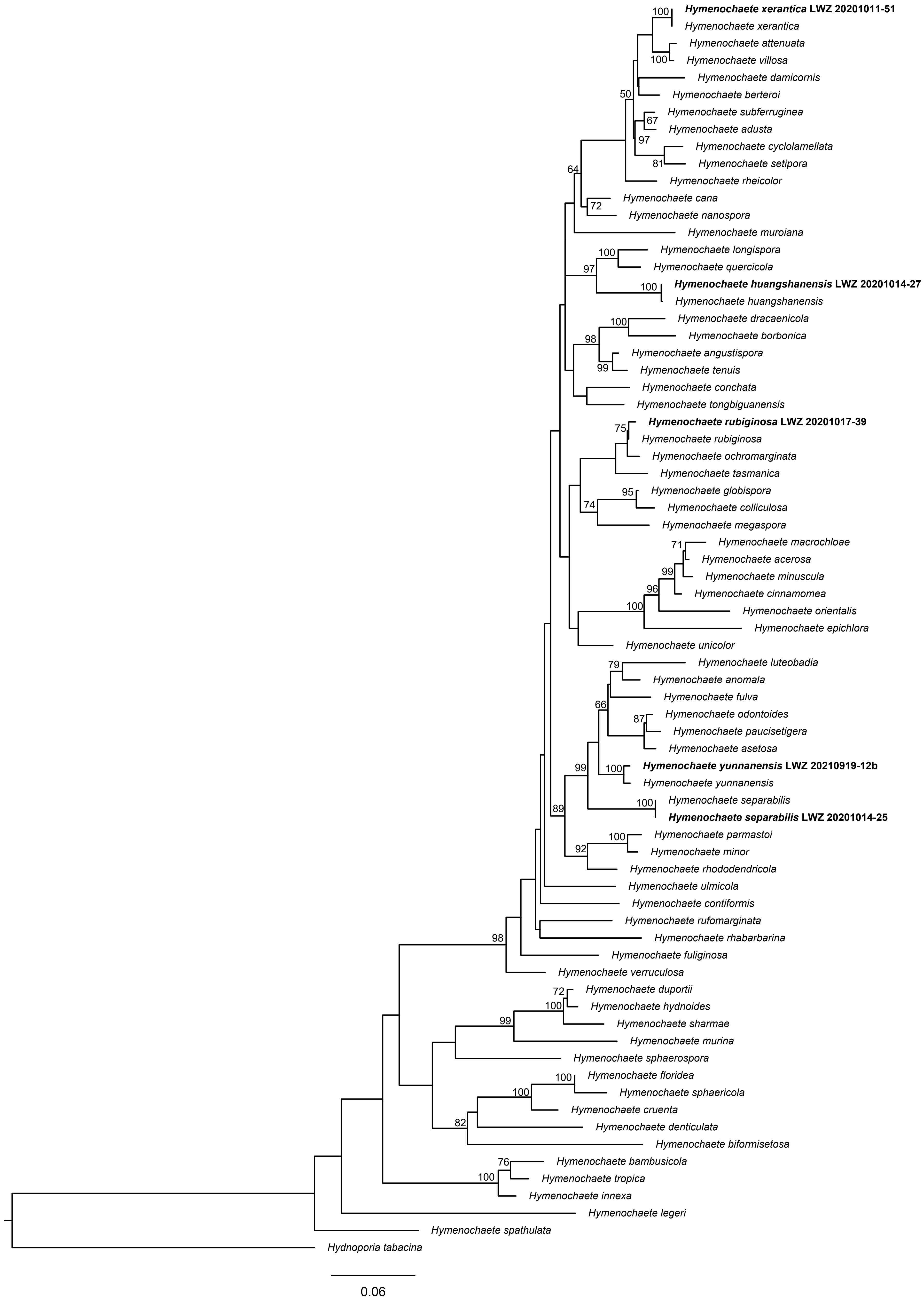

Hymenochaete xerantica LWZ 20201011-51
Hymenochaete xerantica
Hymenochaete attenuata
Hymenochaete villosa
Hymenochaete damicornis
Hymenochaete berteroi
Hymenochaete subferruginea
Hymenochaete adusta
Hymenochaete cyclolamellata
Hymenochaete setipora
Hymenochaete rheicolor
Hymenochaete cana
Hymenochaete nanospora
Hymenochaete muroiana
Hymenochaete longispora
Hymenochaete quercicola
Hymenochaete huangshanensis LWZ 20201014-27
Hymenochaete huangshanensis
Hymenochaete dracaenicola
Hymenochaete borbonica
Hymenochaete angustispora
Hymenochaete tenuis
Hymenochaete conchata
Hymenochaete tongbiguanensis
Hymenochaete rubiginosa LWZ 20201017-39
Hymenochaete rubiginosa
Hymenochaete ochromarginata
Hymenochaete tasmanica
Hymenochaete globispora
Hymenochaete colliculosa
Hymenochaete megaspora
Hymenochaete macrochloae
Hymenochaete acerosa
Hymenochaete minuscula
Hymenochaete cinnamomea
Hymenochaete orientalis
Hymenochaete epichlora
Hymenochaete unicolor
Hymenochaete luteobadia
Hymenochaete anomala
Hymenochaete fulva
Hymenochaete odontoides
Hymenochaete paucisetigera
Hymenochaete asetosa
Hymenochaete yunnanensis LWZ 20210919-12b
Hymenochaete yunnanensis
Hymenochaete separabilis
Hymenochaete separabilis LWZ 20201014-25
Hymenochaete parmastoi
Hymenochaete minor
Hymenochaete rhododendricola
Hymenochaete ulmicola
Hymenochaete contiformis
Hymenochaete rufomarginata
Hymenochaete rhabarbarina
Hymenochaete fuliginosa
Hymenochaete verruculosa
Hymenochaete duportii
Hymenochaete hydnoides
Hymenochaete sharmae
Hymenochaete murina
Hymenochaete sphaerospora
Hymenochaete floridea
Hymenochaete sphaericola
Hymenochaete cruenta
Hymenochaete denticulata
Hymenochaete biformisetosa
Hymenochaete bambusicola
Hymenochaete tropica
Hymenochaete innexa
Hymenochaete legeri
Hymenochaete spathulata
Hydnoporia tabacina
0.06

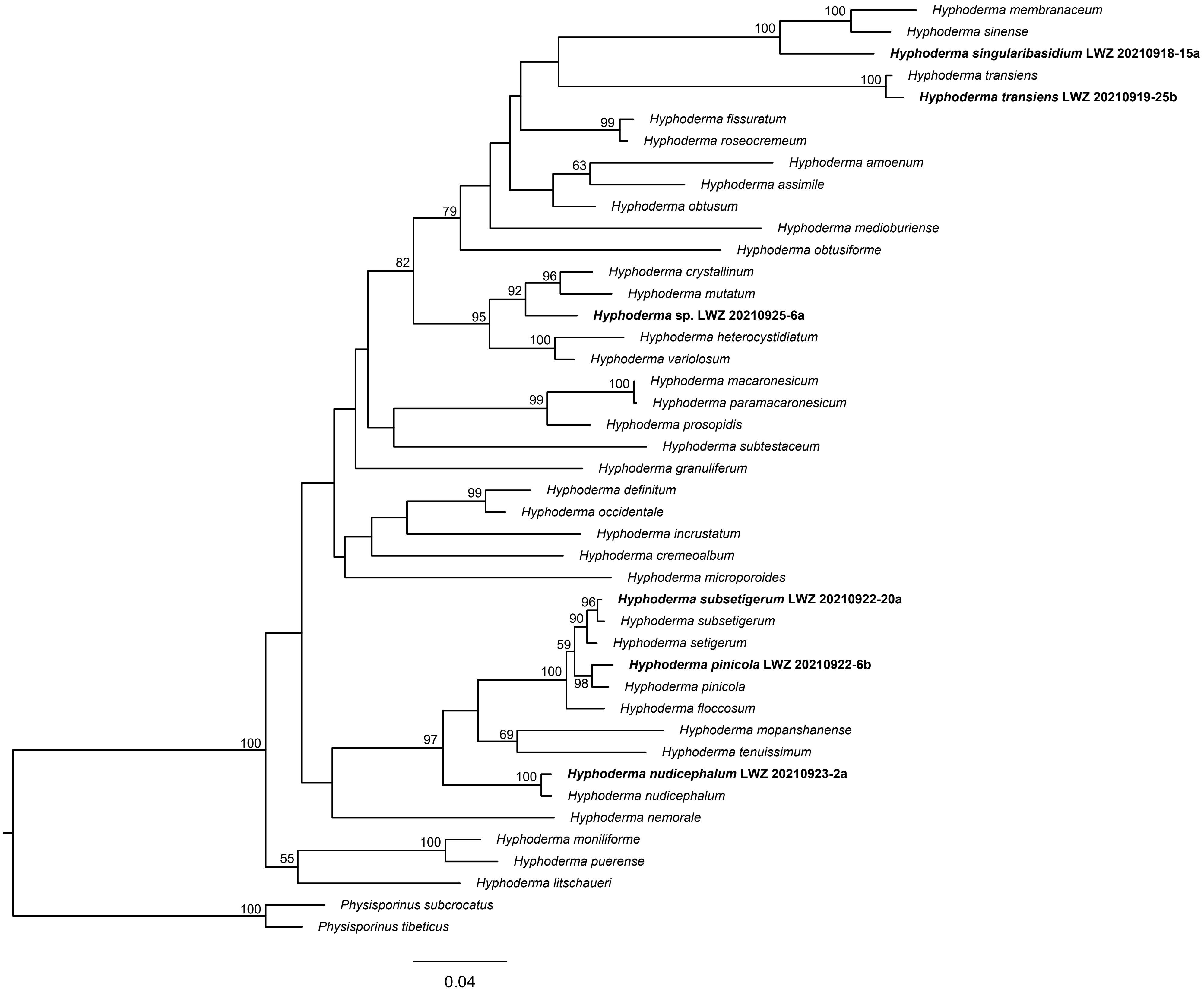

100
Hyphoderma membranaceum
100
Hyphoderma sinense
Hyphoderma singularibasidium LWZ 20210918-15a
100
Hyphoderma transiens
Hyphoderma transiens LWZ 20210919-25b
99
Hyphoderma fissuratum
Hyphoderma roseocremeum
63
Hyphoderma amoenum
Hyphoderma assimile
Hyphoderma obtusum
Hyphoderma medioburiense
79
Hyphoderma obtusiforme
82
96
Hyphoderma crystallinum
92
Hyphoderma mutatum
Hyphoderma sp. LWZ 20210925-6a
95
100
Hyphoderma heterocystidiatum
Hyphoderma variolosum
100
Hyphoderma macaronesicum
99
Hyphoderma paramacaronesicum
Hyphoderma prosopidis
Hyphoderma subtestaceum
Hyphoderma granuliferum
99
Hyphoderma definitum
Hyphoderma occidentale
Hyphoderma incrustatum
Hyphoderma cremeoalbum
Hyphoderma microporoides
96
Hyphoderma subsetigerum LWZ 20210922-20a
90
Hyphoderma subsetigerum
59
Hyphoderma setigerum
100
Hyphoderma pinicola LWZ 20210922-6b
98
Hyphoderma pinicola
Hyphoderma floccosum
69
Hyphoderma mopanshanense
100
97
Hyphoderma tenuissimum
100
Hyphoderma nudicephalum LWZ 20210923-2a
Hyphoderma nudicephalum
Hyphoderma nemorale
100
Hyphoderma moniliforme
55
Hyphoderma puerense
Hyphoderma litschaueri
100
Physisporinus subcrocatus
Physisporinus tibeticus
0.04

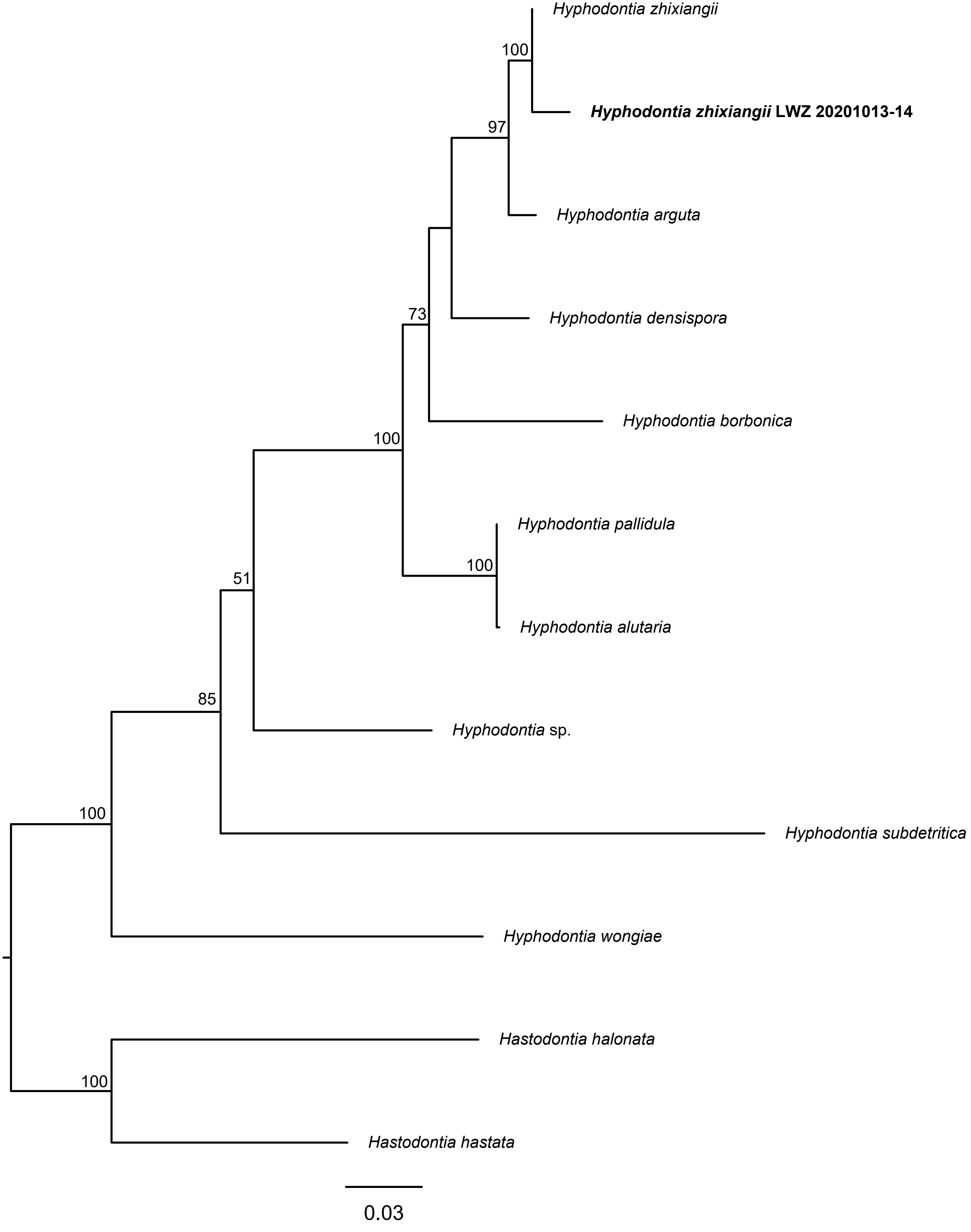
Hyphodontia zhixiangii
100
Hyphodontia zhixiangii LWZ 20201013-14
97
Hyphodontia arguta
Hyphodontia densispora
73
Hyphodontia borbonica
100
Hyphodontia pallidula
100
51
Hyphodontia alutaria
85
Hyphodontia sp.
100
Hyphodontia subdetritica
Hyphodontia wongiae
Hastodontia halonata
100
Hastodontia hastata
0.03

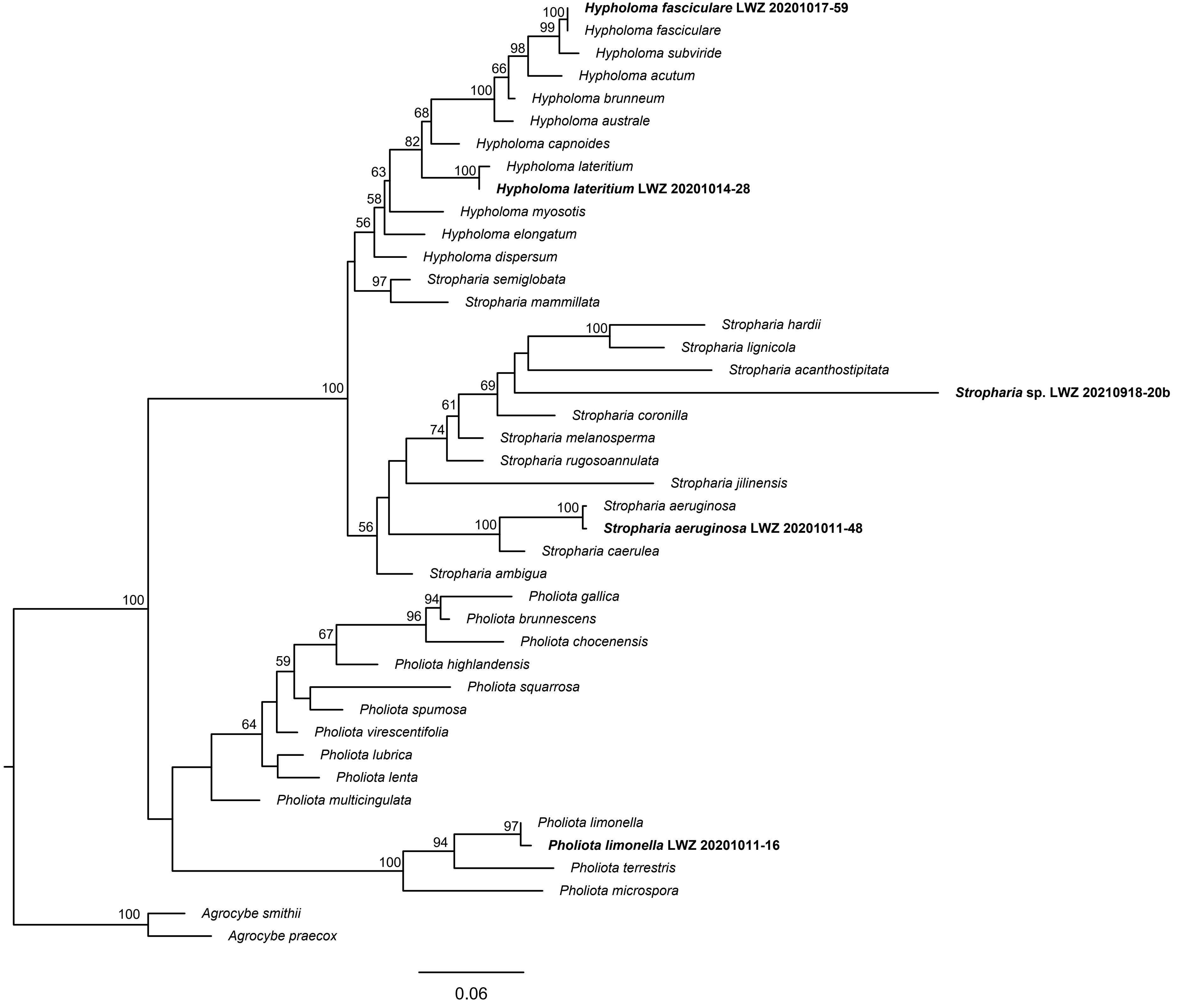

Hypholoma fasciculare LWZ 20201017-59
Hypholoma fasciculare
Hypholoma subviride
Hypholoma acutum
Hypholoma brunneum
Hypholoma australe
Hypholoma capnoides
Hypholoma lateritium
Hypholoma lateritium LWZ 20201014-28
Hypholoma myosotis
Hypholoma elongatum
Hypholoma dispersum
Stropharia semiglobata
Stropharia mammillata
Stropharia hardii
Stropharia lignicola
Stropharia acanthostipitata
Stropharia sp. LWZ 20210918-20b
Stropharia coronilla
Stropharia melanosperma
Stropharia rugosoannulata
Stropharia jilinensis
Stropharia aeruginosa
Stropharia aeruginosa LWZ 20201011-48
Stropharia caerulea
Stropharia ambigua
Pholiota gallica
Pholiota brunnescens
Pholiota chocenensis
Pholiota highlandensis
Pholiota squarrosa
Pholiota spumosa
Pholiota virescentifolia
Pholiota lubrica
Pholiota lenta
Pholiota multicingulata
Pholiota limonella
Pholiota limonella LWZ 20201011-16
Pholiota terrestris
Pholiota microspora
Agrocybe smithii
Agrocybe praecox
0.06

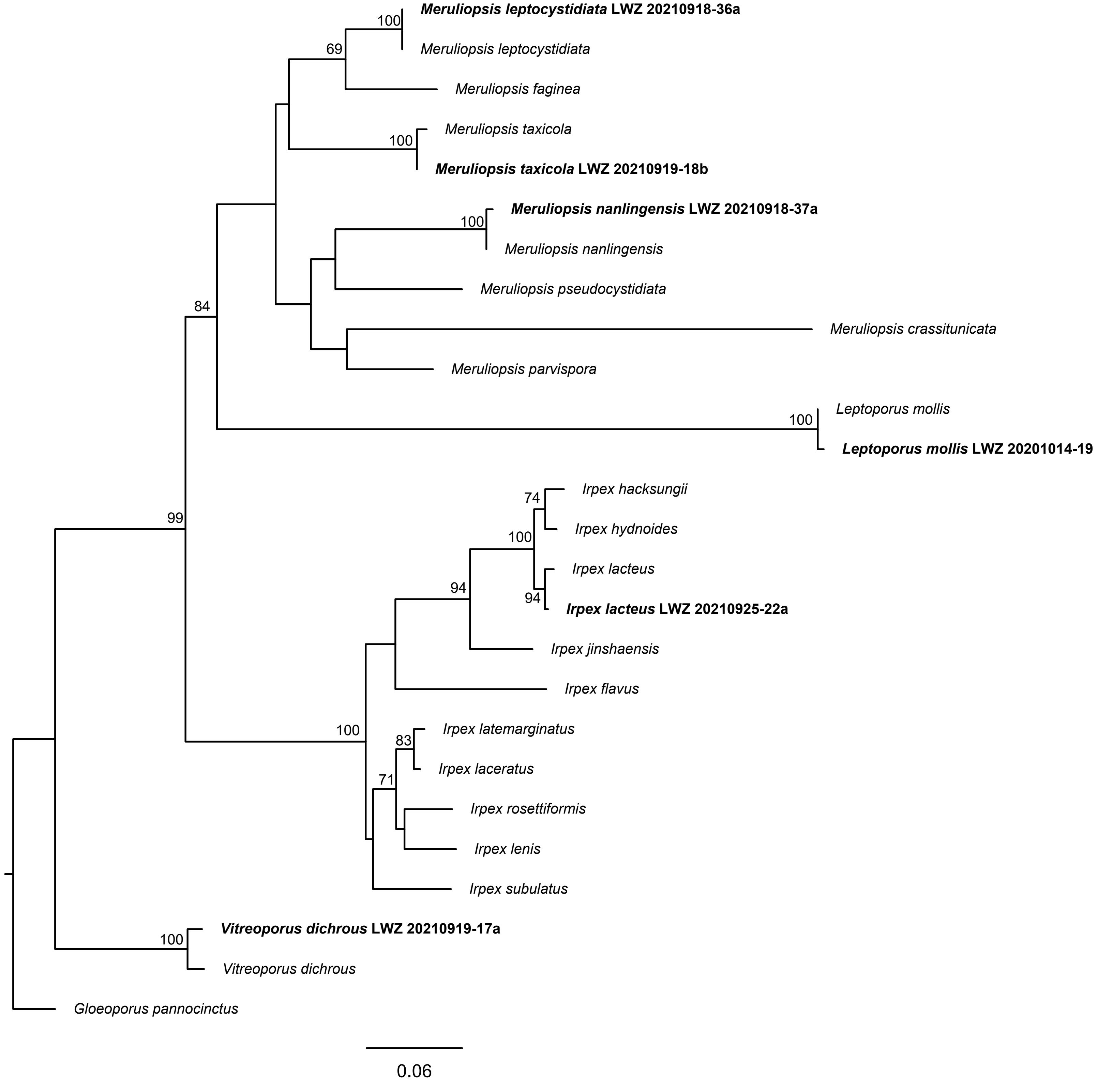
Meruliopsis leptocystidiata LWZ 20210918-36a
Meruliopsis leptocystidiata
Meruliopsis faginea
Meruliopsis taxicola
Meruliopsis taxicola LWZ 20210919-18b
Meruliopsis nanlingensis LWZ 20210918-37a
Meruliopsis nanlingensis
Meruliopsis pseudocystidiata
Meruliopsis crassitunicata
Meruliopsis parvispora
Leptoporus mollis
Leptoporus mollis LWZ 20201014-19
Irpex hacksungii
Irpex hydnoides
Irpex lacteus
Irpex lacteus LWZ 20210925-22a
Irpex jinshaensis
Irpex flavus
Irpex latemarginatus
Irpex laceratus
Irpex rosettiformis
Irpex lenis
Irpex subulatus
Vitreoporus dichrous LWZ 20210919-17a
Vitreoporus dichrous
Gloeoporus pannocinctus
100
69
100
100
84
100
74
100
94
94
99
100
83
71
100
0.06

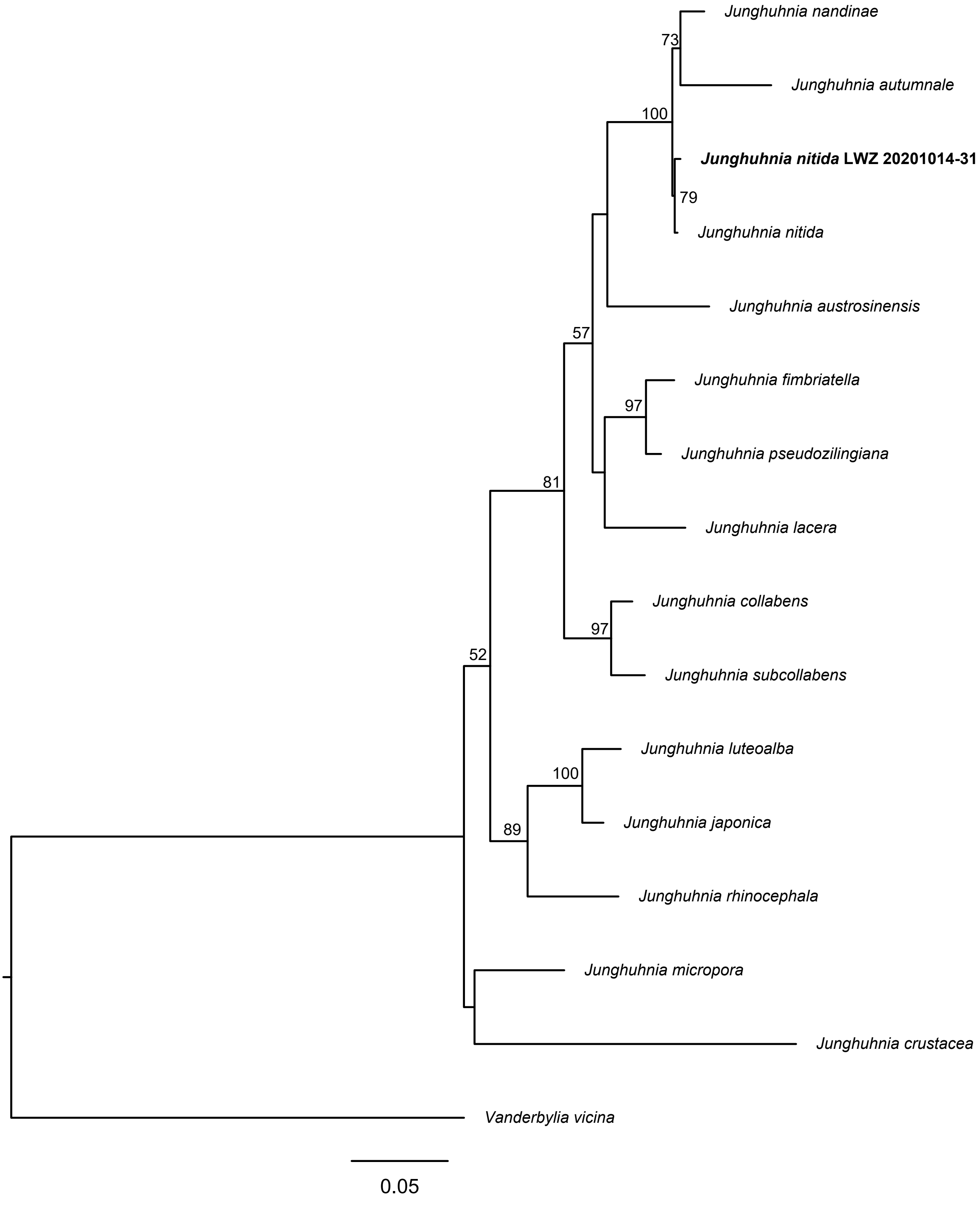
Junghuhnia nandinae
73
Junghuhnia autumnale
100
Junghuhnia nitida LWZ 20201014-31
79
Junghuhnia nitida
Junghuhnia austrosinensis
57
Junghuhnia fimbriatella
97
Junghuhnia pseudozilingiana
81
Junghuhnia lacera
Junghuhnia collabens
97
Junghuhnia subcollabens
52
Junghuhnia luteoalba
100
Junghuhnia japonica
89
Junghuhnia rhinocephala
Junghuhnia micropora
Junghuhnia crustacea
Vanderbylia vicina
0.05

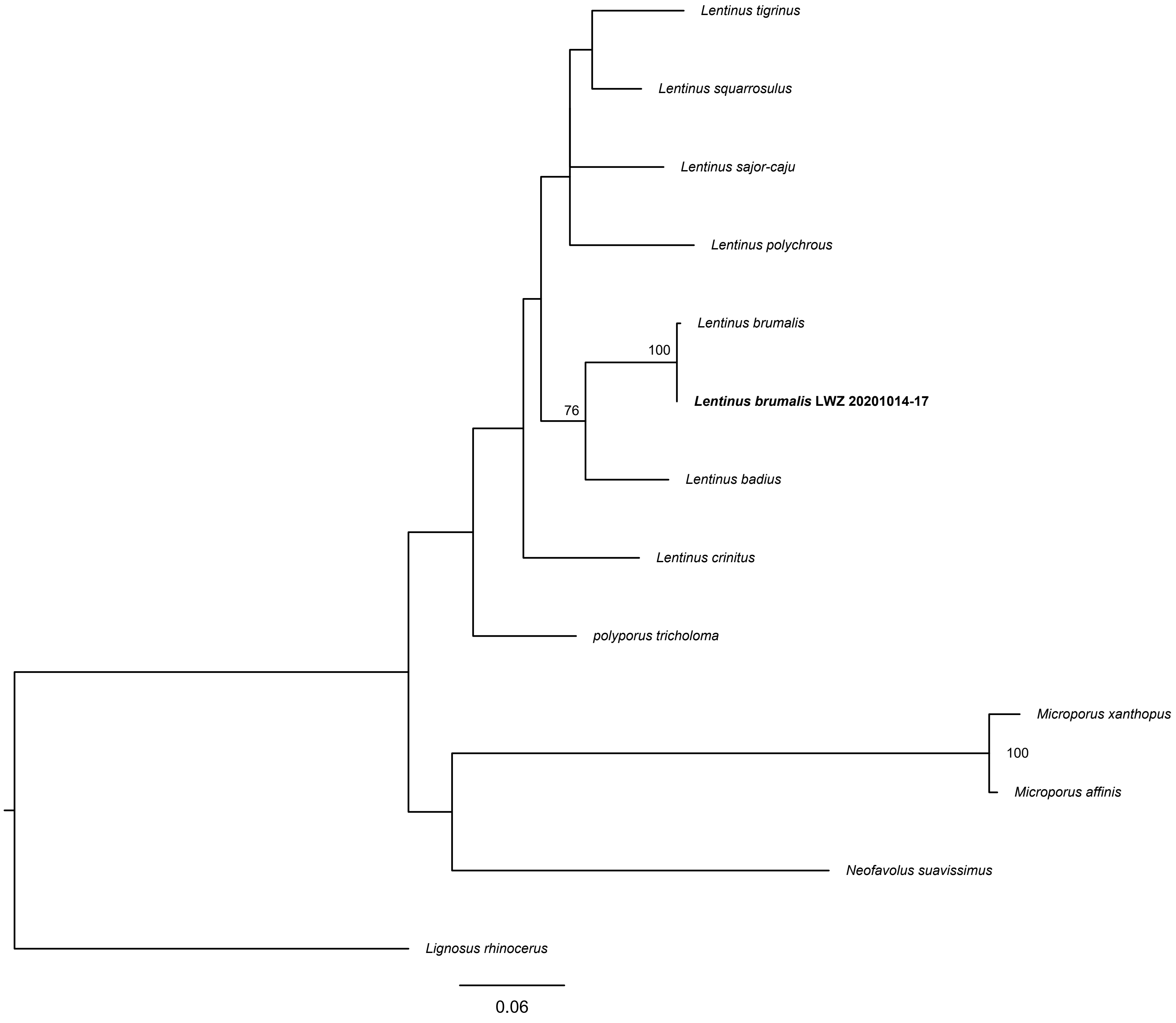

Lentinus tigrinus
Lentinus squarrosulus
Lentinus sajor-caju
Lentinus polychrous
Lentinus brumalis
100
Lentinus brumalis LWZ 20201014-17
76
Lentinus badius
Lentinus crinitus
polyporus tricholoma
Microporus xanthopus
100
Microporus affinis
Neofavolus suavissimus
Lignosus rhinocerus
0.06

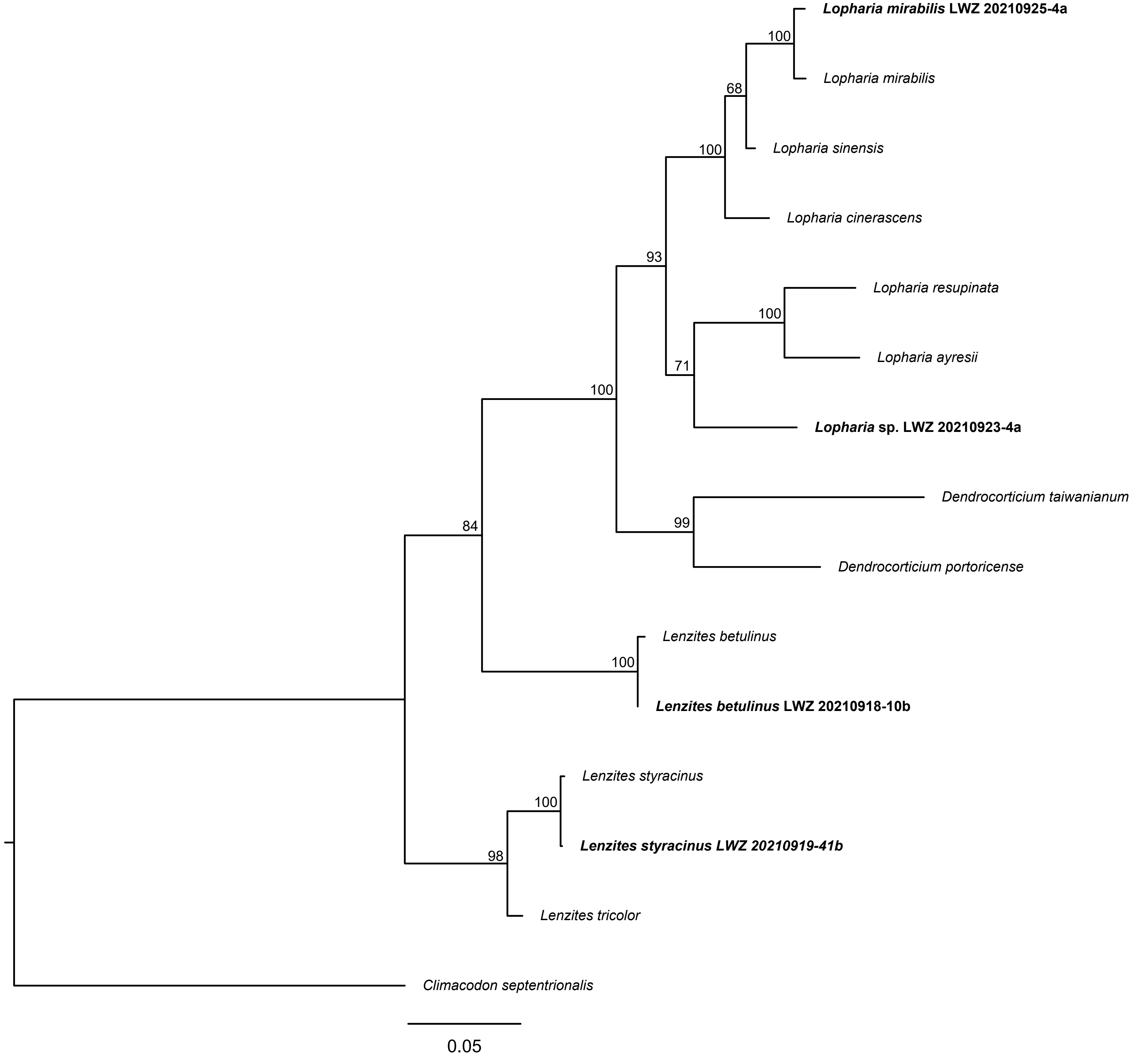

Lopharia mirabilis LWZ 20210925-4a
Lopharia mirabilis
Lopharia sinensis
Lopharia cinerascens
Lopharia resupinata
Lopharia ayresii
Lopharia sp. LWZ 20210923-4a
Dendrocorticium taiwanianum
Dendrocorticium portoricense
Lenzites betulinus
Lenzites betulinus LWZ 20210918-10b
Lenzites styracinus
Lenzites styracinus LWZ 20210919-41b
Lenzites tricolor
Climacodon septentrionalis
100
68
100
93
100
71
100
99
84
100
100
98
0.05

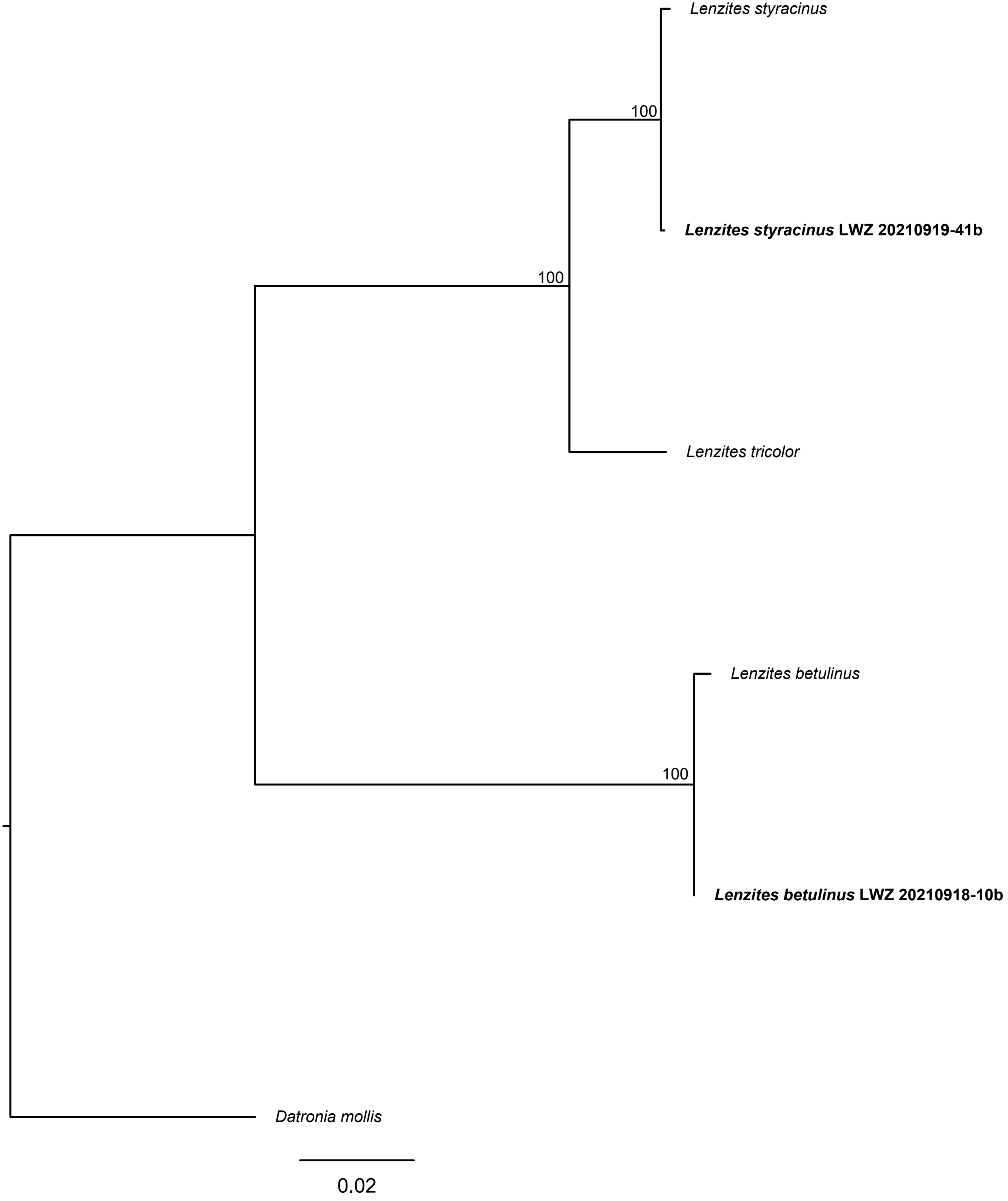

Lenzites styracinus
100
Lenzites styracinus LWZ 20210919-41b
100
Lenzites tricolor
Lenzites betulinus
100
Lenzites betulinus LWZ 20210918-10b
Datronia mollis
0.02

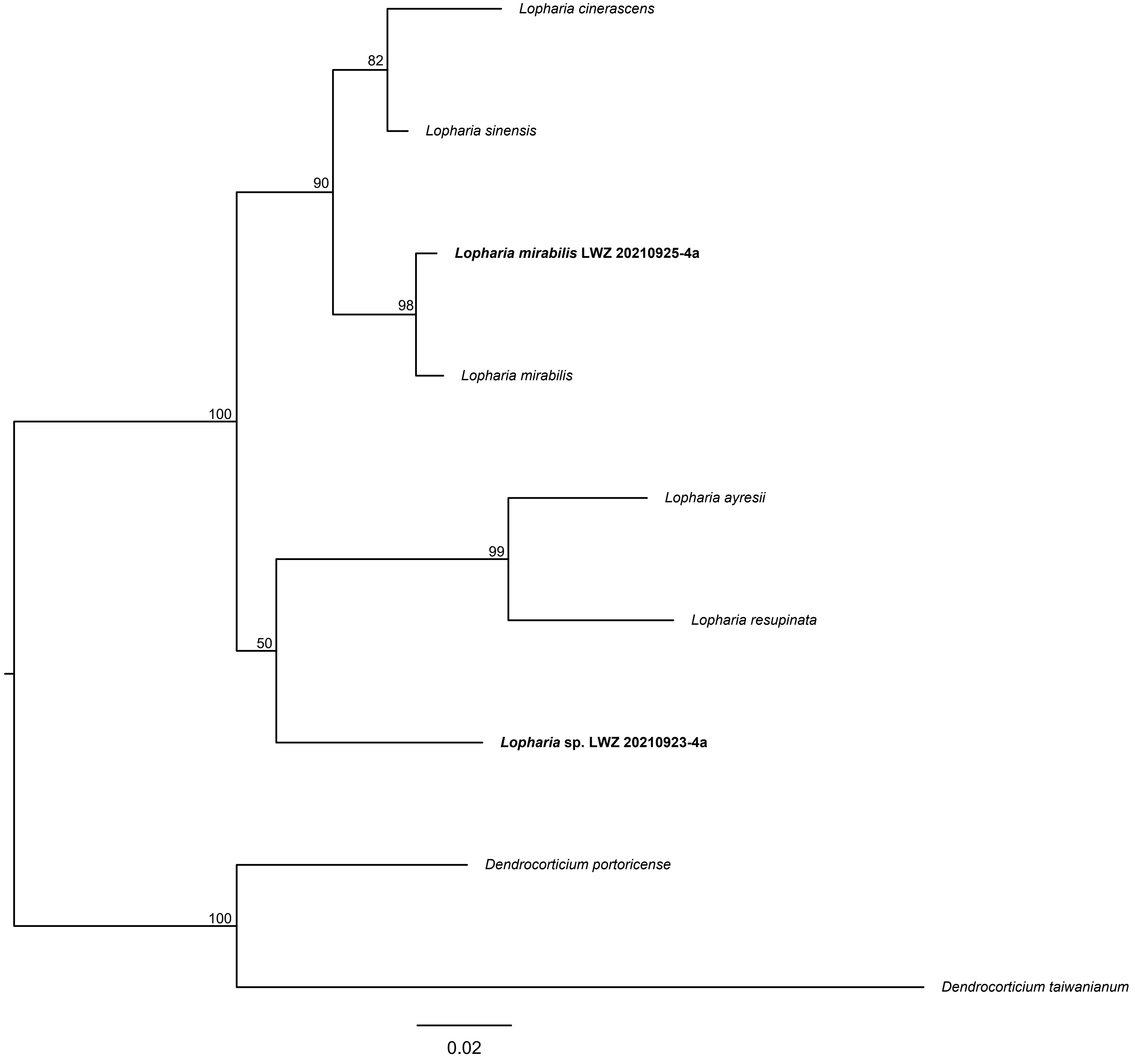

Lopharia cinerascens
82
Lopharia sinensis
90
Lopharia mirabilis LWZ 20210925-4a
98
Lopharia mirabilis
100
Lopharia ayresii
99
Lopharia resupinata
50
Lopharia sp. LWZ 20210923-4a
Dendrocorticium portoricense
100
Dendrocorticium taiwanianum
0.02

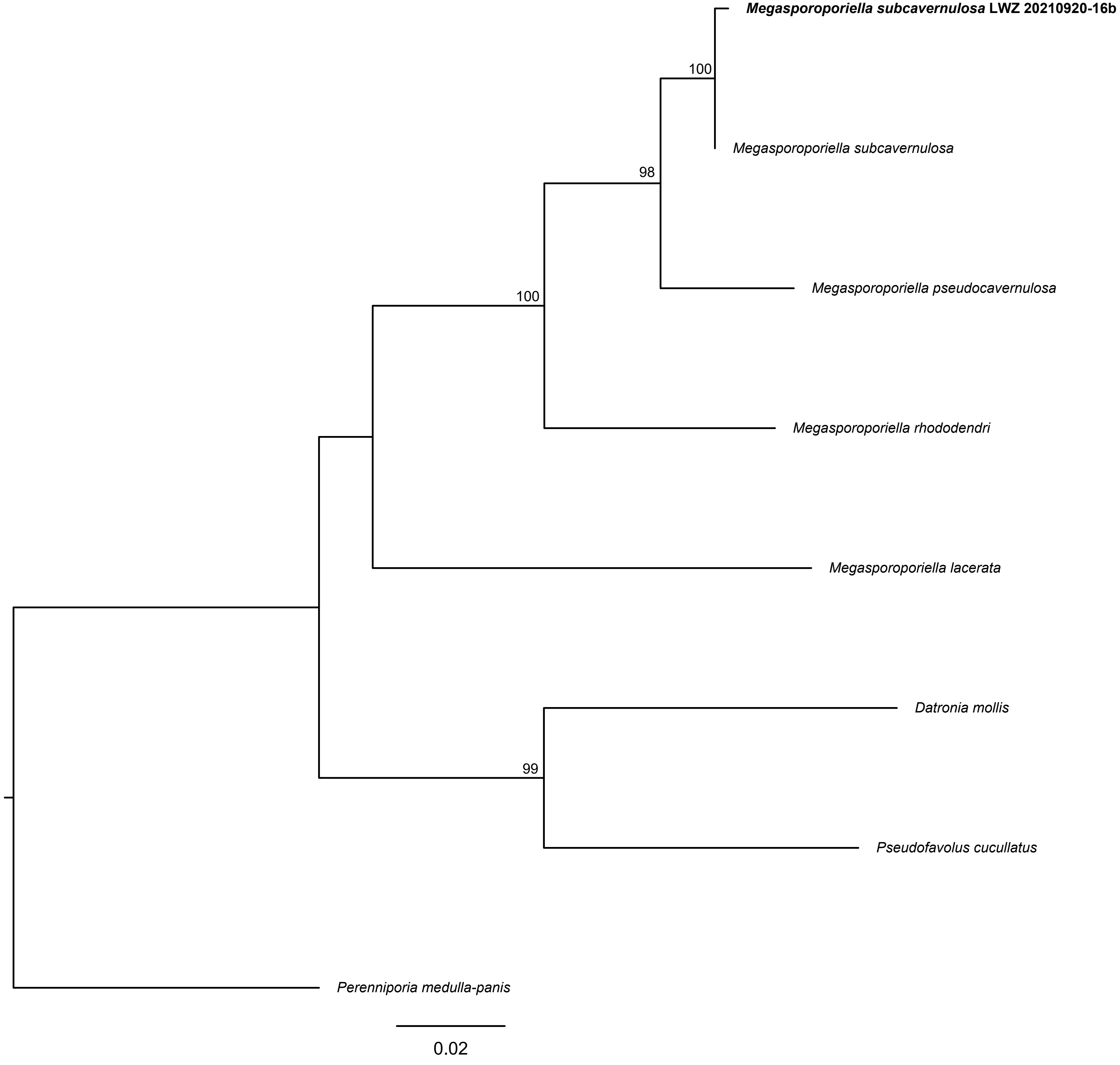

Megasporoporiella subcavernulosa **LWZ 20210920-16b**
100
Megasporoporiella subcavernulosa
98
Megasporoporiella pseudocavernulosa
100
Megasporoporiella rhododendri
Megasporoporiella lacerata
Datronia mollis
99
Pseudofavolus cucullatus
Perenniporia medulla-panis
0.02

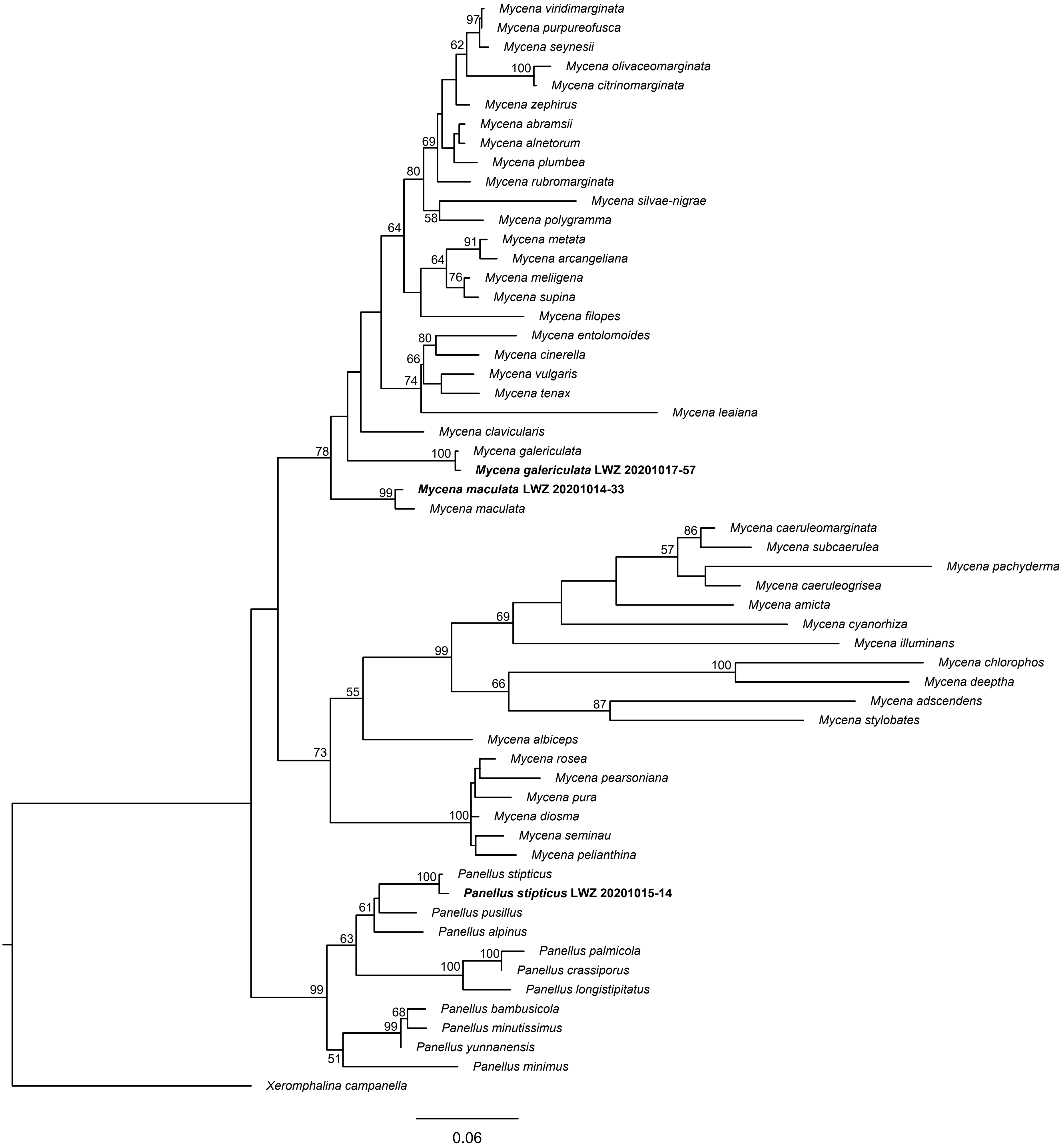

Mycena viridimarginata
Mycena purpureofusca
Mycena seynesii
Mycena olivaceomarginata
Mycena citrinomarginata
Mycena zephirus
Mycena abramsii
Mycena alnetorum
Mycena plumbea
Mycena rubromarginata
Mycena silvae-nigrae
Mycena polygramma
Mycena metata
Mycena arcangeliana
Mycena meliigena
Mycena supina
Mycena filopes
Mycena entolomoides
Mycena cinerella
Mycena vulgaris
Mycena tenax
Mycena leaiana
Mycena clavicularis
Mycena galericulata
Mycena galericulata LWZ 20201017-57
Mycena maculata LWZ 20201014-33
Mycena maculata
Mycena caeruleomarginata
Mycena subcaerulea
Mycena pachyderma
Mycena caeruleogrisea
Mycena amicta
Mycena cyanorhiza
Mycena illuminans
Mycena chlorophos
Mycena deeptha
Mycena adscendens
Mycena stylobates
Mycena albiceps
Mycena rosea
Mycena pearsoniana
Mycena pura
Mycena diosma
Mycena seminau
Mycena pelianthina
Panellus stipticus
Panellus stipticus LWZ 20201015-14
Panellus pusillus
Panellus alpinus
Panellus palmicola
Panellus crassiporus
Panellus longistipitatus
Panellus bambusicola
Panellus minutissimus
Panellus yunnanensis
Panellus minimus
Xeromphalina campanella
97
62
100
69
80
58
64
91
64
76
80
66
74
78
100
99
86
57
69
99
100
66
87
55
73
100
100
61
100
63
100
99
68
99
51
0.06

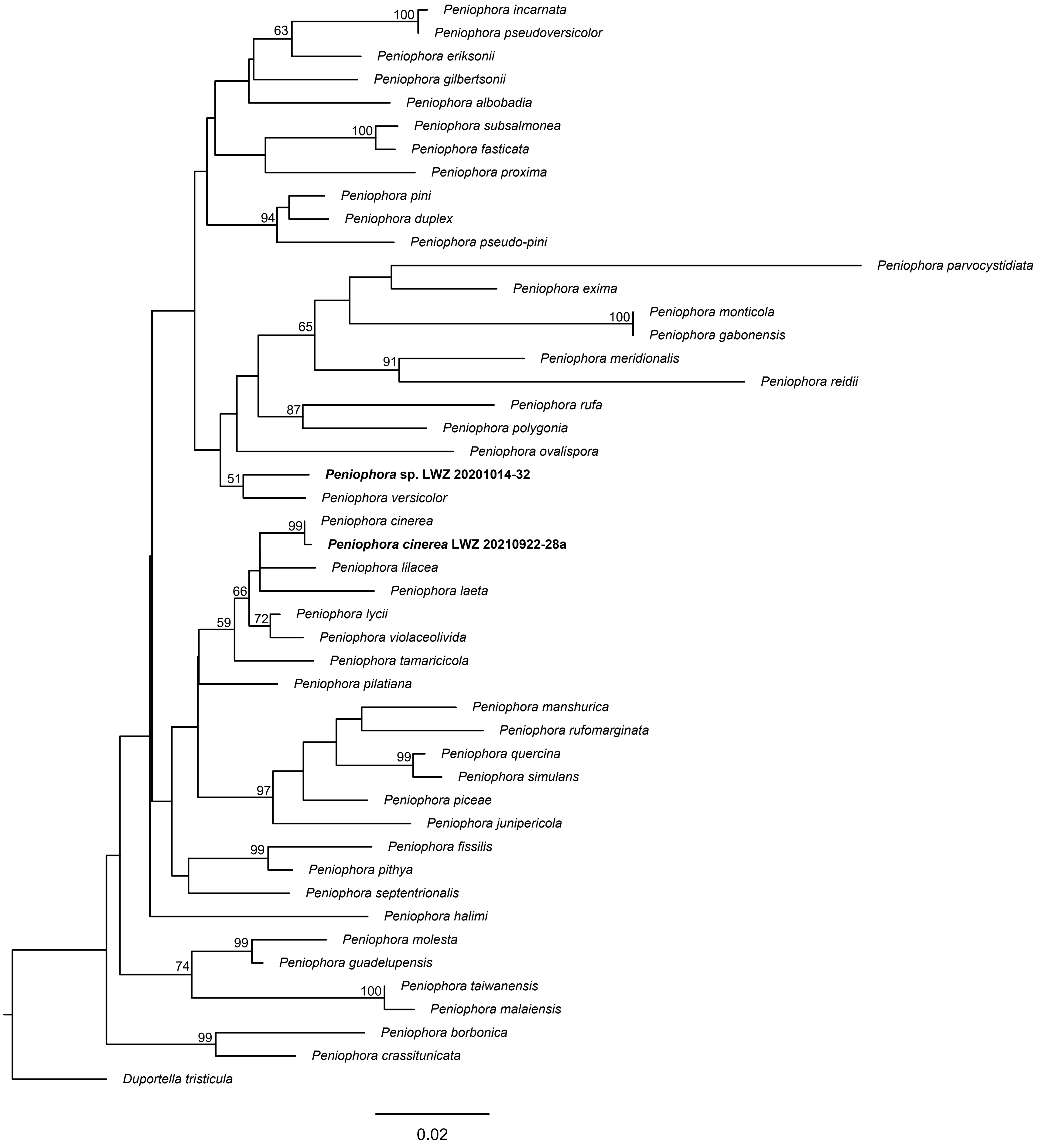

Peniophora incarnata
Peniophora pseudoversicolor
Peniophora eriksonii
Peniophora gilbertsonii
Peniophora albobadia
Peniophora subsalmonea
Peniophora fasticata
Peniophora proxima
Peniophora pini
Peniophora duplex
Peniophora pseudo-pini
Peniophora parvocystidiata
Peniophora exima
Peniophora monticola
Peniophora gabonensis
Peniophora meridionalis
Peniophora reidii
Peniophora rufa
Peniophora polygonia
Peniophora ovalispora
Peniophora sp. LWZ 20201014-32
Peniophora versicolor
Peniophora cinerea
Peniophora cinerea LWZ 20210922-28a
Peniophora lilacea
Peniophora laeta
Peniophora lycii
Peniophora violaceolivida
Peniophora tamaricicola
Peniophora pilatiana
Peniophora manshurica
Peniophora rufomarginata
Peniophora quercina
Peniophora simulans
Peniophora piceae
Peniophora junipericola
Peniophora fissilis
Peniophora pithya
Peniophora septentrionalis
Peniophora halimi
Peniophora molesta
Peniophora guadelupensis
Peniophora taiwanensis
Peniophora malaiensis
Peniophora borbonica
Peniophora crassitunicata
Duportella tristicula
100
63
100
94
65
100
91
87
51
99
66
72
59
99
97
99
99
74
100
99
0.02

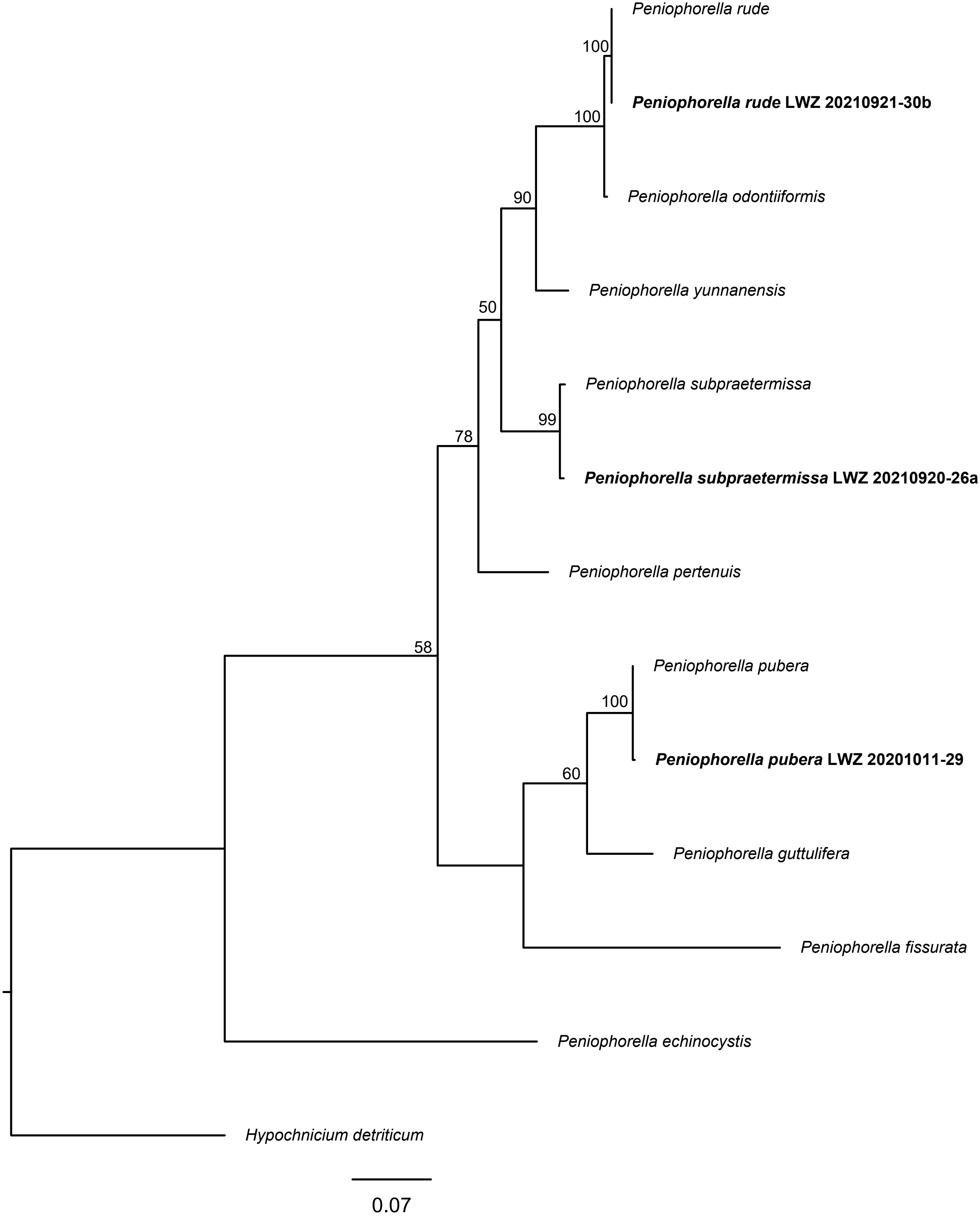
Peniophorella rude
100
Peniophorella rude LWZ 20210921-30b
100
Peniophorella odontiiformis
90
Peniophorella yunnanensis
50
Peniophorella subpraetermissa
99
Peniophorella subpraetermissa LWZ 20210920-26a
78
Peniophorella pertenuis
58
Peniophorella pubera
100
Peniophorella pubera LWZ 20201011-29
60
Peniophorella guttulifera
Peniophorella fissurata
Peniophorella echinocystis
Hypochnicium detriticum
0.07

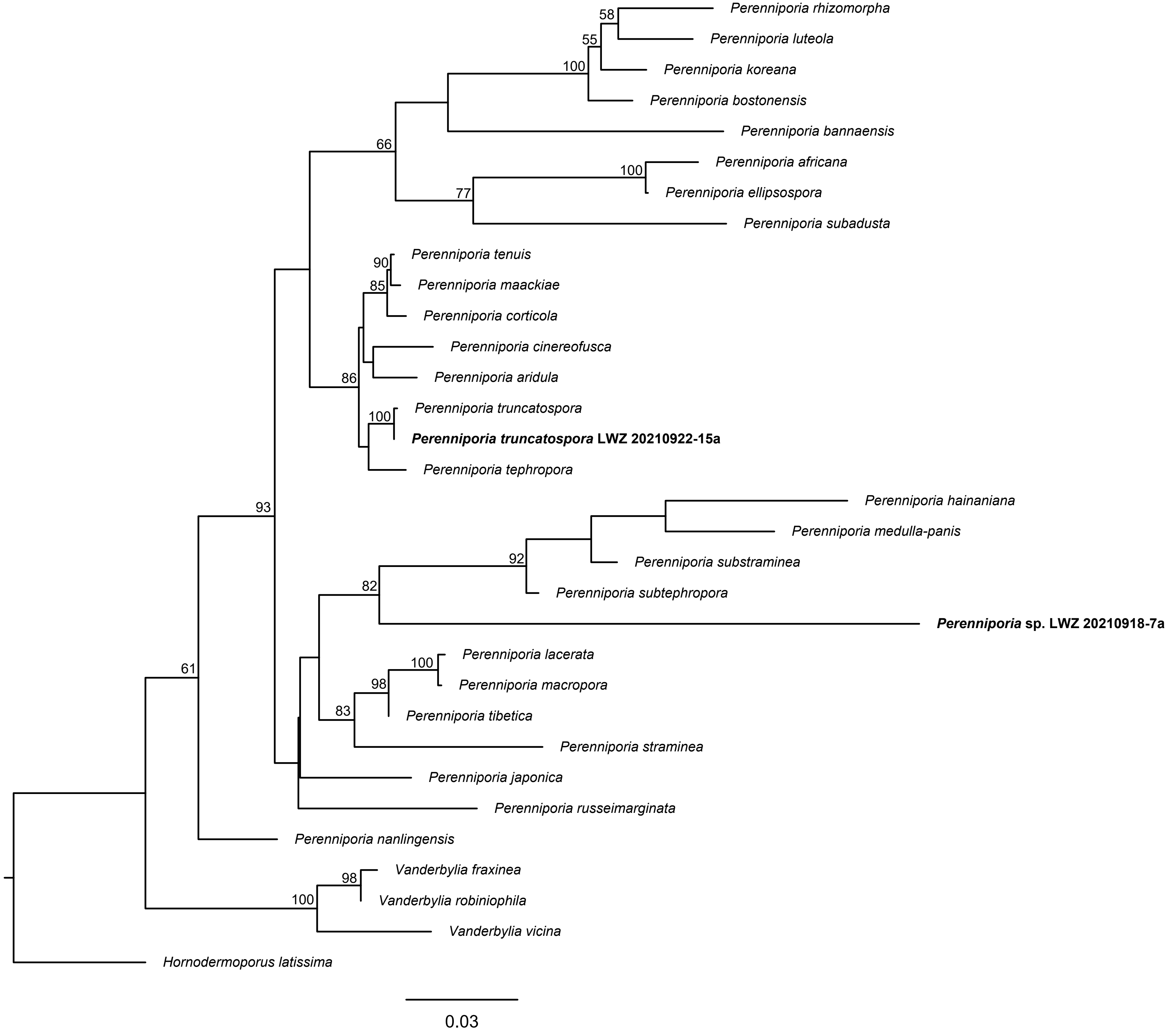
Perenniporia rhizomorpha
Perenniporia luteola
Perenniporia koreana
Perenniporia bostonensis
Perenniporia bannaensis
Perenniporia africana
Perenniporia ellipsospora
Perenniporia subadusta
Perenniporia tenuis
Perenniporia maackiae
Perenniporia corticola
Perenniporia cinereofusca
Perenniporia aridula
Perenniporia truncatospora
Perenniporia truncatospora LWZ 20210922-15a
Perenniporia tephropora
Perenniporia hainaniana
Perenniporia medulla-panis
Perenniporia substraminea
Perenniporia subtephropora
Perenniporia sp. LWZ 20210918-7a
Perenniporia lacerata
Perenniporia macropora
Perenniporia tibetica
Perenniporia straminea
Perenniporia japonica
Perenniporia russeimarginata
Perenniporia nanlingensis
Vanderbylia fraxinea
Vanderbylia robiniophila
Vanderbylia vicina
Hornodermoporus latissima
58
55
100
66
100
77
90
85
86
100
93
92
82
100
98
83
61
98
100
0.03

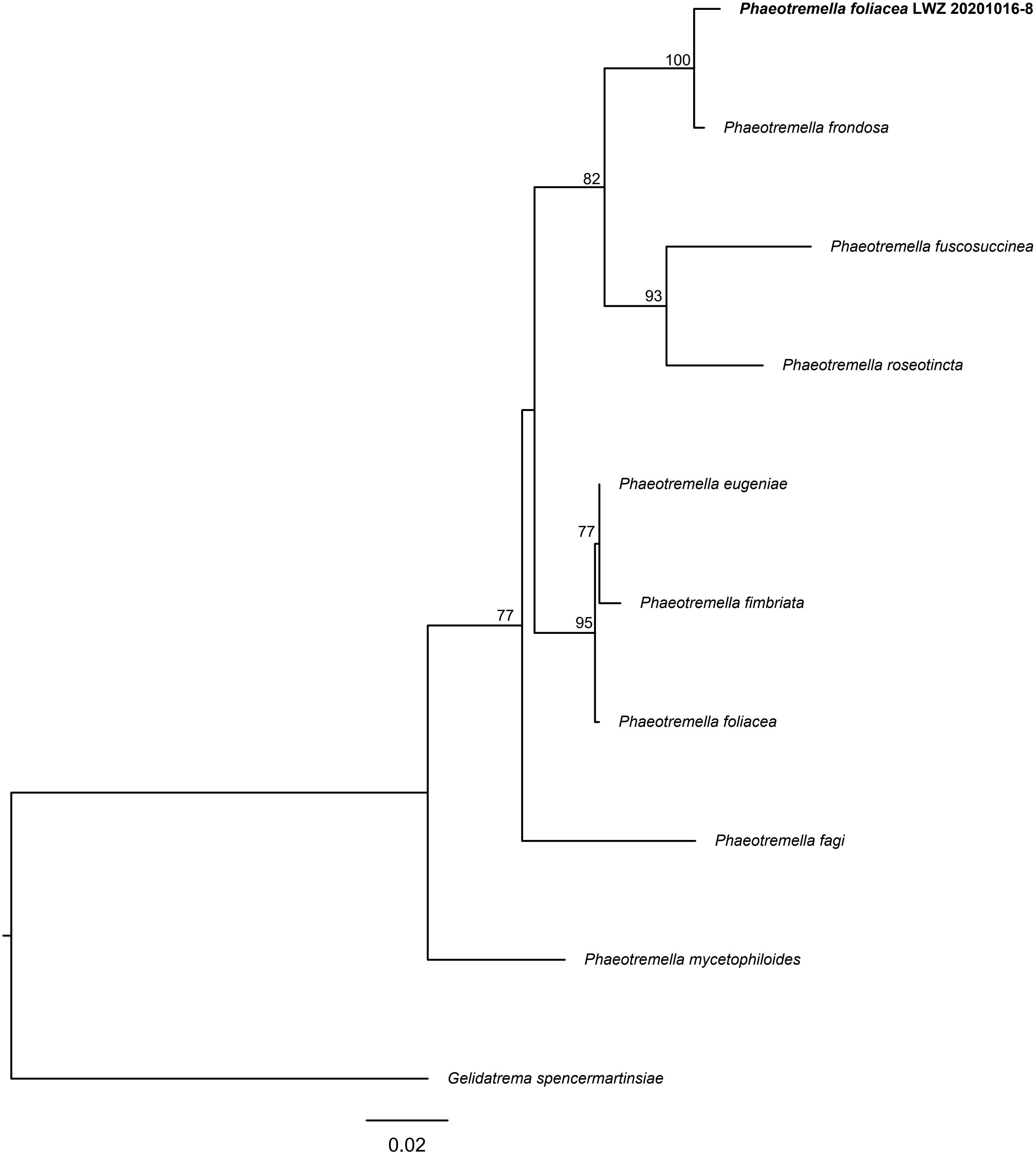

Phaeotremella foliacea LWZ 20201016-8
100
Phaeotremella frondosa
82
Phaeotremella fuscosuccinea
93
Phaeotremella roseotincta
Phaeotremella eugeniae
77
Phaeotremella fimbriata
77
95
Phaeotremella foliacea
Phaeotremella fagi
Phaeotremella mycetophiloides
Gelidatrema spencermartinsiae
0.02

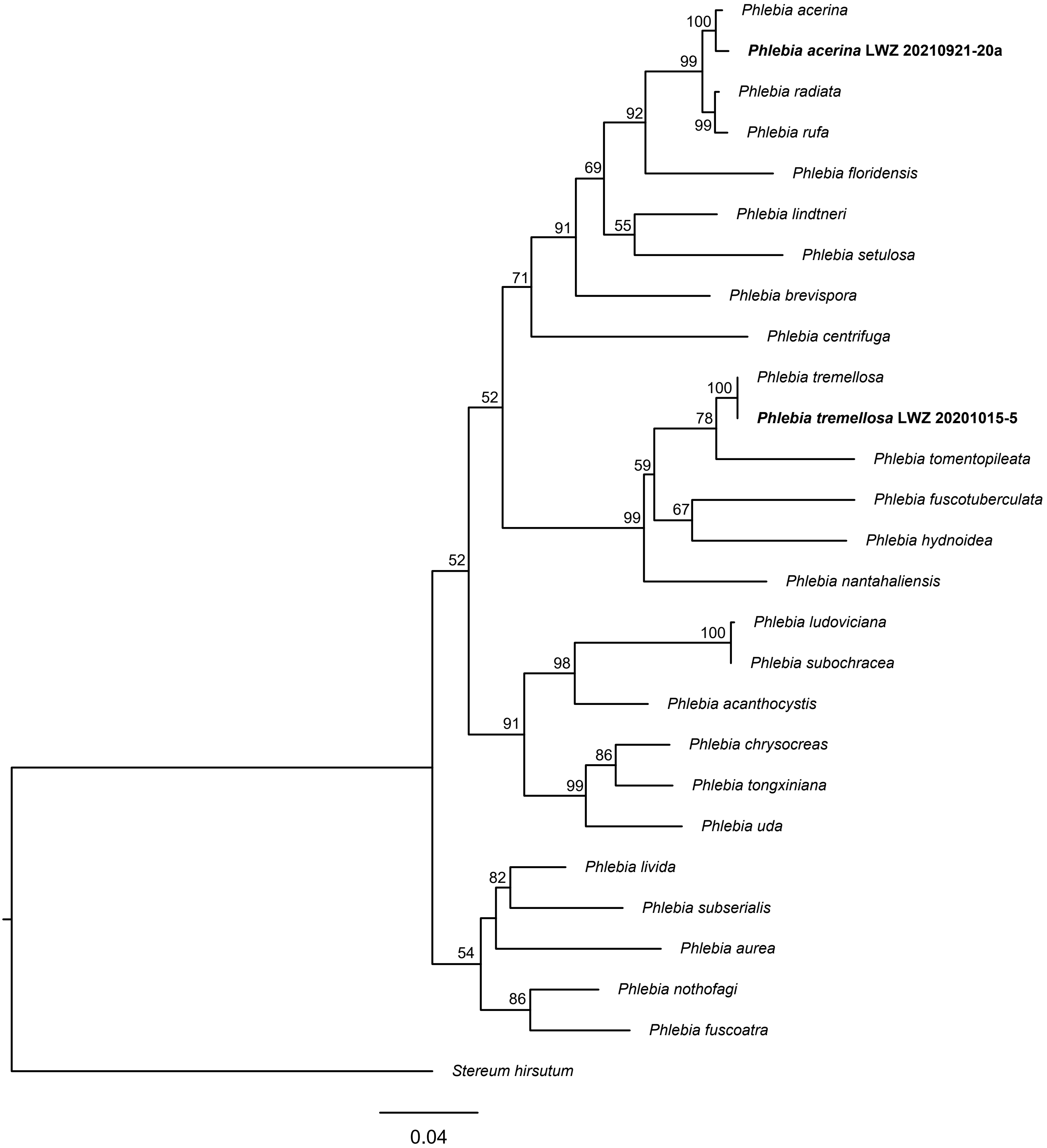

Phlebia acerina
Phlebia acerina LWZ 20210921-20a
Phlebia radiata
Phlebia rufa
Phlebia floridensis
Phlebia lindtneri
Phlebia setulosa
Phlebia brevispora
Phlebia centrifuga
Phlebia tremellosa
Phlebia tremellosa LWZ 20201015-5
Phlebia tomentopileata
Phlebia fuscotuberculata
Phlebia hydnoidea
Phlebia nantahaliensis
Phlebia ludoviciana
Phlebia subochracea
Phlebia acanthocystis
Phlebia chrysocreas
Phlebia tongxiniana
Phlebia uda
Phlebia livida
Phlebia subserialis
Phlebia aurea
Phlebia nothofagi
Phlebia fuscoatra
Stereum hirsutum
100
99
99
92
69
55
91
71
52
100
78
59
67
99
52
100
98
91
86
99
82
54
86
0.04

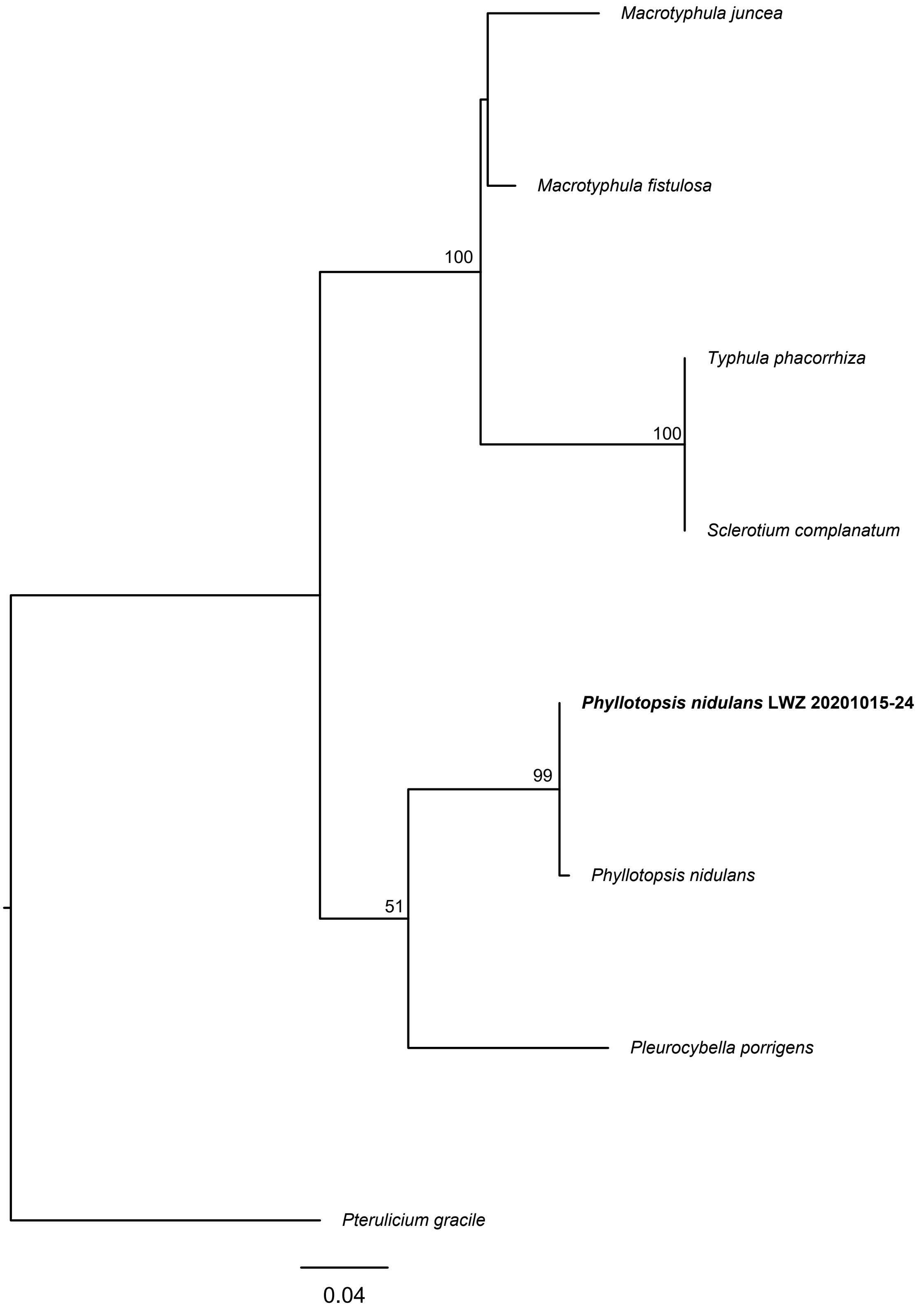
Macrotyphula juncea
Macrotyphula fistulosa
100
Typhula phacorrhiza
100
Sclerotium complanatum
Phyllotopsis nidulans LWZ 20201015-24
99
Phyllotopsis nidulans
51
Pleurocybella porrigens
Pterulicium gracile
0.04

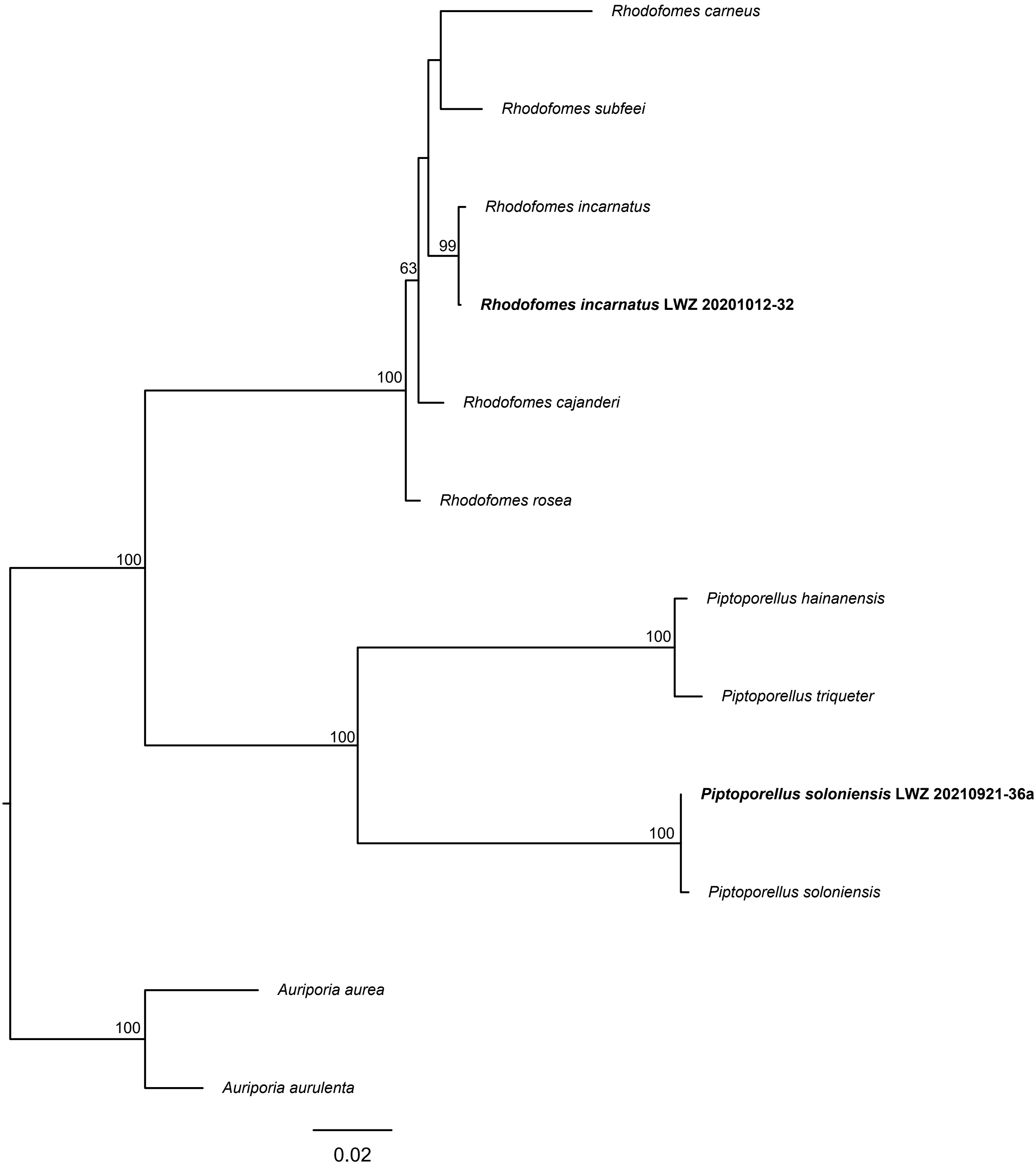
Rhodofomes carneus
Rhodofomes subfeei
Rhodofomes incarnatus
99
63
Rhodofomes incarnatus LWZ 20201012-32
100
Rhodofomes cajanderi
Rhodofomes rosea
100
Piptoporellus hainanensis
100
Piptoporellus triqueter
100
Piptoporellus soloniensis LWZ 20210921-36a
100
Piptoporellus soloniensis
Auriporia aurea
100
Auriporia aurulenta
0.02

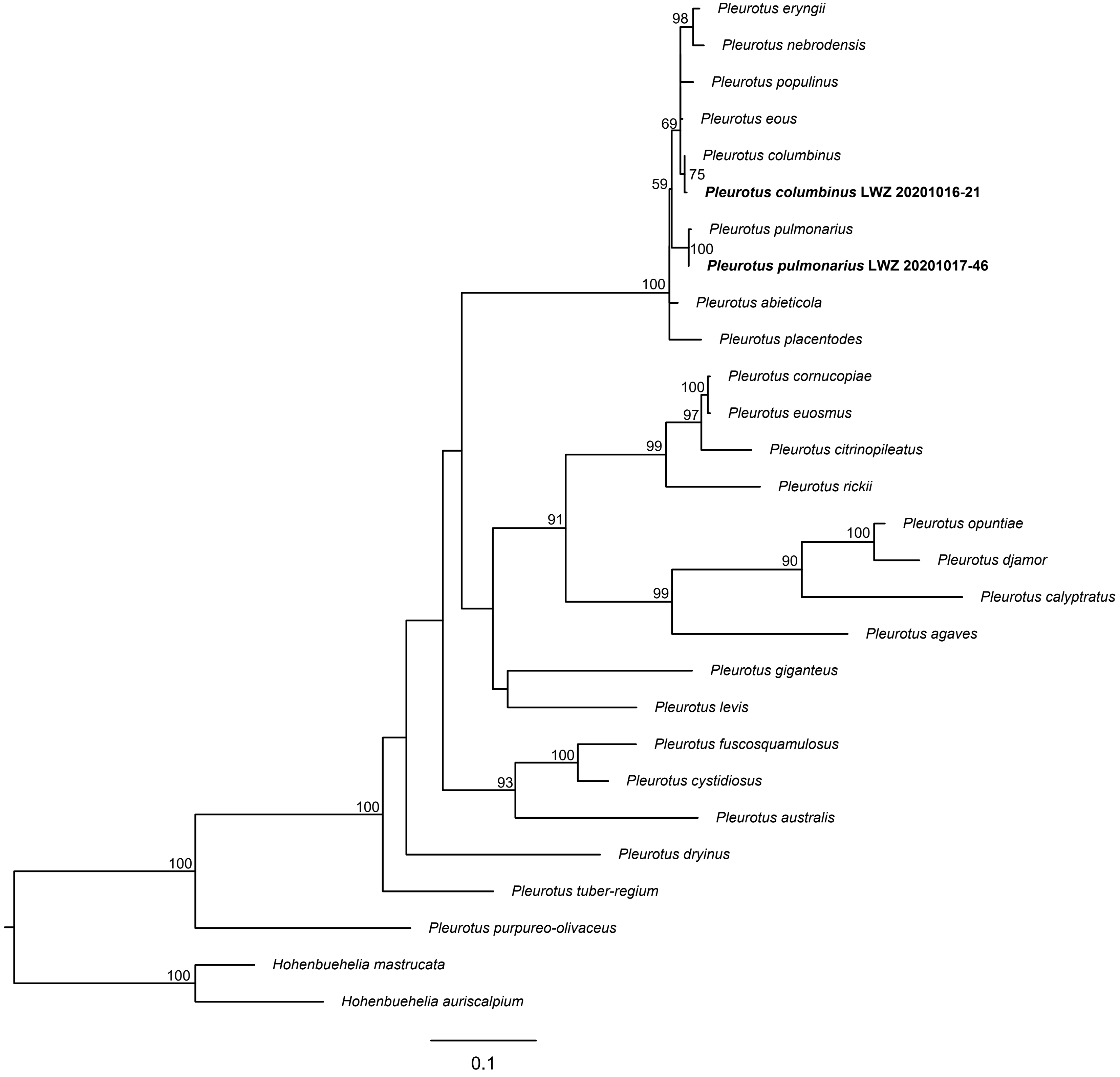
Pleurotus eryngii
98
Pleurotus nebrodensis
Pleurotus populinus
Pleurotus eous
69
Pleurotus columbinus
75
59
Pleurotus columbinus LWZ 20201016-21
Pleurotus pulmonarius
100
Pleurotus pulmonarius LWZ 20201017-46
100
Pleurotus abieticola
Pleurotus placentodes
Pleurotus cornucopiae
100
Pleurotus euosmus
97
99
Pleurotus citrinopileatus
Pleurotus rickii
91
Pleurotus opuntiae
100
90
Pleurotus djamor
Pleurotus calyptratus
99
Pleurotus agaves
Pleurotus giganteus
Pleurotus levis
Pleurotus fuscosquamulosus
100
Pleurotus cystidiosus
93
Pleurotus australis
100
Pleurotus dryinus
100
Pleurotus tuber-regium
Pleurotus purpureo-olivaceus
Hohenbuehelia mastrucata
100
Hohenbuehelia auriscalpium
0.1

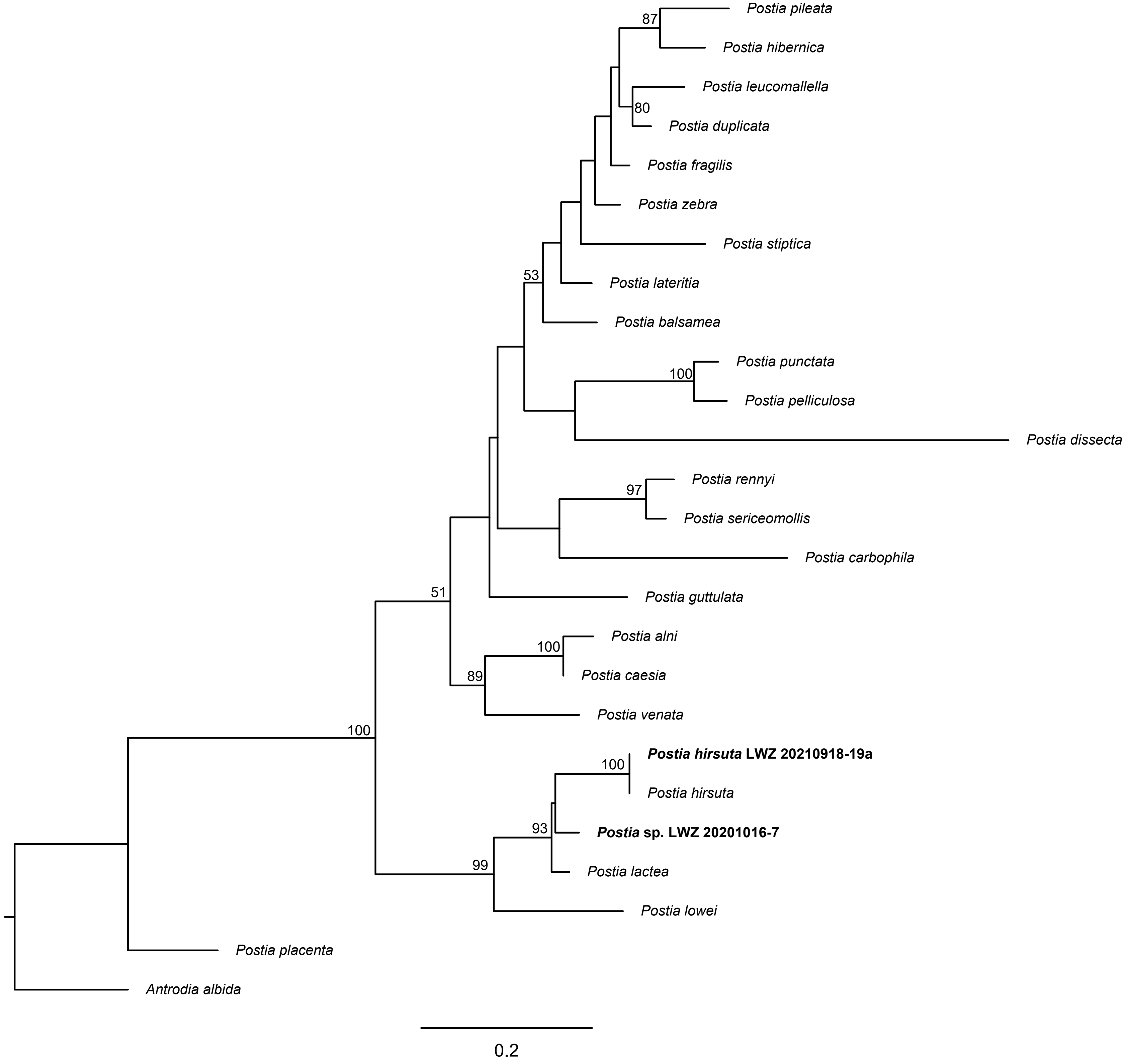

87
Postia pileata
Postia hibernica
Postia leucomallella
80
Postia duplicata
Postia fragilis
Postia zebra
Postia stiptica
53
Postia lateritia
Postia balsamea
100
Postia punctata
Postia pelliculosa
Postia dissecta
97
Postia rennyi
Postia sericeomollis
Postia carbophila
51
Postia guttulata
100
Postia alni
Postia caesia
89
Postia venata
100
100
Postia hirsuta LWZ 20210918-19a
Postia hirsuta
93
Postia sp. LWZ 20201016-7
99
Postia lactea
Postia lowei
Postia placenta
Antrodia albida
0.2

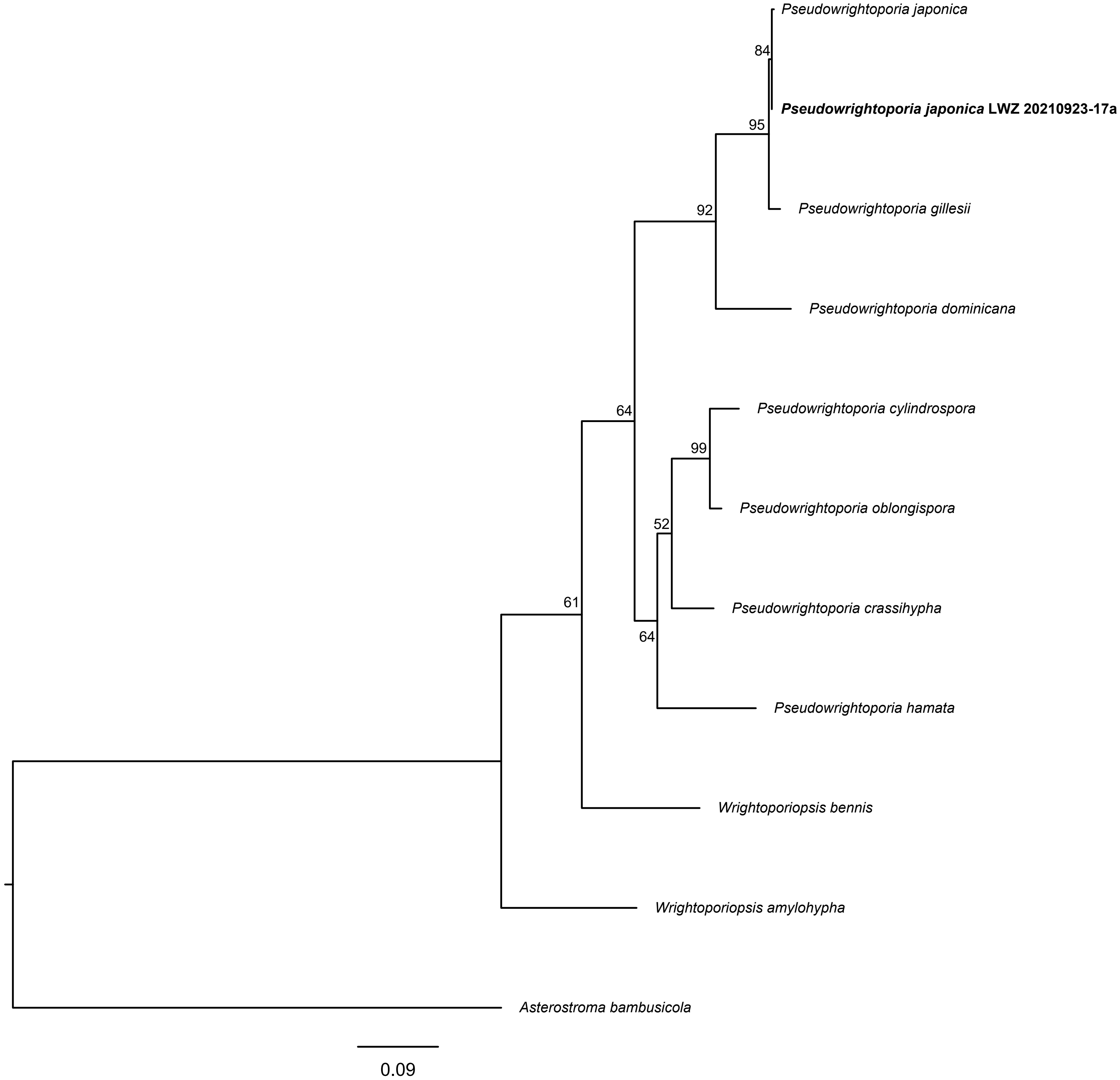

Pseudowrightoporia japonica
84
Pseudowrightoporia japonica LWZ 20210923-17a
95
Pseudowrightoporia gillesii
92
Pseudowrightoporia dominicana
64
Pseudowrightoporia cylindrospora
99
Pseudowrightoporia oblongispora
52
Pseudowrightoporia crassihypha
61
64
Pseudowrightoporia hamata
Wrightoporiopsis bennis
Wrightoporiopsis amylohypha
Asterostroma bambusicola
0.09

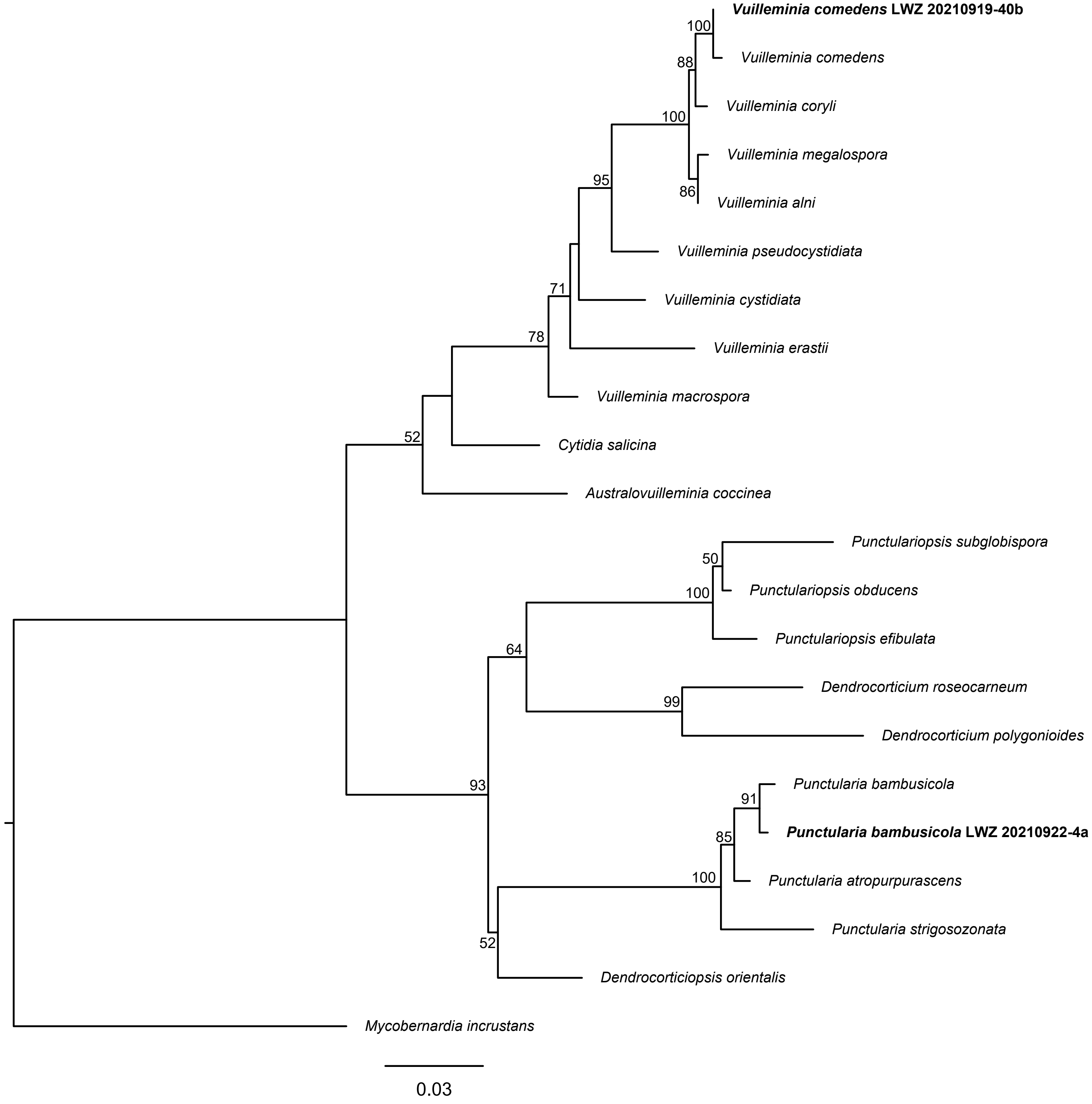

Vuilleminia comedens LWZ 20210919-40b
Vuilleminia comedens
Vuilleminia coryli
Vuilleminia megalospora
Vuilleminia alni
Vuilleminia pseudocystidiata
Vuilleminia cystidiata
Vuilleminia erastii
Vuilleminia macrospora
Cytidia salicina
Australovuilleminia coccinea
Punctulariopsis subglobispora
Punctulariopsis obducens
Punctulariopsis efibulata
Dendrocorticium roseocarneum
Dendrocorticium polygonioides
Punctularia bambusicola
Punctularia bambusicola LWZ 20210922-4a
Punctularia atropurpurascens
Punctularia strigosozonata
Dendrocorticiopsis orientalis
Mycobernardia incrustans
100
88
100
95
86
71
78
52
50
100
64
99
93
91
85
100
52
0.03

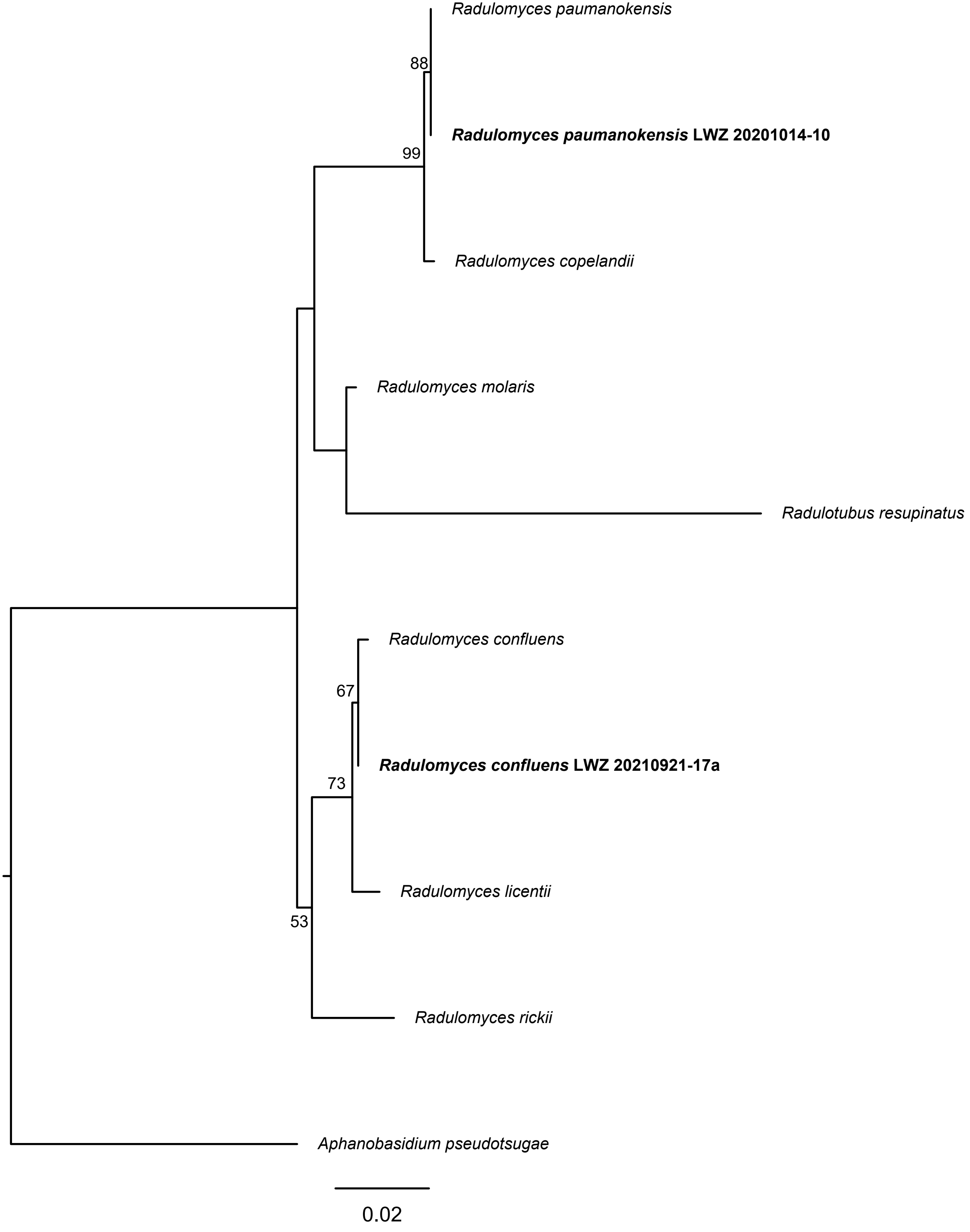
Radulomyces paumanokensis
88
Radulomyces paumanokensis LWZ 20201014-10
99
Radulomyces copelandii
Radulomyces molaris
Radulotubus resupinatus
Radulomyces confluens
67
Radulomyces confluens LWZ 20210921-17a
73
Radulomyces licentii
53
Radulomyces rickii
Aphanobasidium pseudotsugae
0.02

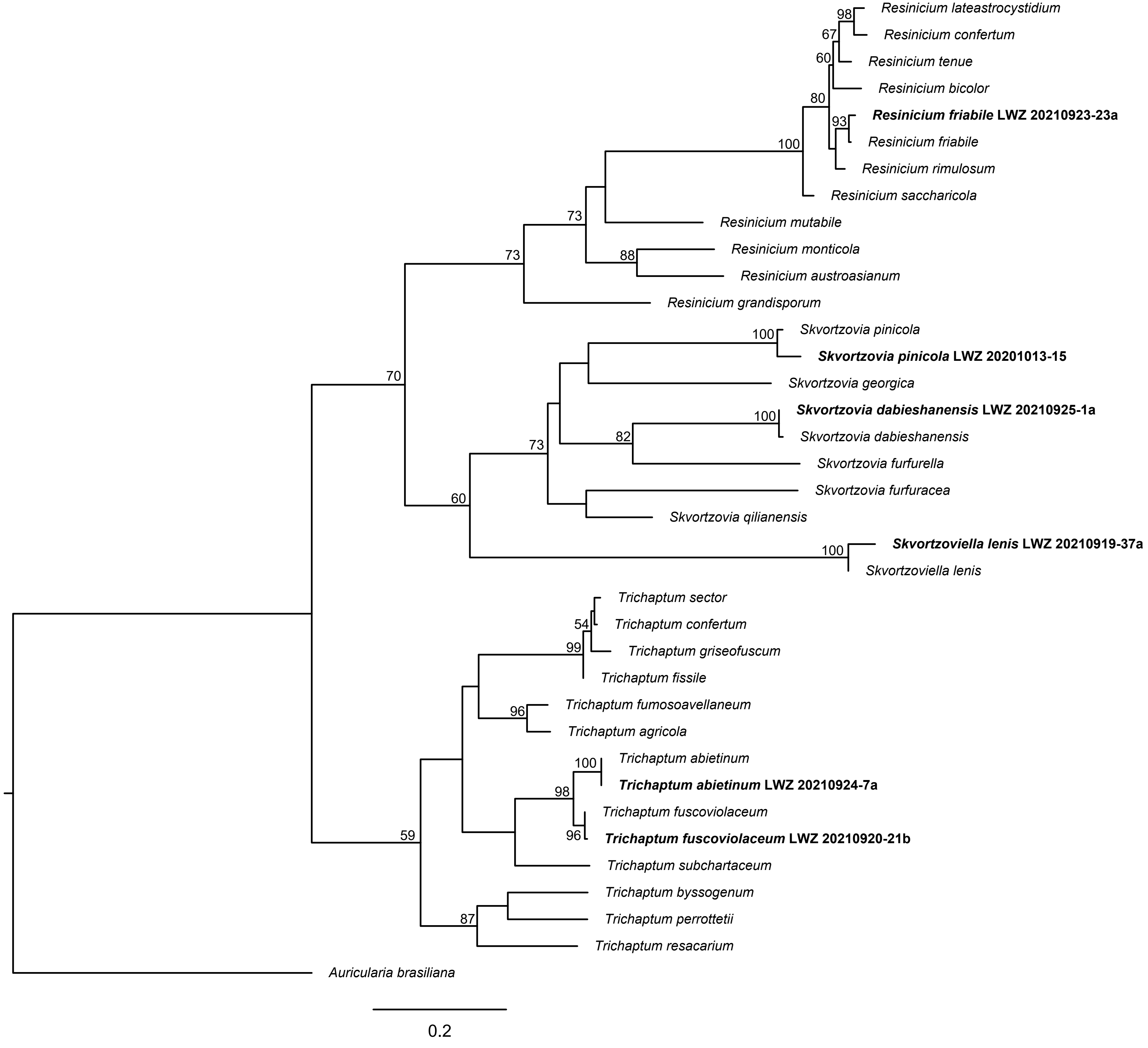

Resinicium lateastrocystidium
Resinicium confertum
Resinicium tenue
Resinicium bicolor
Resinicium friabile LWZ 20210923-23a
Resinicium friabile
Resinicium rimulosum
Resinicium saccharicola
Resinicium mutabile
Resinicium monticola
Resinicium austroasianum
Resinicium grandisporum
Skvortzovia pinicola
Skvortzovia pinicola LWZ 20201013-15
Skvortzovia georgica
Skvortzovia dabieshanensis LWZ 20210925-1a
Skvortzovia dabieshanensis
Skvortzovia furfurella
Skvortzovia furfuracea
Skvortzovia qilianensis
Skvortzoviella lenis LWZ 20210919-37a
Skvortzoviella lenis
Trichaptum sector
Trichaptum confertum
Trichaptum griseofuscum
Trichaptum fissile
Trichaptum fumosoavellaneum
Trichaptum agricola
Trichaptum abietinum
Trichaptum abietinum LWZ 20210924-7a
Trichaptum fuscoviolaceum
Trichaptum fuscoviolaceum LWZ 20210920-21b
Trichaptum subchartaceum
Trichaptum byssogenum
Trichaptum perrottetii
Trichaptum resacarium
Auricularia brasiliana
98
67
60
80
93
100
73
88
73
100
70
100
82
73
60
100
54
99
96
100
98
96
59
87
0.2

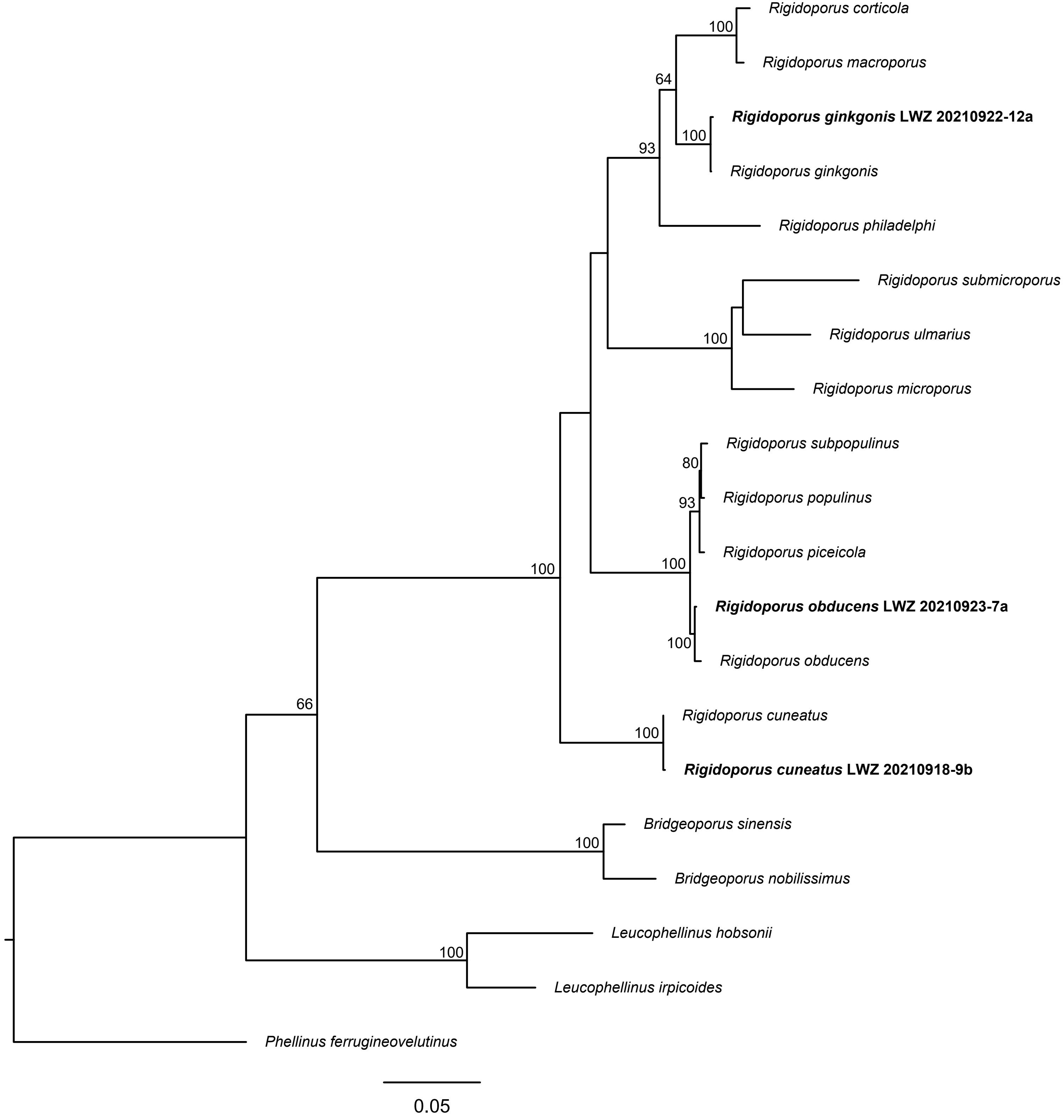
Rigidoporus corticola
Rigidoporus macroporus
Rigidoporus ginkgonis LWZ 20210922-12a
Rigidoporus ginkgonis
Rigidoporus philadelphi
Rigidoporus submicroporus
Rigidoporus ulmarius
Rigidoporus microporus
Rigidoporus subpopulinus
Rigidoporus populinus
Rigidoporus piceicola
Rigidoporus obducens LWZ 20210923-7a
Rigidoporus obducens
Rigidoporus cuneatus
Rigidoporus cuneatus LWZ 20210918-9b
Bridgeoporus sinensis
Bridgeoporus nobilissimus
Leucophellinus hobsonii
Leucophellinus irpicoides
Phellinus ferrugineovelutinus
100
64
100
93
100
80
93
100
100
100
100
66
100
100
0.05

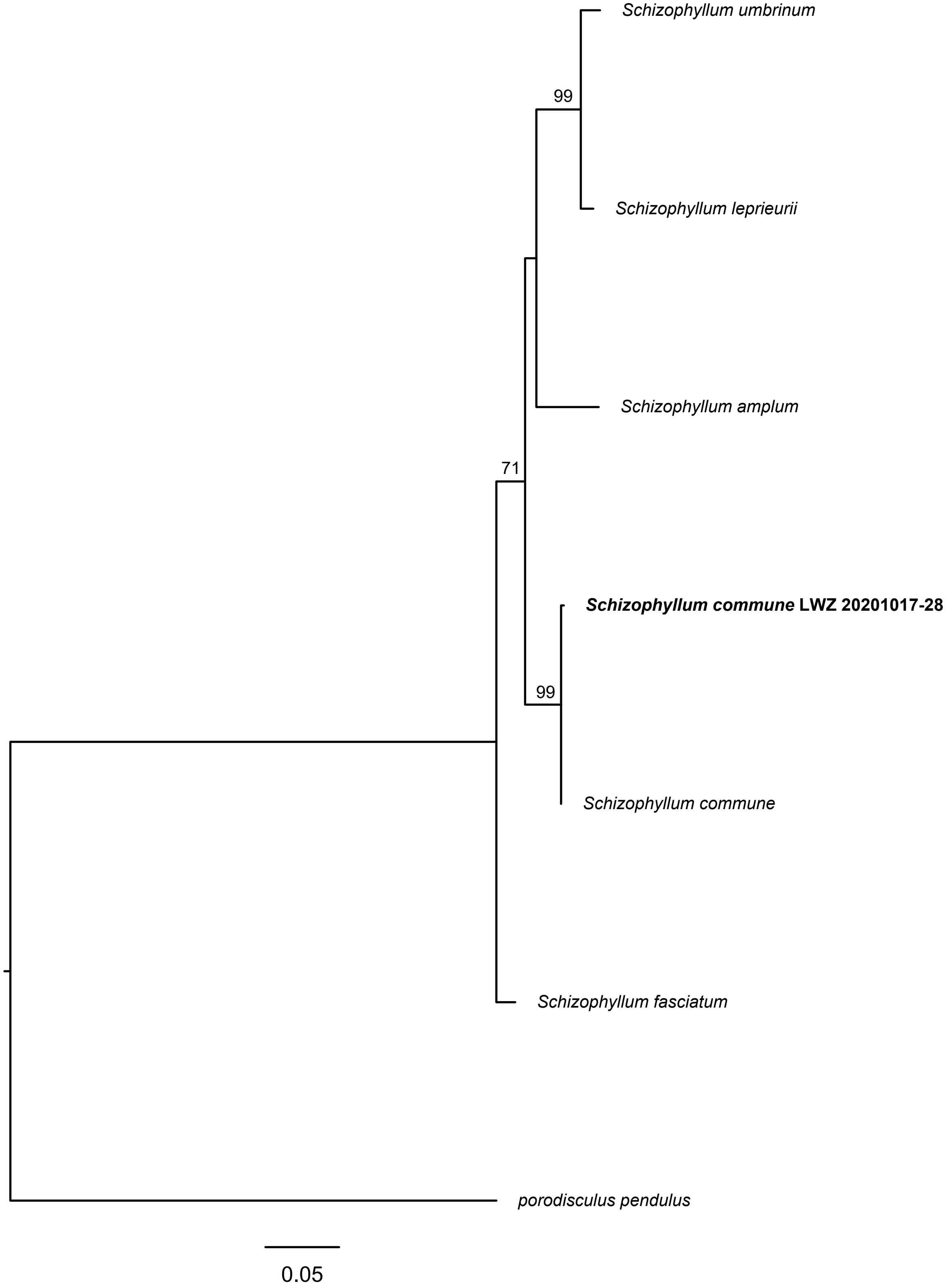
Schizophyllum umbrinum
99
Schizophyllum leprieurii
Schizophyllum amplum
71
Schizophyllum commune LWZ 20201017-28
99
Schizophyllum commune
Schizophyllum fasciatum
porodisculus pendulus
0.05

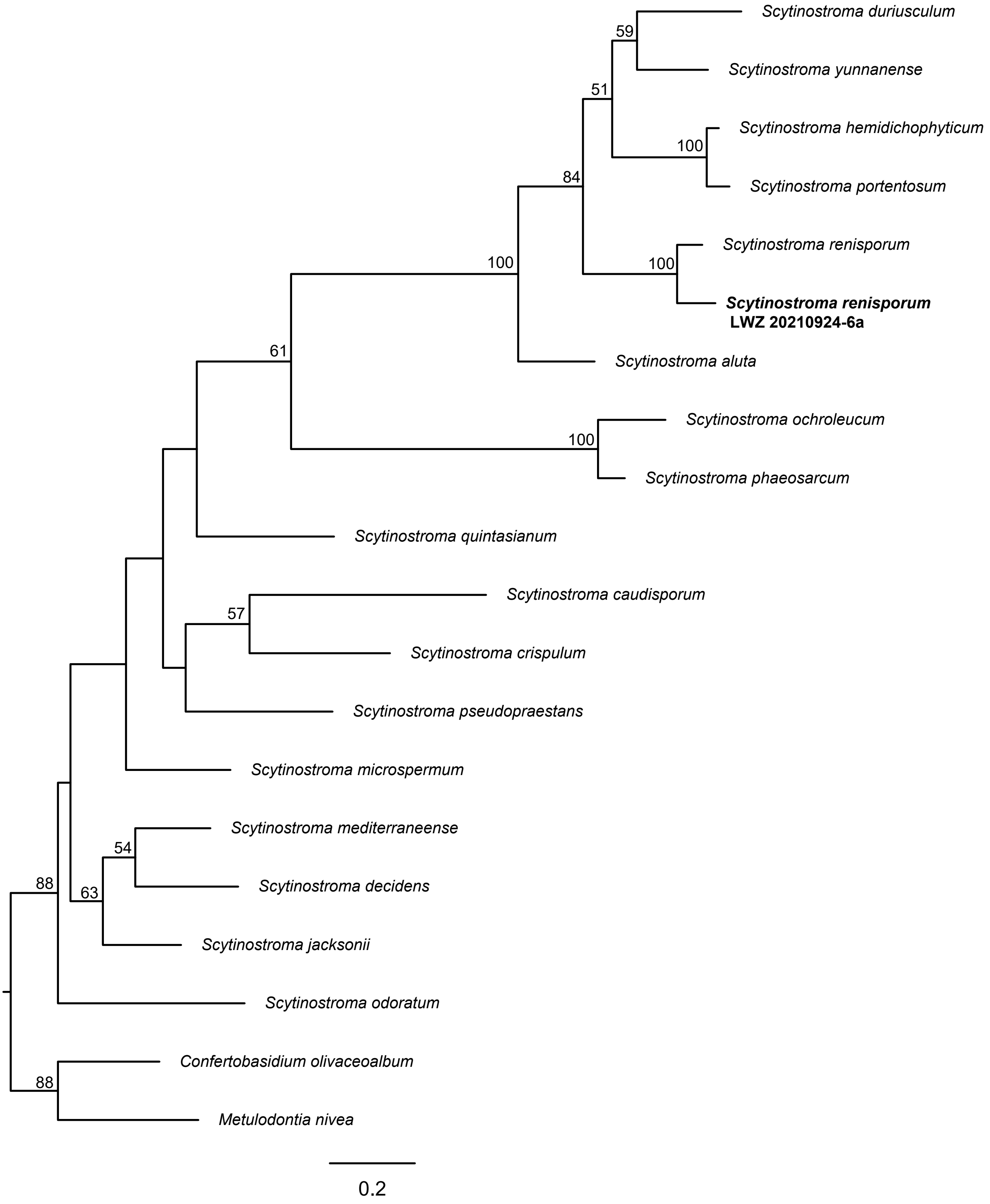
Scytinostroma duriusculum
Scytinostroma yunnanense
Scytinostroma hemidichophyticum
Scytinostroma portentosum
Scytinostroma renisporum
Scytinostroma renisporum LWZ 20210924-6a
Scytinostroma aluta
Scytinostroma ochroleucum
Scytinostroma phaeosarcum
Scytinostroma quintasianum
Scytinostroma caudisporum
Scytinostroma crispulum
Scytinostroma pseudopraestans
Scytinostroma microspermum
Scytinostroma mediterraneense
Scytinostroma decidens
Scytinostroma jacksonii
Scytinostroma odoratum
Confertobasidium olivaceoalbum
Metulodontia nivea
59
51
100
84
100
100
61
100
57
54
63
88
88
0.2

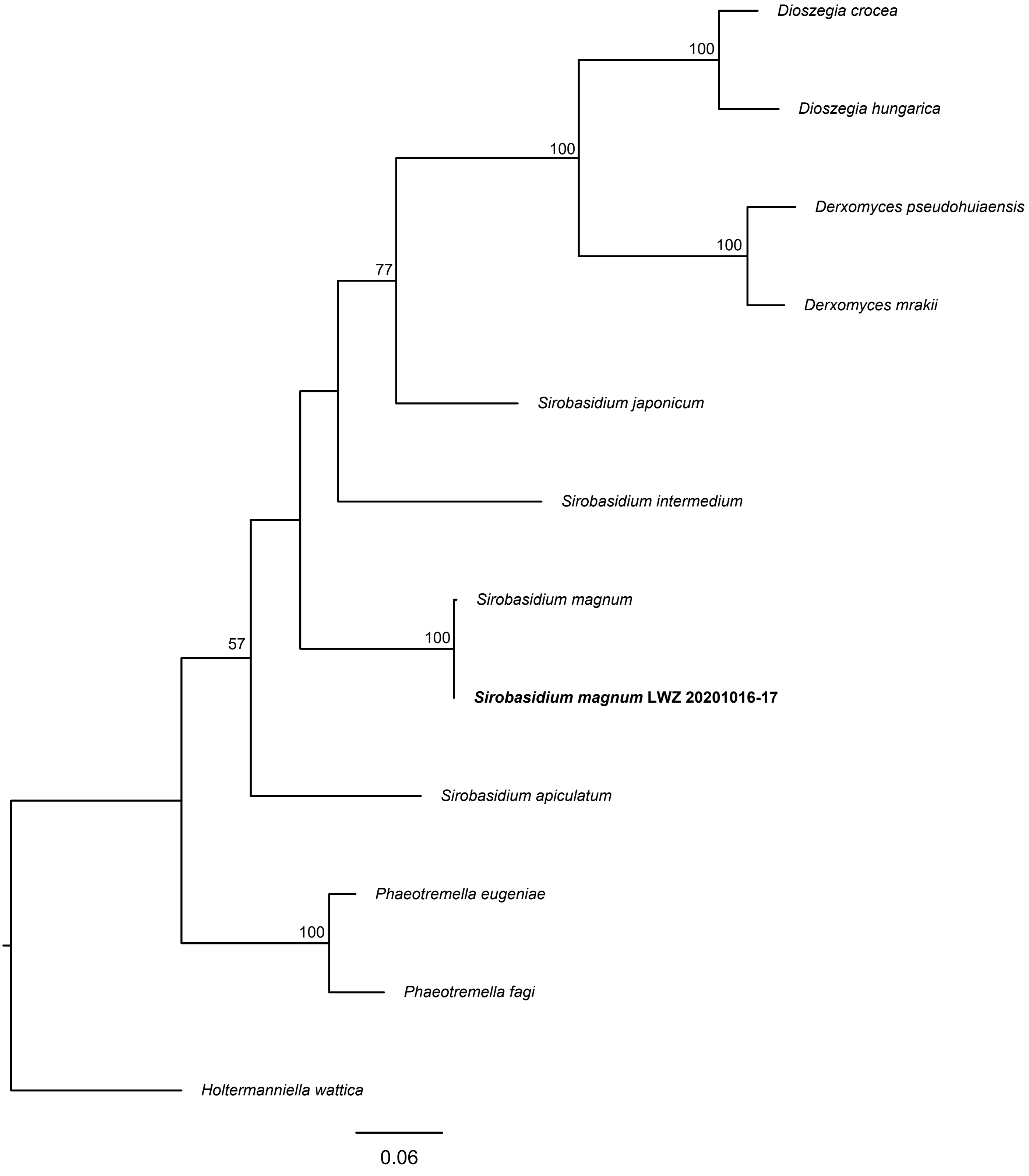
Dioszegia crocea
100
Dioszegia hungarica
100
Derxomyces pseudohuiaensis
100
Derxomyces mrakii
77
Sirobasidium japonicum
Sirobasidium intermedium
Sirobasidium magnum
100
Sirobasidium magnum LWZ 20201016-17
57
Sirobasidium apiculatum
Phaeotremella eugeniae
100
Phaeotremella fagi
Holtermanniella wattica
0.06

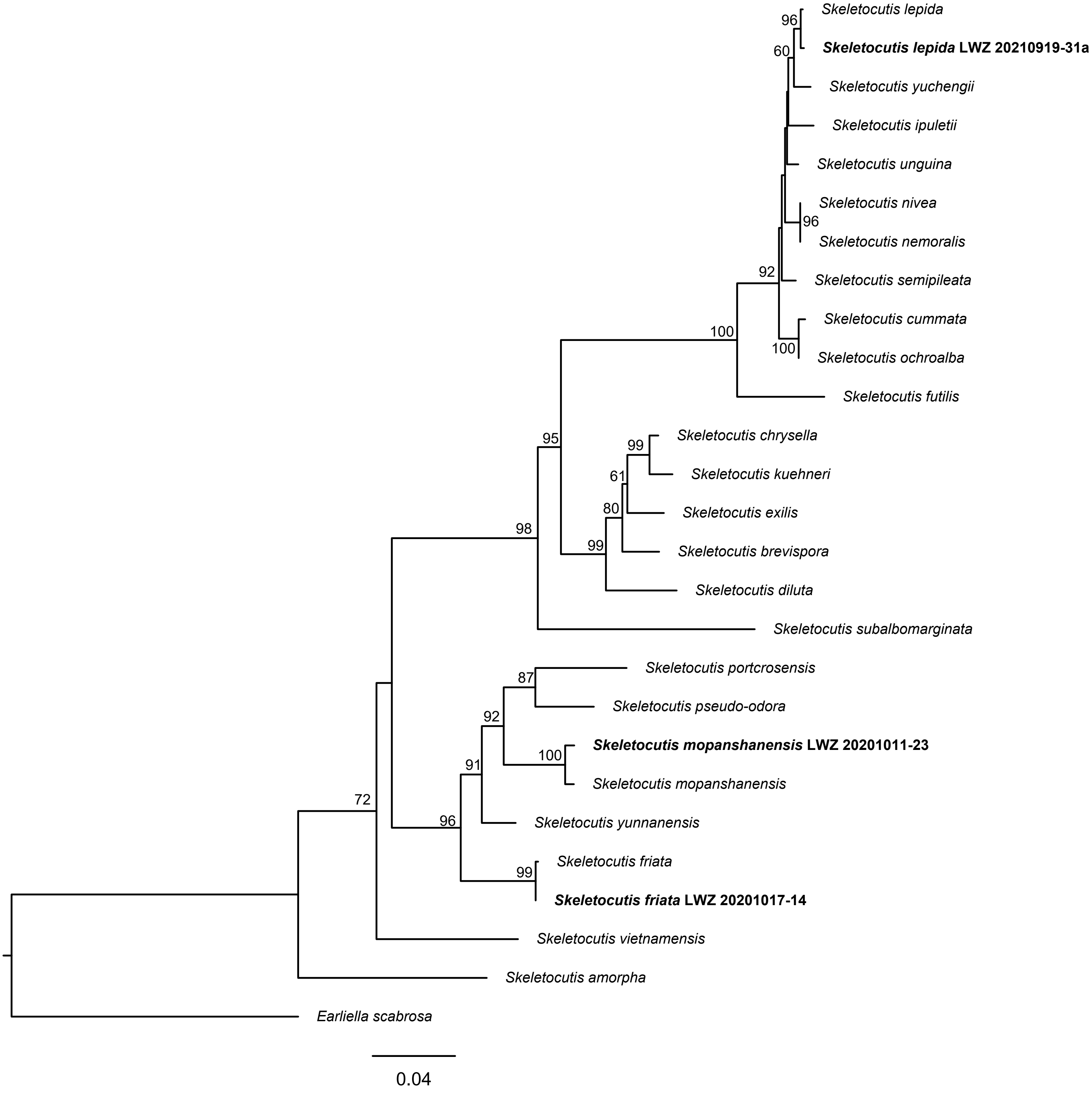

Skeletocutis lepida
96
Skeletocutis lepida LWZ 20210919-31a
60
Skeletocutis yuchengii
Skeletocutis ipuletii
Skeletocutis unguina
Skeletocutis nivea
96
Skeletocutis nemoralis
92
Skeletocutis semipileata
Skeletocutis cummata
100
100
Skeletocutis ochroalba
Skeletocutis futilis
Skeletocutis chrysella
95
99
Skeletocutis kuehneri
61
Skeletocutis exilis
80
98
99
Skeletocutis brevispora
Skeletocutis diluta
Skeletocutis subalbomarginata
Skeletocutis portcrosensis
87
Skeletocutis pseudo-odora
92
Skeletocutis mopanshanensis LWZ 20201011-23
100
91
Skeletocutis mopanshanensis
72
Skeletocutis yunnanensis
96
Skeletocutis friata
99
Skeletocutis friata LWZ 20201017-14
Skeletocutis vietnamensis
Skeletocutis amorpha
Earliella scabrosa
0.04

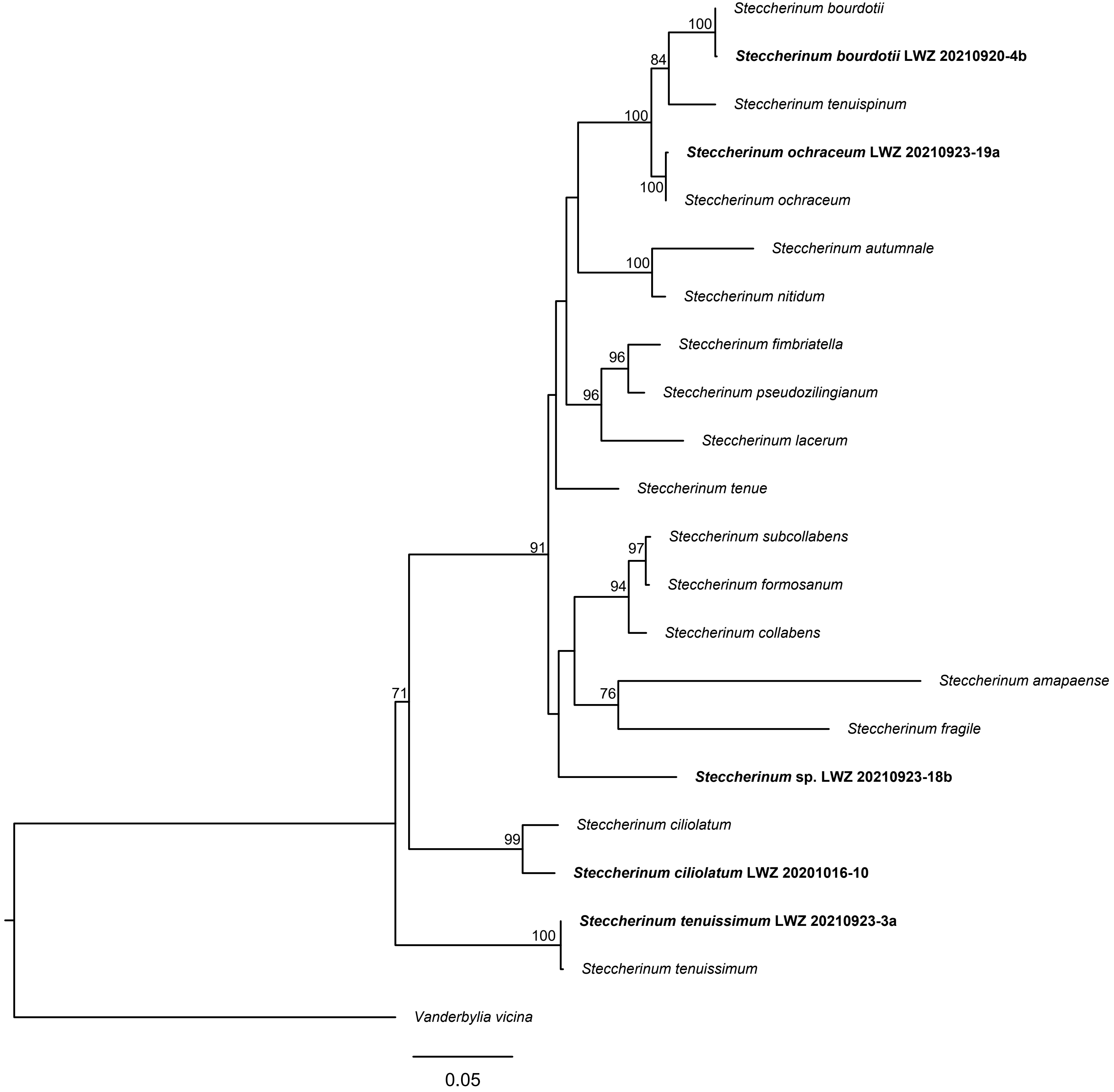

Steccherinum bourdotii
Steccherinum bourdotii LWZ 20210920-4b
Steccherinum tenuispinum
Steccherinum ochraceum LWZ 20210923-19a
Steccherinum ochraceum
Steccherinum autumnale
Steccherinum nitidum
Steccherinum fimbriatella
Steccherinum pseudozilingianum
Steccherinum lacerum
Steccherinum tenue
Steccherinum subcollabens
Steccherinum formosanum
Steccherinum collabens
Steccherinum amapaense
Steccherinum fragile
Steccherinum sp. LWZ 20210923-18b
Steccherinum ciliolatum
Steccherinum ciliolatum LWZ 20201016-10
Steccherinum tenuissimum LWZ 20210923-3a
Steccherinum tenuissimum
Vanderbylia vicina
100
84
100
100
100
96
96
91
97
94
76
71
99
100
0.05

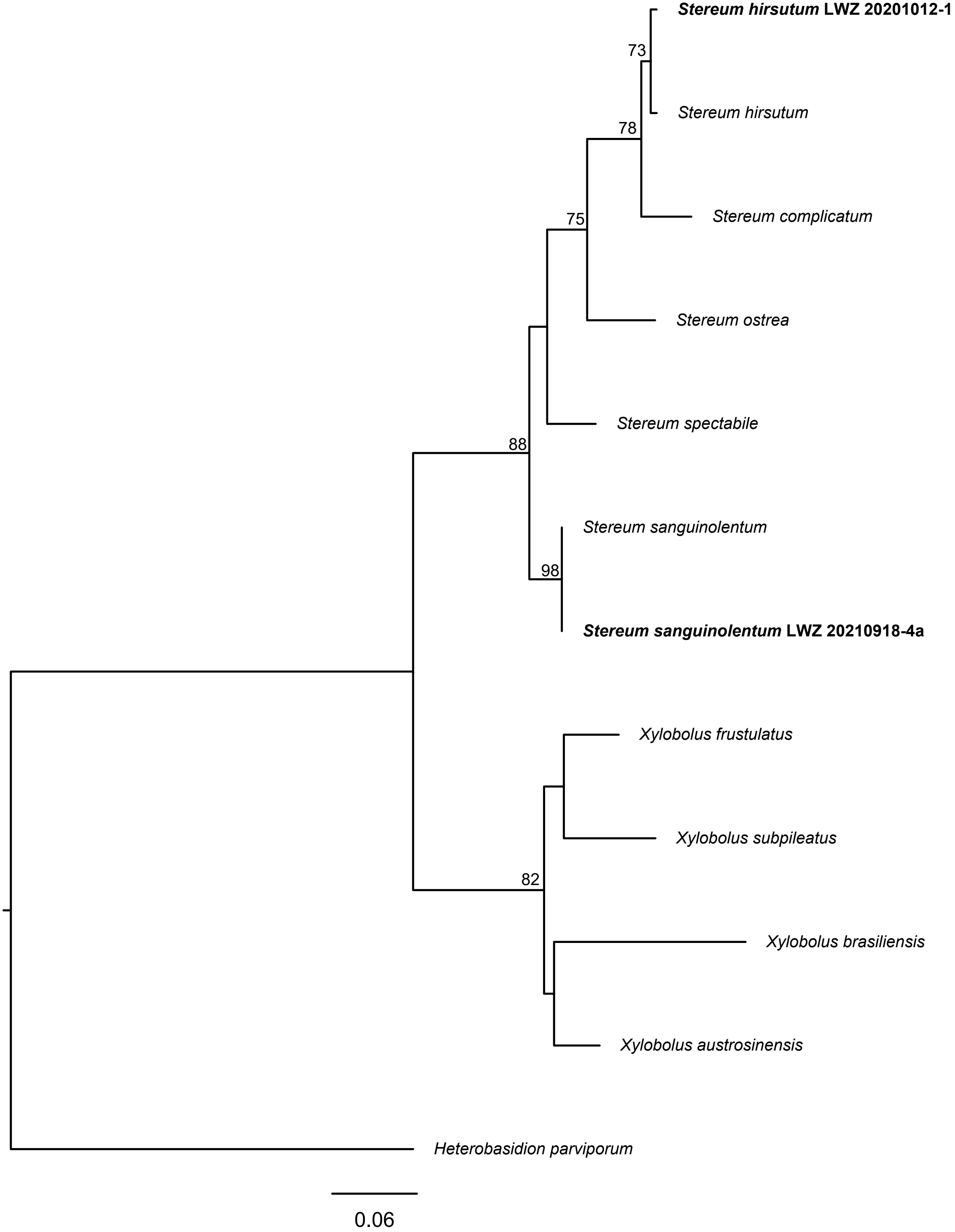

Stereum hirsutum LWZ 20201012-1
73
Stereum hirsutum
78
Stereum complicatum
75
Stereum ostrea
Stereum spectabile
88
Stereum sanguinolentum
98
Stereum sanguinolentum LWZ 20210918-4a
Xylobolus frustulatus
Xylobolus subpileatus
82
Xylobolus brasiliensis
Xylobolus austrosinensis
Heterobasidion parviporum
0.06

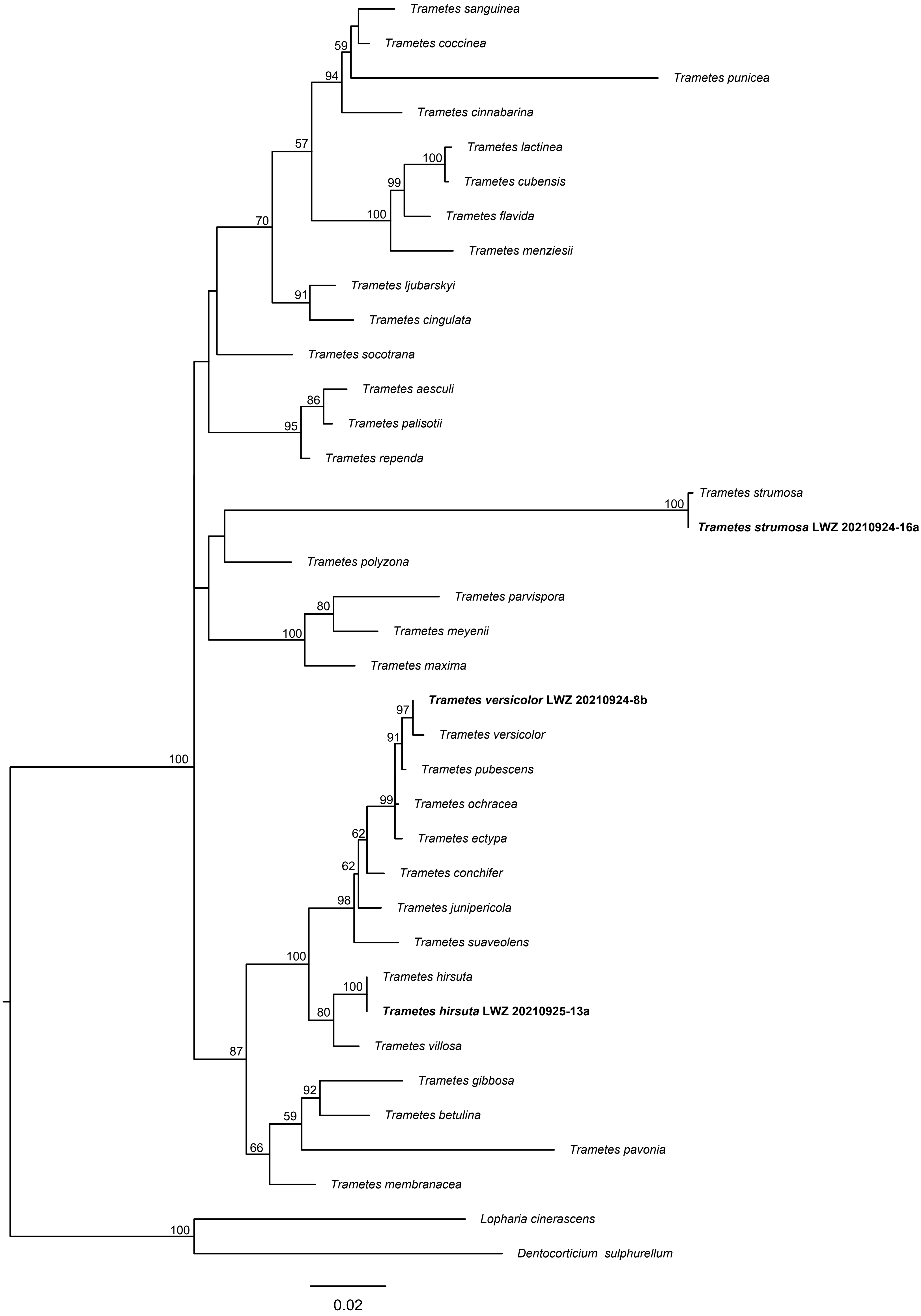
Trametes sanguinea
Trametes coccinea
Trametes punicea
Trametes cinnabarina
Trametes lactinea
Trametes cubensis
Trametes flavida
Trametes menziesii
Trametes ljubarskyi
Trametes cingulata
Trametes socotrana
Trametes aesculi
Trametes palisotii
Trametes rependa
Trametes strumosa
Trametes strumosa LWZ 20210924-16a
Trametes polyzona
Trametes parvispora
Trametes meyenii
Trametes maxima
Trametes versicolor LWZ 20210924-8b
Trametes versicolor
Trametes pubescens
Trametes ochracea
Trametes ectypa
Trametes conchifer
Trametes junipericola
Trametes suaveolens
Trametes hirsuta
Trametes hirsuta LWZ 20210925-13a
Trametes villosa
Trametes gibbosa
Trametes betulina
Trametes pavonia
Trametes membranacea
Lopharia cinerascens
Dentocorticium sulphurellum
0.02

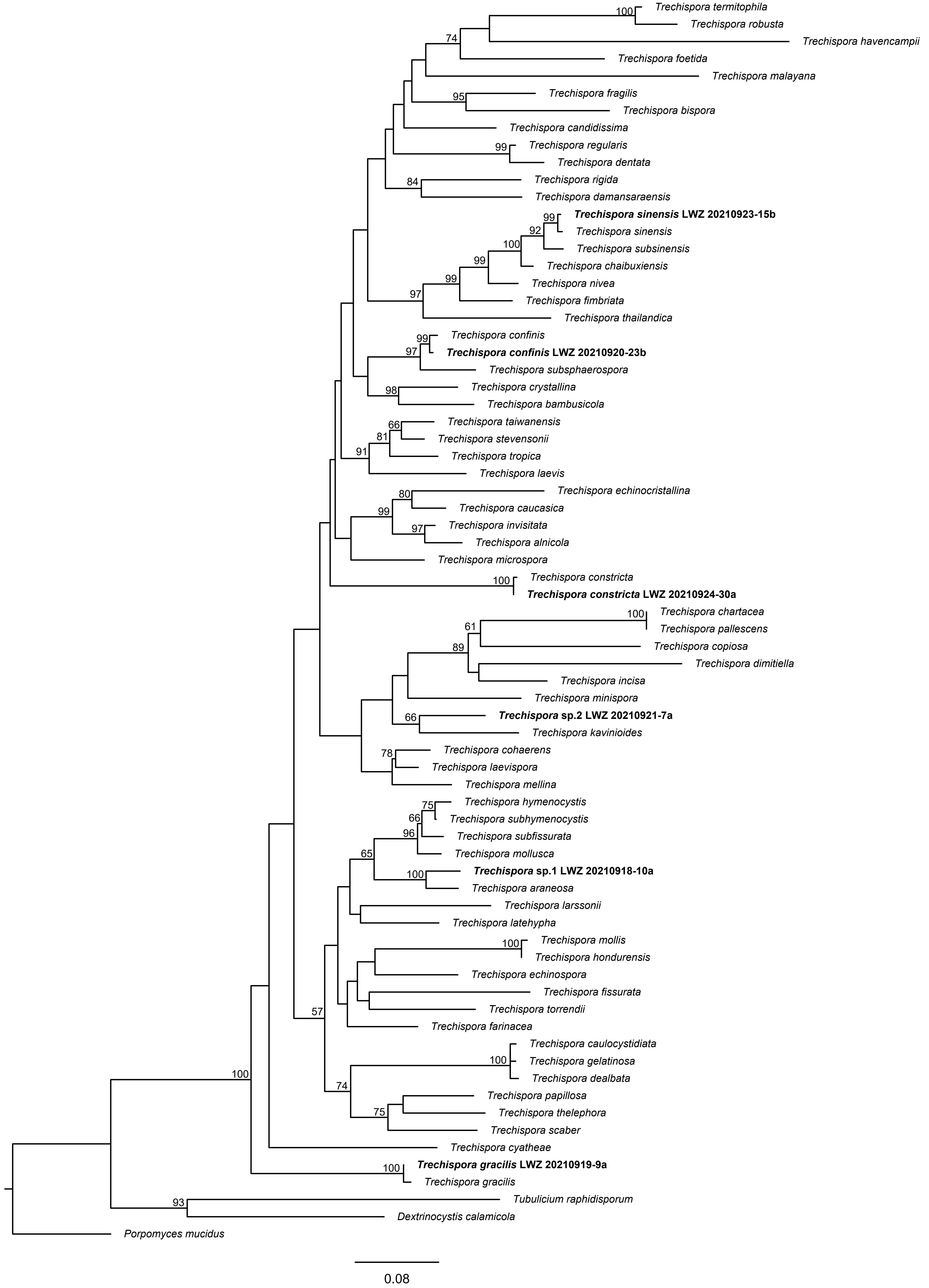

Trechispora termitophila
Trechispora robusta
Trechispora havencampii
Trechispora foetida
Trechispora malayana
Trechispora fragilis
Trechispora bispora
Trechispora candidissima
Trechispora regularis
Trechispora dentata
Trechispora rigida
Trechispora damansaraensis
Trechispora sinensis LWZ 20210923-15b
Trechispora sinensis
Trechispora subsinensis
Trechispora chaibuxiensis
Trechispora nivea
Trechispora fimbriata
Trechispora thailandica
Trechispora confinis
Trechispora confinis LWZ 20210920-23b
Trechispora subsphaerospora
Trechispora crystallina
Trechispora bambusicola
Trechispora taiwanensis
Trechispora stevensonii
Trechispora tropica
Trechispora laevis
Trechispora echinocristallina
Trechispora caucasica
Trechispora invisitata
Trechispora alnicola
Trechispora microspora
Trechispora constricta
Trechispora constricta LWZ 20210924-30a
Trechispora chartacea
Trechispora pallescens
Trechispora copiosa
Trechispora dimitiella
Trechispora incisa
Trechispora minispora
Trechispora sp.2 LWZ 20210921-7a
Trechispora kavinioides
Trechispora cohaerens
Trechispora laevispora
Trechispora mellina
Trechispora hymenocystis
Trechispora subhymenocystis
Trechispora subfissurata
Trechispora mollusca
Trechispora sp.1 LWZ 20210918-10a
Trechispora araneosa
Trechispora larssonii
Trechispora latehypha
Trechispora mollis
Trechispora hondurensis
Trechispora echinospora
Trechispora fissurata
Trechispora torrendii
Trechispora farinacea
Trechispora caulocystidiata
Trechispora gelatinosa
Trechispora dealbata
Trechispora papillosa
Trechispora thelephora
Trechispora scaber
Trechispora cyatheae
Trechispora gracilis LWZ 20210919-9a
Trechispora gracilis
Tubulicium raphidisporum
Dextrinocystis calamicola
Porpomyces mucidus
0.08

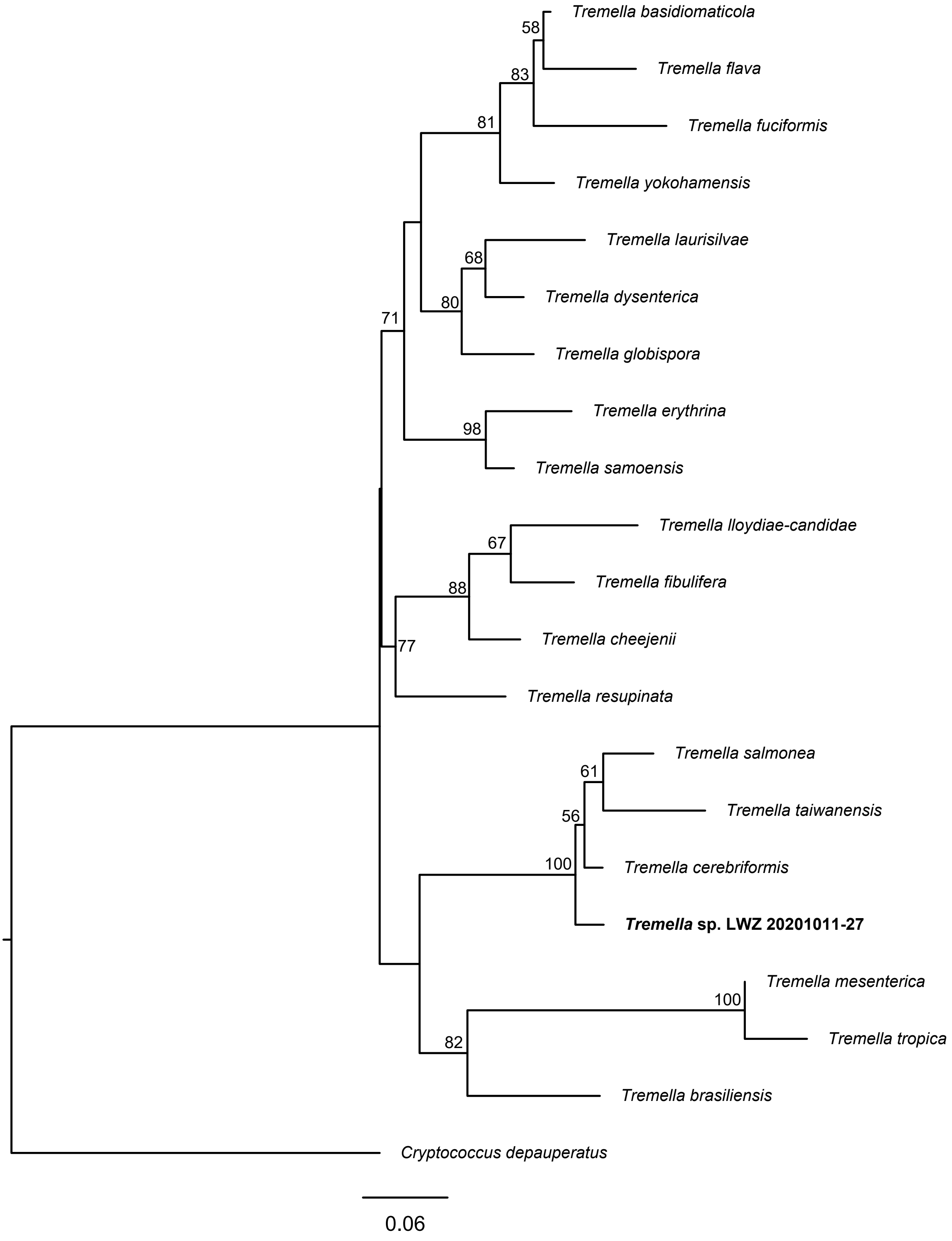

Tremella basidiomaticola
58
Tremella flava
83
Tremella fuciformis
81
Tremella yokohamensis
Tremella laurisilvae
68
Tremella dysenterica
80
71
Tremella globispora
Tremella erythrina
98
Tremella samoensis
Tremella lloydiae-candidae
67
Tremella fibulifera
88
Tremella cheejenii
77
Tremella resupinata
Tremella salmonea
61
Tremella taiwanensis
56
Tremella cerebriformis
100
Tremella sp. LWZ 20201011-27
Tremella mesenterica
100
Tremella tropica
82
Tremella brasiliensis
Cryptococcus depauperatus
0.06

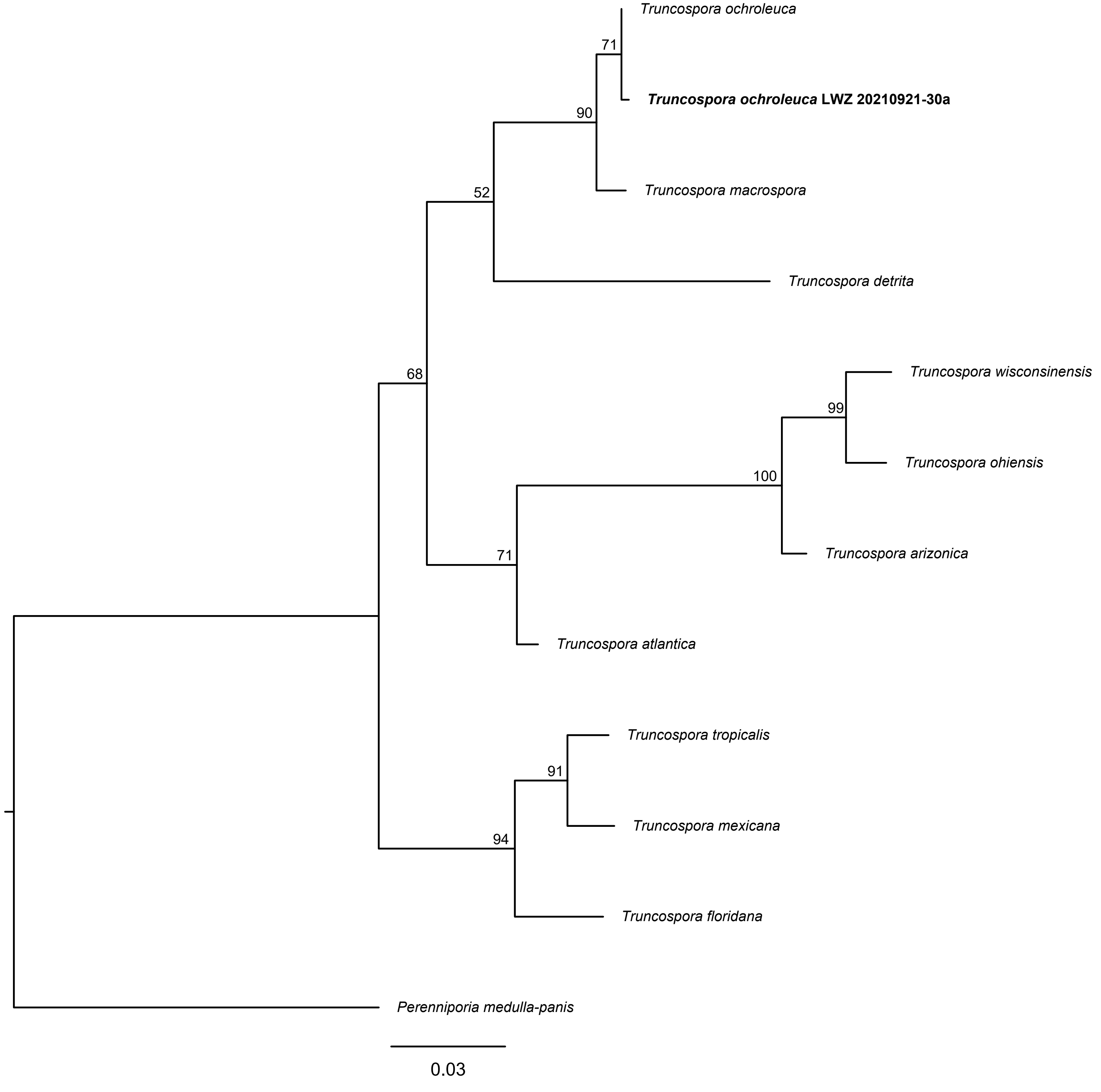
Truncospora ochroleuca
71
Truncospora ochroleuca LWZ 20210921-30a
90
Truncospora macrospora
52
Truncospora detrita
68
Truncospora wisconsinensis
99
Truncospora ohiensis
100
Truncospora arizonica
71
Truncospora atlantica
Truncospora tropicalis
91
Truncospora mexicana
94
Truncospora floridana
Perenniporia medulla-panis
0.03

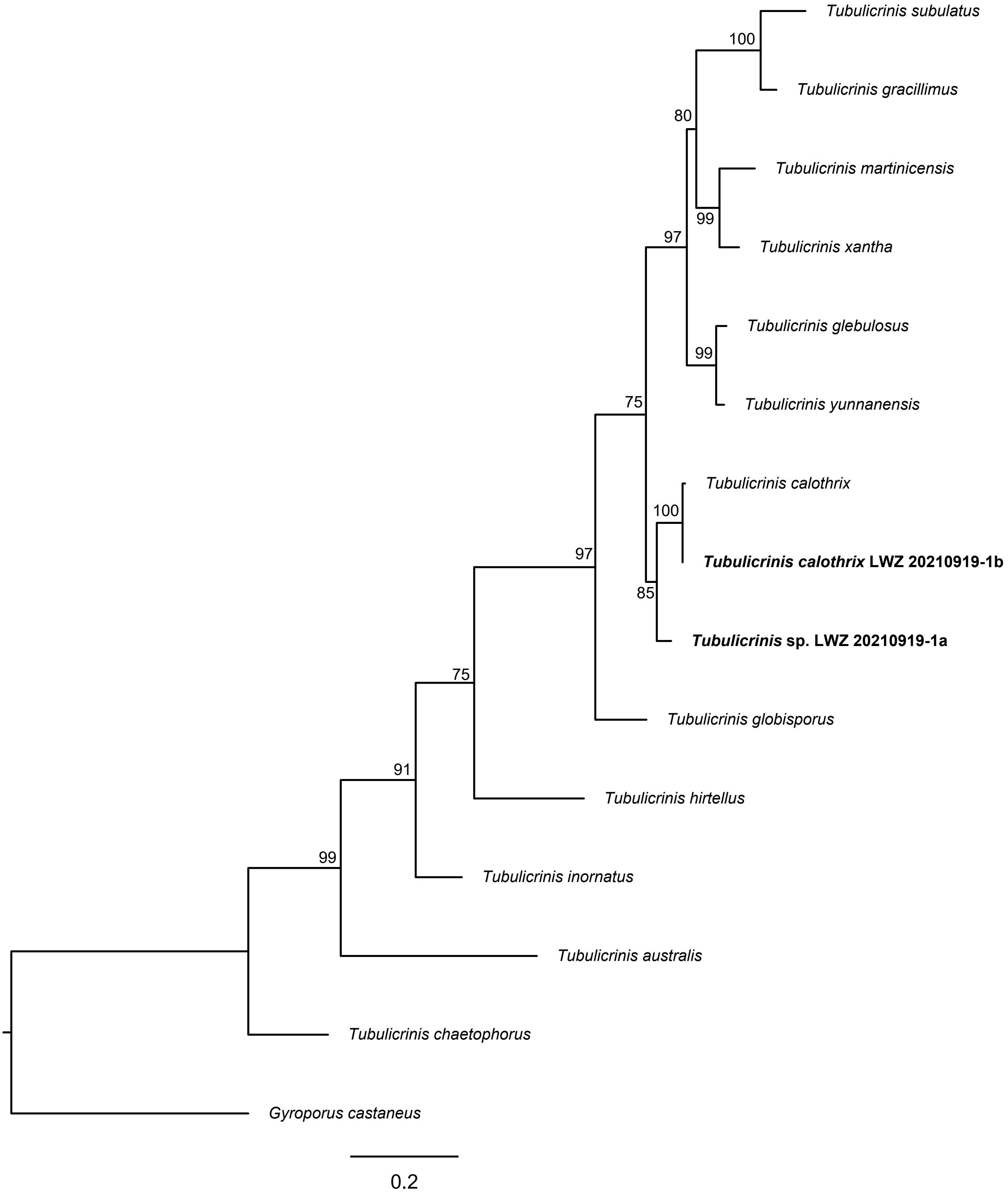
Tubulicrinis subulatus
Tubulicrinis gracillimus
Tubulicrinis martinicensis
Tubulicrinis xantha
Tubulicrinis glebulosus
Tubulicrinis yunnanensis
Tubulicrinis calothrix
Tubulicrinis calothrix LWZ 20210919-1b
Tubulicrinis sp. LWZ 20210919-1a
Tubulicrinis globisporus
Tubulicrinis hirtellus
Tubulicrinis inornatus
Tubulicrinis australis
Tubulicrinis chaetophorus
Gyroporus castaneus
100
80
99
97
99
75
100
85
97
75
91
99
0.2

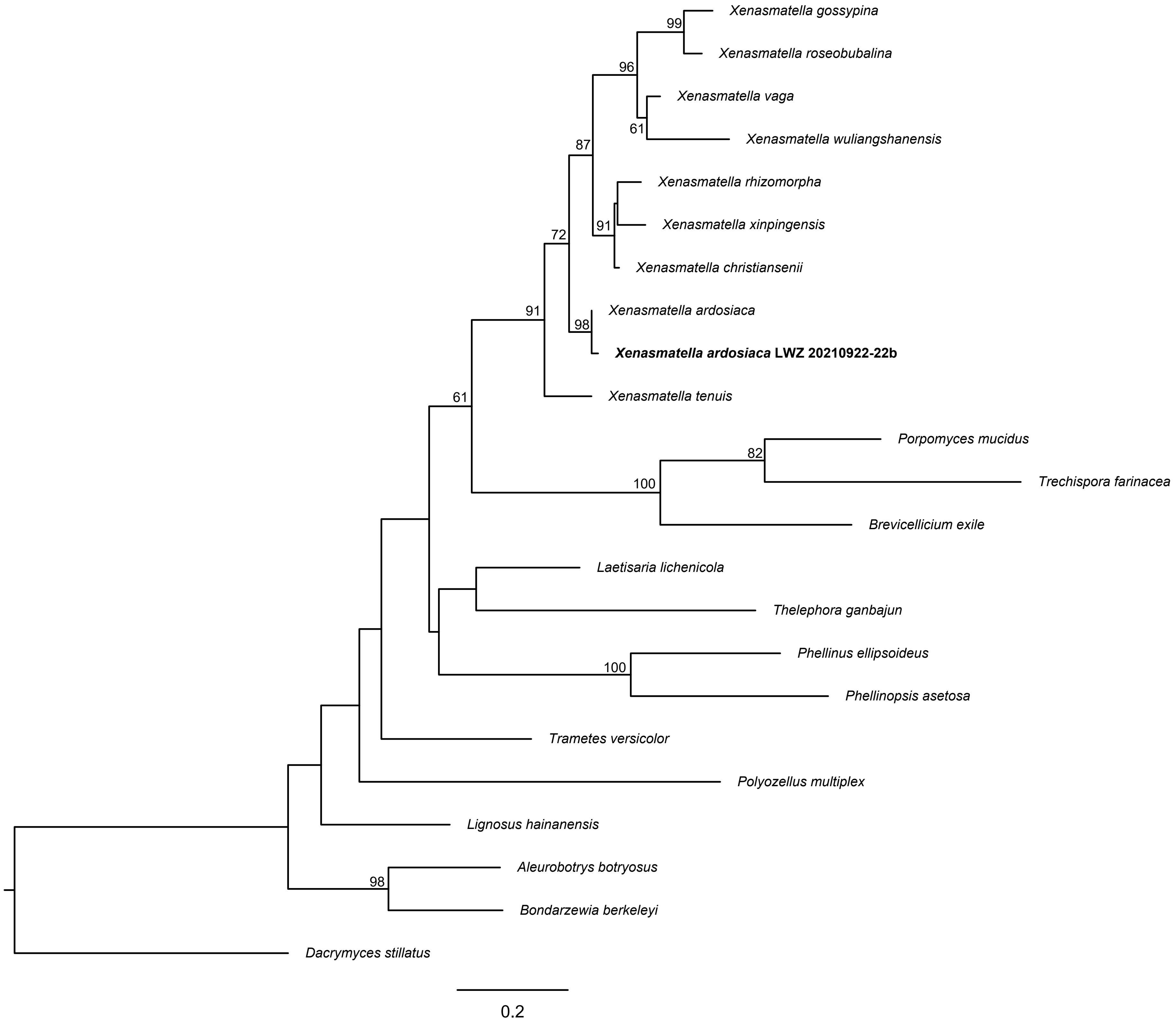
Xenasmatella gossypina
Xenasmatella roseobubalina
Xenasmatella vaga
Xenasmatella wuliangshanensis
Xenasmatella rhizomorpha
Xenasmatella xinpingensis
Xenasmatella christiansenii
Xenasmatella ardosiaca
Xenasmatella ardosiaca LWZ 20210922-22b
Xenasmatella tenuis
Porpomyces mucidus
Trechispora farinacea
Brevicellicium exile
Laetisaria lichenicola
Thelephora ganbajun
Phellinus ellipsoideus
Phellinopsis asetosa
Trametes versicolor
Polyozellus multiplex
Lignosus hainanensis
Aleurobotrys botryosus
Bondarzewia berkeleyi
Dacrymyces stillatus
99
96
61
87
91
72
98
91
61
82
100
100
98
0.2

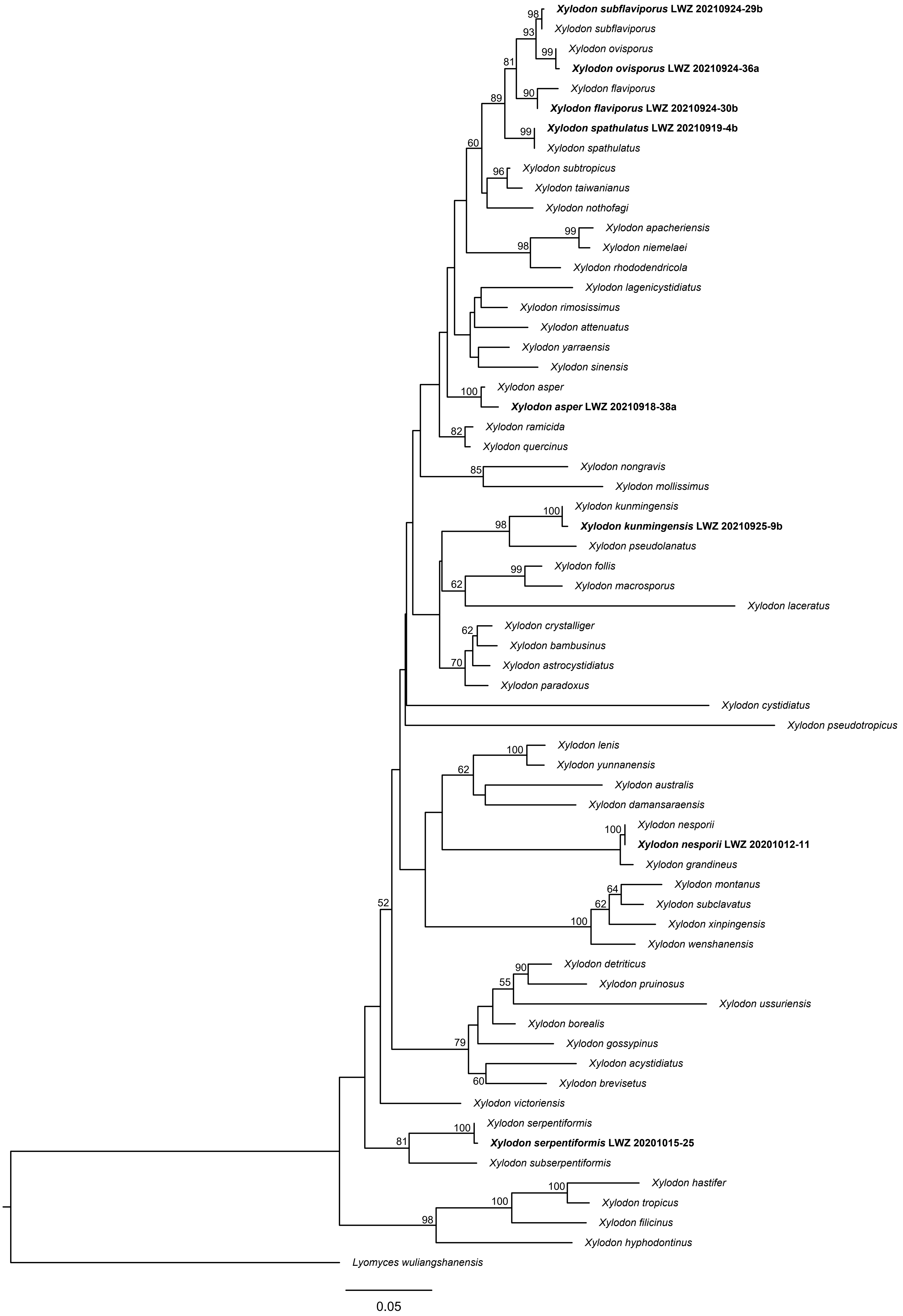
Xylodon subflaviporus LWZ 20210924-29b
Xylodon subflaviporus
Xylodon ovisporus
Xylodon ovisporus LWZ 20210924-36a
Xylodon flaviporus
Xylodon flaviporus LWZ 20210924-30b
Xylodon spathulatus LWZ 20210919-4b
Xylodon spathulatus
Xylodon subtropicus
Xylodon taiwanianus
Xylodon nothofagi
Xylodon apacheriensis
Xylodon niemelaei
Xylodon rhododendricola
Xylodon lagenicystidiatus
Xylodon rimosissimus
Xylodon attenuatus
Xylodon yarraensis
Xylodon sinensis
Xylodon asper
Xylodon asper LWZ 20210918-38a
Xylodon ramicida
Xylodon quercinus
Xylodon nongravis
Xylodon mollissimus
Xylodon kunmingensis
Xylodon kunmingensis LWZ 20210925-9b
Xylodon pseudolanatus
Xylodon follis
Xylodon macrosporus
Xylodon laceratus
Xylodon crystalliger
Xylodon bambusinus
Xylodon astrocystidiatus
Xylodon paradoxus
Xylodon cystidiatus
Xylodon pseudotropicus
Xylodon lenis
Xylodon yunnanensis
Xylodon australis
Xylodon damansaraensis
Xylodon nesporii
Xylodon nesporii LWZ 20201012-11
Xylodon grandineus
Xylodon montanus
Xylodon subclavatus
Xylodon xinpingensis
Xylodon wenshanensis
Xylodon detriticus
Xylodon pruinosus
Xylodon ussuriensis
Xylodon borealis
Xylodon gossypinus
Xylodon acystidiatus
Xylodon brevisetus
Xylodon victoriensis
Xylodon serpentiformis
Xylodon serpentiformis LWZ 20201015-25
Xylodon subserpentiformis
Xylodon hastifer
Xylodon tropicus
Xylodon filicinus
Xylodon hyphodontinus
Lyomyces wuliangshanensis
0.05
