# Supplementary material for: Preliminary species diversity and community phylogenetics of wood-inhabiting basidiomycetous fungi in the Dabie Mountains, Central China reveal unexpected richness
Source: IMA Fungus. 2023 Nov 14;14:23. doi: 10.1186/s43008-023-00130-9 (PMC10644440; doi:10.1186/s43008-023-00130-9)
Supplement: Supplementary file 4 — Additional file 4. An annotated checklist of wood-inhabiting basidiomycetous fungi in the Dabie Mountain. [file 43008_2023_130_MOESM4_ESM.docx]

Supplementary File 2. An annotated checklist of wood-inhabiting basidiomycetous fungi in the Dabie Mountain. The species are listed following alphabetical order of their taxonomic position from class downwards. The 12 edible species are indicated by dots (⦁), the 28 medicinal species by asterisks (*), the four poisonous species by hashtags (#), and the seven forest pathogens by diamonds (⬧).

*Agaricomycetes*

*Agaricales*

*Crepidotaceae*

*Crepidotus*

***Crepidotus crocophyllus***

Specimen examined: TM: on fallen trunk of angiosperm, 13 Oct. 2020, *LWZ 20201013-13* (HMAS 256328).

*Cyphellaceae*

*Chondrostereum*

**⬧*Chondrostereum purpureum***

Specimen examined: WFS: on fallen trunk of angiosperm, 17 Oct. 2020, *LWZ 20201017-51* (HMAS 256460).

*Gloeostereum*

***Gloeostereum* sp.**

Specimen examined: DBS: on fallen trunk of angiosperm, 20 Sept. 2021, *LWZ 20210920-5a* (HMAS 256611).

*Hymenogastraceae*

*Gymnopilus*

***#Gymnopilus penetrans***

Specimens examined: TM: on fallen trunk of angiosperm, 11 Oct. 2020, *LWZ 20201011-30* (HMAS 256268), on fallen trunk of angiosperm, 13 Oct. 2020, *LWZ 20201013-19* (HMAS 256333); WFS: on fallen trunk of angiosperm, 17 Oct. 2020, *LWZ 20201017-25* (HMAS 256441).

*Mycenaceae*

*Mycena*

**⦁**Mycena galericulata***

Specimen examined: WFS: on fallen trunk of angiosperm, 17 Oct. 2020, *LWZ 20201017-57* (HMAS 256465).

***Mycena maculata***

Specimen examined: YLP: on stump of angiosperm, 14 Oct. 2020, *LWZ 20201014-33* (HMAS 256362).

*Panellus*

***Panellus stipticus***

Specimens examined: YLP: on fallen trunk of angiosperm, 15 Oct. 2020, *LWZ 20201015-14* (HMAS 256384), on fallen trunk of *Pinus*, 14 Oct. 2020, *LWZ 20201014-42* (HMAS 256370).

*Phyllotopsidaceae*

*Phyllotopsis*

**⦁*Phyllotopsis nidulans***

Specimens examined: YLP: on fallen trunk of angiosperm, 14 Oct. 2020, *LWZ 20201014-5* (HMAS 256337), on fallen trunk of angiosperm, 15 Oct. 2020, *LWZ 20201015-24* (HMAS 256393).

*Physalacriaceae*

*Armillaria*

**⦁**Armillaria gallica***

Specimen examined: TM: on fallen trunk of angiosperm, 11 Oct. 2020, *LWZ 20201011-36* (HMAS 256272), on stump of angiosperm, 12 Oct. 2020, *LWZ 20201012-25* (HMAS 256306), on stump of angiosperm, 13 Oct. 2020, *LWZ 20201013-6* (HMAS 256322); WFS: on fallen trunk of angiosperm, 17 Oct. 2020, *LWZ 20201017-47* (HMAS 256458).

**⦁**Armillaria mellea***

Specimens examined: YLP: on fallen trunk of *Quercus*, 14 Oct. 2020, *LWZ 20201014-43* (HMAS 256371); WFS: on stump of angiosperm, 17 Oct. 2020, *LWZ 20201017-35* (HMAS 256451).

*Cylindrobasidium*

***Cylindrobasidium laeve***

Specimens examined: TM: on fallen trunk of *Ailanthus*, 11 Oct. 2020, *LWZ 20201011-9* (HMAS 256249); YLP: on fallen trunk of angiosperm, 14 Oct. 2020, *LWZ 20201014-36* (HMAS 256365), on fallen trunk of angiosperm, 14 Oct. 2020, *LWZ 20201014-38* (HMAS 256367); WFS: on fallen trunk of angiosperm, 16 Oct. 2020, *LWZ 20201016-23* (HMAS 256417), on fallen branch of angiosperm, 17 Oct. 2020, *LWZ 20201017-19* (HMAS 256436), on fallen branch of *Pinus*, 17 Oct. 2020, *LWZ 20201017-29* (HMAS 256445), on fallen trunk of angiosperm, 17 Oct. 2020, *LWZ 20201017-37* (HMAS 256452).

*Pleurotaceae*

*Pleurotus*

***Pleurotus columbinus***

Specimen examined: WFS: on fallen trunk of angiosperm, 16 Oct. 2020, *LWZ 20201016-21* (HMAS 256415).

**⦁**Pleurotus pulmonarius***

Specimens examined: YLP: on dead angiosperm, 14 Oct. 2020, *LWZ 20201014-16* (HMAS 256347); WFS: on fallen trunk of angiosperm, 16 Oct. 2020, *LWZ 20201016-16* (HMAS 256412), on fallen trunk of angiosperm, 17 Oct. 2020, *LWZ 20201017-46* (HMAS 256457).

*Radulomycetaceae*

*Radulomyces*

***Radulomyces confluens***

Specimens examined: TM: on fallen trunk of *Caprifoliaceae*, 11 Oct. 2020, *LWZ 20201011-7* (HMAS 256247), on fallen trunk of angiosperm, 12 Oct. 2020, *LWZ 20201012-26* (HMAS 256307); WNS: on fallen branch of angiosperm, 21 Sept. 2021, *LWZ 20210921-17a* (HMAS 256678).

***Radulomyces paumanokensis***

Specimens examined: YLP: on fallen trunk of angiosperm, 14 Oct. 2020, *LWZ 20201014-10* (HMAS 256342), on a cone of *Pinus*, 14 Oct. 2020, *LWZ 20201014-40* (HMAS 256369); WFS: on fallen trunk of *Quercus*, 17 Oct. 2020, *LWZ 20201017-8* (HMAS 256425); DBS: on dead angiosperm, 19 Sept. 2021, *LWZ 20210919-28a* (HMAS 256575).

*Schizophyllaceae*

*Schizophyllum*

**⦁*⬧*Schizophyllum commune***

Specimens examined: WFS: on fallen branch of angiosperm, 17 Oct. 2020, *LWZ 20201017-28* (HMAS 256444), on fallen branch of angiosperm, 17 Oct. 2020, *LWZ 20201017-60* (HMAS 256468); WNS: on fallen branch of angiosperm, 21 Sept. 2021, *LWZ 20210921-16a* (HMAS 256676).

*Strophariaceae*

*Hypholoma*

***#*Hypholoma fasciculare***

Specimens examined: WFS: on fallen trunk of angiosperm, 16 Oct. 2020, *LWZ 20201016-4* (HMAS 256402), on fallen trunk of angiosperm, 17 Oct. 2020, *LWZ 20201017-59* (HMAS 256467).

***#Hypholoma lateritium***

Specimens examined: TM: on fallen trunk of angiosperm, 11 Oct. 2020, *LWZ 20201011-46* (HMAS 256282), on stump of angiosperm, 11 Oct. 2020, *LWZ 20201011-49* (HMAS 256285), on stump of *Quercus*, Oct 12 2020, *LWZ 20201012-3* (HMAS 256291), on stump of angiosperm, 12 Oct. 2020, *LWZ 20201012-31* (HMAS 256310); YLP: on stump of angiosperm, 14 Oct. 2020, *LWZ 20201014-8* (HMAS 256340), on stump of angiosperm, 14 Oct. 2020, *LWZ 20201014-9* (HMAS 256341), on stump of angiosperm, 14 Oct. 2020, *LWZ 20201014-28* (HMAS 256358), on stump of angiosperm, 15 Oct. 2020, *LWZ 20201015-7* (HMAS 256377).

*Pholiota*

***Pholiota limonella***

Specimens examined: TM: on fallen trunk of angiosperm, 11 Oct. 2020, *LWZ 20201011-16* (HMAS 256255), on fallen trunk of angiosperm, 12 Oct. 2020, *LWZ 20201012-24* (HMAS 256305), on dead angiosperm, 13 Oct. 2020, *LWZ 20201013-18* (HMAS 256332); WFS: on fallen trunk of angiosperm, 17 Oct. 2020, *LWZ 20201017-52* (HMAS 256461).

*Stropharia*

**⦁*Stropharia aeruginosa***

Specimens examined: TM: on fallen trunk of angiosperm, 11 Oct. 2020, *LWZ 20201011-48* (HMAS 256284), on fallen trunk of angiosperm, 12 Oct. 2020, *LWZ 20201012-37* (HMAS 256313), on fallen trunk of angiosperm, 12 Oct. 2020, *LWZ 20201012-33* (HMAS 256312).

***Stropharia* sp.**

Specimens examined: TM, on stump of *Pinus*, 11 Oct. 2020, *LWZ 20201011-39* (HMAS 256275); THC: on stump of *Pinus*, 18 Sept. 2021, *LWZ 20210918-20b* (HMAS 256514).

*Amylocorticiales*

*Amylocorticiaceae*

*Anomoloma*

***Anomoloma luteoalbum***

Specimen examined: THC: on fallen branch of angiosperm, 18 Sept. 2021, *LWZ 20210918-39a* (HMAS 256344).

*Irpicodon*

***Irpicodon pendulus***

Specimens examined: WFS: on fallen trunk of *Pinus*, 16 Oct. 2020, *LWZ 20201016-2* (HMAS 256401), on dead *Pinus*, 17 Oct. 2020, *LWZ 20201017-13* (HMAS 256430); WNS: on dead angiosperm, 21 Sept. 2021, *LWZ 20210921-43a* (HMAS 256708); JLS: on stump of *Pinus*, 24 Sept. 2021, *LWZ 20210924-31a* (HMAS 256826); JGS: on dead angiosperm, 25 Sept. 2021, *LWZ 20210925-8b* (HMAS 256843).

*Plicaturopsis*

***Plicaturopsis crispa***

Specimens examined: TM: on fallen trunk of angiosperm, 11 Oct. 2020, *LWZ 20201011-12* (HMAS 256252); YLP: on fallen trunk of angiosperm, 15 Oct. 2020, *LWZ 20201015-30* (HMAS 256398); WFS: on fallen branch of angiosperm, 17 Oct. 2020, *LWZ 20201017-11* (HMAS 256428), on fallen branch of angiosperm, 17 Oct. 2020, *LWZ 20201017-44* (HMAS 256455), on fallen trunk of angiosperm, 17 Oct. 2020, *LWZ 20201017-56* (HMAS 256464).

*Atheliales*

*Atheliaceae*

*Athelia*

***Athelia* sp.**

Specimen examined: TM: on fallen trunk of *Pinus*, 12 Oct. 2020, *LWZ 20201012-13* (HMAS 256297).

*Amphinema*

***Amphinema* sp.**

Specimen examined: THC: on fallen trunk of angiosperm, 18 Sept. 2021, *LWZ 20210918-41a* (HMAS 256532).

*Piloderma*

***Piloderma* sp.**

Specimen examined: DBS: on base of angiosperm, 19 Sept. 2021, *LWZ 20210919-39b* (HMAS 256595).

*Auriculariales*

*Auriculariaceae*

*Aporpium*

***Aporpium caryae***

Specimen examined: THC: on fallen trunk of *Pinus*, 18 Sept. 2021, *LWZ 20210918-21b* (HMAS 256516).

*Auricularia*

**⦁**Auricularia cornea***

Specimen examined: TM: on fallen trunk of angiosperm, 13 Oct. 2020, *LWZ 20201013-16* (HMAS 256331).

*Eichleriella*

***Eichleriella aculeobasidiata***

Specimens examined: YLP: on dead *Quercus*, 14 Oct. 2020, *LWZ 20201014-15* (HMAS 256346); WFS: on fallen branch of angiosperm, 16 Oct. 2020, *LWZ 20201016-20* (HMAS 256414), on fallen trunk of angiosperm, 17 Oct. 2020, *LWZ 20201017-67* (HMAS 256475); THC: on fallen trunk of angiosperm, 18 Sept. 2021, *LWZ 20210918-21a* (HMAS 256515); DBS: on fallen branch of *Pinus*, 19 Sept. 2021, *LWZ 20210919-24a* (HMAS 256568), on fallen branch of angiosperm, 19 Sept. 2021, *LWZ 20210919-29b* (HMAS 256577), on fallen branch of angiosperm, 20 Sept. 2021, *LWZ 20210920-10a* (HMAS 256619), on fallen branch of angiosperm, 20 Sept. 2021, *LWZ 20210920-17b* (HMAS 256632); JGS: on fallen twig of angiosperm, 25 Sept. 2021, *LWZ 20210925-11a* (HMAS 256845), on fallen branch of angiosperm, 25 Sept. 2021, *LWZ 20210925-19a* (HMAS 256856), on fallen branch of angiosperm, 25 Sept. 2021, *LWZ 20210925-23a* (HMAS 256859).

***Eichleriella* sp.**

Specimens examined: THC: on fallen branch of angiosperm, 18 Sept. 2021, *LWZ 20210918-23a* (HMAS 256518); DBS: on fallen branch of angiosperm, 20 Sept. 2021, *LWZ 20210920-15b* (HMAS 256628).

*Exidia*

**⦁#*Exidia glandulosa***

Specimens examined: WFS: on fallen trunk of angiosperm, 17 Oct. 2020, *LWZ 20201017-65* (HMAS 256473), on fallen trunk of angiosperm, 17 Oct. 2020, *LWZ 20201017-66* (HMAS 256474).

*Boletales*

*Coniophoraceae*

*Coniophora*

***Coniophora arida***

Specimen examined: DBS: on fallen trunk of *Pinus*, 19 Sept. 2021, *LWZ 20210919-16a* (HMAS 256555).

*Corticiales*

*Corticiaceae*

*Corticium*

***Corticium minnsiae***

Specimen examined: DBS: on fallen trunk of *Pinus*, 20 Sept. 2021, *LWZ 20210920-14a* (HMAS 256625).

*Punctulariaceae*

*Punctularia*

***Punctularia bambusicola***

Specimen examined: SZF: on stump of bamboo, 22 Sept. 2021, *LWZ 20210922-4a* (HMAS 256713).

*Vuilleminiaceae*

***Vuilleminia comedens***

Specimens examined: TM: on fallen trunk of *Quercus*, 11 Oct. 2020, *LWZ 20201011-3* (HMAS 256243); YLP: on fallen trunk of angiosperm, 14 Oct. 2020, *LWZ 20201014-23* (HMAS 256353); THC: on fallen branch of angiosperm, 18 Sept. 2021, *LWZ 20210918-3a* (HMAS 256480); DBS: on fallen branch of angiosperm, 19 Sept. 2021, *LWZ 20210919-10a* (HMAS 256546), on fallen twig of angiosperm, 19 Sept. 2021, *LWZ 20210919-23b* (HMAS 256567), on fallen twig of angiosperm, 19 Sept. 2021, *LWZ 20210919-24b* (HMAS 256569), on fallen twig of angiosperm, 19 Sept. 2021, *LWZ 20210919-26b* (HMAS 256573), on fallen branch of angiosperm, 19 Sept. 2021, *LWZ 20210919-38a* (HMAS 256592), on fallen twig of angiosperm, 19 Sept. 2021, *LWZ 20210919-40b* (HMAS 256596).

*Hymenochaetales*

*Hymenochaetaceae*

*Fomitiporia*

***⬧*Fomitiporia torreyae***

Specimen examined: JLS: on branch of living angiosperm, 24 Sept. 2021, *LWZ 20210924-1b* (HMAS 256777).

*Fuscoporia*

****Fuscoporia gilva***

Specimens examined: TM: on fallen trunk of angiosperm, 11 Oct. 2020, *LWZ 20201011-21* (HMAS 256260), on fallen trunk of angiosperm, 11 Oct. 2020, *LWZ 20201011-25* (HMAS 256264); WNS: on fallen branch of angiosperm, 21 Sept. 2021, *LWZ 20210921-1b* (HMAS 256654), on fallen branch of angiosperm, 21 Sept. 2021, *LWZ 20210921-4b* (HMAS 256657), on base of angiosperm, 21 Sept. 2021, *LWZ 20210921-24b* (HMAS 256688), on fallen trunk of angiosperm, 21 Sept. 2021, *LWZ 20210921-41a* (HMAS 256706); SZF: on fallen trunk of angiosperm, 22 Sept. 2021, *LWZ 20210922-12b* (HMAS 256721), on fallen trunk of angiosperm, 22 Sept. 2021, *LWZ 20210922-20b* (HMAS 256730); JLS: on dead angiosperm, 24 Sept. 2021, *LWZ 20210924-12b* (HMAS 256793).

*Hydnoporia*

***Hydnoporia yasudae***

Specimen examined: SZF: on fallen twig of angiosperm, 22 Sept. 2021, *LWZ 20210922-22a* (HMAS 256731); JLS: on fallen twig of angiosperm, 24 Sept. 2021, *LWZ 20210924-26a* (HMAS 256818).

***Hydnoporia*** **sp.**

Specimen examined: TM: on fallen branch of angiosperm, 12 Oct. 2020, *LWZ 20201012-41* (HMAS 256316); THC: on fallen trunk of angiosperm, 18 Sept. 2021, *LWZ 20210918-27a* (HMAS 256521).

*Hymenochaete*

***Hymenochaete huangshanensis***

Specimens examined: TM: on fallen branch of angiosperm, 12 Oct. 2020, *LWZ 20201012-17* (HMAS 256299); YLP: on fallen branch of angiosperm, 14 Oct. 2020, *LWZ 20201014-27* (HMAS 256357), on fallen branch of angiosperm, 15 Oct. 2020, *LWZ 20201015-10* (HMAS 256380).

***Hymenochaete rubiginosa***

Specimens examined: WFS: on dead angiosperm, 17 Oct. 2020, *LWZ 20201017-32* (HMAS 256448), on fallen trunk of angiosperm, 17 Oct. 2020, *LWZ 20201017-39* (HMAS 256454).

***Hymenochaete separabilis***

Specimen examined: YLP: on fallen branch of angiosperm, 14 Oct. 2020, *LWZ 20201014-25* (HMAS 256355).

***Hymenochaete yunnanensis***

Specimen examined: DBS: on fallen branch of angiosperm, 19 Sept. 2021, *LWZ 20210919-12b* (HMAS 256550).

***Hymenochaete xerantica***

Specimen examined: TM: on stump of angiosperm, 11 Oct. 2020, *LWZ 20201011-51* (HMAS 256287).

*Tubulicrinis*

***Tubulicrinis calothrix***

Specimen examined: DBS: on fallen twig of angiosperm, 19 Sept. 2021, *LWZ 20210919-1b* (HMAS 256534).

***Tubulicrinis* sp.**

Specimen examined: DBS: on fallen branch of *Pinus*, 19 Sept. 2021, *LWZ 20210919-1a* (HMAS 256533).

*Hyphodontiaceae*

*Hyphodontia*

***Hyphodontia zhixiangii***

Specimen examined: TM: on fallen trunk of angiosperm, 13 Oct. 2020, *LWZ 20201013-14* (HMAS 256329).

*Oxyporaceae*

*Rigidoporus*

***Rigidoporus cuneatus***

Specimens examined: WFS: on fallen trunk of angiosperm, 17 Oct. 2020, *LWZ 20201017-34* (HMAS 256450); THC: on dead *Pinus*, 18 Sept. 2021, *LWZ 20210918-9b* (HMAS 256493), on fallen twig of *Pinus*, 18 Sept. 2021, *LWZ 20210918-12b* (HMAS 256499), on fallen branch of angiosperm, 18 Sept. 2021, *LWZ 20210918-13a* (HMAS 256500), on dead angiosperm, 18 Sept. 2021, *LWZ 20210918-14a* (HMAS 256502).

***Rigidoporus ginkgonis***

Specimen examined: SZF: on stump of *Picea*, 22 Sept. 2021, *LWZ 20210922-12a* (HMAS 256720).

***Rigidoporus obducens***

Specimen examined: HNDBS: on fallen branch of angiosperm, 23 Sept. 2021, *LWZ 20210923-7a* (HMAS 256748).

*Schizoporaceae*

*Basidioradulum*

***Basidioradulum radula***

Specimen examined: WFS: on fallen branch of *Pinus*, 17 Oct. 2020, *LWZ 20201017-49* (HMAS 256459), on dead angiosperm, 17 Oct. 2020, *LWZ 20201017-62* (HMAS 256470); DBS: on fallen branch of angiosperm, 20 Sept. 2021, *LWZ 20210920-7a* (HMAS 256615).

*Lyomyces*

***Lyomyces crustosus***

Specimen examined: DBS: on fallen twig of angiosperm, 20 Sept. 2021, *LWZ 20210920-20b* (HMAS 256638).

***Lyomyces microfasciculatus***

Specimen examined: HNDBS: on fallen twig of angiosperm, 23 Sept. 2021, *LWZ 20210923-4b* (HMAS 256745).

*Poriodontia*

***Poriodontia* sp.**

Specimen examined: DBS: on fallen branch of *Picea*, 20 Sept. 2021, *LWZ 20210920-25b* (HMAS 256647).

*Xylodon*

***Xylodon asper***

Specimens examined: TM: on fallen trunk of *Pinus*, 11 Oct. 2020, *LWZ 20201011-38* (HMAS 256274), on stump of *Pinus*, 11 Oct. 2020, *LWZ 20201011-40* (HMAS 256276), on fallen trunk of *Pinus*, 12 Oct. 2020, *LWZ 20201012-14* (HMAS 256298), on fallen trunk of *Pinus*, 12 Oct. 2020, *LWZ 20201012-19* (HMAS 256301), on fallen trunk of *Pinus*, 12 Oct. 2020, *LWZ 20201012-20* (HMAS 256302); YLP: on fallen trunk of *Pinus*, 14 Oct. 2020, *LWZ 20201014-24* (HMAS 256354), on fallen trunk of *Pinus*, 15 Oct. 2020, *LWZ 20201015-21* (HMAS 256390); WFS: on stump of *Pinus*, 16 Oct. 2020, *LWZ 20201016-24* (HMAS 256418), on fallen trunk of angiosperm, 17 Oct. 2020, *LWZ 20201017-64* (HMAS 256472); THC: on stump of *Pinus*, 18 Sept. 2021, *LWZ 20210918-5a*, on fallen branch of *Picea*, 18 Sept. 2021, *LWZ 20210918-38a* (HMAS 256529).

***Xylodon flaviporus***

Specimens examined: THC: on fallen branch of *Pinus*, 18 Sept. 2021, *LWZ 20210918-16a* (HMAS 256506), on fallen twig of *Pinus*, 18 Sept 2021, *LWZ 20210918-18b* (HMAS 256510), on fallen trunk of angiosperm, 18 Sept. 2021, *LWZ 20210918-22a* (HMAS 256517), on fallen branch of *Pinus*, 18 Sept. 2021, *LWZ 20210918-28a* (HMAS 256522); DBS: on fallen twig of angiosperm, 19 Sept. 2021, *LWZ 20210919-8b* (HMAS 256543), on base of angiosperm, 19 Sept. 2021, *LWZ 20210919-22a* (HMAS 256564), on fallen trunk of angiosperm, 19 Sept. 2021, *LWZ 20210919-25a* (HMAS 256570), on fallen trunk of *Pinus*, 19 Sept. 2021, *LWZ 20210919-34a* (HMAS 256585), on fallen branch of *Pinus*, 19 Sept. 2021, *LWZ 20210919-35a* (HMAS 256587), on fallen branch of angiosperm, 19 Sept. 2021, *LWZ 20210919-35b* (HMAS 256588), on fallen branch of *Quercus*, 19 Sept. 2021, *LWZ 20210919-47a* (HMAS 256603); WNS: on fallen branch of angiosperm, 21 Sept. 2021, *LWZ 20210921-9a* (HMAS 256665), on fallen branch of angiosperm, 21 Sept. 2021, *LWZ 20210921-22b* (HMAS 256685), on fallen trunk of angiosperm, 21 Sept. 2021, *LWZ 20210921-28a* (HMAS 256691), on fallen twig of angiosperm, 21 Sept. 2021, *LWZ 20210921-28b* (HMAS 256692); SZF: on fallen twig of angiosperm, 22 Sept. 2021, *LWZ 20210922-24a* (HMAS 256735), on fallen twig of angiosperm, 22 Sept. 2021, *LWZ 20210922-29a* (HMAS 256739); JLS: on fallen twig of angiosperm, 24 Sept. 2021, *LWZ 20210924-8a* (HMAS 256785), on dead angiosperm, 24 Sept. 2021, *LWZ 20210924-11a* (HMAS 256790), on fallen twig of angiosperm, 24 Sept. 2021, *LWZ 20210924-14b* (HMAS 256796), on fallen twig of angiosperm, 24 Sept. 2021, *LWZ 20210924-23a* (HMAS 256812), on fallen branch of angiosperm, 24 Sept. 2021, *LWZ 20210924-30b* (HMAS 256825).

***Xylodon kunmingensis***

Specimens examined: TM: on fallen branch of angiosperm, 11 Oct. 2020, *LWZ 20201011-19* (HMAS 256258); YLP: on fallen branch of angiosperm, 15 Oct. 2020, *LWZ 20201015-8* (HMAS 256378); THC: on fallen branch of angiosperm, 18 Sept. 2021, *LWZ 20210918-34a* (HMAS 256526); DBS: on fallen twig of angiosperm, 19 Sept. 2021, *LWZ 20210919-7b* (HMAS 256542), on fallen branch of angiosperm, 20 Sept. 2021, *LWZ 20210920-24a* (HMAS 256645); WNS: on fallen branch of angiosperm, 21 Sept. 2021, *LWZ 20210921-29b* (HMAS 256694), on fallen branch of angiosperm, 21 Sept. 2021, *LWZ 20210921-31b* (HMAS 256698), on fallen twig of angiosperm, 21 Sept. 2021, *LWZ 20210921-42a* (HMAS 256707); HNDBS: on fallen twig of angiosperm, 23 Sept. 2021, *LWZ 20210923-1b* (HMAS 256740), on fallen twig of angiosperm, 23 Sept. 2021, *LWZ 20210923-3b* (HMAS 256743), on fallen twig of *Pinus*, 23 Sept. 2021, *LWZ 20210923-10a* (HMAS 256750), on fallen twig of angiosperm, 23 Sept. 2021, *LWZ 20210923-11b* (HMAS 256753), on fallen twig of angiosperm, 23 Sept. 2021, *LWZ 20210923-12a* (HMAS 256754), on fallen twig of angiosperm, 23 Sept. 2021, *LWZ 20210923-18a* (HMAS 256760), on fallen twig of angiosperm, 23 Sept. 2021, *LWZ 20210923-21a* (HMAS 256766); JLS: on fallen trunk of angiosperm, 24 Sept. 2021, *LWZ 20210924-1a* (HMAS 256776), on fallen twig of *Pinus*, 24 Sept. 2021, *LWZ 20210924-20a* (HMAS 256807), on fallen twig of angiosperm, 24 Sept. 2021, *LWZ 20210924-21a* (HMAS 256809), on fallen twig of angiosperm, 24 Sept. 2021, *LWZ 20210924-22a* (HMAS 256810), on fallen branch of angiosperm, 24 Sept. 2021, *LWZ 20210924-24b* (HMAS 256815), on fallen twig of angiosperm, 24 Sept. 2021, *LWZ 20210924-25a* (HMAS 256816), on fallen branch of angiosperm, 24 Sept. 2021, *LWZ 20210924-25b* (HMAS 256817), on fallen twig of angiosperm, 24 Sept. 2021, *LWZ 20210924-28b* (HMAS 256821); JGS: on fallen twig of angiosperm, 25 Sept. 2021, *LWZ 20210925-1b* (HMAS 256833), on fallen twig of angiosperm, 25 Sept. 2021, *LWZ 20210925-2a* (HMAS 256834), on stump of angiosperm, 25 Sept. 2021, *LWZ 20210925-2b* (HMAS 256835), on fallen twig of angiosperm, 25 Sept. 2021, *LWZ 20210925-5b* (HMAS 256840), on fallen twig of angiosperm, 25 Sept. 2021, *LWZ 20210925-6b* (HMAS 256842), on fallen branch of angiosperm, 25 Sept. 2021, *LWZ 20210925-9b* (HMAS 256844).

***Xylodon nesporii***

Specimen examined: TM: on fallen trunk of *Pinus*, 12 Oct. 2020, *LWZ 20201012-11* (HMAS 256295).

***Xylodon ovisporus***

Specimens examined: TM: on fallen trunk of *Pinus*, 11 Oct. 2020, *LWZ 20201011-35* (HMAS 256271), on fallen branch of *Pinus*, 12 Oct. 2020, *LWZ 20201012-23* (HMAS 256304); YLP: on fallen trunk of angiosperm, 14 Oct. 2020, *LWZ 20201014-11* (HMAS 256343), on fallen branch of *Pinus*, 14 Oct. 2020, *LWZ 20201014-21* (HMAS 256351); WFS: on fallen trunk of angiosperm, 16 Oct. 2020, *LWZ 20201016-5* (HMAS 256403), on dead *Pinus*, 17 Oct. 2020, *LWZ 20201017-17* (HMAS 256434); THC: on fallen twig of angiosperm, 18 Sept. 2021, *LWZ 20210918-1b* (HMAS 256477), on dead *Pinus*, 18 Sept. 2021, *LWZ 20210918-3b* (HMAS 256481), on fallen trunk of angiosperm, 18 Sept. 2021, *LWZ 20210918-13b* (HMAS 256501), on fallen twig of *Pinus*, 18 Sept. 2021, *LWZ 20210918-16b* (HMAS 256507), on stump of angiosperm, 18 Sept. 2021, *LWZ 20210918-20a* (HMAS 256513); DBS: on fallen trunk of angiosperm, 19 Sept. 2021, *LWZ 20210919-33a* (HMAS 256583), on fallen twig of angiosperm, 20 Sept. 2021, *LWZ 20210920-1b* (HMAS 256606), on fallen branch of angiosperm, 20 Sept. 2021, *LWZ 20210920-3a* (HMAS 256607), on fallen trunk of *Pinus*, 20 Sept. 2021, *LWZ 20210920-4a* (HMAS 256609), on fallen trunk of angiosperm, 20 Sept. 2021, *LWZ 20210920-12a* (HMAS 256622), on fallen trunk of angiosperm, 20 Sept. 2021, *LWZ 20210920-13b* (HMAS 256624), on fallen trunk of angiosperm, 20 Sept. 2021, *LWZ 20210920-14b* (HMAS 256626), on fallen branch of *Picea*, 20 Sept. 2021, *LWZ 20210920-24b* (HMAS 256646); WNS: on fallen trunk of angiosperm, 21 Sept. 2021, *LWZ 20210921-24a* (HMAS 256687); SZF, on fallen twig of *Pinus*, 22 Sept. 2021, *LWZ 20210922-3a* (HMAS 256711); HNDBS: on fallen twig of angiosperm, 23 Sept. 2021, *LWZ 20210923-21b* (HMAS 256767), on fallen twig of angiosperm, 23 Sept. 2021, *LWZ 20210923-24a* (HMAS 256771); JLS: on fallen twig of *Pinus*, 24 Sept. 2021, *LWZ 20210924-9b* (HMAS 256787),on stump of angiosperm, 24 Sept. 2021, *LWZ 20210924-16b* (HMAS 256800), on fallen branch of angiosperm, 24 Sept. 2021, *LWZ 20210924-36a* (HMAS 256830).

***Xylodon subflaviporus***

Specimens examined: THC: on fallen branch of angiosperm, 18 Sept. 2021, *LWZ 20210918-14b* (HMAS 256503); on fallen branch of angiosperm, 18 Sept. 2021, *LWZ 20210918-29a* (HMAS 256523); DBS: on dead *Pinus*, 19 Sept. 2021, *LWZ 20210919-22b* (HMAS 256565), on fallen trunk of angiosperm, 20 Sept. 2021, *LWZ 20210920-16a* (HMAS 256629), on fallen branch of angiosperm, 20 Sept. 2021, LWZ 20210920-19a (HMAS 256635); SZF: on dead angiosperm, 22 Sept. 2021, *LWZ 20210922-6a* (HMAS 256715), on fallen trunk of angiosperm, 22 Sept. 2021, *LWZ 20210922-10a* (HMAS 256719), on fallen branch of angiosperm, 22 Sept. 2021, *LWZ 20210922-13b* (HMAS 256722); JLS: on dead angiosperm, 24 Sept. 2021, *LWZ 20210924-2b* (HMAS 256778), on dead angiosperm, 24 Sept. 2021, *LWZ 20210924-17b* (HMAS 256802), on dead *Pinus*, 24 Sept. 2021, *LWZ 20210924-18b* (HMAS 256804), on fallen branch of angiosperm, 24 Sept. 2021, *LWZ 20210924-19b* (HMAS 256806), on fallen branch of angiosperm, 24 Sept. 2021, *LWZ 20210924-24a* (HMAS 256814), on fallen twig of angiosperm, 24 Sept. 2021, *LWZ 20210924-29b* (HMAS 256823).

***Xylodon spathulatus***

Specimens examined: DBS: on fallen trunk of angiosperm, 19 Sept. 2021, *LWZ 20210919-4b* (HMAS 256538), on base of angiosperm, 19 Sept. 2021, *LWZ 20210919-36b* (HMAS 256589); WNS: on fallen trunk of angiosperm, 21 Sept. 2021, *LWZ 20210921-6a* (HMAS 256659); SZF: on stump of angiosperm, 22 Sept. 2021, *LWZ 20210922-25a* (HMAS 256736); JLS: on fallen twig of *Picea*, 24 Sept. 2021, *LWZ 20210924-3a* (HMAS 256779).

***Xylodon serpentiformis***

Specimen examined: YLP: on fallen branch of angiosperm, 15 Oct. 2020, *LWZ 20201015-25* (HMAS 256394).

*Xenasmataceae*

*Xenasmatella*

***Xenasmatella ardosiaca***

Specimen examined: SZF: on fallen twig of angiosperm, 22 Sept. 2021, *LWZ 20210922-22b* (HMAS 256732).

Incertae sedis

*Peniophorella*

***Peniophorella pubera***

Specimen examined: TM: on fallen trunk of angiosperm, 11 Oct. 2020, *LWZ 20201011-29* (HMAS 256267).

***Peniophorella rude***

Specimens examined: THC: on dead *Pinus*, 18 Sept. 2021, *LWZ 20210918-4b* (HMAS 256483), on fallen trunk of *Pinus*, 18 Sept. 2021, *LWZ 20210918-6b* (HMAS 256487); DBS: on fallen twig of angiosperm, 19 Sept. 2021, *LWZ 20210919-5b* (HMAS 256539), on fallen branch of angiosperm, 19 Sept. 2021, *LWZ 20210919-13b* (HMAS 256552), on dead angiosperm, 19 Sept. 2021, *LWZ 20210919-31b* (HMAS 256581); WNS: on fallen trunk of angiosperm, 21 Sept. 2021, *LWZ 20210921-30b* (HMAS 256696).

***Peniophorella subpraetermissa***

Specimen examined: DBS: on fallen trunk of *Picea*, 19 Sept. 2021, *LWZ 20210919-9b* (HMAS 256545), on fallen branch of angiosperm, 19 Sept. 2021, *LWZ 20210919-11b* (HMAS 256548), on fallen twig of *Picea*, 20 Sept. 2021, *LWZ 20210920-22b* (HMAS 256642), on fallen trunk of *Pinus*, 20 Sept. 2021, *LWZ 20210920-26a* (HMAS 256648); WNS: on fallen twig of angiosperm, 21 Sept. 2021, *LWZ 20210921-5b* (HMAS 256658); JLS: on fallen branch of angiosperm, 24 Sept. 2021, *LWZ 20210924-4b* (HMAS 256781); JGS: on fallen twig of angiosperm, 25 Sept. 2021, *LWZ 20210925-14a* (HMAS 256849).

*Resinicium*

***Resinicium friabile***

Specimen examined: HNDBS: on fallen twig of angiosperm, 23 Sept. 2021, *LWZ 20210923-23a* (HMAS 256769).

*Skvortzovia*

***Skvortzovia dabieshanensis***

Specimens examined: TM: on fallen trunk of angiosperm, 11 Oct. 2020, *LWZ 20201011-22* (HMAS 256261), on fallen trunk of *Pinus*, 12 Oct. 2020, *LWZ 20201012-18* (HMAS 256300), on fallen trunk of *Pinus*, 12 Oct. 2020, *LWZ 20201012-22* (HMAS 256303); YLP: on fallen trunk of *Pinus*, 14 Oct. 2020, *LWZ 20201014-18* (HMAS 256349); WFS: on fallen trunk of angiosperm, 17 Oct. 2020, *LWZ 20201017-12* (HMAS 256429), on fallen trunk of angiosperm, 17 Oct. 2020, *LWZ 20201017-55* (HMAS 256463); THC: on stump of *Pinus*, 18 Sept. 2021, *LWZ 20210918-6a* (HMAS 256486), on fallen branch of angiosperm, 18 Sept. 2021, *LWZ 20210918-8a* (HMAS 256490), on fallen branch of *Pinus*, 18 Sept. 2021, *LWZ 20210918-15b* (HMAS 256505), on fallen trunk of *Pinus*, 18 Sept. 2021, *LWZ 20210918-25a* (HMAS 256519), on fallen branch of angiosperm, 18 Sept. 2021, *LWZ 20210918-40a* (HMAS 256531); DBS: on fallen trunk of *Pinus*, 19 Sept. 2021, *LWZ 20210919-4a* (HMAS 256537), on fallen branch of *Pinus*, 19 Sept. 2021, *LWZ 20210919-23a* (HMAS 256566), on fallen branch of angiosperm, 19 Sept. 2021, *LWZ 20210919-33b* (HMAS 256584), on fallen trunk of *Pinus*, 19 Sept. 2021, *LWZ 20210919-42a* (HMAS 256599), on fallen branch of angiosperm, 20 Sept. 2021, *LWZ 20210920-33a* (HMAS 256651); WNS: on fallen trunk of *Pinus*, 21 Sept. 2021, *LWZ 20210921-38a* (HMAS 256703), on fallen trunk of *Pinus*, 21 Sept. 2021, *LWZ 20210921-39a* (HMAS 256704), on fallen trunk of *Pinus*, 21 Sept. 2021, *LWZ 20210921-40a* (HMAS 256705); SZF: on fallen branch of *Pinus*, 22 Sept. 2021, *LWZ 20210922-1a* (HMAS 256709); HNDBS: on fallen branch of angiosperm, 23 Sept. 2021, *LWZ 20210923-13b* (HMAS 256755); JGS: on fallen twig of angiosperm, 25 Sept. 2021, *LWZ 20210925-1a* (HMAS 256832).

***Skvortzovia pinicola***

Specimens examined: TM: on fallen trunk of angiosperm, 11 Oct. 2020, *LWZ 20201011-14* (HMAS 256253), on dead angiosperm, 11 Oct. 2020, *LWZ 20201011-18* (HMAS 256257), on fallen trunk of angiosperm, 11 Oct. 2020, *LWZ 20201011-20* (HMAS 256259), on fallen trunk of angiosperm, 13 Oct. 2020, *LWZ 20201013-15* (HMAS 256330).

*Skvortzoviella*

***Skvortzoviella lenis***

Specimens examined: DBS: on fallen branch of angiosperm, 19 Sept. 2021, *LWZ 20210919-37a* (HMAS 256590); SZF: on fallen branch of angiosperm, 22 Sept. 2021, *LWZ 20210922-17b* (HMAS 256726).

*Trichaptum*

****Trichaptum abietinum***

Specimens examined: TM: on base of *Pinus*, 11 Oct. 2020, *LWZ 20201011-53* (HMAS 256289); YLP: on fallen trunk of *Pinus*, 15 Oct. 2020, *LWZ 20201015-16* (HMAS 256386); THC: on fallen branch of angiosperm, 18 Sept. 2021, *LWZ 20210918-9a* (HMAS 256492); DBS: on fallen branch of *Pinus*, 19 Sept. 2021, *LWZ 20210919-3a* (HMAS 256535), on fallen branch of *Pinus*, 19 Sept. 2021, *LWZ 20210919-15a* (HMAS 256554), on fallen branch of angiosperm, 19 Sept. 2021, *LWZ 20210919-37b* (HMAS 256591), on fallen branch of *Pinus*, 20 Sept. 2021, *LWZ 20210920-15a* (HMAS 256627), on fallen trunk of angiosperm, 20 Sept. 2021, *LWZ 20210920-17a* (HMAS 256631); SZF: on fallen branch of angiosperm, 22 Sept. 2021, *LWZ 20210922-4b* (HMAS 256714); JLS: on fallen branch of *Pinus*, 24 Sept. 2021, *LWZ 20210924-7a* (HMAS 256784).

****Trichaptum fuscoviolaceum***

Specimens examined: THC: on fallen trunk of *Pinus*, 18 Sept. 2021, *LWZ 20210918-5b* (HMAS 256485), on fallen branch of angiosperm, 18 Sept. 2021, *LWZ 20210918-30a* (HMAS 256524); DBS: on fallen trunk of angiosperm, 20 Sept. 2021, *LWZ 20210920-20a* (HMAS 256637), on fallen branch of *Picea*, 20 Sept. 2021, *LWZ 20210920-21b* (HMAS 256640).

*Polyporales*

*Cerrenaceae*

*Cerrena*

***Cerrena albocinnamomea***

Specimens examined: TM: on fallen trunk of angiosperm, 11 Oct. 2020, *LWZ 20201011-17* (HMAS 256256), on dead *Pinus*, 11 Oct. 2020, *LWZ 20201011-41* (HMAS 256277); THC: on fallen trunk of *Pinus*, 18 Sept. 2021, *LWZ 20210918-8b* (HMAS 256491), on fallen branch of angiosperm, 18 Sept. 2021, *LWZ 20210918-11b* (HMAS 256497), on fallen trunk of angiosperm, 18 Sept. 2021, *LWZ 20210918-12a* (HMAS 256498).

****Cerrena zonata***

Specimens examined: TM: on fallen trunk of angiosperm, 12 Oct. 2020, *LWZ 20201012-43* (HMAS 256317), on stump of angiosperm, 12 Oct. 2020, *LWZ 20201012-40* (HMAS 256315), on stump of angiosperm, 13 Oct. 2020, *LWZ 20201013-1*(HMAS 256321), on fallen trunk of angiosperm, 13 Oct. 2020, *LWZ 20201013-10* (HMAS 256326); WFS: on fallen trunk of angiosperm, 16 Oct. 2020, *LWZ 20201016-6* (HMAS 256404); DBS: on stump of angiosperm, 19 Sept. 2021, *LWZ 20210919-39a* (HMAS 256594), on fallen twig of angiosperm, 20 Sept. 2021, *LWZ 20210920-5b* (HMAS 256612), on fallen branch of angiosperm, 20 Sept. 2021, *LWZ 20210920-6a* (HMAS 256613), on fallen trunk of angiosperm, 20 Sept. 2021, *LWZ 20210920-6b* (HMAS 256614); SZF: on fallen branch of angiosperm, 22 Sept. 2021, *LWZ 20210922-18a* (HMAS 256727); HNDBS: on stump of *Pinus*, 23 Sept. 2021, *LWZ 20210923-11a* (HMAS 256752); JLS: on fallen trunk of angiosperm, 24 Sept. 2021, *LWZ 20210924-19a* (HMAS 256805).

*Dacryobolaceae*

*Dacryobolus*

***Dacryobolus karstenii***

Specimen examined: THC: on fallen branch of *Pinus*, 18 Sept. 2021, *LWZ 20210918-17b* (HMAS 256509).

*Postia*

****Postia hirsuta***

Specimens examined: TM: on fallen trunk of angiosperm, 12 Oct. 2020, *LWZ 20201012-29* (HMAS 256309); THC: on stump of *Pinus*, 18 Sept. 2021, *LWZ 20210918-19a* (HMAS 256511); JGS: on fallen branch of angiosperm, 25 Sept. 2021, *LWZ 20210925-11b* (HMAS 256846).

***Postia*** **sp.**

Specimen examined: WFS: on fallen trunk of angiosperm, 16 Oct. 2020, *LWZ 20201016-7* (HMAS 256405).

*Fibroporiaceae*

*Fibroporia*

***Fibroporia albicans***

Specimen examined: DBS: on fallen trunk of angiosperm, 19 Sept. 2021, *LWZ 20210919-3b* (HMAS 256536).

*Fomitopsidaceae*

*Antrodia*

****Antrodia tanakae***

Specimen examined: DBS: on fallen trunk of angiosperm, 20 Sept. 2021, *LWZ 20210920-31a* (HMAS 256649).

***Antrodia* sp. 1**

Specimen examined: TM: on fallen branch of *Quercus*, 12 Oct. 2020, *LWZ 20201012-9* (HMAS 256294).

***Antrodia* sp. 2**

Specimen examined: HNDBS: on fallen twig of angiosperm, 23 Sept. 2021, *LWZ 20210923-17b* (HMAS 256759).

*Daedalea*

****Daedalea dickinsii***

Specimens examined: TM: on fallen trunk of angiosperm, 11 Oct. 2020, *LWZ 20201011-45* (HMAS 256281); WFS: on fallen trunk of angiosperm, 17 Oct. 2020, *LWZ 20201017-21* (HMAS 256438), on fallen trunk of angiosperm, 17 Oct. 2020, *LWZ 20201017-27* (HMAS 256443).

*Fomitopsis*

***Fomitopsis palustris***

Specimen examined: JLS: on fallen trunk of angiosperm, 24 Sept. 2021, *LWZ 20210924-5a* (HMAS 256782).

***⬧*Fomitopsis pinicola***

Specimens examined: DBS: on fallen trunk of angiosperm, 20 Sept. 2021, *LWZ 20210920-11b* (HMAS 256621), on fallen trunk of *Pinus*, 20 Sept. 2021, *LWZ 20210920-13a* (HMAS 256623).

*Rhodofomes*

***Rhodofomes incarnatus***

Specimen examined: TM: on fallen trunk of angiosperm, 12 Oct. 2020, *LWZ 20201012-32* (HMAS 256311).

*Gelatoporiaceae*

*Cinereomyces*

***Cinereomyces lindbladii***

Specimens examined: YLP: on fallen trunk of angiosperm, 15 Oct. 2020, *LWZ 20201015-22* (HMAS 256391); THC: on fallen trunk of *Pinus*, 18 Sept. 2021, *LWZ 20210918-7b* (HMAS 256489), on fallen branch of *Pinus*, 18 Sept. 2021, *LWZ 20210918-11a* (HMAS 256496); SZF: on fallen branch of angiosperm, 22 Sept. 2021, *LWZ 20210922-3b* (HMAS 256712).

*Grifolaceae*

*Grifola*

**⦁**Grifola frondosa***

Specimen examined: TM: on stump of angiosperm, 11 Oct. 2020, *LWZ 20201011-10* (HMAS 256250).

*Hyphodermataceae*

*Hyphoderma*

***Hyphoderma singularibasidium***

Specimens examined: THC: on fallen trunk of *Pinus*, 18 Sept. 2021, *LWZ 20210918-15a* (HMAS 256504); DBS: on fallen branch of angiosperm, 19 Sept. 2021, *LWZ 20210919-17b* (HMAS 256558).

***Hyphoderma nudicephalum***

Specimen examined: HNDBS: on falllen branch of angiosperm, 23 Sept. 2021, *LWZ 20210923-2a* (HMAS 256741).

***Hyphoderma pinicola***

Specimen examined: SZF: on fallen twig of angiosperm, 22 Sept. 2021, *LWZ 20210922-6b* (HMAS 256716).

***Hyphoderma subsetigerum***

Specimens examined: DBS: on fallen branch of angiosperm, 19 Sept. 2021, *LWZ 20210919-34b* (HMAS 256586), on fallen branch of angiosperm, 20 Sept. 2021, *LWZ 20210920-3b* (HMAS 256608), on fallen branch of angiosperm, 20 Sept. 2021, *LWZ 20210920-18a* (HMAS 256633); WNS: on fallen twig of angiosperm, 21 Sept. 2021, *LWZ 20210921-19a* (HMAS 256680), on fallen twig of angiosperm, 21 Sept. 2021, *LWZ 20210921-32b* (HMAS 256699); SZF: on fallen twig of angiosperm, 22 Sept. 2021, *LWZ 20210922-20a* (HMAS 256729), on fallen twig of angiosperm, 22 Sept. 2021, *LWZ 20210922-23b* (HMAS 256734); JLS: on fallen branch of *Pinus*, 24 Sept. 2021, *LWZ 20210924-29a* (HMAS 256822).

***Hyphoderma transiens***

Specimen examined: DBS: on fallen twig of angiosperm, 19 Sept. 2021, *LWZ 20210919-25b* (HMAS 256571).

***Hyphoderma* sp.**

Specimen examined: JGS: on fallen twig of angiosperm, 25 Sept. 2021, *LWZ 20210925-6a* (HMAS 256841).

*Incrustoporiaceae*

*Skeletocutis*

***Skeletocutis friata***

Specimens examined: TM: on fallen trunk of angiosperm, 11 Oct. 2020, *LWZ 20201011-33* (HMAS 256270); YLP: on fallen trunk of angiosperm, 14 Oct. 2020, *LWZ 20201014-6* (HMAS 256338), on fallen trunk of *Quercus*, 14 Oct. 2020, *LWZ 20201014-35* (HMAS 256364), on fallen trunk of angiosperm, 15 Oct. 2020, *LWZ 20201015-15* (HMAS 256385); WFS: on fallen branch of angiosperm, 16 Oct. 2020, *LWZ 20201016-15* (HMAS 256411), on dead angiosperm, 17 Oct. 2020, *LWZ 20201017-14* (HMAS 256431).

***Skeletocutis lepida***

Specimen examined: YLP: on dead *Quercus*, 14 Oct. 2020, *LWZ 20201014-26* (HMAS 256356); DBS: on fallen branch of angiosperm, 19 Sept. 2021, *LWZ 20210919-14a* (HMAS 256553), on fallen branch of angiosperm, 19 Sept. 2021, *LWZ 20210919-31a* (HMAS 256580).

***Skeletocutis mopanshanensis***

Specimen examined: TM: on fallen trunk of angiosperm, 11 Oct. 2020, *LWZ 20201011-23* (HMAS 256262).

*Irpicaceae*

*Irpex*

****Irpex lacteus***

Specimens examined: THC: on fallen branch of angiosperm, 18 Sept. 2021, *LWZ 20210918-26a* (HMAS 256520); DBS: on fallen trunk of angiosperm, 20 Sept. 2021, *LWZ 20210920-11a* (HMAS 256620), on fallen trunk of *Pinus*, 20 Sept. 2021, *LWZ 20210920-36a* (HMAS 256652); WNS: on fallen twig of angiosperm, 21 Sept. 2021, *LWZ 20210921-1a* (HMAS 256653), on fallen twig of angiosperm, 21 Sept. 2021, *LWZ 20210921-6b* (HMAS 256660), on fallen branch of angiosperm, 21 Sept. 2021, *LWZ 20210921-7b* (HMAS 256662), on fallen twig of angiosperm, 21 Sept. 2021, *LWZ 20210921-10a* (HMAS 256667), on fallen twig of angiosperm, 21 Sept. 2021, *LWZ 20210921-11b* (HMAS 256669), on fallen branch of angiosperm, 21 Sept. 2021, *LWZ 20210921-14a* (HMAS 256673), on fallen branch of angiosperm, 21 Sept. 2021, *LWZ 20210921-14b* (HMAS 256674), on fallen twig of angiosperm, 21 Sept. 2021, *LWZ 20210921-15b* (HMAS 256675), on fallen trunk of angiosperm, 21 Sept. 2021, *LWZ 20210921-31a* (HMAS 256697); SZF: on fallen branch of *Pinus*, 22 Sept. 2021, *LWZ 20210922-14a* (HMAS 256723); HNDBS: on fallen branch of angiosperm, 23 Sept. 2021, *LWZ 20210923-24b* (HMAS 256772), on fallen trunk of angiosperm, 23 Sept. 2021, *LWZ 20210923-25b* (HMAS 256773); JLS: on fallen trunk of angiosperm, 24 Sept. 2021, *LWZ 20210924-23b* (HMAS 256813), on fallen twig of angiosperm, 24 Sept. 2021, *LWZ 20210924-37a* (HMAS 256831); JGS: on fallen twig of angiosperm, 25 Sept. 2021, *LWZ 20210925-15a* (HMAS 256851), on fallen twig of angiosperm, 25 Sept. 2021, *LWZ 20210925-22a* (HMAS 256858).

*Leptoporus*

***Leptoporus mollis***

Specimens examined: TM: on fallen trunk of *Pinus*, 12 Oct. 2020, *LWZ 20201012-27* (HMAS 256308); YLP: on dead *Pinus*, 14 Oct. 2020, *LWZ 20201014-19* (HMAS 256350).

*Meruliopsis*

***Meruliopsis leptocystidiata***

Specimen examined: THC: on fallen branch of *Picea*, 18 Sept. 2021, *LWZ 20210918-36a* (HMAS 256527).

***Meruliopsis nanlingensis***

Specimen examined: THC: on fallen branch of angiosperm, 18 Sept. 2021, *LWZ 20210918-37a* (HMAS 256528).

***Meruliopsis taxicola***

Specimens examined: DBS: on fallen branch of angiosperm, 19 Sept. 2021, *LWZ 20210919-18b* (HMAS 256560), on fallen trunk of angiosperm, 19 Sept. 2021, *LWZ 20210919-20b* (HMAS 256562).

*Vitreoporus*

***Vitreoporus dichrous***

Specimen examined: DBS: on fallen trunk of angiosperm, 19 Sept. 2021, *LWZ 20210919-17a* (HMAS 256557).

*Meruliaceae*

*Hydnophlebia*

***Hydnophlebia subchrysorhiza***

Specimen examined: TM: on fallen trunk of angiosperm, 11 Oct. 2020, *LWZ 20201011-28* (HMAS 256266).

*Mycoacia*

***Mycoacia fuscoatra***

Specimen examined: TM: on fallen trunk of angiosperm, 11 Oct. 2020, *LWZ 20201011-24* (HMAS 256263).

***Mycoacia livida***

Specimen examined: YLP: on fallen trunk of angiosperm, 15 Oct. 2020, *LWZ 20201015-13* (HMAS 256383).

***Mycoacia lutea***

Specimens examined: DBS: on fallen branch of angiosperm, 19 Sept. 2021, *LWZ 20210919-30a* (HMAS 256578); JLS: on dead angiosperm, 24 Sept. 2021, *LWZ 20210924-20b* (HMAS 256808), on fallen trunk of *Pinus*, 24 Sept. 2021, *LWZ 20210924-32a* (HMAS 256827).

*Phlebia*

***Phlebia acerina***

Specimens examined: THC: on stump of angiosperm, 18 Sept. 2021, *LWZ 20210918-19b* (HMAS 256512); DBS: on fallen branch of angiosperm, 19 Sept. 2021, *LWZ 20210919-18a* (HMAS 256559); WNS: on fallen branch of angiosperm, 21 Sept. 2021, *LWZ 20210921-20a* (HMAS 256681).

****Phlebia tremellosa***

Specimens examined: TM: on fallen trunk of angiosperm, 11 Oct. 2020, *LWZ 20201011-50* (HMAS 256286), on fallen trunk of angiosperm, 13 Oct. 2020, *LWZ 20201013-7* (HMAS 256323), on fallen trunk of angiosperm, 13 Oct. 2020, *LWZ 20201013-23* (HMAS 256335); YLP: on fallen trunk of angiosperm, 15 Oct. 2020, *LWZ 20201015-5* (HMAS 256375); WFS: on fallen branch of *Pinus*, 17 Oct. 2020, *LWZ 20201017-16* (HMAS 256433), on dead angiosperm, 17 Oct. 2020, *LWZ 20201017-24* (HMAS 256440); DBS: on fallen trunk of angiosperm, 19 Sept. 2021, *LWZ 20210919-27a* (HMAS 256574).

*Phanerochaetaceae*

*Bjerkandera*

***⬧*Bjerkandera adusta***

Specimens examined: TM: on fallen trunk of angiosperm, 13 Oct. 2020, *LWZ 20201013-9* (HMAS 256325); WFS: on fallen trunk of angiosperm, 17 Oct. 2020, *LWZ 20201017-33* (HMAS 256449), on fallen trunk of angiosperm, 17 Oct. 2020, *LWZ 20201017-38* (HMAS 256453).

**⦁**Bjerkandera fumosa***

Specimen examined: WFS: on fallen trunk of angiosperm, 16 Oct. 2020, *LWZ 20201016-9* (HMAS 256407).

*Phaeophlebiopsis*

***Phaeophlebiopsis himalayensis***

Specimen examined: WNS: on fallen branch of angiosperm, 21 Sept. 2021, *LWZ 20210921-8a* (HMAS 256663).

*Phanerochaete*

***Phanerochaete canolutea***

Specimen examined: WNS: on fallen branch of angiosperm, 21 Sept. 2021, *LWZ 20210921-4a* (HMAS 256656).

***Phanerochaete* *concrescens***

Specimens examined: WNS: on fallen twig of angiosperm, 21 Sept. 2021, *LWZ 20210921-18a* (HMAS 256679), on fallen twig of angiosperm, 21 Sept. 2021, *LWZ 20210921-20b* (HMAS 256682), on fallen twig of angiosperm, 21 Sept. 2021, *LWZ 20210921-26b* (HMAS 256689); HNDBS: on fallen branch of angiosperm, 23 Sept. 2021, *LWZ 20210923-20a* (HMAS 256764); JLS: on fallen twig of angiosperm, 24 Sept. 2021, *LWZ 20210924-13b* (HMAS 256794), on fallen branch of angiosperm, 24 Sept. 2021, *LWZ 20210924-15b* (HMAS 256798); JGS: on fallen twig of angiosperm, 25 Sept. 2021, *LWZ 20210925-4b* (HMAS 256838), on fallen branch of angiosperm, 25 Sept. 2021, *LWZ 20210925-14b* (HMAS 256850).

***Phanerochaete* *laevis***

Specimen examined: DBS: on base of *Pinus*, 19 Sept. 2021, *LWZ 20210919-16b* (HMAS 256556).

***Phanerochaete* *sordida***

Specimen examined: DBS: on fallen trunk of angiosperm, 19 Sept. 2021, *LWZ 20210919-38b* (HMAS 256593).

*Phlebiopsis*

***Phlebiopsis* *castanea***

Specimens examined: YLP: on fallen trunk of *Pinus*, 15 Oct. 2020, *LWZ 20201015-3* (HMAS 256373), on fallen trunk of angiosperm, 15 Oct. 2020, *LWZ 20201015-4* (HMAS 256374).

***Phlebiopsis* *crassa***

Specimens examined: TM: on fallen trunk of angiosperm, 11 Oct. 2020, *LWZ 20201011-2* (HMAS 256242); WFS: on fallen trunk of *Quercus*, 17 Oct. 2020, *LWZ 20201017-9* (HMAS 256426).

***Phlebiopsis* *gigantea***

Specimens examined: DBS: on dead *Pinus*, 19 Sept. 2021, *LWZ 20210919-13a* (HMAS 256551); JLS: on dead *Pinus*, 24 Sept. 2021, *LWZ 20210924-33a* (HMAS 256828).

*Porostereum*

***Porostereum* *spadiceum***

Specimen examined: TM: on fallen trunk of angiosperm, 11 Oct. 2020, *LWZ 20201011-8* (HMAS 256248).

*Terana*

***Terana* *coerulea***

Specimens examined: TM: on fallen branch of angiosperm, 12 Oct. 2020, *LWZ 20201012-46* (HMAS 256320); YLP: on fallen branch of angiosperm, 15 Oct. 2020, *LWZ 20201015-33* (HMAS 256400).

*Polyporaceae*

*Abundisporus*

***Abundisporus* *pubertatis***

Specimen examined: YLP: on fallen trunk of angiosperm, 15 Oct. 2020, *LWZ 20201015-2* (HMAS 256372).

*Cyanosporus*

***Cyanosporus* *coeruleivirens***

Specimen examined: TM: on fallen trunk of angiosperm, 11 Oct. 2020, *LWZ 20201011-15* (HMAS 256254).

***Cyanosporus* *ungulatus***

Specimen examined: WFS: on fallen trunk of angiosperm, 16 Oct. 2020, *LWZ 20201016-14* (HMAS 256410).

*Daedaleopsis*

****Daedaleopsis* *tricolor***

Specimens examined: WFS: on dead angiosperm, 17 Oct. 2020, *LWZ 20201017-5* (HMAS 256423); DBS: on fallen trunk of angiosperm, 19 Sept. 2021, *LWZ 20210919-29a* (HMAS 256576), on fallen branch of angiosperm, 20 Sept. 2021, *LWZ 20210920-21a* (HMAS 256639), on fallen trunk of angiosperm, 20 Sept. 2021, *LWZ 20210920-32a* (HMAS 256650); WNS: on fallen trunk of angiosperm, 21 Sept. 2021, *LWZ 20210921-12b* (HMAS 256671), on fallen trunk of angiosperm, 21 Sept. 2021, *LWZ 20210921-23b* (HMAS 256686), on fallen branch of angiosperm, 21 Sept. 2021, *LWZ 20210921-29a* (HMAS 256693); HNDBS: on fallen branch of angiosperm, 23 Sept. 2021, *LWZ 20210923-26a* (HMAS 256774); JLS: on dead angiosperm, 24 Sept. 2021, *LWZ 20210924-10b* (HMAS 256789), on dead angiosperm, 24 Sept. 2021, *LWZ 20210924-12a* (HMAS 256792), on fallen trunk of angiosperm, 24 Sept. 2021, *LWZ 20210924-22b* (HMAS 256811); JGS: on fallen trunk of angiosperm, 25 Sept. 2021, *LWZ 20210925-5a* (HMAS 256839), on fallen trunk of angiosperm, 25 Sept. 2021, *LWZ 20210925-18a* (HMAS 256854), on fallen trunk of *Picea*, 25 Sept. 2021, *LWZ 20210925-18b* (HMAS 256855).

*Datronia*

***Datronia* *mollis***

Specimens examined: YLP: on fallen trunk of angiosperm, 14 Oct. 2020, *LWZ 20201014-29* (HMAS 256359); WFS: on fallen trunk of angiosperm, 16 Oct. 2020, *LWZ 20201016-13* (HMAS 256409), on fallen trunk of angiosperm, 17 Oct. 2020, *LWZ 20201017-31* (HMAS 256447).

*Ganoderma*

***Ganoderma* *gibbosum***

Specimens examined: TM: on stump of angiosperm, 13 Oct. 2020, *LWZ 20201013-8* (HMAS 256324), on dead angiosperm, 13 Oct. 2020, *LWZ 20201013-11* (HMAS 256327); HNDBS: on fallen twig of angiosperm, 23 Sept. 2021, *LWZ 20210923-6b* (HMAS 256747), on stump of angiosperm, 23 Sept. 2021, *LWZ 20210923-10b* (HMAS 256751).

****Ganoderma* *lingzhi***

Specimen examined: HNDBS: on base of angiosperm, 23 Sept. 2021, *LWZ 20210923-22b* (HMAS 256768).

*Lentinus*

***Lentinus* *brumalis***

Specimen examined: YLP: on fallen trunk of angiosperm, 14 Oct. 2020, *LWZ 20201014-17* (HMAS 256348).

*Lenzites*

****Lenzites* *betulinus***

Specimen examined: THC: on fallen branch of angiosperm, 18 Sept. 2021, *LWZ 20210918-10b* (HMAS 256495), on fallen trunk of angiosperm, 19 Sept. 2021, *LWZ 20210919-50a* (HMAS 256605).

***Lenzites* *styracinus***

Specimen examined: DBS: on dead angiosperm, 19 Sept 2021, *LWZ 20210919-30b* (HMAS 256579), on fallen branch of angiosperm, 19 Sept. 2021, *LWZ 20210919-41b* (HMAS 256598).

*Lopharia*

***Lopharia* *mirabilis***

Specimens examined: YLP: on fallen trunk of angiosperm, 14 Oct. 2020, *LWZ 20201014-37* (HMAS 256366); WNS: on fallen twig of angiosperm, 21 Sept. 2021, *LWZ 20210921-10b* (HMAS 256668); HNDBS, on fallen twig of angiosperm, 23 Sept. 2021, *LWZ 20210923-16b* (HMAS 256757); JLS: on fallen trunk of angiosperm, 24 Sept. 2021, *LWZ 20210924-27a* (HMAS 256820); JGS: on fallen twig of angiosperm, 25 Sept. 2021, *LWZ 20210925-4a* (HMAS 256837).

***Lopharia* sp.**

Specimen examined: HNDBS: on fallen twig of angiosperm, 23 Sept. 2021, *LWZ 20210923-4a* (HMAS 256744).

*Mariorajchenbergia*

***Mariorajchenbergia* *subcavernulosa***

Specimens examined: TM: on fallen trunk of *Quercus*, 12 Oct. 2020, *LWZ 20201012-6* (HMAS 256292); DBS: on fallen branch of angiosperm, 20 Sept. 2021, *LWZ 20210920-18b* (HMAS 256634).

*Megasporoporiella*

***Megasporoporiella* *subcavernulosa***

Specimen examined: DBS: on fallen branch of angiosperm, 20 Sept. 2021, *LWZ 20210920-16b* (HMAS 256630).

*Perenniporia*

**⬧*Perenniporia* *truncatospora***

Specimen examined: SZF: on stump of angiosperm, 22 Sept. 2021, *LWZ 20210922-15a* (HMAS 256724).

***Perenniporia*** **sp**.

Specimen examined: THC: on fallen branch of angiosperm, 18 Sept. 2021, *LWZ 20210918-7a* (HMAS 256488).

*Trametes*

****Trametes* *hirsuta***

Specimens examined: TM: on fallen trunk of angiosperm, 11 Oct. 2020, *LWZ 20201011-4* (HMAS 256244), on fallen trunk of angiosperm, 11 Oct. 2020, *LWZ 20201011-11* (HMAS 256251); JGS: on fallen twig of angiosperm, 25 Sept. 2021, *LWZ 20210925-13a* (HMAS 256847).

***Trametes* *strumosa***

Specimen examined: JLS: on fallen branch of angiosperm, 24 Sept. 2021, *LWZ 20210924-16a* (HMAS 256799).

****Trametes* *versicolor***

Specimens examined: TM: on fallen branch of angiosperm, 11 Oct. 2020, *LWZ 20201011-6* (HMAS 256246); YLP: on branch of living angiosperm, 15 Oct. 2020, *LWZ 20201015-27* (HMAS 256396); WFS: on fallen trunk of angiosperm, 16 Oct. 2020, *LWZ 20201016-22* (HMAS 256416), on fallen branch of angiosperm, 17 Oct. 2020, *LWZ 20201017-6* (HMAS 256424), on dead angiosperm, 17 Oct. 2020, *LWZ 20201017-30* (HMAS 256446), on fallen trunk of angiosperm, 17 Oct. 2020, *LWZ 20201017-54* (HMAS 256462); THC: on fallen branch of angiosperm, 18 Sept. 2021, *LWZ 20210918-2a* (HMAS 256478); DBS: on base of *Pinus*, 19 Sept. 2021, *LWZ 20210919-43a* (HMAS 256600), on fallen branch of angiosperm, 20 Sept. 2021, *LWZ 20210920-19b* (HMAS 256636); JLS: on fallen twig of *Pinus*, 24 Sept. 2021, *LWZ 20210924-8b* (HMAS 256786).

*Truncospora*

***Truncospora* *ochroleuca***

Specimens examined: DBS: on dead angiosperm, 20 Sept. 2021, *LWZ 20210920-23a* (HMAS 256643); WNS: on fallen branch of angiosperm, 21 Sept. 2021, *LWZ 20210921-21b* (HMAS 256684), on fallen trunk of angiosperm, 21 Sept. 2021, *LWZ 20210921-30a* (HMAS 256695).

*Steccherinaceae*

*Antrodiella*

***Antrodiella* *onychoides***

Specimens examined: YLP: on fallen trunk of angiosperm, 14 Oct. 2020, *LWZ 20201014-14* (HMAS 256345); JLS: on fallen branch of angiosperm, 24 Sept. 2021, *LWZ 20210924-18a* (HMAS 256803).

*Junghuhnia*

***Junghuhnia nitida***

Specimens examined: YLP: on fallen branch of angiosperm, 14 Oct. 2020, *LWZ 20201014-31* (HMAS 256360), on fallen trunk of angiosperm, 15 Oct. 2020, *LWZ 20201015-26* (HMAS 256395); THC: on fallen trunk of *Picea*, 18 Sept. 2021, *LWZ 20210918-17a* (HMAS 256508).

*Steccherinum*

***Steccherinum* *bourdotii***

Specimen examined: DBS: on fallen branch of angiosperm, 20 Sept. 2021, *LWZ 20210920-4b* (HMAS 256610).

***Steccherinum* *ciliolatum***

Specimen examined: WFS: on fallen trunk of angiosperm, 16 Oct. 2020, *LWZ 20201016-10* (HMAS 256408); DBS: on stump of *Pinus*, 19 Sept. 2021, *LWZ 20210919-45a* (HMAS 256602); HNDBS: on fallen trunk of angiosperm, 23 Sept. 2021, *LWZ 20210923-27a* (HMAS 256775).

***Steccherinum* *ochraceum***

Specimen examined: HNDBS: on fallen branch of angiosperm, 23 Sept. 2021, *LWZ 20210923-19a* (HMAS 256762).

***Steccherinum* *tenuissimum***

Specimens examined: WNS: on fallen twig of *Pinus*, 21 Sept. 2021, *LWZ 20210921-33a* (HMAS 256700); HNDBS: on fallen twig of angiosperm, 23 Sept. 2021, *LWZ 20210923-3a* (HMAS 256742); SZF: on fallen twig of angiosperm, 22 Sept. 2021, *LWZ 20210922-26a* (HMAS 256737).

***Steccherinum* sp.**

Specimen examined: HNDBS: on fallen twig of angiosperm, 23 Sept. 2021, *LWZ 20210923-18b* (HMAS 256761).

Incertae sedis

*Amaropostia*

***Amaropostia stiptica***

Specimen examined: SZF: on stump of angiosperm, 22 Sept. 2021, *LWZ 20210922-19a* (HMAS 256728).

*Cystidiopostia*

***Cystidiopostia hibernica***

Specimen examined: DBS: on fallen branch of angiosperm, 20 Sept. 2021, *LWZ 20210920-7b* (HMAS 256616).

*Fuscopostia*

***Fuscopostia fragilis***

Specimens examined: TM: on fallen trunk of *Pinus*, 11 Oct. 2020, *LWZ 20201011-42* (HMAS 256278), on fallen trunk of *Pinus*, 12 Oct. 2020, *LWZ 20201012-12* (HMAS 256296).

*Hypochnicium*

***Hypochnicium karstenii***

Specimens examined: WFS: on fallen branch of angiosperm, 17 Oct. 2020, *LWZ 20201017-18* (HMAS 256435); DBS: on stump of *Pinus*, 19 Sept. 2021, *LWZ 20210919-41a* (HMAS 256597); SZF: on dead *Pinus*, 22 Sept. 2021, *LWZ 20210922-16a* (HMAS 256725); JLS: on fallen twig of *Pinus*, 24 Sept. 2021, *LWZ 20210924-11b* (HMAS 256791), on fallen twig of angiosperm, 24 Sept. 2021, *LWZ 20210924-17a* (HMAS 256801), on fallen twig of angiosperm, 24 Sept. 2021, *LWZ 20210924-34a* (HMAS 256829).

***Hypochnicium pini***

Specimen examined: WNS: on fallen trunk of angiosperm, 21 Sept. 2021, *LWZ 20210921-21a* (HMAS 256683).

*Piptoporellus*

***Piptoporellus soloniensis***

Specimen examined: YLP: on fallen trunk of angiosperm, 15 Oct. 2020, *LWZ 20201015-23* (HMAS 256392); WNS: on stump of *Pinus*, 21 Sept. 2021, *LWZ 20210921-36a* (HMAS 256701).

*Russulales*

*Auriscalpiaceae*

*Artomyces*

***Artomyces* *microsporus***

Specimen examined: WNS: on dead angiosperm, 21 Sept. 2021, *LWZ 20210921-27b* (HMAS 256690).

*Auriscalpium*

***Auriscalpium* *orientale***

Specimen examined: TM: on a cone of *Pinus*, 11 Oct. 2020, *LWZ 20201011-44* (HMAS 256280).

*Bondarzewiaceae*

*Heterobasidion*

***Heterobasidion* *araucariae***

Specimens examined: DBS: on stump of *Pinus*, 19 Sept. 2021, *LWZ 20210919-26a* (HMAS 256572); SZF: on stump of *Pinus*, 22 Sept. 2021, *LWZ 20210922-2a* (HMAS 256710), on fallen twig of angiosperm, 22 Sept. 2021, *LWZ 20210922-23a* (HMAS 256733).

*Hericiaceae*

*Pseudowrightoporia*

***Pseudowrightoporia* *japonica***

Specimen examined: HNDBS: on fallen twig of angiosperm, 23 Sept. 2021, *LWZ 20210923-17a* (HMAS 256758).

*Peniophoraceae*

*Asterostroma*

***Asterostroma* *andinum***

Specimen examined: TM: on fallen trunk of angiosperm, 12 Oct. 2020, *LWZ 20201012-38* (HMAS 256314).

*Peniophora*

***Peniophora* *cinerea***

Specimens examined: YLP: on fallen twig of bamboo, 15 Oct. 2020, *LWZ 20201015-29* (HMAS 256397); DBS: on fallen branch of angiosperm, 20 Sept. 2021, *LWZ 20210920-22a* (HMAS 256641); WNS: on fallen twig of angiosperm, 21 Sept. 2021, *LWZ 20210921-8b* (HMAS 256664), on fallen twig of angiosperm, 21 Sept. 2021, *LWZ 20210921-16b* (HMAS 256677); SZF: on fallen twig of angiosperm, 22 Sept. 2021, *LWZ 20210922-28a* (HMAS 256738); HNDBS: on fallen twig of angiosperm, 23 Sept. 2021, *LWZ 20210923-20b* (HMAS 256765); JGS: on fallen twig of angiosperm, 25 Sept. 2021, *LWZ 20210925-17a* (HMAS 256853).

***Peniophora* sp.**

Specimen examined: YLP: on fallen branch of angiosperm, 14 Oct. 2020, *LWZ 20201014-32* (HMAS 256361).

*Scytinostroma*

***Scytinostroma* *renisporum***

Specimens examined: YLP: on fallen trunk of angiosperm, 14 Oct. 2020, *LWZ 20201014-22* (HMAS 256352), on fallen trunk of angiosperm, 15 Oct. 2020, *LWZ 20201015-18* (HMAS 256387), on fallen branch of angiosperm, 15 Oct. 2020, *LWZ 20201015-20* (HMAS 256389); WFS: on fallen branch of angiosperm, 17 Oct. 2020, *LWZ 20201017-63* (HMAS 256471); DBS: on fallen branch of angiosperm, 19 Sept. 2021, *LWZ 20210919-49a* (HMAS 256604); JLS: on fallen branch of angiosperm, 24 Sept. 2021, *LWZ 20210924-6a* (HMAS 256783), on fallen trunk of angiosperm, 24 Sept. 2021, *LWZ 20210924-14a* (HMAS 256795), on fallen twig of angiosperm, 24 Sept. 2021, *LWZ 20210924-15a* (HMAS 256797).

*Stereaceae*

*Aleurodiscus*

***Aleurodiscus formosanus***

Specimen examined: WFS: on fallen branch of angiosperm, 17 Oct. 2020, *LWZ 20201017-58* (HMAS 256466).

*Gloeocystidiellum*

***Gloeocystidiellum* *kenyense***

Specimen examined: WFS: on fallen trunk of angiosperm, 17 Oct. 2020, *LWZ 20201017-61* (HMAS 256469).

*Stereum*

****Stereum* *hirsutum***

Specimens examined: TM: on fallen trunk of angiosperm, 11 Oct. 2020, *LWZ 20201011-5* (HMAS 256245), on fallen branch of angiosperm, 12 Oct. 2020, *LWZ 20201012-1* (HMAS 256290); WFS: on stump of angiosperm, 17 Oct. 2020, *LWZ 20201017-1* (HMAS 256421), on base of angiosperm, 17 Oct. 2020, *LWZ 20201017-23* (HMAS 256439), on fallen branch of *Pinus*, 17 Oct. 2020, *LWZ 20201017-45* (HMAS 256456); DBS: on fallen branch of angiosperm, 19 Sept. 2021, *LWZ 20210919-11a* (HMAS 256547); WNS: on fallen branch of angiosperm, 21 Sept. 2021, *LWZ 20210921-2b* (HMAS 256655), on fallen twig of angiosperm, 21 Sept. 2021, *LWZ 20210921-9b* (HMAS 256666), on fallen branch of angiosperm, 21 Sept. 2021, *LWZ 20210921-13a* (HMAS 256672).

**⬧*Stereum* *sanguinolentum***

Specimens examined: TM: on fallen branch of *Quercus*, 12 Oct. 2020, *LWZ 20201012-8* (HMAS 256293); YLP: on fallen branch of angiosperm, 14 Oct. 2020, *LWZ 20201014-3* (HMAS 256336), on fallen branch of angiosperm, 14 Oct. 2020, *LWZ 20201014-39* (HMAS 256368); WFS: on stump of *Pinus*, 16 Oct. 2020, *LWZ 20201016-27* (HMAS 256420), on dead angiosperm, 17 Oct. 2020, *LWZ 20201017-10* (HMAS 256427), on fallen branch of *Pinus*, 17 Oct. 2020, *LWZ 20201017-15* (HMAS 256432), on dead angiosperm, 17 Oct. 2020, *LWZ 20201017-26* (HMAS 256442); THC: on dead *Pinus*, 18 Sept. 2021, *LWZ 20210918-2b* (HMAS 256479), on fallen branch of angiosperm, 18 Sept. 2021, *LWZ 20210918-4a* (HMAS 256482).

*Trechisporales*

*Hydnodontaceae*

*Trechispora*

***Trechispora confinis***

Specimen examined: DBS: on fallen branch of *Picea*, 20 Sept. 2021, *LWZ 20210920-23b* (HMAS 256644).

***Trechispora* *constricta***

Specimen examined: JLS: on fallen trunk of *Pinus*, 24 Sept. 2021, *LWZ 20210924-30a* (HMAS 256824).

***Trechispora gracilis***

Specimens examined: DBS: on fallen branch of *Pinus*, 19 Sept. 2021, *LWZ 20210919-9a* (HMAS 256544); SZF: on fallen twig of *Pinus*, 22 Sept. 2021, *LWZ 20210922-7b* (HMAS 256717).

***Trechispora sinensis***

Specimen examined: HNDBS: on fallen twig of *Pinus*, 23 Sept. 2021, *LWZ 20210923-15b* (HMAS 256756); JGS: on fallen twig of angiosperm, 25 Sept. 2021, *LWZ 20210925-3a* (HMAS 256836), on fallen twig of angiosperm, 25 Sept. 2021, *LWZ 20210925-13b* (HMAS 256848).

***Trechispora* sp. 1**

Specimen examined: THC: on fallen branch of angiosperm, 18 Sept. 2021, *LWZ 20210918-10a* (HMAS 256494).

***Trechispora* sp. 2**

Specimen examined: WNS: on fallen twig of angiosperm, 21 Sept. 2021, *LWZ 20210921-7a* (HMAS 256661).

*Tremellomycetes*

*Tremellales*

*Tremellaceae*

*Phaeotremella*

**⦁**Phaeotremella* *foliacea***

Specimen examined: WFS: on fallen trunk of angiosperm, 16 Oct. 2020, *LWZ 20201016-8* (HMAS 256406).

*Tremella*

***Tremella* sp.**

Specimen examined: TM: on fallen branch of angiosperm, 11 Oct. 2020, *LWZ 20201011-27* (HMAS 256265).

Incertae sedis

*Sirobasidium*

***Sirobasidium* *magnum***

Specimen examined: WFS: on fallen branch of angiosperm, 16 Oct. 2020, *LWZ 20201016-17* (HMAS 256413).
