# Supplementary figures and images for: Preliminary species diversity and community phylogenetics of wood-inhabiting basidiomycetous fungi in the Dabie Mountains, Central China reveal unexpected richness
Source: IMA Fungus. 2023 Nov 14;14:23. doi: 10.1186/s43008-023-00130-9 (PMC10644440; doi:10.1186/s43008-023-00130-9)

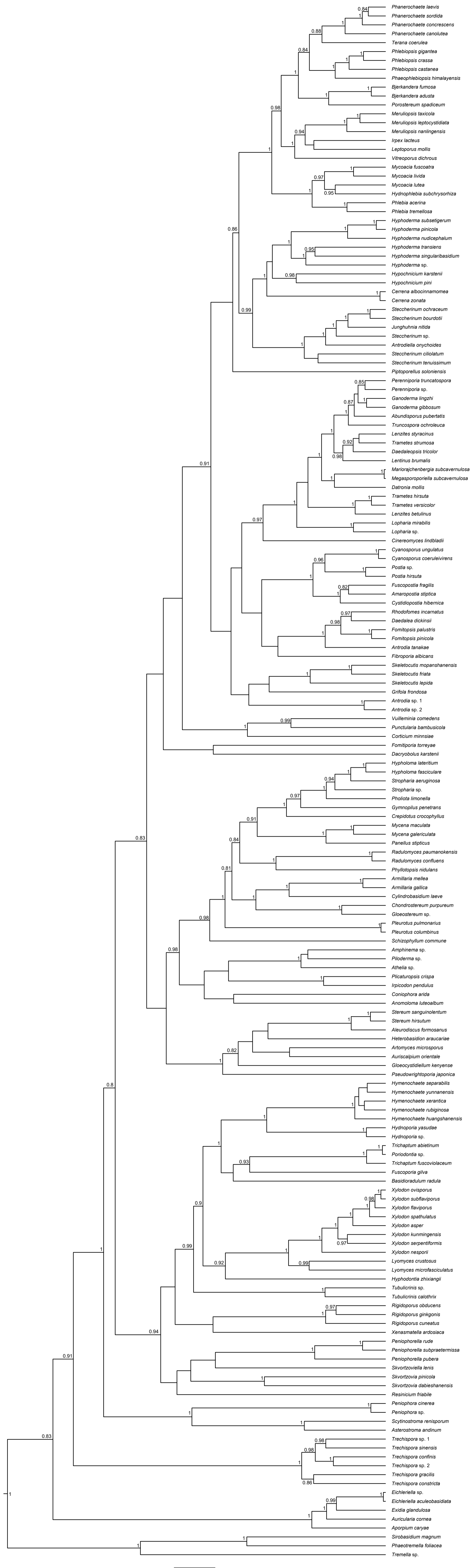

Supplement: Supplementary file 6 — Additional file 6. The phylogenetic relationships among the 175 wood-inhabiting basidiomycetous species in the Dabie Mountains. The maximum-clade-credibility tree was inferred from ITS and nLSU regions. The Bayesian posterior probabilities above 0.8 are labeled at the nodes. [file 43008_2023_130_MOESM6_ESM.pdf]
